# Active textiles move in confined areas

**DOI:** 10.1126/sciadv.aee7156

**Published:** 2026-09-25

**Authors:** Maija Vaara, Pedro E. S. Silva, Jaana Vapaavuori

**Affiliations:** Department of Chemistry and Materials Science, School of Chemical Engineering, Aalto University, Kemistintie 1, Espoo, 02150, Finland.

## Abstract

Integrating shape-morphing materials into higher hierarchical level architectures can enable programmable, complex behaviors, but it remains unclear how thread architecture can turn local contraction into coordinated motion without changing the textiles’ overall shape. Here, we demonstrate that embedding contracting threads into the versatile topologies of bobbin lace creates reconfigurable textiles that operate within fixed boundaries. Varying the mix of passive and active threads, their routing, and node placement makes it possible to transmit, cancel, or redirect motion. When lace architectures are classified by ground type, further regularities can be identified. While thread materials determine amplitude, activation thresholds, and multifunctional response, the rules governing pattern motion are primarily geometric. These design considerations can program permeability, reversible contacts, and other related responses across fabrics, establishing lace topology as a design strategy for reconfigurable textiles as well as soft devices and robots built from them.

## INTRODUCTION

Recent advances in soft materials have led to the development of smart textiles that respond to external inputs through sensing, communication, or mechanical transformation (*1*, *2*). Among them, active textiles represent a growing class of structures whose ability to undergo controlled shape changes offers new opportunities for programmable and adaptive fabric systems. When embedded through textile-crafting techniques, active fibers shrink, bend, and twist upon activation, modifying the fabric’s porosity, drape, global shape, transparency, visual appearance, and haptic feel (*3*–*6*). The actuation can be introduced by integrating stimuli-responsive components directly into the fabric, including shape-memory alloys (*7*–*9*), shape-memory polymers (*10*–*12*), pneumatic actuators (*13*–*15*), hygroscopic composites (*16*–*18*), electroactive polymers (*19*–*21*), and liquid crystalline elastomers (LCEs) (*3*, *22*–*26*). When integrating these active components within a structure that restricts motion, the resulting mechanical constraints will often suppress the desired actuation response (*3*, *27*). New design strategies are needed to enable localized and programmable deformations while preserving the structural integrity and overall function of the textile.

Here, we investigate the kinematics of active threads in textiles with fixed boundaries. By imposing such constraints, we explore designs that rely on local actuation, enabling controlled and reversible shape changes. We focus on a particular type of crafting technique, bobbin lace, which provides a wealth of topologically diverse motifs (*28*, *29*). Historically, bobbin lace was designed to create static decorative patterns by suppressing thread movement and elasticity, often using materials that resist deformation (metals, linen, and cotton) (*30*). In contrast, we show that introducing active threads turns rule-based constraints into design variables for controlled reversible shape change. We first identify the geometric and mechanical rules imposed by the lacing process within ready-made patterns, establishing the design principles and experimental context in which we explore responsive bobbin lace pattern designs. We then critically reassess traditional pattern choices, showing how they impart new functionalities within a rigid frame. Next, we quantify the changes in tessellation induced by the actuation, focusing on the porosity of the fabric. The experiments are further analyzed through simulations to uncover in detail the reversibility of deformations. Last, we demonstrate three device-level functions in lace: (i) capture under actuation, where the mesh tightens and traps millimeter-scaled spheres; (ii) release under actuation, in which pores open and free trapped spheres; and (iii) an actuation-gated electrical contact, in which conductive threads are brought into and out of contact to switch a circuit. Together, these results establish geometry-based design rules (routing, turning nodes, and activation placement) that govern boundary-constrained reconfiguration and enable programmable permeability and connectivity, and related functions, in active textiles.

### Design principles of responsive laces

**Fig. 1.**
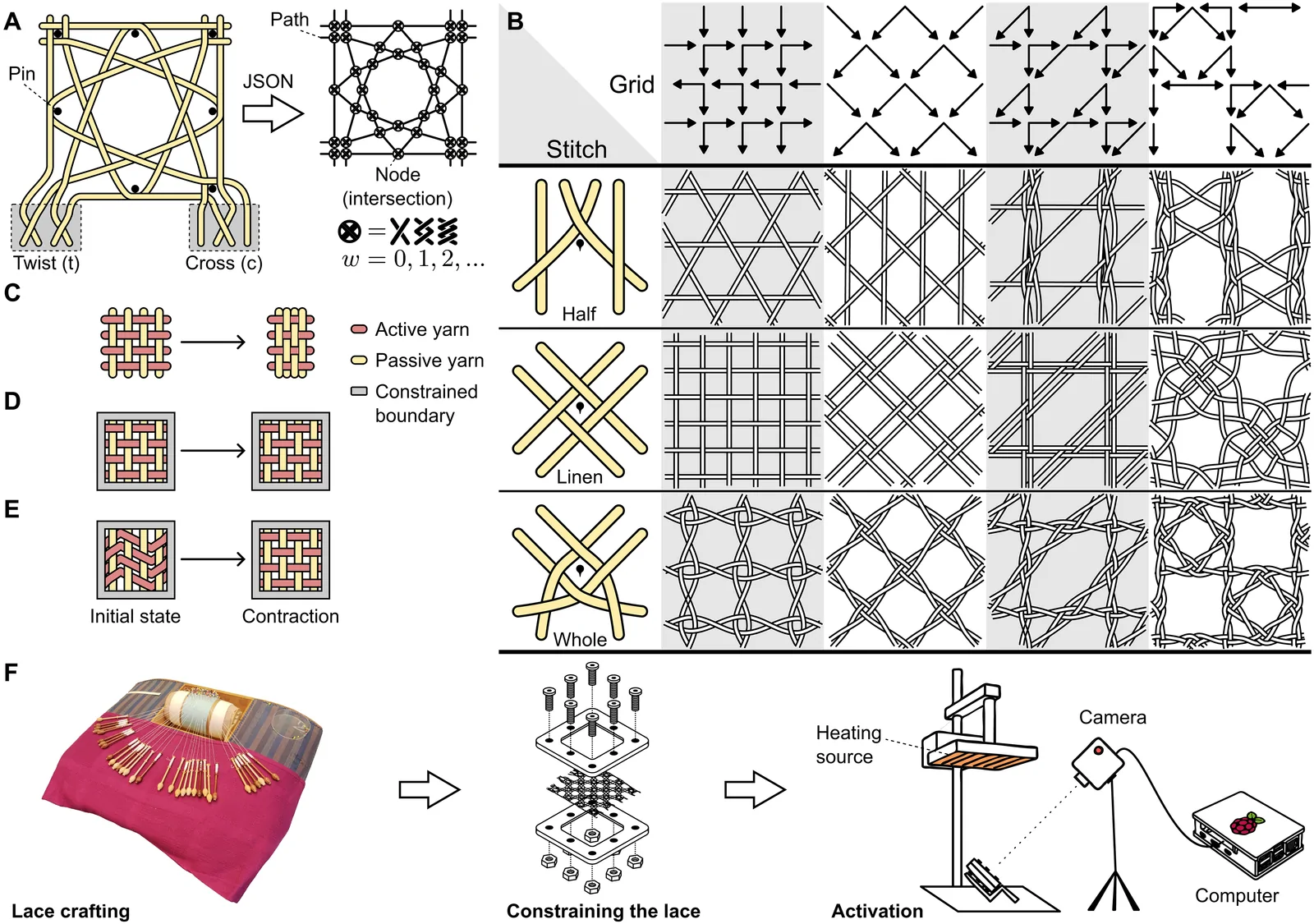
Bobbin lace textiles with active threads and constrained boundaries. (**A**) Bobbin lace basics: Two pairs of threads are systematically crossed and twisted, forming a repetitive pattern, the ground. Pins act as rigid anchors, holding threads in specific positions. Each lace ground can be defined by a set of parameters stored in a JSON file, which describes the intersection positions where a pair of threads intersects and winds around each other (nodes) and unit paths (figs. S9 and S10). (**B**) Diversity of laces: Pairs of threads, presented as arrows, travel along a grid, where at each point two arrows enter and two exit. The stitch at each point of the grid can also change, for example, half (ct), linen (ctc), and whole (ctt). Pins indicate the location of the anchoring points. (**C** to **E**) Conceptual schematic of constrained active-passive textiles: (C) A plain weave freely contracts along the direction of the active threads when actuated; (D) when constrained, the same plain weave constrained within a rigid boundary cannot contract upon activation; (E) more complex textile patterns, as those found in laces, can still undergo changes in the overall geometry even when contraction at the boundaries is restricted. (**F**) Experimental setup: The lace is first crafted, then constrained within a 50 mm–by–50 mm frame, and the visualization is recorded upon activation of the sample.

This work establishes fundamental design principles to enhance the performance of active textiles by leveraging the knowledge of traditional crafts, specifically bobbin lace. While often used for decorative purposes, lace motifs have an intrinsic mechanical richness due to their construction (*31*–*33*). Bobbin lace is a textile craft technique that combines features of weaving and braiding: Each thread interlaces with another by alternately passing over and under, forming a stable network. Bobbin lace is made by manipulating two pairs of threads to perform the following operations (Fig. 1A): twist, each pair of threads winds around its own axis, preserving the pair; or cross, the central threads of adjacent pairs interlace. The combination of these operations defines different stitches (Fig. 1B) (*34*).

Lace making allows for control of the shape of individual thread paths, guided by freely placed anchor points, pins. For comparison, the more commonly used weaving has often only straight thread paths, but in bobbin lace, a different kind of meandering is possible. This structural freedom enables a wide range of topologies, with high variability in density (porosity), symmetry, and connectivity (*31*, *35*). In theory, there is an infinite number of self-repeating lace patterns, also known as grounds, which are repeatable meshes, traditionally used to fill the space between the main motifs of elaborate figured laces (*30*). They can be sorted on the basis of grid geometry, stitch type, and anchoring configuration, as highlighted in Fig. 1B.

In most active textiles, actuation is achieved by using active threads that shrink or expand in response to an external stimulus, while passive threads provide structure (*3*). This behavior relies on the fabric’s ability to deform freely, without rigid boundary constraints, as illustrated in Fig. 1C. However, when the same textile is clamped along its edges, this global contraction is largely suppressed (Fig. 1D), and the macroscopic strain from active threads cannot develop despite the internal activation. Our hypothesis is that more complex geometries, such as the ones found in laces, can still undergo meaningful deformation under similar constraints by rearranging the pattern through thread sliding, elasticity, and geometric freedoms intrinsic to the motif (Fig. 1E).

To test this hypothesis, we fabricated a series of lace samples adapting patterns from the seminal bobbin lace book *Gründe mit System* (*36*). Different motifs were selected to investigate the orientation and shape of the thread paths, the types of crossings, the aspect ratio of the pores in the common repeat, as well as the density and location of passive/active threads. We focused on simple patterns to make it easier to differentiate between variables affecting the movement. As the first design consideration, a thread that is straight and secured at both ends can no longer be shortened. However, activating this type of thread can still affect effective stiffness and load distribution within the thread network. Consequently, when a thread is longer than the distance between its attachment points, it can shrink to at most the length of the distance between the attachment points.

To structure our pattern exploration, the first objective was to map the properties of simple repetitive structures based on traditional motifs called half, linen, and whole stitches and their combinations (Fig. 1B). Each of these motifs is formed using four threads by either crossing the middle threads or twisting them together as two pairs (Fig. 1A). In a grid with only horizontal and vertical thread paths (i.e., the first column in Fig. 1B), the structure made with linen stitch reduces to the plain weave. The half stitch omits one crossing relative to the linen stitch, yielding threads in three orientations, and an open structure. The whole stitch introduces an additional twist between crossings, leading to a structure with threads following more closely the grid.

Each experimental sample combined passive mercerized two-ply cotton with active shrinking threads, pemotex or LCE. Mercerized cotton has a smooth surface to facilitate sliding, and it is commercially available in the same thickness with active threads. Pemotex is a commercial polyester thread that undergoes strong permanent (irreversible) contraction when exposed to heat and was used in most prototypes due to its availability and large shrinkage. The self-fabricated LCE thread contracts on heating and returns to its original length on cooling (reversible actuation) (*3*, *22*–*25*). We fabricated structures using only active threads or combinations of active and passive threads. The goal of this initial exploration was to study how different lace topologies accommodate contraction and redistribute internal strain under actuation. The experimental setup is illustrated in Fig. 1F. Following crafting, each lace was attached to a rigid frame with a 50 mm–by–50 mm window and exposed to a thermal stimulus ($>90^{\circ }C$) to trigger active threads. The resulting deformation dynamics was recorded with a camera aligned normal to the sample plane. Next, we explore the transformation of the laces under identical loading and constraints.

## RESULTS

### Actuation across active lace architectures

In a moving lace within a rigid frame, deformation is set by how active threads are routed, namely, their path orientation, crossing density, and pinning. We explored periodic grids (repeating patterns), where the complexity scales with the size of the repeating motif. On a larger scale, individual pairs follow the grids that serve as the basis of the structure and determine where the pairs meet and where to place the pins as anchor points. In its simplest form, the grid is a regular lattice (*28*).

Standard rules govern thread paths in these grids. At each node (intersection), each line presents a pair of threads and two lines enter and two depart as represented with arrows in Fig. 1B. Each path may continue straight or turn (vertical, diagonal, or horizontal) but does not cross or overlap except at nodes (*37*). When a path passes straight through an intersection node, anchor points have little effect on the local geometry. When a path turns, the anchor point opens the stitch, increasing the airiness of the structure. For the sake of simplicity, in this work, we use grids based on vertical, horizontal, and 45° lines. A simple way to vary the bobbin lace structure is to repeat the same stitch on different grids (Fig. 1B). Alternatively, different stitches can also be combined on a fixed grid, which greatly expands the design space (fig. S4).

**Fig. 2.**
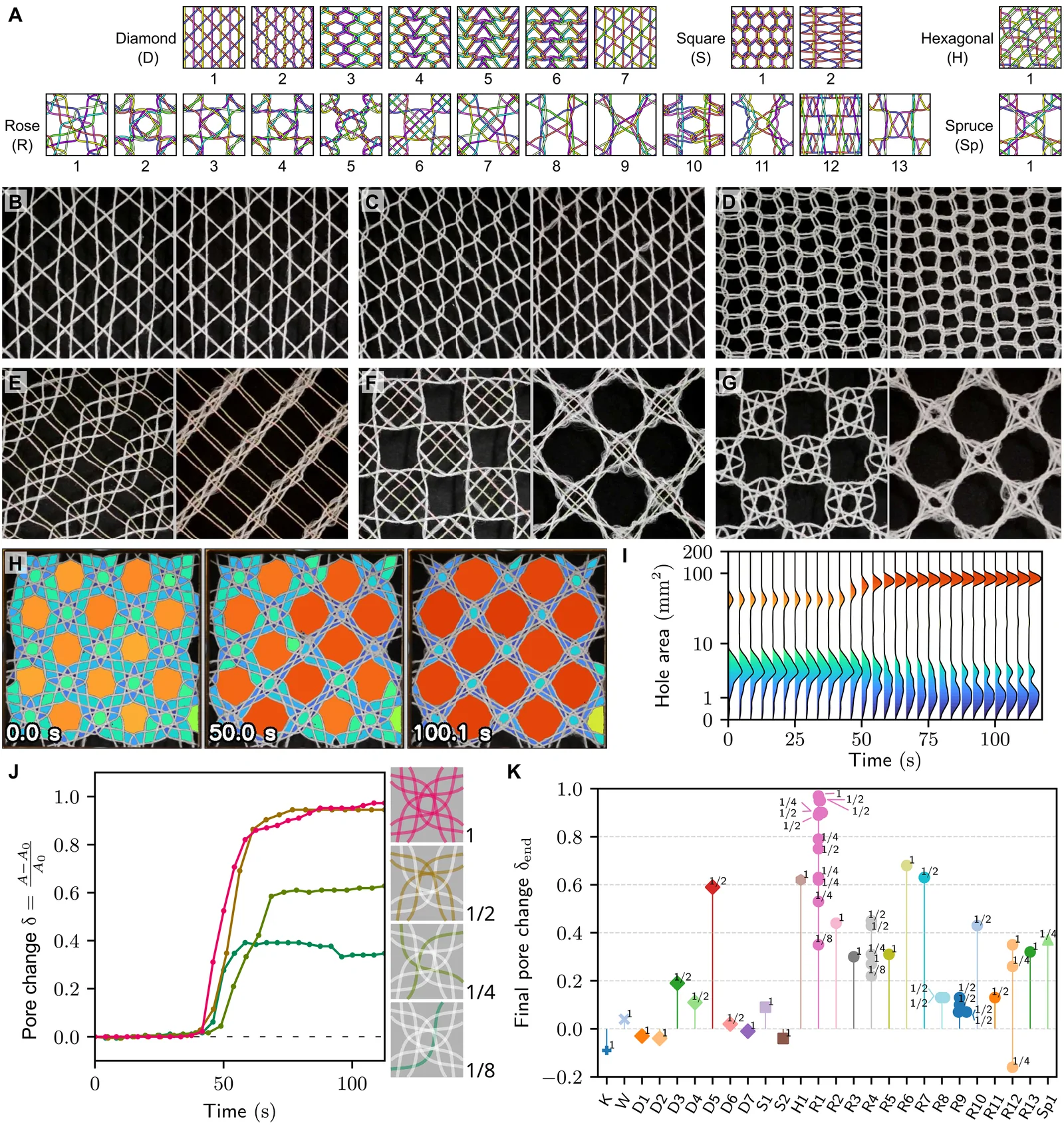
Response of boundary-constrained active laces. (**A**) Library of 24 lace architectures grouped by grounds. (**B** to **G**) Snapshots of six patterns before (left) and after activation (right). (B) Diamond-1. (C) Diamond-7. (D) Square-1. (E) Hexagonal-1. (F) Rose-6. (G) Rose-5. (**H**) Pore maps highlighting area distribution (color map log-scaled) and (**I**) waterfall plot of pore area distributions over time for the Rose-1 pattern. Lace total area is 50 mm by 50 mm. (**J**) Temporal evolution of the biggest pores, extracted from the density distribution peaks in (I), for four Rose-1 laces with different active/total threads ratios. (**K**) Final relative pore area change across all lace patterns, $\delta_{\text{end}}$. Each marker is labeled by active/total threads ratio. Horizontal jitter was added to markers for clarity.

Our experiments comprised 24 distinct lace structures spanning five grounds, diamond (D), square (S), hexagonal (H), rose (R), and spruce (Sp), highlighted in Fig. 2A, along with iterations of selected lace structures to test the effect of varying the number and placement of active pemotex threads relative to passive threads. All samples were activated with a radiant heater, and the motion was recorded on video, as showcased by before and after snapshots in Fig. 2 (B to G). We quantified the pore dynamics from videos using a segmentation algorithm, which identified the pores between the threads, and then performed a distribution-based analysis (details in the Supplementary Materials). Each video was cropped to a region of interest, converted to high-contrast grayscale, thresholded to isolate thread crossings, and cleaned with erosion/dilation operations to remove specks. We then detected all closed contours (pores between threads), converted their pixel areas to real units, and time-stamped the results. Because of intrinsic problems related with tracking changes when pores become small and fibrillation of the pemotex, smaller pores cannot be disambiguated reliably. Therefore, each frame was treated as an independent area distribution. Pore areas, *A*, were transformed to ${\text{log}}_{10}\left(A+1\right)$ to stretch the low-area and compress the high-area regions, improving the separation of pores with smaller areas and grouping equivalent bigger peaks at larger areas, and the unit offset avoids negative values. A weighted Epanechnikov kernel density estimate was then fit with weights proportional to the *A*, emphasizing pores that contribute more to the total open area. We followed the dominant density peak(s) over time and reported their relative change $\delta =\left(A-A_{0}\right)/A_{0}$, where $A_{0}$ is the area value at the beginning of the recording. All the analyzed samples are documented in the Supplementary Materials.

Figure 2H illustrates the pore detection algorithm through different instants of an example experiment: Each empty space between the threads of the sample is segmented (in color) and measured. In all panels, pores are colored by log-scaled areas using the Turbo color map with a consistent scale following the logarithmic transformation: $\text{color}={\text{log}}_{10}\left(A+1\right)/{\text{log}}_{10}\left(A_{\text{max}}\right)$, where *A* is the pore area and $A_{\text{max}}$ is a user-defined variable that sets the upper limit of the expected largest area observed and kept fixed for all experiments. Figure 2I displays the distribution of pore sizes over time in a waterfall diagram, enabling direct visualization of growth and shrinkage trends of different regions. The color coding matches the one used in the video overlays. Because the largest pores dominate the visual appearance of the structure and can be used for the first estimate for applicability (e.g., openness and permeability), we track the largest-pore peak for cross-experiment comparison. Figure 2J illustrates this comparison for a single geometry with varying active-thread ratios (1, 1/2, 1/4, and 1/8).

Figure 2K extends the comparison between all experiments and reveals a wide variety of behaviors. Samples without visual changes of pore sizes share common design features: In structures, for instance the one shown in Fig. 2B where all threads are straight, contraction cannot develop because threads have no space available to shrink. In structures, illustrated in Fig. 2 (C and D), where the meandering threads are tightly interwoven at curvature points, interlocking suppresses sliding. In contrast, some samples show their largest pores increasing significantly upon activation, as shown in Fig. 2 (E to G). Here, active threads integrated into the structure allow movement within the motif.

In the case of all threads in a lace being active, outcomes range from no change to significant area and/or shape changes. The placement of active threads plays a significant role in determining the change in pore size upon activation. Pores are always bounded by the surrounding threads, and if the threads on the edge of the pore are active and curved, the pores can expand. This can be clearly seen in the pattern Rose-1 or Rose-6 (Fig. 2, H and F). Overall, two results stand out with patterns blending active and passive threads: Some blends can match the actuation of the fully active case (e.g., Rose-1 achieves similar results with 1/2 or even 1/4 of the threads activated; Fig. 2K), whereas some combinations can outperform the fully active case (e.g., Rose-4 displays a larger areal change for two different 1/2 blends than the fully active sample).

As our main observation, the mobility of the pattern upon activation does not reduce into linear influence of any of the pattern design variables. The number of crossings and twists varies among different structures but alone does not correlate with the structure’s mobility. For example, Rose-1 and Rose-6 have the same number of them but exhibit distinct responses (Fig. 2K). From the perspective of a single thread within a pattern, shrinkage capacity depends on two variables: the length of the thread compared to the distance between the attachment points at its ends and the freedom at turning points. No single design variable solely determines these thread properties; however, in a grid composed only of straight lines traversing the entire structure, changing stitches does not create sufficient freedom for motion. By contrast, turning nodes increase the mobility of threads. Since there are infinite number of potential ground designs that can enable “shrinkable paths” for the threads, we next turn into computational analysis to map the feasibility of large number of different lace tesselations.

### Computational modeling and reversibility

**Fig. 3.**
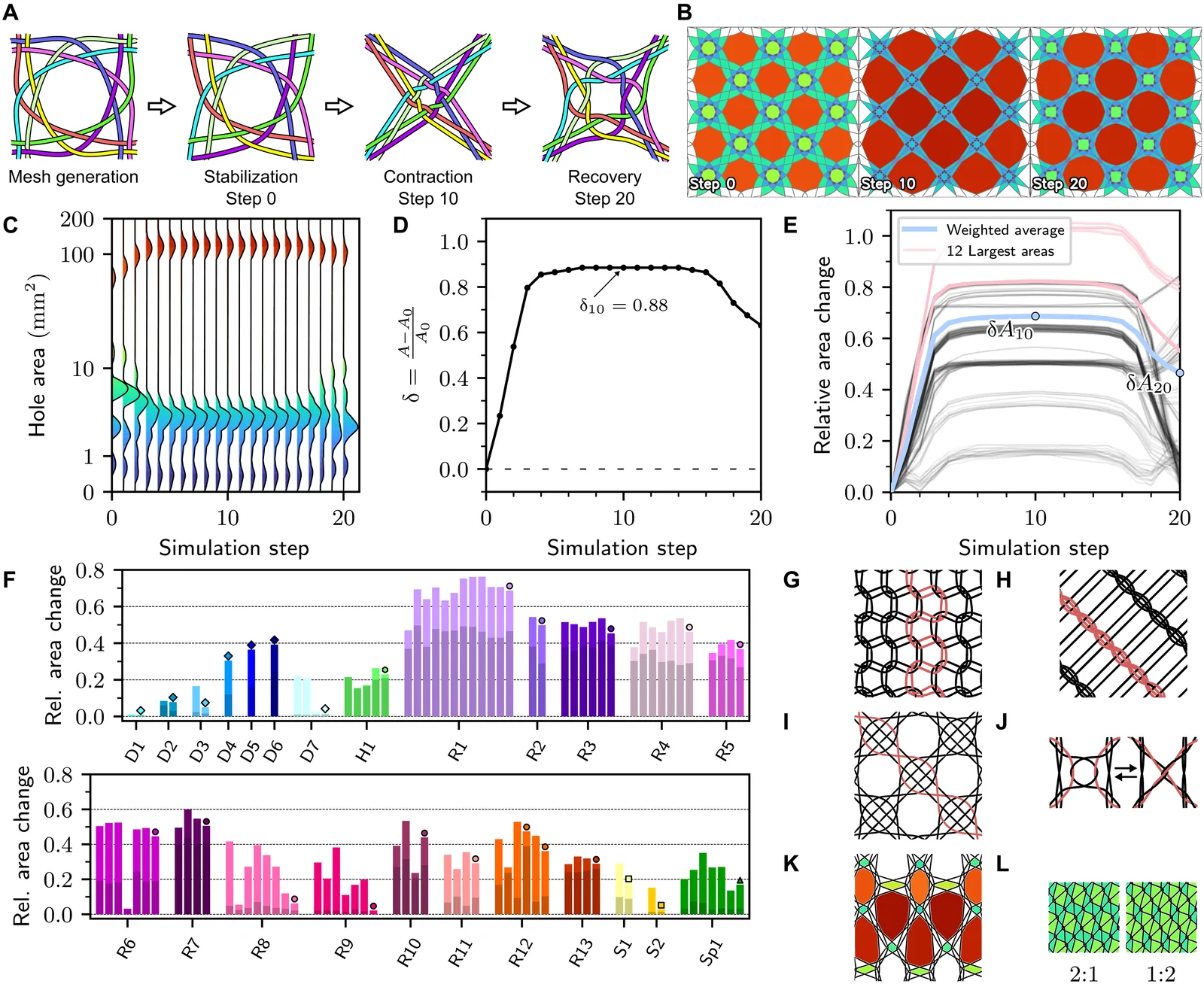
Simulations and reversibility of active constrained laces. (**A**) Overview of the simulation protocol: mesh generation—the lace JSON file is converted into an input format for LAMMPS; stabilization—energy minimization and relaxation of the initial mesh; contraction—active threads are shrunk by 25%; recovery—contraction stress is removed from active threads. (**B** to **E**) Simulation results for the Rose-1 with only active threads: (B) pore maps highlighting area distribution at different simulations steps (color map log-scaled); (C) waterfall plot of pore area distributions at each simulation step; (D) evolution of the largest pores, extracted from the density distribution peaks; (E) relative pore area change. Area size is 50 mm by 50 mm. (**F**) Summary of all simulated patterns: bars displaying area change under maximum stress $\delta A_{10}$ (light color) and final relative area change after stress release $\delta A_{20}$ (dark color), where values farther from 0 are less reversible. (**G** to **L**) Key design principles: (G) patterns with vertical/horizontal S-shaped threads tend to lock under full contraction (Square-1); (H) patterns with diagonal S-shaped allows contraction when all threads are activated (Rose-6); (I) patterns lacking threads opposing contraction tend to recover poorly (Hexa-1); (J) some stitches, like cttc, allow pores to fully close and making threads apart become into contact, reversibly (Rose-12); (K) while certain geometries produce complex pore distributions (Rose-8); (L) others allow control over pore distributions, for example, small:large pore ratio by activating different threads (Diamond-7).

The experimental results display a wide range of deformations achieved under boundary-constrained activation. Further understanding of the different mechanisms governing these responses and, additionally, reversibility requires a controlled and trackable system. Therefore, a minimal computation was developed to simulate active laces under geometric constraints (Fig. 3A). In this model, threads are represented as elastic filaments with stretching and bending rigidities, and a soft repulsive potential prevents the overlap of the filaments. All threads are modeled as frictionless, providing an idealized upper bound on mobility and a clean baseline, to which more detailed frictional models can be compared. Despite these simplifications, the computation captures the key deformations and enables a systematic exploration of the constraints imposed by the different lace architectures and recovery behaviors. Full simulation details are provided in the Supplementary Materials.

Lace patterns are directly constructed from custom JSON files and converted into readable LAMMPS inputs (*38*). The simulation protocol has two stages: (i) energy minimization to allow mesh reorganization and tightening under the imposed constraints and (ii) a quasi-static actuation in which designated active threads undergo a 25% reduction of their base length in 10 steps, followed by an additional 10-step reversal to assess recovery.

The simulation workflow enables selective activation of any subset of threads and, from the resulting thread positions, extracts two quantities, adding further insights to the experiments: thread length and pore area. For each simulation, the lace is projected onto the *xy* plane, and all areas between the threads that define closed contours are assigned a persistent label so that pores can be followed at every step (figs. S15 and S16). In Fig. 3B, the pores are color-coded by area using the same logarithmic map as in the experiments [$\text{color}={\text{log}}_{10}\left(A+1\right)/{\text{log}}_{10}\left(A_{\text{max}}\right)$].

Comparing with the example case shown in Fig. 2 (H to J), Rose-1 with 8/8 active threads, the simulation reproduces the experimentally observed contraction, (Fig. 3, B to E). Figure 3B shows snapshots of the color-coded pore map where, upon activation, steps 0 to 10, the pattern dynamics displays pores shrinking (green to blue) and dilating (orange to red), mirroring the behavior in Fig. 2H. The distribution analysis in Fig. 3 (C and D) complements the visual analysis, although there are some small differences in the lowest-area regions in comparison with the experimental results. These differences can be linked to the areal resolution of data between experiments and simulations and small discrepancies in the initial geometry of the physical and digital laces. Simulations also enable plotting a pore-wise relative area change (Fig. 3E): Each line tracks a single pore through actuation and recovery. Figure 3D captures global variation through the dominant peak of the areal distribution, where Fig. 3E shows the individual behavior of all individual pores. The growth of the largest pores in Fig. 3D is consistent with the pink curves (Fig. 3E), which report the individual relative area changes of the largest pores.

Rose patterns show diverse reversibility behavior for contraction upon activation. In Fig. 3F, each motif is grouped by different sets of experiments; each light color bar corresponds to maximum contraction $\delta A_{10}$ and darker color bar to recovery $\delta A_{20}$. Ideally, a good performance corresponds to tall light and short dark bars. Grouping by the dominant path routing, vertical-threaded variants exhibit the best recovery (e.g., Rose-8, Rose-9, and Rose-12 achieve best $\delta A_{10}=${41.6%, 38.2%, 52.8%} with $\delta A_{20}=\left\{4.9\%,\;2.3\%,\;9.3\%\right\}$, respectively), whereas diagonal-threaded variants undergo larger areal change but recover less (e.g., Rose-1 and Rose-4 achieve best $\delta A_{10}=\left\{76.2\%,\;53.6\%\right\}$ with $\delta A_{20}=\left\{46.3\%,\;28.1\%\right\}$, respectively). Hence, by modifying the locations of twists and crossings in patterns, one can create motifs that both move significantly and restore well. It is noteworthy that even when a pattern does not fully return to its initial shape, it can still deliver substantial reconfiguration under activation. The required degree of recovery is ultimately application dependent, where different use cases value different trade-offs, like contraction amplitude, stability, or autonomous resetting.

Figure 3 (G to L) compiles the main observed behaviors across different patterns, thus establishing a toolbox of motifs that also can be combined into higher level lace constructions. The following examples illustrate the structure’s ability to change and return to its original shape, when at least some of its threads meander. Case G in Fig. 3, vertical cancellation: Although all threads meander in their paths, the active-thread routing in Square-1 describes a vertical S between the boundaries. Upon activation, all curved segments pull against each other, so the resulting contraction is greatly affected. The result is local tightening at the turning nodes with minimal pore area change and reduced net shortening. Case H in Fig. 3, hybrid diagonal contraction: Hexa-1 combines oblique straight paths in one direction with curved paths in the other. Under activation, the curved segments can freely slide and contract at nodes, allowing the structure to change its shape. However, no counteracting threads oppose the shrinkage, thus the moving threads do not retrace their sliding, so recovery to the original state is poor. Case I in Fig. 3, diagonal contraction: Contrasting with the previous case, the active routing in Rose-6 follows oblique paths, allowing the contraction and sliding in different intersections. The bigger pores increase their sizes at the cost of the coalescence of smaller ones.

Among the tested structures, a few individual cases emerged, whose properties are particularly interesting. Case J in Fig. 3, reversible thread-thread contact/closure: Some stitches, such as “cross, double twist, cross” sequence used in Rose-12, activation not only closes the pore in the center but also brings previously distant threads into contact. On relaxation, the effect reverses opening the pore and the contacting threads separate. This mechanism is purely geometric; however, with conductive threads, the same motion can provide a stimulus-gated electrical contact suitable for simple textile logic gates. Case K in Fig. 3, symmetry and route length: Patterns with diagonal routing, Rose-1 to Rose-7, have fourfold rotational symmetry, whereas those with vertical routing, Rose-8 to Rose-13, have twofold only. Fewer axes of symmetry seem to result in greater variation in individual thread paths within a motif. If we count how many motifs a single thread must pass through before it begins to repeat the same route, patterns with only two axes of symmetry stand out. For Rose-8 (Fig. 3K), an individual thread repeats only after two motifs horizontally and up to six vertically. In this setup, the thread does not have space to repeat any previously traveled path in terms of the shape, so placement of active threads can significantly alter the activated geometry and result more complex pore distributions and orientations. Case L in Fig. 3, selective pore partition: Diamond-7 highlights some unique cases, specifically in this example, where active/passive blends yield pores with controllable area ratios. In this pattern, threads traveling right to left (A) are straight, while left to right threads (B) meander and loop around adjacent neighbors. Activating a single B thread contracts it and shrinks the pores between itself and adjacent threads. By controlling which B threads are active, the lattice partitions into repeating large/small pores with predictable proportions. In our tests, activating every third (1-in-3) and two of every thirds (2-in-3) produced 2:1 and 1:2 large:small pore patterns, respectively. Taken the design outcomes together, we select new motifs that are relevant for different tasks and demonstrate corresponding applications.

The resulting motif diversity extends beyond conventional textile crafts, namely, weaving, knitting, and braiding: the freedom of the technique to allow complex thread routing and placement generate responses not accessible to less flexible techniques. Furthermore, the simulation enables a faster screening by exploring multiple possibilities of selective activation and placements and mapping the response landscape, narrowing candidates of interest before fabrication. Guided by these outcomes, we designed motifs targeted to specific functions and next demonstrate application prototypes.

### Applications

Through both our experimental and computational analysis, we have shown that different categories of active lace patterns operating under rigid boundary conditions can be created. Overall, these patterns open up a new class of active textiles, which can deliver significant morphological and functional changes within a rigid frame, thus differing from the active textiles presented so far. Since different applications call for different changes in properties, the establishment and optimization of structure-property relationships only become meaningful when a specific application context is chosen. Here, two conceptually different applications, highlighting wide-ranging relevance, are showcased in detail.

**Fig. 4.**
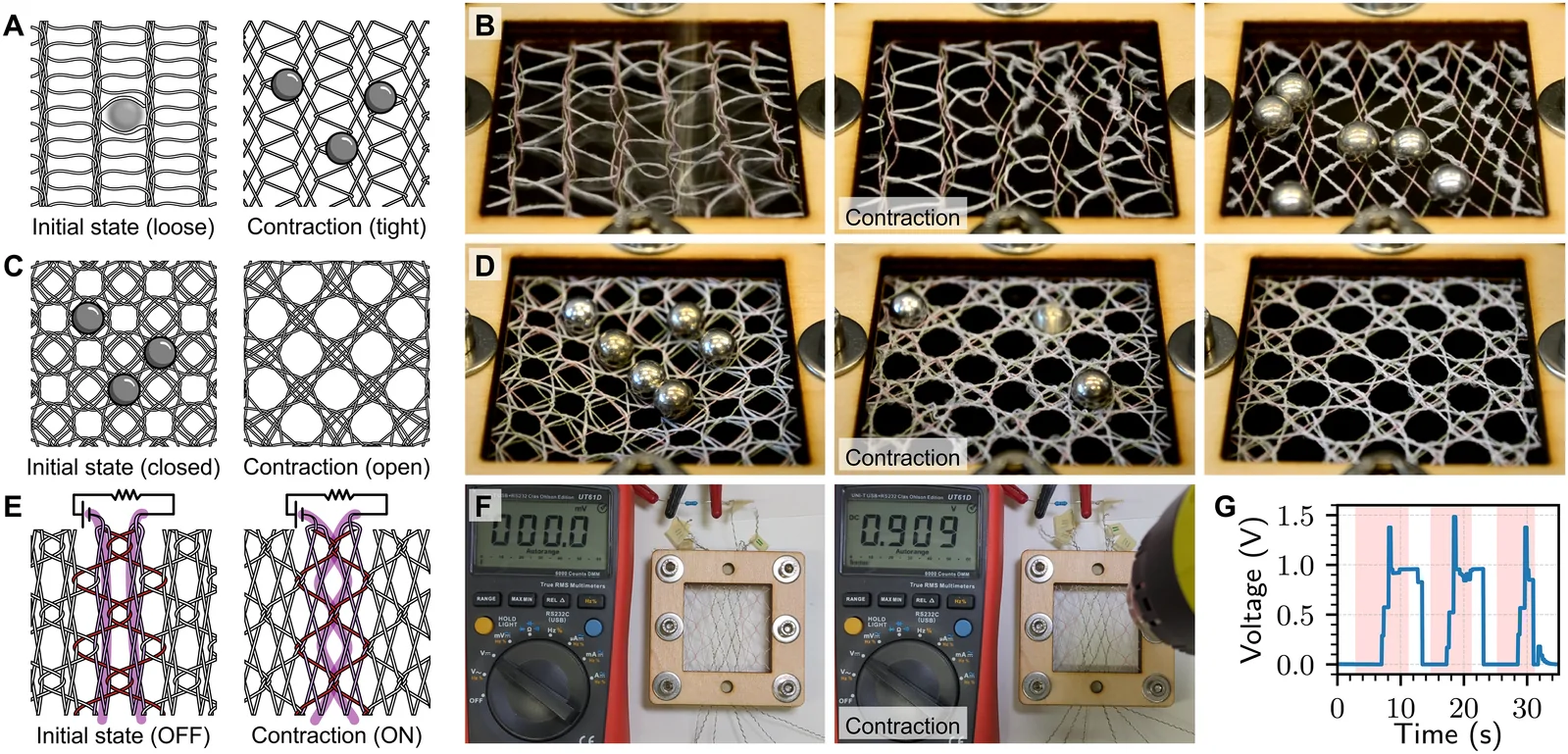
Programmable filters and logic textiles. (**A** and **B**) Capture under activation: active threads tighten the network, narrowing pores and arresting passing spheres. (A) Schematic of the tightening mechanism. (B) Video stills: passing spheres, thread activation (pemotex), and spheres capture. (**C** and **D**) Release under activation: Activation opens pore pathways, creating channels that free previously trapped spheres. (C) Schematic of pore opening. (D) Video stills: trapped spheres, thread activation (pemotex), and spheres release. (**E** to **G**) Actuation-gated electrical contact: Active threads drive conductive thread pairs into/out of contact to realize an OR logic element. (E) Schematic: either one of the contacts, top, middle, or bottom, closes the circuit. (F) Stills with voltmeter readout toggling between OFF (0 V) and ON (∼1 V) upon recovery/activation of active threads (LCE). (G) Voltage versus time across repeated cycles (activation windows in red background).

The potential of our textiles as highly customizable active filters was tested by attaching the textile to the same wooden frame previously used in this work and dropping balls (6.3 mm and 0.35 g) onto it. We prepared two types of functional textile filters: capture and release under activation. The closing filter structure shown in Fig. 4 (A and B) is a modified structure Rose-12. Before actuation, the active threads are loose, and passive threads are straight and close to each other, so balls can easily pass through. When activated, the contracting threads pull the passive threads apart, and their braid-like structure opens up. To realize an opening filter, shown in Fig. 4 (C and D), we slightly modified Rose-6 by placing the straight threads more evenly within the structure, and it did not allow any balls to pass through before activation but all fell through after. More uniform distances between straight threads significantly enhance the performance of the lace when used as a filter. Straight threads at the edges of pores strengthen the structure and prevent particles from passing through the textile before it is activated.

Considering the future applicability of these patterns as filters, the intriguing addition is the active nature of the filtering—i.e., the cutoff size can be adjusted rapidly, when needed. Another advantage is related to the possibility to add structure supporting and additional functionality providing threads as a part of the network. This could enable, for instance, active filters that can clean themselves upon activation of the selective threads that scrape the deposits from the top of the filter. The size can be scaled both smaller and larger, with the only limiting factor being the thickness of the available threads.

As an example of other possible applications, we tested using the Rose-12 structure as a switch. As we have previously demonstrated (Fig. 3, E to G), in this structure, active threads can pull passive ones into contact with each other. When we used LCE thread as the active component, repeated heating and cooling reversibly pulled the conductive silver-coated wires into contact and separate it, producing clear ON/OFF transitions in the measured voltage. The LCE-induced tension is sufficient to maintain stable contact under the operating conditions. This demonstration establishes a programmable textile logic element in which material responsiveness and electrical switching are integrated purely via the textile architecture, without discrete rigid components and under a constrained topology. LCE could be replaced with any other stimuli-responsive contracting thread type, such as hygromorphic over-twisted cellulose fibers (*39*). This approach allows developing context-aware e-textile circuits capable of computational reconfiguration, which can allow preparing new types of privacy-preserving textiles capable of sensing and giving haptic feedback completely without integrated microcontrollers and external cloud-based information processing, thus requiring only a fraction of electric and computational power of today’s e-textiles.

## DISCUSSION

As showcased by our work, bobbin lace offers a versatile and rule-based approach to programmable textiles. The grid geometry of the pattern sets the playground for designing thread paths and intersection locations; anchoring points (pins) enforce the direction changes of threads within the structure; and stitches (sequences of cross and twist operation) set how pairs exchange in nodes. When a thread is contracting (or expanding), these are the main design variables in governing how actuation starts and propagates through the motif.

Under constrained boundaries, lace geometry does enable meaningful deformation via thread sliding and route reconfiguration, as hypothesized. Across our experiments, even small variations in any of the design variables significantly affected the properties of the moving textile. However, not every change is effective: Straight paths and symmetric counter-acting threads have limited motion, whereas turns near active routes enable it. Another important outcome is that not all threads need to be active to optimize overall movement. Blends of active and passive threads can match or exceed the performance of the patterns made of only active threads, when well placed (e.g., Rose-1 50 and 25% blends produced similar results as 100% active), thus showing that where the pattern is activated is more important than how many threads are activated.

Since the effects of the single design variables on the overall actuation of the pattern are highly nonlinear, the simplest way to characterize a large number of tesselations is to simulate them. Our minimal model isolates mechanisms under idealized conditions. In the simulation, threads are perfectly smooth, uniform in thickness, and have no torsional rigidity. Even so, the simulations reproduce the measured contraction pathways while enabling a precise tracking of the pore changes, measurements of shrinking and extension of threads, and local distribution of forces in the net during activation and recovery. Minor differences between experiments and simulations arise from geometrical mismatches between physical and digital laces; also, a quantitative prediction will require adding torsional rigidity, friction, actuation hysteresis, and diameter variability once target materials and operating regimes are specified. Despite its simplicity, the presented minimal model provides an adequately accurate description for exploring the diverse functional capabilities of the studied lace patterns, which is the main objective of this work

The type of response that emerges under actuation, in our experiments and simulations, can be classified into four types: (i) pore size; (ii) pore shape and orientation; (iii) tension; and (iv) reversible thread contact (e.g., pore closure and switch-like contact in Rose-12). These behaviors can be tweaked through pattern design and enable numerous applications. The idea of a simple filter presented in this work could be further developed by using material that returns to its original size after contracting. For example, in the same textile, threads that react at different temperatures (or other stimuli) could also be used, and the threads do not necessarily need to use their full contraction potential. This way, the filters would have different properties at different temperatures, and the sliding of the yarns with respect to the other yarns could be used for scraping deposits from them. It is noteworthy that it is also possible to regulate the shape of the pores, allowing the filtration of objects with different shapes, as well as making larger area laces, where different parts of the lace filter for different sizes/shapes of objects. Moreover, overlaying multiple moving lace patterns can give rise to interesting light diffraction features.

Last, to highlight the realm of other potential applications, these active laces may find uses as new control units for microfluidics. For example, some of the threads in the patterns could be replaced by microfluidic tubes, and then selective activation of the lace could lead to kinks decreasing or stopping the fluid flow. Furthermore, other soft materials could be placed in-between the active lace, the selective activation of which could then lead to controllable multimodal haptic interface for human-computer and human-robotic interfaces. The same routing and node freedom can extend to a new type of textile logic: Our actuation-gated contact forms a basic switch, but more complex series/parallel placements in combination with threads that expand upon activation (*40*) can enable other logic without leaving the lace architecture. In all cases, the geometry sets the function, while the materials tune the amplitudes and thresholds, reducing the dependence on external electronics.

In terms of functionality, designing different textile variations is easy. Since the bobbin lace technique is already industrialized (*32*, *41*, *42*), it has the potential to rise as a textile production method on side of woven, knitted, and nonwoven fabrics. As shown here, this technique’s versatility in thread positioning can enable numerous new applications beyond the capabilities of current dominant production methods. This opens exciting future possibilities for how multifunctionality embedded into textiles structure can transform the role of textiles in everyday life, for example, through wearable on-site information processing and active health navigation (including continuous monitoring and individual local feedback). Our simulations are scaleless, and it is predicted that the same design principles will work for elements of very different thickness, when the structure is scaled in the same proportion.

We demonstrate programmable reconfiguration of active textiles within fixed boundary conditions enabled by bobbin lace topologies and a simple set of design rules. Many prior textile actuation approaches assume largely unconstrained boundaries, whereas operation under fixed edges is important when moving textile needs to be fitted in predefined-sized space. Within the lace, routing and node placement with combinations of passive and active threads provide geometric design rules to transmit, cancel, or redirect motion. Selecting specific threads can result in various multifunctional outcomes, such as combining environmental responsiveness with conductivity. This geometric grammar could guide the design of textiles with programmable permeability, light transmission, reversible contacts, and related responses and could be applied to soft devices and robots built from such textiles.

## MATERIALS AND METHODS

### Bobbin lace textile sample preparation

For lace making, we used a roller pillow that is traditionally used in Rauma of Western Finland. It enables the creation of long continuous-thread laces. The model of the used bobbins is Binche (Brugge) chosen for its suitability for operating with thin threads because of its lightweight and smooth nature. The wider head of the bobbin facilitates handling and gentle tightening. As attaching points, 0.6-mm straight pins were used. The 4-mm grid used for the base was printed on paper that was then pinned on the roller pillow on top of a thin and dense cardboard (see fig. S6).

The passive yarn used was 100% gassed mercerized superior cotton Filoscozia Nm 65/2, and the chosen active yarn was approximately the same thickness of pemotex that is 100% polyester. Because of its internal structure, pemotex shrinks significantly when heated. On the edges of the laces and between samples, the threads are secured with a tight four-strand braid. Braiding is done by alternating crossing and twisting (see fig. S6).

For the application demonstrators, silver-coated nylon wire was used as the conductive thread and home-spun LCE as the active thread in logic textile. The LCE yarn was produced by ultraviolet (UV) melt spinning as reported by Lin *et al.* (*43*). A thiol-acrylate oligomer was synthesized via a two-step click reaction, loaded into a syringe, and extruded at a fixed flow rate. The extruded filament was photocrosslinked under a 365-nm UV illumination and collected on a rotating bobbin. The resulting yarns contract reversibly along their length upon heating and recover upon cooling.

Each lace was attached to a rigid frame with a 50 mm–by–50 mm window and exposed to a thermal stimulus (>90°C) with infrared heater (1500 W) placed above the sample to trigger active threads. The resulting deformation dynamics was recorded with a camera (Z6/2 Nikon) aligned normal to the sample plane.

### Lace pattern specifications for simulations

Lace patterns were translated into a custom JSON format that describes how yarns navigate between crossing points. Each pattern file is organized into four sections: nodes, which define crossing locations as two-dimensional coordinates together with a twist parameter τ that specifies whether yarns simply cross (τ = 0) or additionally wind around each other (τ > 0); paths, which define the repeating routes connecting the nodes, including shift vectors that consider additional spatial translations between repeated instances of the same node; unit_yarns, include the instructions for individual yarns following these paths, including starting position, initial over/under configuration, and the repetition vectors that tile the yarns across the whole design landscape; and roi_bonds, which set the bounding box for the simulation region. A detailed description of the JSON structure, including worked examples, is provided in the Supplementary Materials.

### Mesh generation

A Python script converted each JSON pattern file into a mesh of particles suitable for molecular dynamics simulation. First, the translation vectors between consecutive nodes in each path are computed, incorporating any user-defined shifts. For nodes with nonzero twist, auxiliary points are generated to produce a helical-like crossing geometry. Yarn points are then built along the repeat unit paths, while the *z* coordinate oscillates between a negative and positive height value at successive crossings to enforce alternating over/under interlacing. Each yarn segment is extended along its primary repetition vector and then smoothed using a parametric cubic spline (SciPy splprep; smoothing factor, 0.1), and, afterward, the curve is resampled at a uniform arc-length spacing L≈0.25 (in simulation units), chosen to match the target equilibrium bond distance. The smoothed yarns are replicated along the second repetition vector to fill the simulation region, and any particles falling outside the region of interest are discarded. Last, bonds are assigned between consecutive particles of the same yarn, and angles are defined over consecutive triplets. Particle coordinates, bonds, angles, and force field parameters are written into a file compatible with LAMMPS.

### Molecular dynamics simulations

Simulations were performed using LAMMPS with dimensionless (Lennard-Jones) units and a molecular atom style. The intraconnectivity of the yarns was modeled with harmonic bond and angle potentials, $E_{\text{bond}}=k_{\text{bond}}{\left(r-r_{0}\right)}^{2}$ and $E_{\text{angle}}=k_{\text{angle}}{\left(\theta -\theta_{0}\right)}^{2}$, where $r_{0}$ is the equilibrium distance between consecutive particles and $\theta_{0}=0$ enforces straight yarns. The interconnectivity and collision were handled by a custom soft_exclude pair potential, $E_{\text{soft}}=A\left[1+\text{cos}\left(\mathrm{\pi r}/r_{\text{cut}}\right)\right]$, with *A* = 200 and a cutoff $r_{\text{cut}}=1.0$ (equal to twist of the effective yarn radius of 0.5). This cosine potential is finite a *r* = 0, which prevents numerical divergence but might have yarns crossing each other, if too close when initiated. Pairwise interaction between particles separated by fewer than 16 bonds along the same molecule were excluded to avoid interference with the bonded potentials.

All the particles at the boundaries were frozen (forces set to zero), and all other particles were integrated using the velocity-Verlet (NVE) with a viscous damping term to dissipate kinetic energy. Nonperiodic boundary conditions were set in all directions.

Two sequential simulations were run for each pattern. In the first (stabilization) phase, the mesh was relaxed via energy minimization followed by a dynamic run, during which equilibrium bond distances were typically reduced by 4% to tighten the initial fabric configuration. In the second (actuation) phase, a combination of yarn types up to 8 was contracted by iteratively reducing their equilibrium bond distances lengths by 25% in 10 steps, followed by a reversal relaxation back to the original lengths. At each contraction step, the simulation was run for a fixed number of timesteps to allow the structure to equilibrate before proceeding to the next increment. The complete LAMMPS input scripts are provided in the Supplementary Materials and on GitHub at https://github.com/p3d2/lacemaker.

### Postprocessing of simulation data

The simulation output files were parsed to extract particle position, forces, and potential energies at each recorded timestep. The particles were sorted by their sequential identifiers to reconstruct yarn connectivity. To follow the yarn lengths, particles were grouped by their yarn (molecule) identifier, and the contour length of each yarn was computed as the sum of Euclidean distances between consecutive particles. Yarns shorter than half of the simulation box width were excluded from analysis to avoid boundary artifacts. The pore identification and tracking quantified changes in fabric porosity, so the yarn mesh was projected into the *xy* plane. In this projection, each pair of consecutive particles within a yarn was connected by a line segment and expanded into a thin rectangle with a thickness of 20% of the diameter of the yarn. This reduced buffer width was chosen because of some limitation on identifying pores in tight mesh configurations, which might have caused adjacent pores to merge or split in the two-dimensional projection. The union of all buffered segments produces closed polygons, where the interior holes of these polygons correspond to the pores of the fabric. The area of each pore was computed directly from its boundary polygon. Each pore was assigned a persistent label to follow the behavior of individual pores across simulation steps. In the first frame, every detected pore received a unique identifier. In subsequent frames, pores were matched to their previous identifier assignments by minimizing the Euclidean distance between centroids, given a maximum displacement threshold. Any pore whose centroid moved beyond this threshold was treated as a new pore and assigned a fresh label. To summarize the dynamics of the fabric response, a baseline-weighted overall curve was calculated by weighting each pore’s relative change by its initial area (bigger pores have higher impact). A loss fraction curve was also defined as the share of total detected area at each step not assigned to any traced label to monitor the reliability of the tracking algorithm.

### Video processing of physical laces

Physical lace samples were recorded during actuation, and each frame was trimmed, rescaled from pixels to millimeters, and subsampled at a fixed interval. Selected frames were converted to high-contrast gray images, thresholded to isolate bright yarn cross sections, and cleaned with morphological erosion and dilation operations to remove small artifacts. Closed contours were detected, and their enclosed areas were computed, providing a per-frame distribution of pore sizes. Because individual pores were not tracked uniquely, as in the simulations, the analysis focused on the dominant peak(s) of the log-transformed area distribution, fitted with a weighted Epanechnikov kernel density estimate. The peak position was monitored over time and converted to a relative area change. In cases where the dominant peak split during actuation, both resulting peaks were tracked independently. The complete procedure and algorithms are given in the Supplementary Materials.

## Supplementary Materials

### The PDF file includes:

Extended Materials and Methods Supplementary Results Figs. S1 to S175 Tables S1 and S2 Legends for movies S1 to S5
