# Supplementary material for: Active textiles move in confined areas
Source: Sci Adv. 2026 Sep 25;12(39):eaee7156. doi: 10.1126/sciadv.aee7156 (PMC13614380; doi:10.1126/sciadv.aee7156)
Supplement: Supplementary file 1 [file sciadv.aee7156_sm.pdf]

Science Advances
AAAS

Supplementary Materials for

Active textiles move in confined areas

Maija Vaara *et al*.

Corresponding author: Jaana Vapaavuori, jaana.vapaavuori@aalto.fi

Sci. Adv. **12**, eaee7156 (2026)
DOI: 10.1126/sciadv.aee7156

The PDF file includes:

Extended Materials and Methods
Supplementary Results
Figs. S1 to S175
Tables S1 and S2
Legends for movies S1 to S5

Other Supplementary Material for this manuscript includes the following:

Movies S1 to S5

Extend materials and methods

Bobbin lace textile sample preparation

This work does not follow the work flow of traditional handmade bobbin lace. One main difference is that the thread material may change while turning on the edge of the pattern. This is because we consider the structure to be infinite in the simulation. In the hand-made prototypes threads are swapped between edges in those cases, where turning at the edge would result in wrong material thread. This is mostly the case when pairs are traveling through the pattern and the whole pair is made of active thread. The handcrafting process allows making very small samples and uses very small amounts of thread. This is important since we are partially employing experimental materials (in our case LCEs), for which the larger-scale production is yet to be established.

Bobbin lace stitches are combinations of the intersections of two pairs of threads and they are made by moving the bobbins over each other. The right bobbin of the same pair going over its pair bobbin is called ”twist”. This is always done with two pairs simultaneously, if not mentioned otherwise. ”Crossing” takes place between two adjacent bobbins of two adjacent pairs and the bobbin on the left side moves to the right over the other pairs bobbin (*34*).

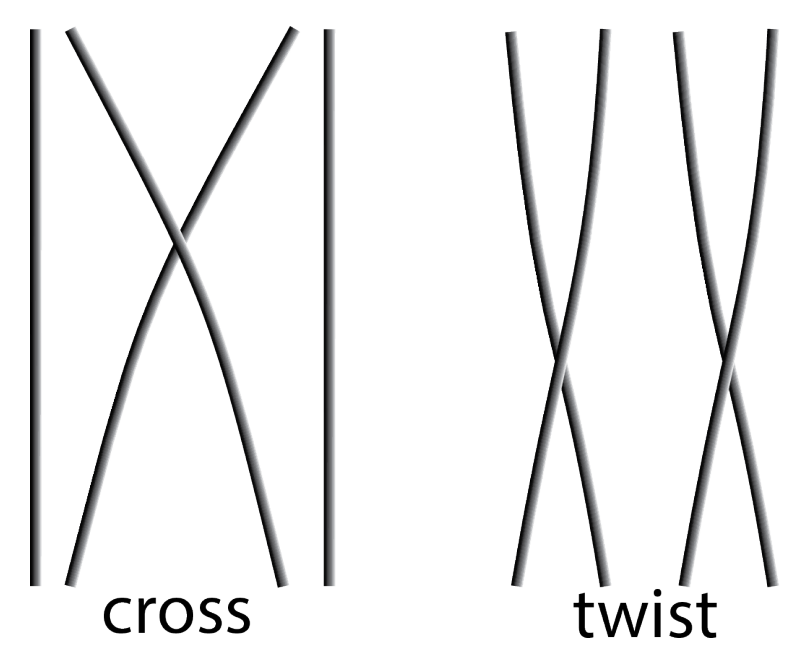

Figure S1: Cross (c) and twist (t) are the main movements of traditional bobbin lace stitches.

All the prototyped textile samples are based on universal grid with horizontal, vertical, and 45° lines, presented in the book Gründe mit System (*36*). The intervals used between the vertical and horizontal lines are 4 mm. Many grid variations fit to this universal grid and only following rules need to be followed while working with the roller lace pillow. The working direction is always downward or directly sideways, so that there is no possibility to return to area that has already been completed. S2

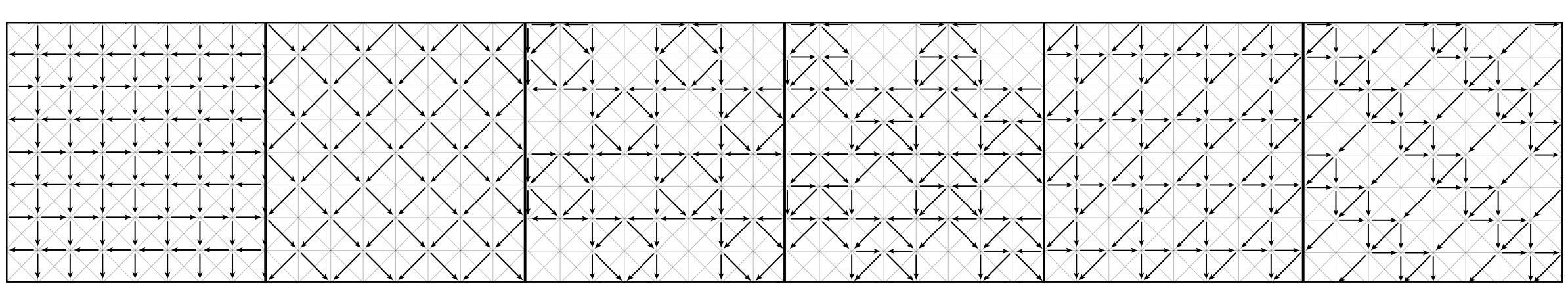

Figure S2: Grids used in this work. In this order in the figure: square, diamond, rose and rose variation, tilted star and tilted star variation (*28*). The arrow present the working direction and at the same time the path of a yarn pair.

The grid is a base for the bobbin lace pattern, but the same grid can be executed with many different

stitch variations. The simplest way is to repeat the same stitch combination at every interaction point of two pairs. This gives an interesting library of different possible patterns. Note that crossing and twisting must be alternated, or the result will be just pairs of threads twisted together. S3 The pattern can also be a mix of different stitch combinations. The structure of the pattern shows significant changes, even if only a small subsection of the stitches is varied. This is easy to see in the example, where the middle parts of rose ground patterns are always similar but the stitches between them varies. S4

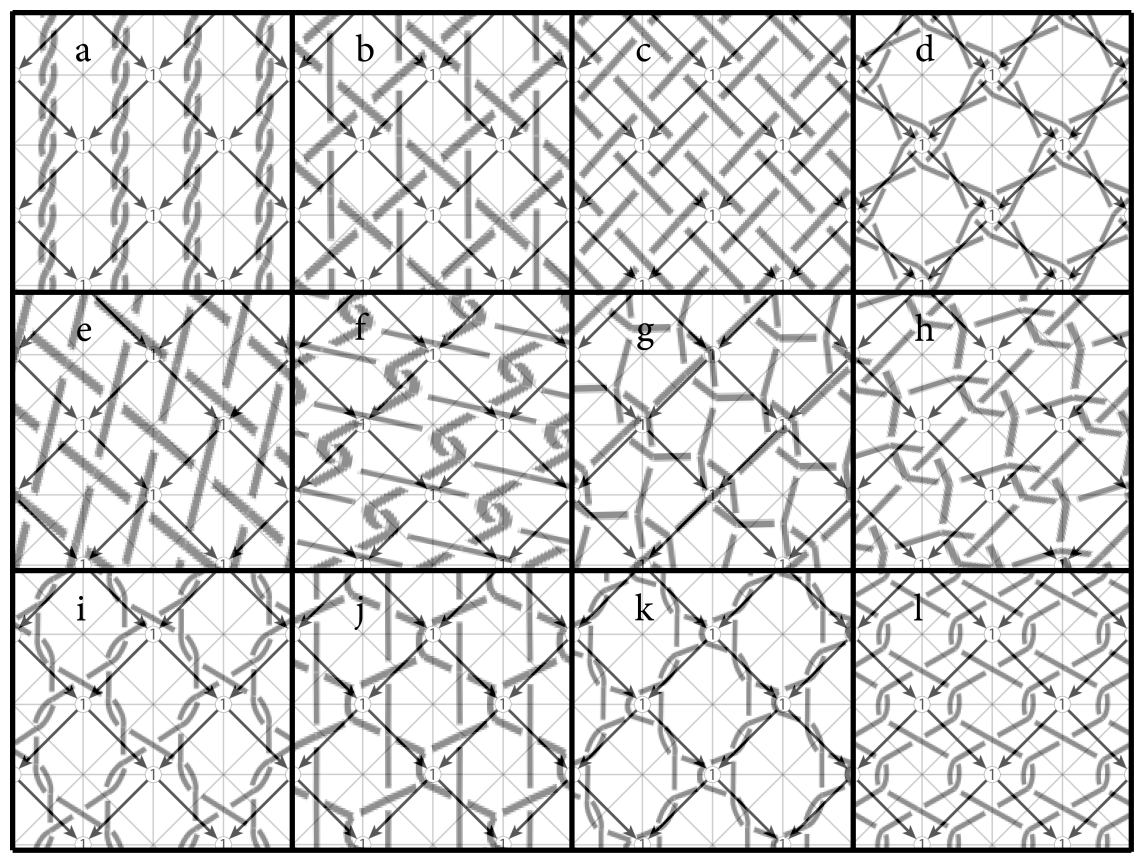

Figure S3: Diamond1: a t, b tc, c ctc, d ctct, e lc, f llc, g lclc, h lcrc, i ttc, j cct, k ttcc and l cttc. Shortenings: c = cross, t = twist, p = pin, l = twist only the left pair and r = twist only the right pair. Pattern visualizations were generated using the online tool https://d-bl.github.io/GroundForge.

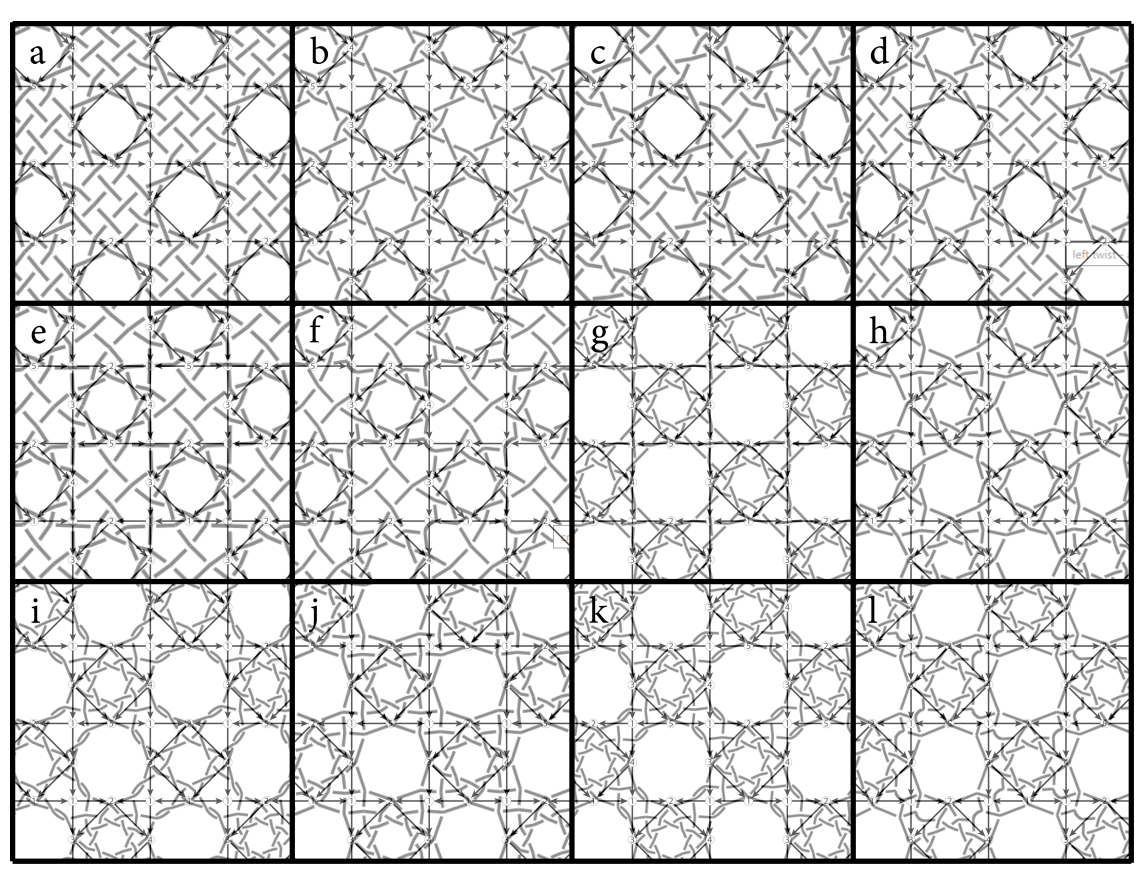

Figure S4: All of these roseground (rose5) presented follow the same pattern in intersection points 2-4: 2) ctpct, 3) ctpcr, 4) ctpcl, 5) ctpc. Point 1 vary: a) -, b) t, c) l, d) l/r, e) c, f) cc, g) tct, h) ct/tc, i) ctt/ttc, j) ctc, k) cttc, l) cctcc. If two different stitches are given they mirror each others creating symmetrical pattern. This is also an example of different possibilities in presenting the patterns. Pattern b is at the same time one version of rose5 and simply diamond1 with ctpct only. Shortenings: c = cross, t = twist, p = pin, l = twist only the left pair and r = twist only the right pair. Pattern visualizations were generated using the online tool https://d-bl.github.io/GroundForge.

The thread path is the route of the thread in the textile structure. The diversity of possible paths is an interesting specialty of the bobbin lace technique. The threads can have different kind of path within a single

repeating motif, but paths can still have the same shape and direction. In this case they are called the same path in this work. The number of different paths varies between 1-8 in this case, but theoretically in bobbin lace patterns it is unlimited. All these patterns also have a repeating unit that covers all the thread path information of the structure and repeats multiple times. S5

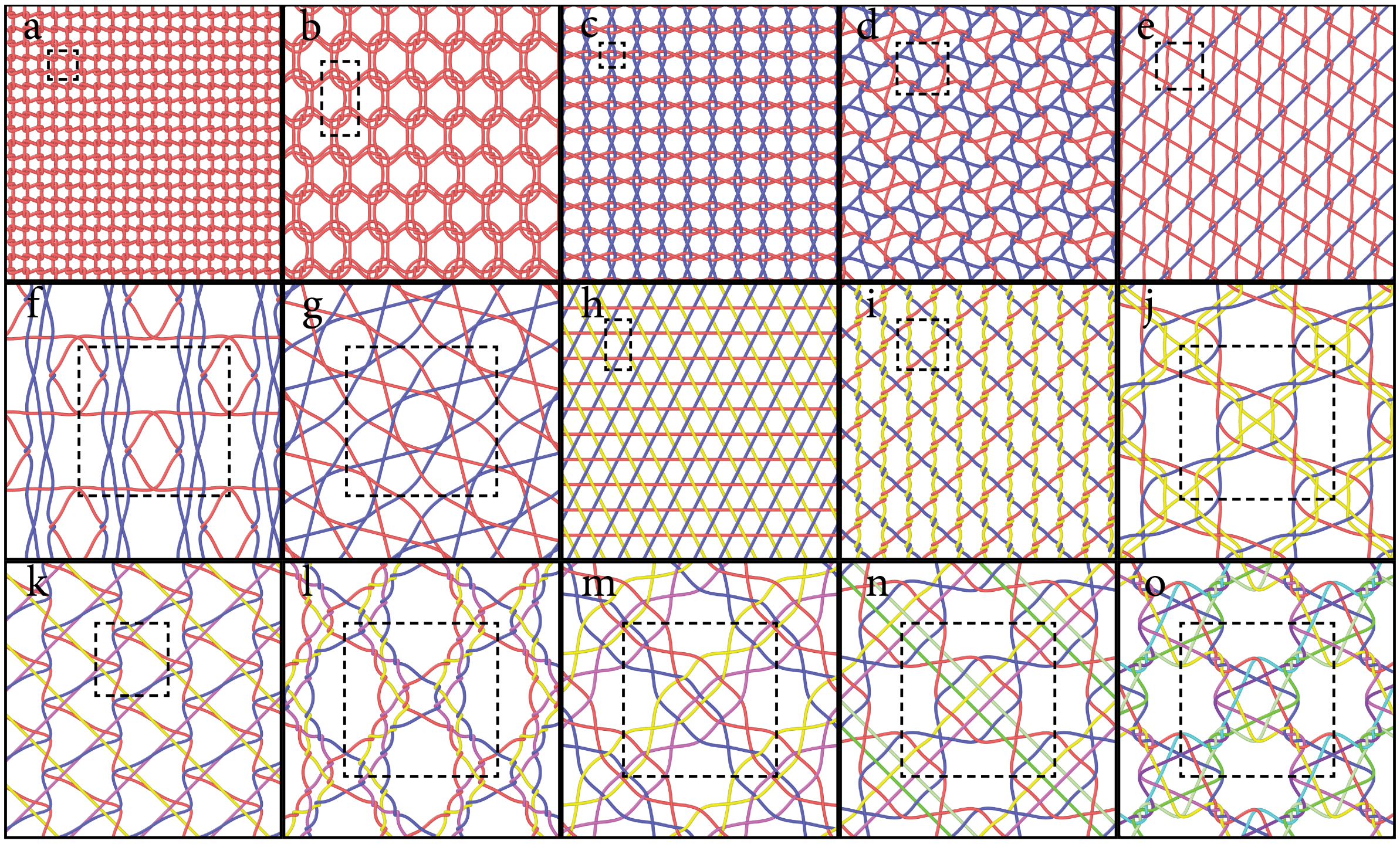

Figure S5: Some examples of different number of yarn paths in bobbin lace patterns: **a-b** 1 path, **c-g** 2 paths, **h-j** 3 paths, **k-m** 4 paths, **n** 6 paths and **o** 8 paths. Repeating units marked with dotted line.

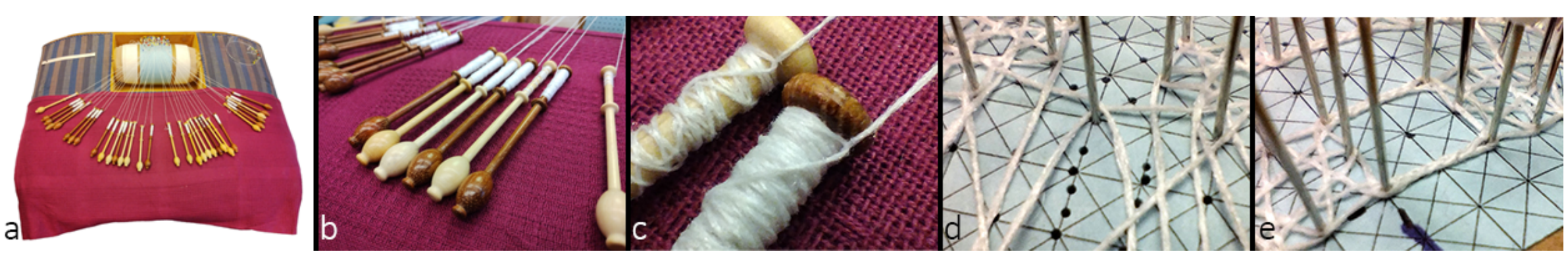

Figure S6: Tools used for making the textile samples: **a** the roller pillow, **b** bobbins, **c** the hitch to secure the yarn, **d** pin as an attaching point, **e** the braid used on edges and between samples.

The lace simulator

JSON structure Lace grounds (patterns) were previously given a rigorous mathematical definition by Irvine and Ruskey. (*31*) Their description approached the ground by setting a braid word to every vertex of a base grid, a combination that guarantees thread conservation and supports an exhaustive enumeration of workable patterns.

While this model is elegant and powerful, its formalism can feel hard to get introduced to designers look for more practice-based approaches, and hard to set specific thread configurations, with more or less loose

sections. We therefore introduce a lighter-weight, JSON-based blueprint that relies on the navigation of yarns between intersections (nodes). A pattern is saved in a single `.json` file organized in four top-level sections:

- `nodes` – base points that define crossings;
- `paths` – routes or lines connecting `nodes`;
- `unit_yarns` – the instructions for individual yarns that follow `paths`;
- `roi_bounds` – the overall boundaries of the design.

When building a lace pattern, the first step is to identify the crossings in the yarn pattern and then define how the yarns repeat along their path over this pattern. Consider the following lace, Fig. S7, for building the JSON structure:

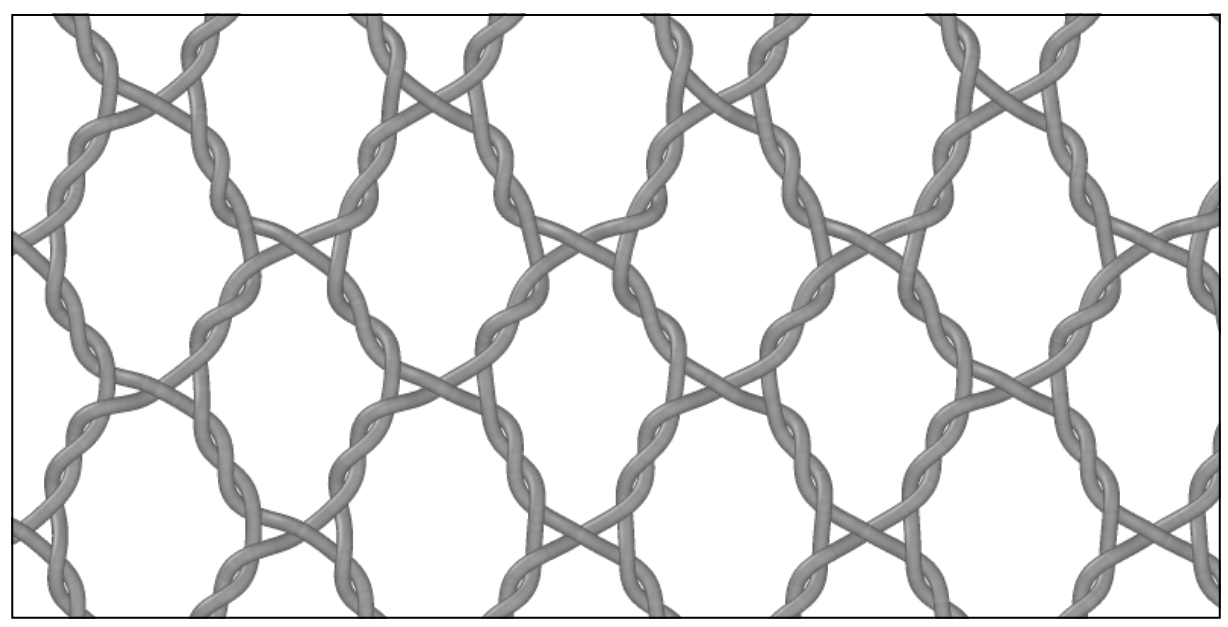

Figure S7: **Example of a lace pattern Diamond-2.**

Visually, there are multiple repeating structures in the fabric, namely the crossings and winding of yarn sequences. The following explanation outlines one possible way to establish those repetitions, as permitted by the mesh generation algorithm in the next subsection.

The first step is to define nodes, which represent interactions between yarns. Nodes are points in a two-dimensional space, characterized by x and y coordinates, similar to points on a graph. An important limitation arises when two yarns not only cross but also wind around each other one or more times, causing the paths defined by connected nodes to overlap. For this reason, we introduce a third property for nodes: the twist, τ.

The twist definition allows us to define two types of nodes, depicted in Fig. S8:

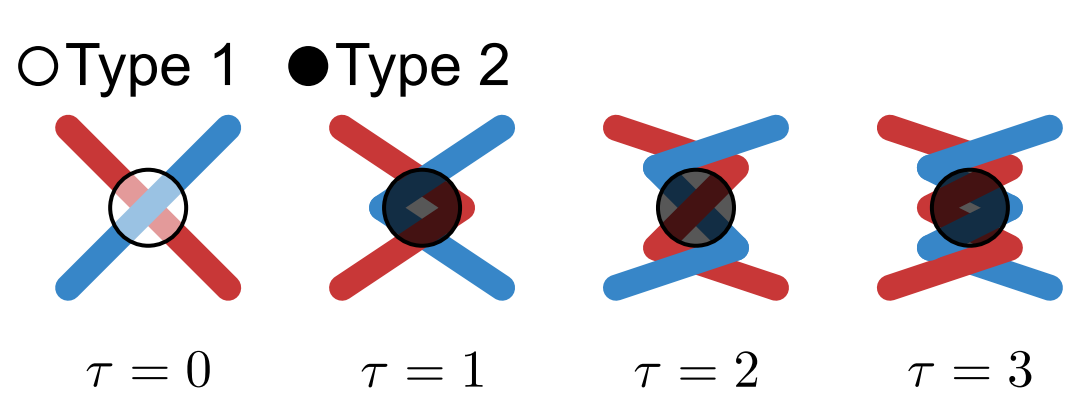

Figure S8: **Entanglement of nodes.** In **Type 1** nodes yarns simply cross, while in **Type 2** yarns, additionally, twist around each other.

- **Type 1** corresponds to $\tau = 0$, where two yarns simply cross without additional entanglement.

- **Type 2** corresponds to $\tau > 0$, where two yarns not only cross but also twist around each other.

The two different types of nodes can be sketched in the mesh using white and black filled circles, Fig. S9. Again, additional nodes can be considered to generate the mesh, but for this pattern, three nodes are enough.

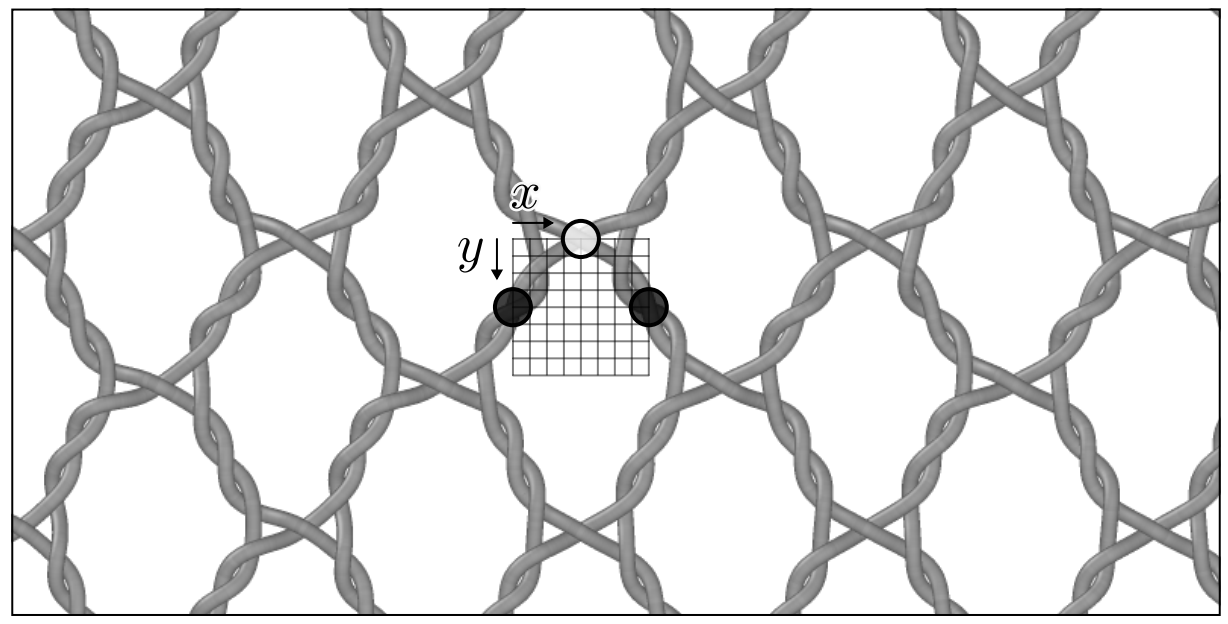

Figure S9: **Node drawing in lace example.**

The type 2 nodes have yarns crossing each other three times, so $\tau = 2$. We can then save all the parameters in the nodes section of the JSON file. In this work, patterns are built typically left to right and top down axis. Furthermore, additional labels are used for referencing in the paths section.

```
"nodes": {
  "0": { "x": 4, "y": 0, "twist": 0 },
  "1": { "x": 0, "y": 4, "twist": 2 },
  "2": { "x": 8, "y": 4, "twist": 2 }
},
```

After defining the nodes, paths describe how yarns connect them, forming the fundamental structure of the fabric. Paths define the basic unit of the yarns. In the simplest case, a single path represents one yarn. However, in more complex configurations, the same path can describe multiple yarns, depending on how they interact and repeat within the structure. To illustrate this concept further, we will use the example case to demonstrate how paths can describe both the simple and more complex designs. Each path consists in an array of node IDs, which outlines the order of connections. Paths also include a dictionary of shifts, which define additional translations. For example, consider a path that goes from point 0 to point 1 and then returns to point 0. Although, both occurrences are labeled with the same node ID, the second point 0 is in a different point in space. In this case, the shift $\Delta_{1,0}$ is applied to adjust the position of the second node 0, 0^* so that the path correctly, and typically $\Delta_{1,0} = p_0^* - p_0$, where $p_i = (x_i, y_i)$.

One way to check all paths starts by selecting the repeating unit and outlining the nodes to be connected. Sketching lines with different colors helps to find the repetitive units. For the case example, the repetitive design can be highlighted as shown in Fig. S10, where lines connect:

- red path: $0 \rightarrow 1 \rightarrow 0$;
- blue path: $0 \rightarrow 2 \rightarrow 0$;
- yellow path: $1 \rightarrow 2 \rightarrow 1$;

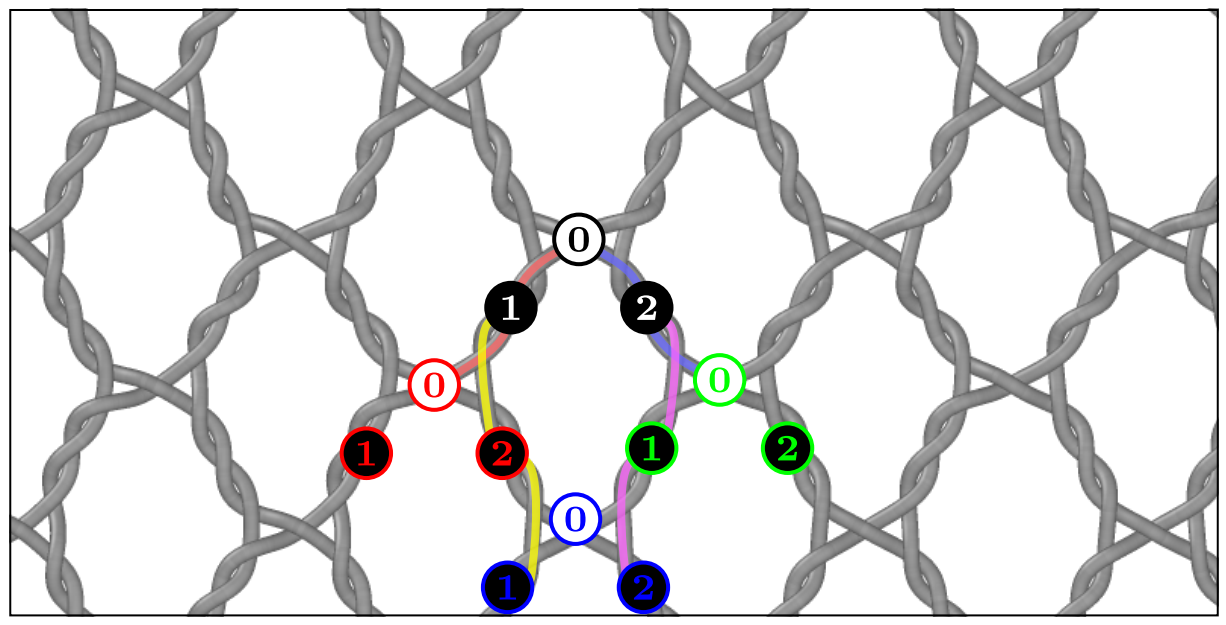

Figure S10: **Path generation in the lace example.**

- pink path: 2 → 1 → 2:

Therefore, we can write the paths section of the JSON file as:

```
"paths": [
    { "path": [ 0, "1l", 0 ], ← red path
      "shifts": [ { "from": 1, "to": 0, "dx": -8.0, "dy": 8.0 } ]
    },
    { "path": [ 0, "2r", 0 ], ← blue path
      "shifts": [ { "from": 2, "to": 0, "dx": 8.0, "dy": 8.0 } ]
    },
    { "path": [ 1, "2l", "1r" ], ← yellow path
      "shifts": [ { "from": 1, "to": 2, "dx": -8.0, "dy": 8.0 },
                  { "from": 2, "to": 1, "dx":  8.0, "dy": 8.0 } ]
    }
    { "path": [ 2, "1r", "2l" ], ← pink path
      "shifts": [ { "from": 2, "to": 1, "dx":  8.0, "dy": 8.0 },
                  { "from": 1, "to": 2, "dx": -8.0, "dy": 8.0 } ]
    }
  ],
```

Here, the colored text after the path list is not part of the JSON file and is used to highlight the different paths. Additionally, each of the type 2 nodes, in this case with IDs 1 and 2, includes an extra string *l* or *r*. This label indicates the side from which the yarn starts the crossing. For instance, in the red path, when traveling from node 0 to node 1, the yarn first crosses with the other yarn from the left side (y-axis uses top-bottom orientation).

In a more simple approach, yellow and pink paths can be used for describing distinct yarns. However, since both paths describe the same pattern, the latter can be discarded.

Unit yarns define how yarns repeat along the pattern. By the end of this section, all parameters should be sufficient to fully replicate the lace pattern. These parameters contain instructions for: which path to follow, `path_id`; where to start on that path, `starting_node` and `start_path_index`; how the yarn lies in the starting point, `z_sign` and `z_height`; and how the yarns extend and repeat throughout the pattern.

From the previous section, the first and second paths (path 0 and path 1) each define a single yarn (Yarn 1 and Yarn 2), while the third path (path 2) describes two yarns (Yarn 3 and Yarn 4). The description of Yarn 1 and Yarn 2 should be straightforward, since both start in node 0 and their positions in the path list use the

first value. Yarn 3 and Yarn 4 start in node 1 and node 2, respectively. The position in the path is different for each of the yarns, while Yarn 3 starts in the first position of the path, Yarn 4 is displaced by one position.

In lace fabrics, each time a yarn crosses another, its position relative to the crossing yarn reverses in the next interaction. This means that if a yarn goes over another at one crossing, it will go under at the next, and vice versa. This alternating behavior is a fundamental characteristic of lace patterns and ensures that the structures are fully interlaced. Therefore, the `z_sign` parameter, which defines the crossing in the starting node, defines not only the crossing in the initial position but in whole pattern. The parameter `z_height` controls how much the z coordinate varies between nodes. A small value of `z_height` may cause collisions of the yarns at crossings, increasing the chance of causing issues during the stabilization step of the simulations. A large value of `z_height` might make the structure excessively wavy and it become too loose. In most cases, a value of 0.5 was used.

The yarns section of the JSON file can be written as:

```
"unit_yarns": {
    "0": { "path_id": 0, "starting_node": 0, "start_path_index": 0,
           "z_sign": -1, "z_height": 0.5, "name": 1,
           "repetitions": [ { "count": 25, "dx": -8.0, "dy": 8.0 },
                            { "count": 50, "dx": 16.0, "dy": 0.0 } ] },
    "1": { "path_id": 1, "starting_node": 0, "start_path_index": 0,
           "z_sign": 1, "z_height": 0.5, "name": 2,
           "repetitions": [ { "count": 25, "dx": 8.0, "dy": 8.0 },
                            { "count": 50, "dx": 16.0, "dy": 0.0 } ] },
    "2": { "path_id": 2, "starting_node": 1, "start_path_index": 0,
           "z_sign": -1, "z_height": 0.5, "name": 3,
           "repetitions": [ { "count": 20, "dx": 0.0, "dy": 16.0 },
                            { "count": 50, "dx": 16.0, "dy": 0.0 } ] },
    "3": { "path_id": 2, "starting_node": 2, "start_path_index": 1,
           "z_sign": 1, "z_height": 0.5, "name": 4,
           "repetitions": [ { "count": 20, "dx": 0.0, "dy": 16.0 },
                            { "count": 50, "dx": 16.0, "dy": 0.0 } ] }
  },
```

The repetitions section defines two spatial transformations applied to the yarns. The first transformation extends the yarn by a factor of `count`, following the vector $\mathbf{v}_{\text{rep1}} = (dx_1, dy_1)$. The second transformation replicates the extended yarn by another factor of `count`, following the vector $\mathbf{v}_{\text{rep2}} = (dx_2, dy_2)$. It is important to note that $\mathbf{v}_{\text{rep1}}$ and $\mathbf{v}_{\text{rep2}}$ do not need to be orthonormal, allowing flexible repetition patterns that accommodate different lace structures.

Fig. S11 illustrates how the different types of repetitions work in the case of Yarn 1.

As stated in the previous subsection, the repetition vectors can take any direction. However, since our simulations use a box-like region, certain patterns – such as the example presented – may not produce a perfect regular repetition. This can result in an irregular arrangement of yarns, as illustrated in Figure S12:

The *roi_bound* set the "frame" or boundary for the entire lace design. The vectors need to be set, namely the `count` values and directions of the vectors, so they produce repetitions that fit within a designated area.

```
"roi_bounds": {
```

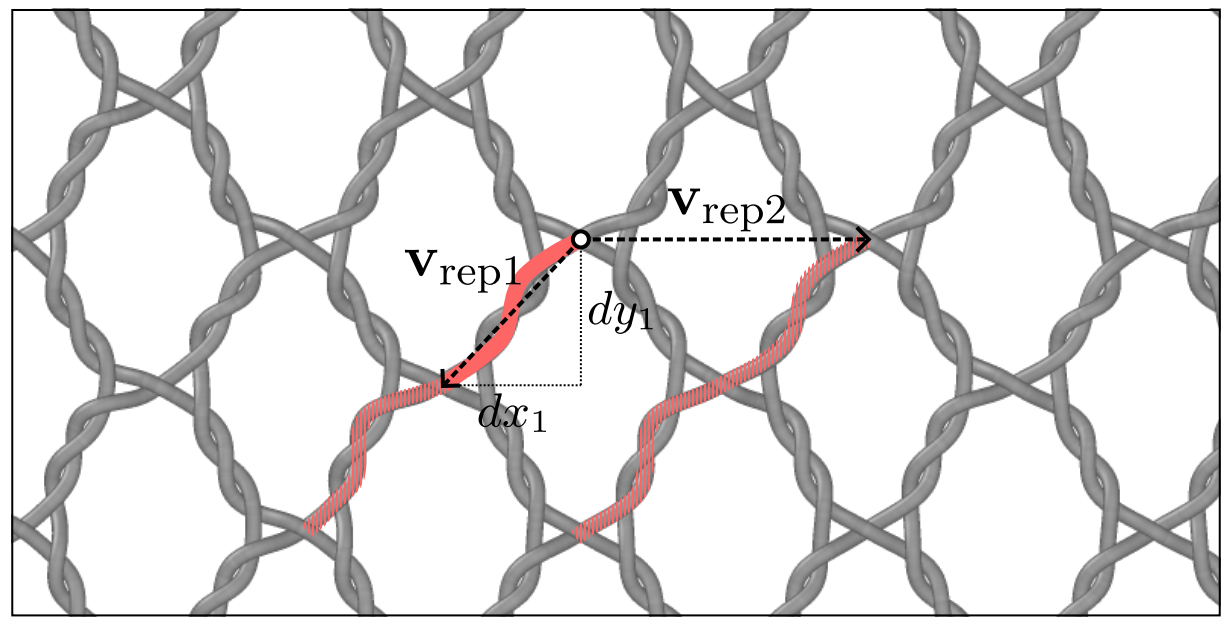

Figure S11: **Yarn generation in the lace example.**

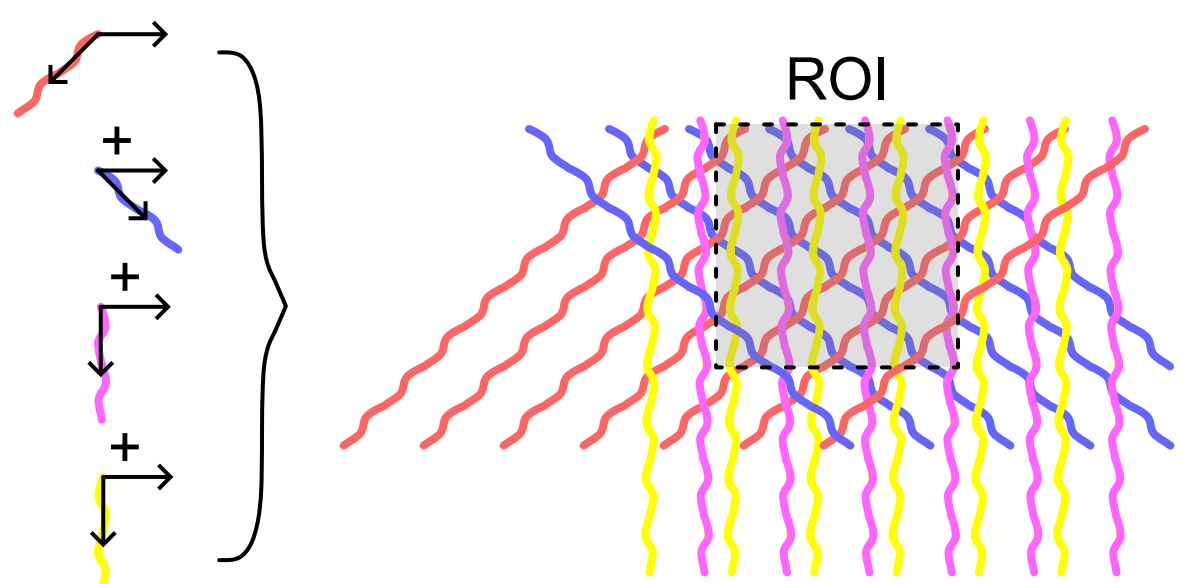

Figure S12: **Cropping a region of the lace example.**

```
    "x_min": 126.0,
    "x_max": 254.0,
    "y_min": -12.0,
    "y_max": 140.0,
    "z_min": -4.0,
    "z_max": 4.0
}
```

Mesh Generation A Python script was developed to generate the mesh from an input JSON file, containing the recipe to generate the lace structures and input parameters for the output file to be used as part of the LAMMPS simulations.

- JSON structure:
  - `nodes`, contains x and y coordinates of crossings, and crossing twists;
  - `paths`, repeat unit path;
  - `unit_yarns`, repeat unit yarns, contains information about which path is used as unit, the starting node position, the starting path position, the crossing z-position at the starting node, the ID, and the repetitions;
  - `roi_bounds` simulation box coordinates;
- LAMMPS parameters:
  - `dist_particles`, this value sets the distance between particles;

- `units`, multiplier for scale all coordinates;
- `mass`, mass for all particles;
- `threshold`, filter for removing yarns with smaller arc lengths;
- `ks1`, `ks2`, and `kb`, stretching constants for passive and active yarns, and bending constants for all yarns;
- `twist_distance`, value to control the radius of crossings that twist;
- `invert`, parameter to mirror y-axis.

The `GenerateMesh` function starts by reading all parameters taking care of the y-axis mirroring, if needed.

```
FUNCTION GenerateMesh():
    PARSE arguments INTO args
    SET data = load_data(args.json_file)
    SET nodes = data["nodes"]
    SET paths = data["paths"]
    SET unit_yarns, and unit_rep FROM data["unit_yarns"]
    SET roi_bounds = data["roi_bounds"]
    SET dist_particles = args.dist_particles
    SET units = args.units
    SET mass = args.mass
    SET threshold = args.threshold
    SET ks1 = args.ks1
    SET ks2 = args.ks2
    SET kb = args.kb
    SET twist_distance = args.twist_distance
    SET invert = args.invert_y
    IF invert < 0 THEN:
        FOR EACH node IN nodes DO:
            SET node['y'] = -node['y']
        FOR EACH path IN paths DO:
            FOR EACH item IN path["path"] DO:
                IF item is a special node (string containing 'l' or 'r'):
                    SWAP 'l' and 'r' in item
            FOR EACH key IN path["shifts"] DO:
                SET path["shifts"][key]['y'] = -path["shifts"][key]['y']
        FOR EACH key IN unit_rep DO:
            INVERT appropriate elements (e.g., 3rd and 6th)
        SWAP roi_bounds['y_min'] and roi_bounds['y_max'] (with sign inversion)
```

The list of `nodes` contain a set of n points,

$$P = \{p_0, p_1, \dots, p_n\}, \tag{S1}$$

and each point p_i is represented by 2D coordinates in space, $p_i = (x_i, y_i)$. The list of `paths` contain how the unit yarns travel along the `nodes`:

$$Q_j = \{q_{j,0}, q_{j,1}, \dots, q_{j,m_j}\}, \tag{S2}$$

where m_j is the number of indices in path j (each $q_{j,i}$ is an index such $p_{q_{j,i}}$ is a node in P. Then, for each path j, the translation vectors can be defined as:

$$\mathbf{s}_{j,i} = (p_{q_{j,i+1}} - p_{q_{j,i}}) + \Delta_{j,i}. \tag{S3}$$

Each translation $s_{j,i}$ is the difference between coordinates of nodes at consecutives positions in the path list. $\Delta_{j,i} = (0,0)$ unless it is defined in `paths.shifts`. The dictionary `paths.shifts` contains four values, `from`, `to`, `dx`, and `dy`, so if (`from`, `to`)= (j,i) then $\Delta_{j,i}$ =(`dx`, `dy`). Then, the list of translation vectors for path j is:

$$S_j = \{\mathbf{s}_{j,0}, \mathbf{s}_{j,1}, \ldots, \mathbf{s}_{k,m_j-1}\}. \tag{S4}$$

Additional considerations need to be taken for different types of nodes. There are two types of nodes: type 1, where the two yarns simply cross, and type 2, where, in addition to crossing, the yarns can also twist around each other. For type 2 nodes, the path information, besides the node number, includes an extra string, either l or r. Therefore, the `calc_translations` function retrieves a list with four values for each translation. The first two values are the x and y components of S_j elements, $(s^x, s^y) = \mathbf{s}$, while the last two values are specific to type 2 nodes. The third value contains the amount of twisting, multiplied by -1 or 1, depending on whether the string in the path information is l or r, respectively. The fourth value is an angle that orients the yarns twisting around each other, thus requiring all paths to be defined first. After calculating all unit paths, the `node_angle` function will check for nodes of type 2 and calculate the third and fourth components accordingly, and define all four components of `calc_translations`, $\{s^x, s^y, \tau, \theta\}$.

For clarity, consider the example shown in Fig. S13: two unit paths – one red and one blue – intersect at point α. In this case, the function `calc_translations` computes S_j for all paths. Both the red and blue path lists include the point α at some position, denoted as $Q_{\text{red}} = \{\ldots, q_{\text{red},\beta}, \ldots\}$ and $Q_{\text{blue}} = \{\ldots, q_{\text{blue},\gamma}, \ldots\}$, respectively. Each of these values contains the numeric ID α, with the red value carrying an l label and the blue an r label. Consequently, for the u and v values, `calc_translations` assigns -1 and 1, respectively, to the third component, given that the twist value is 1. The fourth component, representing the angle of orientation at the crossing for u and v, is calculated by the `node_angle` function. This function begins by retrieving all nodes with l and r labels from all paths and then verifies that there is exactly one l and one r with the same node ID. This check is necessary for computing all the required quantities. Afterwards, for each pair, the angles formed by the two paths entering node α, θ_{α_1}, and leaving node α, θ_{α_2}, are calculated by:

$$\theta_{\alpha_1} = \text{atan2}\left(s^y_{\text{red},u-1} + s^y_{\text{blue},v-1}, s^x_{\text{red},u-1} + s^x_{\text{blue},v-1}\right) \tag{S5}$$

$$\theta_{\alpha_2} = \text{atan2}\left(s^y_{\text{red},u} + s^y_{\text{blue},v}, s^x_{\text{red},u} + s^x_{\text{blue},v}\right), \tag{S6}$$

where atan2 is a function that returns the angle in the plane, in radians, makes around the origin $(0,0)$. Finally, the average value of these two angles is saved as the fourth component:

$$\theta_\alpha = \frac{\theta_{\alpha_1} + \theta_{\alpha_2}}{2} \tag{S7}$$

The next step of the script iterates over each type of yarn and generates all yarns. The function `generate_yarns` initializes a yarn at a user-defined starting point and generates it point by point using a repeat unit path. At this stage, it introduces the z component, whose value oscillates between $\pm z_0$. This

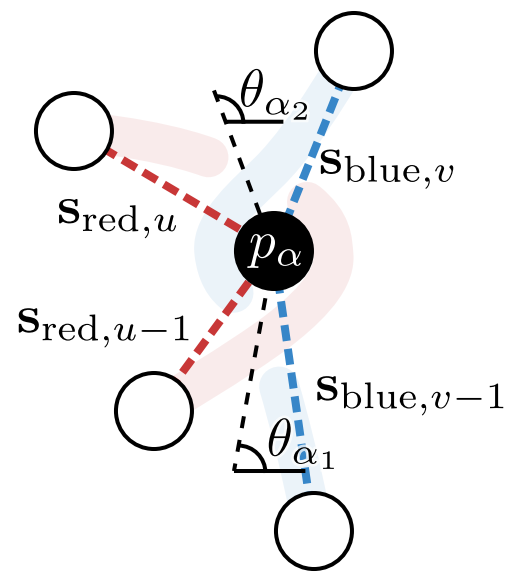

Figure S13: **Diagram illustrating different types of nodes:** type 1 (white filled circles) – yarns simply cross; type 2 (black filled circle) yarns cross and twist (twist of p_α is 1 in this example). The orientation of the crossing is given by the average of the angles formed by the paths entering and leaving p_α.

simplification arises from the fact that all yarns in laces alternate between over and under at crossings. Also, at this point, type 2 nodes need to be further expanded. Therefore, when a node has a non-zero twist, a set of auxiliary points are first built using the third and fourth components previously saved on `path_translations`, $\{\_,\_,\tau,\theta\}$. So, for $i = 0,1,2,\ldots,2|\tau|$ we begin a sequence with $(x_0, y_0) = (-\tau, -\tau)$. The follow up points, $i > 0$ are built so:

$$(x_{i+1}, y_{i+1}) = \begin{cases} (x_i, y_i + 2), & \text{if } \left[\frac{1}{2}(i - 1 - \text{sgn}(\tau)) \mod 4)\right] < 1, \\ (x_i + 2, y_i), & \text{otherwise.} \end{cases} \tag{S8}$$

The conditional formula in Eq. S8 creates a staircase pattern where it starts by moving up (or right) by one unit, and then keeps alternating between right and top (top and right) by two units. The example in Fig. S14 shows a generation of auxiliary points for $\tau = -3$ and $\tau = 3$.

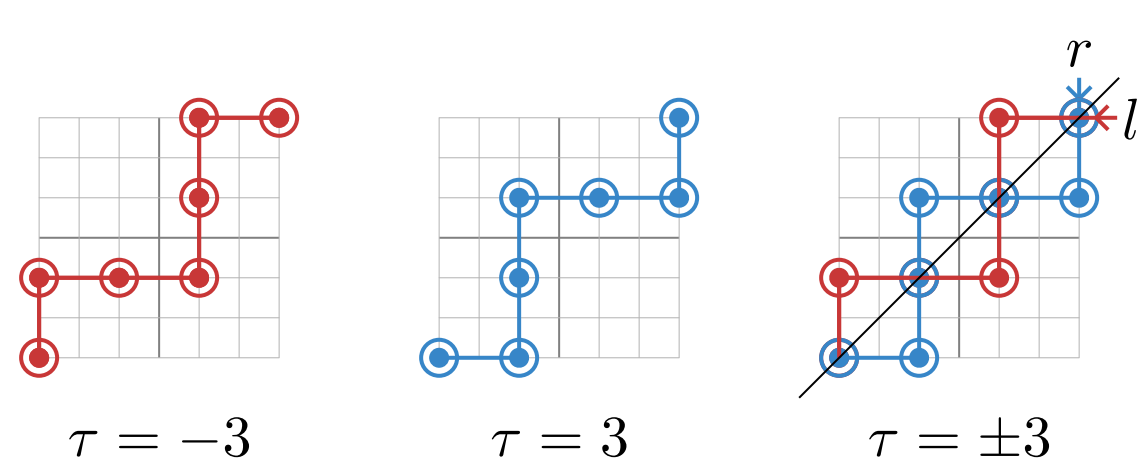

Figure S14: **Diagram illustrating the generation of auxiliary points for replacing type 2 nodes.** The number of points is equal to $2\tau + 1$, which generates path patterns that cross and twist by an amount of τ.

The generation of auxiliary points is not aligned with the primary alignment of the yarns twisting and, therefore, each point needs to be further rotated by an angle $\theta^* = \theta - \pi/4$ to match the orientation of the incoming and outgoing paths:

$$(x^*, y^*) = (x\cos\theta^* - y\sin\theta^*, x\sin\theta^* - y\cos\theta^*) \tag{S9}$$

After obtaining the set of auxiliary points, these points are used to generate the additional points to represent the twisted yarns section:

$$p_{x,i} = p_x + \rho x_i^*, \tag{S10}$$

$$p_{y,i} = p_y + \rho y_i^*, \tag{S11}$$

$$p_{x,i} = c_\pm \cdot z_0 \cos\left(i\frac{\pi}{2}\right), \tag{S12}$$

where the base position of the node is (p_x, p_y), ρ is a scaling factor that controls the amplitude of the auxiliary points, $c_\pm$ is a factor indicating the yarn's position at a crossing – over $(+1)$ or under (-1). The cosine term, $\cos(i \cdot \pi/2)$, cycles through the values of $1, 0, -1, 0, \ldots$ with k, creating a coiling effect that emulates the twisting of the two yarns.

At this stage, the yarns are generated at the scale of unit paths. The next step to replicate these yarn segments according to the repetitions parameters. The function `extend_points` performs this replication by extending the generated yarns points n times, applying a uniform shift in the x and y directions for each instance. For each replication index k, each point p_i is shifted by:

$$p_i^{(k)} = \left(x_i + kv_x, y_i + kv_y, z_i\right), \tag{S13}$$

where v_x and v_y are the shifts in the x and y directions.

Now that all yarns were expanded further the unit path, the function `smooth_yarn` has two purposes: first, as its name suggests, it smooths the path to achieve a shape that more closely resembles a real yarn; second, it subsamples the smoothed curve, providing an evenly spaced representation of the original path.

The fitting of discrete points to a continuous function, on par with the nodes' position, significantly impacts mesh generation. In this work we fitted a parametric spline using SciPy's `splprep` function, with a smoothing factor of 0.1. The spline is then evaluated at a high resolution:

$$C = \{p_i\}_{i=0}^{N-1}, \tag{S14}$$

where $N = 1 \times 10^6$. From this set, a subsequence,

$$C^* = \{q_j\}_{j=0}^{M-1}, \tag{S15}$$

is extracted such that the spacing between consecutive points in C^* is approximately the minimum distance `dist_particles/units`, denoted as L. This value should be close to the intended equilibrium bond distance in the LAMMPS simulation. The points in C^* are determined recursively: starting $q_0 = p_0$, we initialize $C^* = \{p_0\}$. Then, for $i > 0$, the distance $\|p_i - p_0\|$ is iteratively checked, and when $\|p_i - p_0\| \geq L$ is satisfied, p_i is added to $C^* = \{\ldots, p_i\}$ and p_0 is updated to q_i. This process continues until the last point in C is reached.

After smoothing, all yarns are replicated using the function `replicate_points`, similar to how they were previously extended. The key difference between `extend_points` and `replicate_points` is that the former lengthens the yarn by concatenating segments, while the latter generates multiple independent yarns from the same base curve. However, the underlying mathematical shift remains the same in both functions.

The vectors used to extend and replicate yarns do not need to be orthonormal. However, in this work, the output mesh typically is contained in a box-like region. To ensure that only relevant points are retained, the function `filter_points` checks whether each point lies within the specified region of interest (`roi_bounds`).

```
... continuation of FUNCTION GenerateMesh
```

```
    INITIALIZE path_translations AS empty list
    FOR EACH path IN paths DO:
        APPEND calc_translations(nodes, path) TO path_translations
    SET path_translations = node_angle(path_translations, paths)
    INITIALIZE yarns = empty list, mol_max = 0
    FOR index k FROM 0 TO (number of unit_yarns - 1) DO:
        SET current_unit_yarn = unit_yarns[k]
        CALL generate_yarns(nodes, path_translations[current_unit_yarn[0]],
                            current_unit_yarn, twist_distance)
            = RETURNS (yarn, translations)
        SET (n1, vx1, vy1, n2, vx2, vy2, mol) = unit_rep[k]
        IF mol > mol_max THEN:
            SET mol_max = mol
        SET yarn_ext = extend_points(yarn, n1, vx1, vy1)
        SET yarn_sm = smooth_yarn(yarn_ext, arc_length = dist_particles/units)
        FOR l FROM 0 TO n2 DO:
            APPEND replicate_points(yarn_sm, mol, l * vx2, l * vy2) TO yarns
    FOR EACH yarn IN yarns DO:
        SET yarn = filter_points(yarn, roi_bounds)
```

Before writing the mesh structure in a format compatible with LAMMPS, we first label yarns independently of their type. The data is organized so that if the Euclidean distance between two consecutive points exceeds twice the expected spacing, the points are considered to belong to different yarns. Thus, all particles that are closely spaced get the same yarn ID, while a large gap causes a new ID to be assigned.

Functions `calculate_bonds` and `calculate_angles` are called to handle required information for the LAMMPS input file formats. The function `calculate_bonds` connects points belonging to the same yarn. A parameter `threshold` can be used to define special functions to be applied in boundary particles. However, in this work, boundary particles were set as immobile so this functionality was not used. Therefore, for the standard bonds each bond is stored as a tuple of the form:

$$(\texttt{type}, k-1, k)\,, \tag{S16}$$

where k is the particle ID and `type` is given by the yarn type. The function `calculate_angles` considers triplets of consecutive particles that belong to the same yarn. For the angles information, each angle is stored as a tuple:

$$(\texttt{angle\_type}, k-2, k-1, k)\,, \tag{S17}$$

and `angle_type` = `type` was also set by the yarn type.

Finally, the `write_lammps_data` function saves all parameters, specifying the simulation box, initial atom coordinates, bonds, angles, and force parameters.

```
... continuation of FUNCTION GenerateMesh
    INITIALIZE empty list yarn_id, c = 0
    FOR index k FROM 0 TO (length of yarns - 1) DO:
        IF k > 0 THEN:
            SET distance = Euclidean distance between yarns[k] and yarns[k-1]
        IF distance > 2 * dist_particles THEN:
```

```
            INCREMENT c by 1
        APPEND c TO yarn_id
    SET bonds = calculate_bonds(yarns_flat, yarn_id, mol_max)
    SET angles = calculate_angles(yarns_flat, yarn_id)
    CALL write_lammps_data(yarns_flat, yarn_id, bonds, angles, roi_bounds, dist_particles,
                           mass, units, ks1, ks2, kb, output filename)
```

LAMMPS simulations

In this work, two LAMMPS simulations were conducted for each experiment. The first simulation stabilizes the mesh, and in some cases, it also reduces the distance between particles by decreasing the equilibrium bond distances, tightening the fabric. This reduction was typically 4%. The second simulation applies a contraction of up to 25% to selected yarns, at steps of 2.5%, based on the (already reduced) equilibrium bond distances, and an expansion back to their original values. In this section, the LAMMPS code is explicitly shown instead of the pseudocode.

The first LAMMPS script starts by initializing the simulation using Lennard-Jones (dimensionless) units, a molecular atom style, non-periodic boundaries in all directions, and bond and angle styles.

```
units          lj
atom_style     molecular
boundary       m m m
bond_style     harmonic
angle_style    harmonic
```

Bonds and angles use harmonic potentials,

$$E_{\text{bond}} = k_{\text{bond}}(r - r_0)^2, \tag{S18}$$

$$E_{\text{angle}} = k_{\text{angle}}(\theta - \theta_0)^2, \tag{S19}$$

where k_{bond} and k_{angle} are the energy constants, so related with stretching and bending rigidities; r_0 is the equilibrium bond distances, in our case the distance between consecutive particles; and θ_0 is the equilibrium bending angle, the angle made by three consecutive particles. Simulations have typically $r_0 \approx 0.25$ and $\theta_0 = 0$.

The yarn data, generated in the previous subsection, is loaded by using the command

```
read_data    ${folderdata}
```

The `pair_style` computes pairwise interactions, which define the interaction between a particle and its close neighbors. This potential is responsive by the interaction between yarns and sections of the same yarn not in the same location.

```
pair_style    soft_exclude 1.0 16
pair_coeff    pair_coeff * * 200.0
```

We used the style `soft_exclude` which is a custom-made potential for our simulations. This potential is based in the style `soft` which computes pairwise interactions with:

$$E_{\text{soft}} = A\left[1 + \cos\left(\frac{\pi r}{r_c}\right)\right], \quad r < r_c \tag{S20}$$

where A is a constant that control the intensity of the repulsion, and r_c is a cutoff distance, so the potential does not apply when yarns are not in contact. This potential does not diverge as r approaches 0. We considered a yarn radius $r_{\text{yarn}} = 0.5$, a cutoff distance $r_c = 2r_{\text{yarn}} = 1.0$, and $A = 200$. Since $r_0 < r_c$, some neighbor particles might be identified for the pairwise interaction, which might overcome the effect of bond interactions. Therefore, we set another parameter that allows to switch off the intramolecular pairwise interactions of close neighbors. In our simulations, an `exclude` value of 16 was found to work for all simulations.

The same input script was used for all simulations, with eight flags for checking if types exist or not, `b1` to `b8`. This approach was chosen because the maximum number of yarn types in our experiments is in max eight or lower, but the strategy employed in this part can be further expanded.

```
if "${b1} == 1" then "bond_coeff 1 ${ks} ${bond1}"
...
if "${b8} == 1" then "bond_coeff 8 ${ks} ${bond8}"
```

Next, the script defines dump commands to output atom positions, forces, and potential energy per atom to a file while also logging simulation details to a separate log file.

```
variable    filedump string "${sim0_data}"
variable    logdump string "${sim0_log}"
compute     perAtomPE all pe/atom
dump        myDump all custom ${thermodump} ${filedump} id mol type x y z fx fy fz c_perAtomPE
log         ${logdump}
```

Then, several spatial regions are defined based on given coordinates and groups atoms in these regions, so it can later have their forces set to zero (freezing them), while all others are subjected to a viscous drag fix and NVE integration – time integration uses velocity-Verlet algorithm.

```
region    left_block block INF ${xmin} INF INF INF INF units box
region    right_block block ${xmax} INF INF INF INF INF units box
region    bottom_block block INF INF INF ${ymin} INF INF units box
region    top_block block INF INF ${ymax} INF INF INF units box

group     left_wall region left_block
group     right_wall region right_block
group     bottom_wall region bottom_block
group     top_wall region top_block

group     boundary union left_wall right_wall bottom_wall top_wall
fix       freezef boundary setforce 0.0 0.0 0.0
fix       dragU all viscous ${visc}
fix       nve_all all nve
```

An energy minimization is then performed to relax the initial configuration, and neighbor lists are set to update every 100 steps.

```
minimize       1.0e-6 1.0e-7 10000 100000
neigh_modify   every 100
```

The integrator parameters are specified next, setting the timestep and thermodynamic output frequency (with custom output including step, temperature, potential energy, kinetic energy, and total energy). Finally, the script calculates the total number of iterations based on the thermo frequency and number of frames to simulate, runs the simulation for that duration, and writes out a restart file so that the simulation can later be resumed for performing different contraction experiments.

```
timestep        ${tstep}
thermo          ${thermodump}
thermo_style    custom step temp pe ke etotal

variable        iter equal ${thermodump}*${nframes}
run             ${iter}
write_restart   ${restart}
```

The second script uses the stabilized structure, so it begins by reading the restart file. This operation sets up the simulation to continue the calculation from the last state of the previous script. All atom velocities are initialized to zero, and the neighbor list is set to update every 100 timesteps. The integrator settings, and other outputs are configured in a similar way as the previous script, using a custom format, a custom dump outputting IDs, molecule IDs, types, positions, forces, and potential energies per atom, and logging details.

```
read_restart    ${restart}
velocity        all set 0.0 0.0 0.0
neigh_modify    every 100
timestep        ${tstep}
thermo          ${thermodump}
thermo_style    custom step temp pe ke etotal

variable filedump string "${sim1_data}"
variable logdump string "${sim1_log}"
compute  perAtomPE all pe/atom
dump     myDump all custom ${thermodump} ${filedump} id mol type x y z fx fy fz c_perAtomPE
log      ${logdump}
```

The script continues by applying fixes to the system, namely setting the force in boundaries as zero, so fixing the atoms position, while all other particles are subjected to a viscous drag fix and NVE integration. The simulation runs for `thermodump` timesteps, which set the initial configuration of the lace.

```
fix   freezef boundary setforce 0.0 0.0 0.0
fix   dragU all viscous ${visc}
fix   nve_all all nve
run   ${thermodump}
```

Following the initial run, the stage cycles over a contraction loop, which is followed by the recovery loop. Each iteration of the contraction loop, ($1 \leq t \leq N$, if the flag is equal $b_k = 1$, then it changes the bond coefficient of atoms of type k by:

$$r_{0,k}(t) = \left(1 - \frac{t}{N}\delta\right) \cdot r_{0,k}(t=0), \tag{S21}$$

where N is the total number of steps to contract and δ is the amount of contraction. Simulations used $\delta = 0.25$, so when $t = N$, then $r_{0,k}(t) = 0.75 \cdot r_{0,k}(0)$.

```
label loop
variable i loop ${nframes}
    variable coeff1 equal ${bond1}*(1-(${i}/${nframes})*(${contract}))
     ...
    variable coeff8 equal ${bond8}*(1-(${i}/${nframes})*(${contract}))

     if "${b1} == 1" then "bond_coeff 1 ${ks} ${coeff1}"
     ...
     if "${b8} == 1" then "bond_coeff 8 ${ks} ${coeff8}"
    run ${thermodump}
next i
jump ${self} loop
```

In the subsequent loop, the bond equilibrium distances are gradually increased back to their original value, again updating bonds and running the simulation iteratively. For each iteration, ($1 \leq t \leq N$, if the flag is equal $b_k = 1$, then it changes the bond coefficient of atoms of type k by:

$$r_{0,k}(t) = \left(1 - \delta + \frac{t}{N}\delta\right) \cdot r_{0,k}(t = 0), \tag{S22}$$

so when $t = N$, then $r_{0,k}(t) = r_{0,k}(t = 0)$.

```
label loop2
variable j loop ${nframes}
    variable coeff1 equal ${bond1}*(1-${contract}+(${j}/${nframes})*(${contract}))
     ...
    variable coeff8 equal ${bond8}*(1-${contract}+(${j}/${nframes})*(${contract}))

     if "${b1} == 1" then "bond_coeff 1 ${ks} ${coeff1}"
     ...
     if "${b8} == 1" then "bond_coeff 8 ${ks} ${coeff8}"
    run ${thermodump}
next j
jump ${self} loop2
```

Video processing of physical laces

A script in Python opens each video `.mov` and process each frame. It stars by trimming the frame borders and rescales pixels to millimeters, then steps through a time clipping frame interval so only n-th frame is analyzed. Each selected frame is cropped to the region of interest, converted to a high contrast gray image, thresholded to isolate bright fibers cross-sections, and cleaned with erosion/dilation operations to remove specs, for instance tiny fragments of yarn that are typically observed after heating samples. The algorithm finds every closed contour (pores between fibers), converts their pixel areas to real areas, and logs those values with the timestamp. Furthermore, each pore is painted by contour area with a colorful overlay, saving as individual PNGs, which are later combined into an animated GIF and a panel thumbnail for quick visual inspection. When the video ends, the area-vs-time data is saved to a JSON file, and the routines frees the video handle.

```
OPEN video at video_path
```

```
READ frame_count = CAP_PROP_FRAME_COUNT
READ fps         = CAP_PROP_FPS

INITIALIZE areas_data
INITIALIZE frame_idx, frame_counter
INITIALIZE frames_for_gif

WHILE cap IS open:
    READ ret, frame
    IF ret IS FALSE: BREAK

    IF frame_idx < skip_ini:
        frame_idx++ ; CONTINUE

    IF frame_idx MOD frame_skip != 0:
        frame_idx++ ; CONTINUE

    IF frame_counter == 0:
        boundary_contour = CROP_FRAME(frame)
        [x, y, w, h]     = boundingRect(boundary_contour)
        px2mm            = 50  / MAX(w, h)
        boundary_contour = boundary_contour { [x,y] + [xalign,yalign]

    cropped = frame[y-1 : y+h+1 , x-1 : x+w+1]

    gray      = CONVERT_TO_GRAYSCALE(cropped)
    binary    = APPLY_THRESHOLD(gray)
    processed = REMOVE_NOISE(binary)

    IF frame_counter == 0:
        mask = BOUND_FIND(processed, boundary_contour)

    (areas, frame_png) = EXTRACT_AREAS(cropped, processed, mask,
                                       div = px2mm,
                                       time = frame_counter)
    APPEND frame_png   = frames_for_gif
    APPEND { time : frame_counter*frame_skip/fps , areas : areas }
           = areas_data

    INCREMENT frame_idx , frame_counter
END WHILE

WRITE GIF
WRITE 5-PANEL THUMBNAIL
DELETE all temporary frame_png files

RELEASE cap
WRITE JSON(areas_data) → output_json
```

LAMMPS dump files

The LAMMPS simulations generate "dump" files with the extension `.lammpstrj`, which contain the properties of the particles at different timesteps:

- `TIMESTEP`
- `NUMBER OF ATOMS`
- `BOX BOUNDS` – the coordinates of the simulation box boundaries
- `ATOMS`:
  - `id`, a label for each particle;
  - `mol`, a label usually used to link the particle to a molecule. In the case of this work, "molecules" are the different yarns.
  - `type`, a label to be used in the potential functions applied on the particle. In this case, the type label was used to control which yarns were actuated during the simulations;
  - `x`, `y`, and `z`, position of particles in space;
  - `fx`, `fy`, `fz`, components of the force applied in each particle;
  - `pe`, potential energy per particle.

A script in python processes the simulation data by (1) reading and parsing the dump file, then (2) calculates important properties to be followed like the magnitude of force and lengths, (3) identifies pores (or pores), which are the regions created by the gaps between yarns, and generates outputs in the form of animated GIF and JSON files with the extracted numerical data (yarns' lengths and pores' areas). Each task is broken down into the following subsections with pseudo-code.

The function `ReadDumpFile` reads all data so that it can be further processed. Furthermore, a user-defined region of interest (ROI: `xmin`, `xmax`, `ymin`, and `ymax`) was implemented, so the potential energy can focus on specific regions of the yarn mesh. This feature will be important, for instance, when using potentials in LAMMPS that introduce energy values that shadow a correct assessment of the energy landscape.

```
FUNCTION ReadDumpFile(file_path, xmin, xmax, ymin, ymax):
    OPEN file at file_path
    INITIALIZE empty lists
    FOR each line in the file:
        IF line indicates "NUMBER OF ATOMS":
            SET N = integer value from next line
        ELSE IF line indicates "ATOMS":
            FOR each of the next N lines:
                PARSE: id, mol, type, x, y, z, fx, fy, fz, pe
                COMPUTE force_magnitude = sqrt(fx^2 + fy^2 + fz^2)
                IF (xmin < x < xmax) AND (ymin < y < ymax):
                    SET potential_energy = pe
                ELSE:
                    SET potential_energy = 0
```

```
            APPEND data to corresponding lists
    CONVERT lists to arrays
    RETURN arrays
```

In the simulation data, particles are not stored by their ID. Yarns were built in a way that particles are connected by consecutive IDs, and therefore, by sorting particles by their ID, we can correctly compute distances without requiring a connectivity list.

For each time frame, returned arrays (`id_data`, `mol_data`, `type_data`, `coord_data`, `force_data`, `pe_data`), are rearranged by sorting the IDs of particles in ascending order.

```
FOR each time frame k in [0, T-1]:
    LET id_array = id_data[k]
    LET idx_sort = indices that would sort id_array in ascending order
    REORDER:
        id_data[k]      = id_data[k] sorted by idx_sort
        mol_data[k]     = mol_data[k] sorted by idx_sort
        type_data[k]    = type_data[k] sorted by idx_sort
        coord_data[k]   = coord_data[k] sorted by idx_sort
        force_data[k]   = force_data[k] sorted by idx_sort
        pe_data[k]      = pe_data[k] sorted by idx_sort
```

Calculating the length of each yarn Sorting ensures that for any pair of consecutive particles i and $i+1$, their coordinates can be used for calculating the yarn lenghts, apart end points. The next step consists in grouping yarns be their molecule identifier, so the length of each yarn can be calculated. Grouping starts by iterating over all particles for each timestep, extract the molecule identifier, and place coordinates, $\mathbf{r}$, and their types into a dictionary `yarn_dict`. Then, for each group (yarn), we compute their length, L_m yarn by summing the Euclidean distances between consecutive coordinates:

$$L_m = \sum_{i=0}^{n_m-1} ||\mathbf{r}_{i+1} - \mathbf{r}_i||, \tag{S23}$$

where n_m is the number of particles of yarn m and

$$||\mathbf{r}_{i+1} - \mathbf{r}_i|| = \sqrt{(x_{i+1} - x_i)^2 + (y_{i+1} - y_i)^2 + (z_{i+1} - z_i)^2}. \tag{S24}$$

A JSON file is saved containing the yarn ID, the type, and the length information.

```
FOR each time frame k in [0, T-1]:
    LET coords = coord_atoms[k]
    LET mol_ids = mol_data[k]
    LET types   = atom_type[k]
    INITIALIZE empty dictionary yarn_dict
    FOR each atom index i from 0 to N-1:
        LET m = mol_ids[i]
        IF m is not in yarn_dict:
```

```
            yarn_dict[m] = { 'coords': empty list, 'types': empty list }
        APPEND coords[i] to yarn_dict[m]['coords']
        APPEND types[i] to yarn_dict[m]['types']
    INITIALIZE empty list yarn_info_list
    FOR each molecule m in yarn_dict:
        LET c_list = yarn_dict[m]['coords']
        INITIALIZE yarn_length = 0.0
        FOR i from 0 to (length of c_list) - 2:
            LET p1 = c_list[i]
            LET p2 = c_list[i + 1]
            COMPUTE distance = EuclideanNorm(p2 - p1)
            ADD distance to yarn_length
        LET yarn_type = first element of yarn_dict[m]['types']
        APPEND { 'mol': m, 'type': yarn_type, 'length': yarn_length } to yarn_info_list
    RECORD for time frame k: { 'time': k, 'yarns': yarn_info_list }
    WRITE the collected JSON lengths data to a file
```

Identifying areas between yarns Upon actuation, different patterns might allow more or less changes in the overall structure of the yarn mesh. One of those changes is the fabric "porosity", i.e. the gaps in between the yarns, which might open or close drastically during simulations. Considering yarn meshes, in this work, are mostly bi-dimensional, $L_x \approx L_y \gg L_z$, we can project the mesh into the xy-plane. In this step two list of pores (or pores) are saved, one contains all of the pores areas and the other contains pores bigger than a threshold value and with labels.

In a first stage, for each timestep, function `SaveHoles` starts by creating line segments for every consecutive pair of particles, if the atoms are close enough (the Euclidean distance is smaller than 1.0). Every line segment will have a "buffer" width. This buffer should be the same value as the thickness of each yarn, ideally. However, when pores become small, the projection in the xy-plane might have some definition problem, introducing errors in the labeling section. The two most common issues are highlighted in Fig. S15: (1) yarns might at some point in the simulation close the gap in between so regions previously tracked are not saved anymore; (2) yarns can also come into contact without making regions disappear, but it will result in the split of bigger regions. Therefore, lower values (20% of the yarn thickness) were considered instead. Using lower of yarn thickness for the analysis than the values used in simulation will result in bigger absolute values of areas.

```
FUNCTION SaveHoles(positions_over_time, output_folder, dump_filename, roi_area):
    INITIALIZE dictionary for pore labels, next_label = 1
    INITIALIZE list to store pore data for each time frame
    INITIALIZE list to record filenames for each plot image

    FOR each time frame t from 0 to T-1:
        LET P = array of positions at time t (each position is (x, y, z))
        EXTRACT x and y coordinates from P
        INITIALIZE empty list for buffered_segments

        FOR each consecutive pair (p_i, p_{i+1}) in P:
            IF Euclidean distance ||p_{i+1} - p_i|| < 1:
```

```
        CREATE line segment L between p_i and p_{i+1}
        BUFFER L by radius r (i.e., include all points within distance r of L)
        ADD buffered segment to buffered_segments

    COMPUTE merged_shape = union(buffered_segments)
    polygons = GetPolygons(merged_shape)
    ...
```

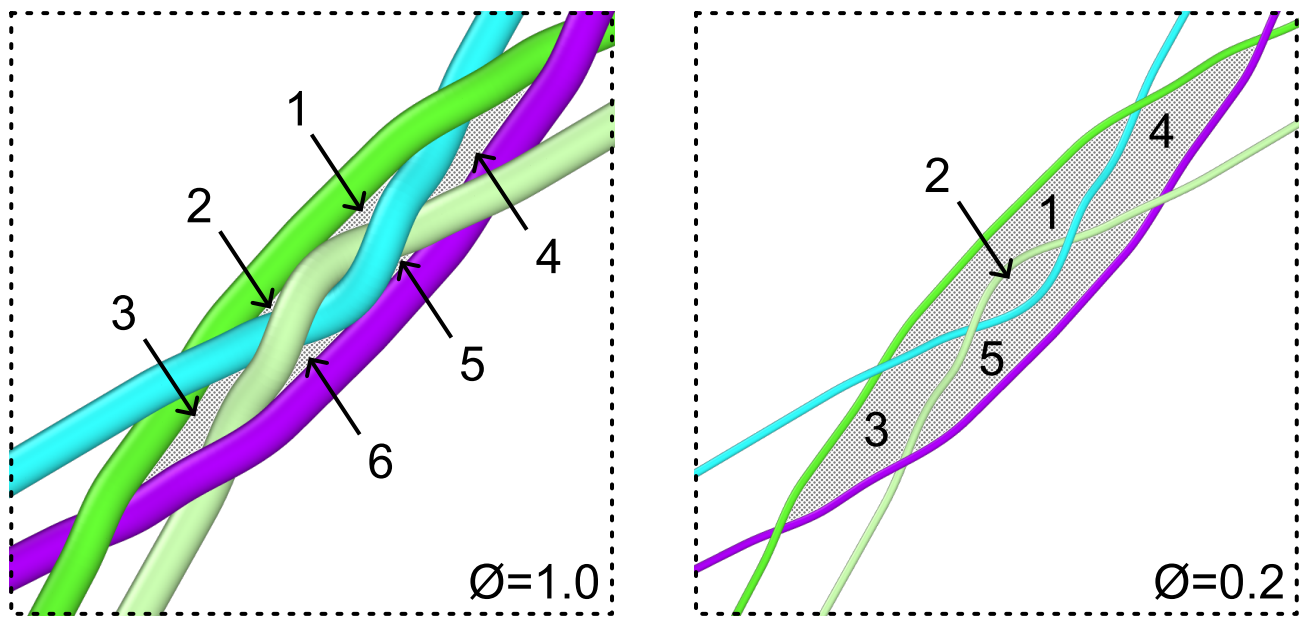

Figure S15: **Snapshot of a simulation illustrating the impact of the yarn thickness.** The gaps formed by the yarns are different when using a bond thickness of 1.0 (left panel) and 0.2 (right panel), giving rise to differences in labeling.

After creating all segments with a buffer, an union operation is performed so it merges all geometries into one. If all the line segments are connected in a way that contains a single area, `polygons` will be a single polygon. If they form multiple disconnected areas, `polygons` will be a list of distinct polygons.

The next section starts by extracting all polygons information. `all_hole_areas` contains only areas of the pores, while `pores_this_frame` will additionally keep the centroid and coordinates. The `MIN_HOLE_AREA` value can be used to filter smaller pores in the case they need to be excluded. Furthermore, all segments are plotted with a dark gray color and areas are colored using the formula:

$$\text{color} = \text{colormap}\left(\frac{\log_{10} A + 1}{\log_{10} A_{\text{max}}}\right) \tag{S25}$$

where A is the area of the pore, and A_{max} is a user defined value so the different generated GIF can be compared among all simulations, and it should be close to the highest observed area. The colormap ranges between $[0, 1]$, and will assign a color of a colormap (in this work we used the `turbo` colormap, more perceptually uniform, and when smaller than 0 or bigger than 1 it will assign the colors of 0 and 1, respectively. The use of the logarithmic formula allows group regions by colors, and give a better color perceptions of areas distribution.

The only missing information is the label of each pore. For the analysis of the simulations it is important to ensure that majority of the label is consistent each timestep, i.e. each pore assigned with a label in the first frame keeps its label in the subsequent iterations. Therefore, in the first frame, a label is assigned to all pores in `pores_this_frame`. In the following frames, the new pores information is compared with the previous `label` dictionary. The value of `MAX_CENTROID_DIST` also sets a maximum distance in which it will ignore pores that move too drastically.

All images of the patterns are saved, Fig. S16 shows one example of the saved image, and then compiled into a GIF, which can be used for tracking the labeling algorithm and give a color perception of the areal change. A JSON file is saved containing the untracked and tracked areas.

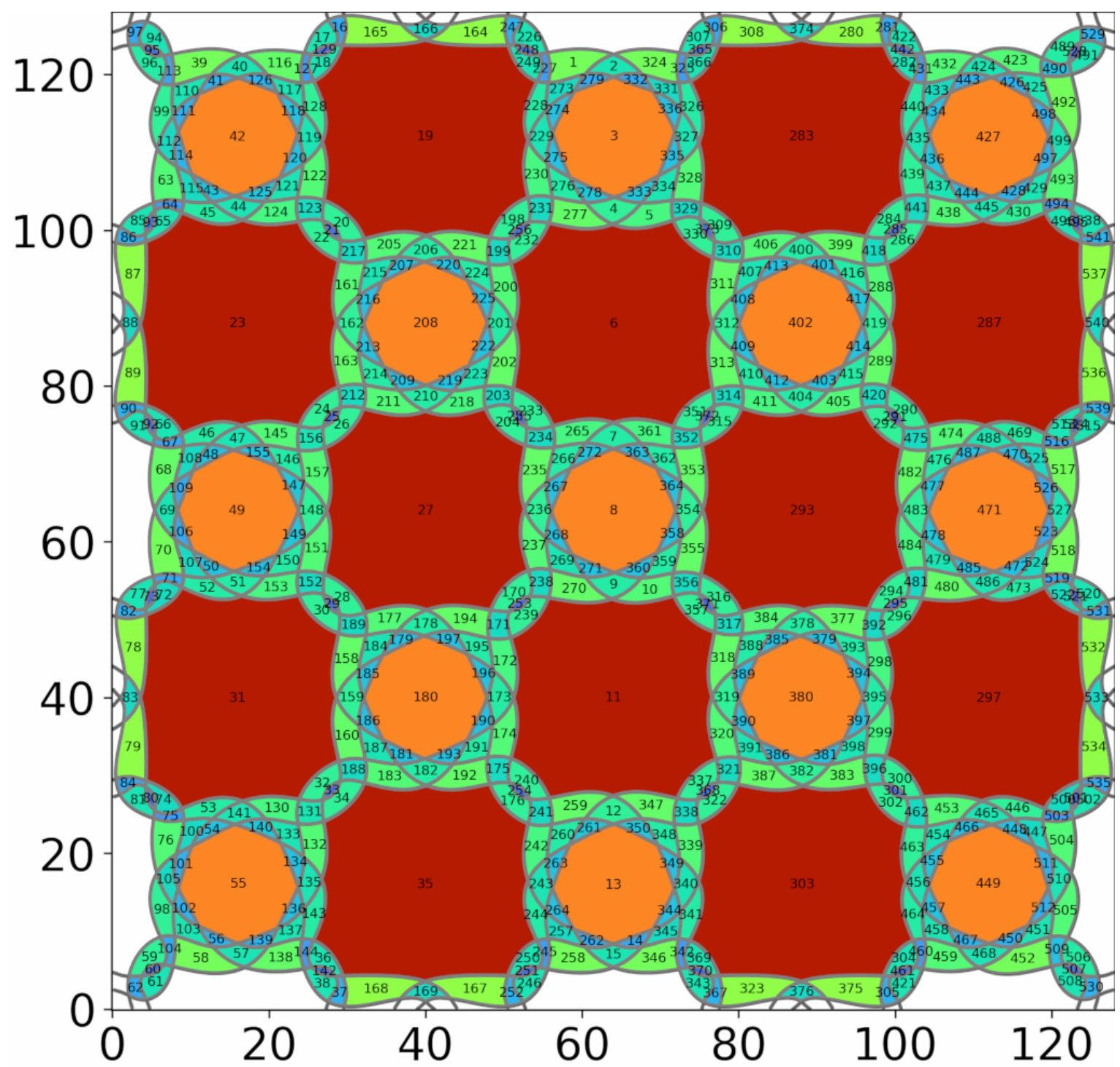

Figure S16: **Example of an image produced by `SaveHoles` function.** The colored areas are scaled logarithmically, so the absolute area differences are less pronounced for larger pores than for smaller ones.

```
... continuation of FUNCTION SaveHoles
        INITIALIZE empty list for all_hole_areas
        INITIALIZE empty list for holes_this_frame
        FOR each polygon in polygons:
            EXTRACT exterior boundary coordinates and add to patches
            FOR each interior boundary (pore) in polygon:
                CONVERT interior boundary into a polygon and COMPUTE area A
                ADD A to all_hole_areas list
                CHOOSE fill color: colormap(log10(A+1) / log10(MAX_AREA))
                CHOOSE edge color: red if A < MIN_HOLE_AREA, otherwise grey
                DRAW pore on plot with determined face and edge colors
                IF A >= MIN_HOLE_AREA:
                    COMPUTE centroid C of the pore
                    STORE pore information (area, centroid, coordinates) in holes_this_frame

        INITIALIZE list frame_labels to store label assignments for this frame
        IF t == 0:
            FOR each pore in holes_this_frame:
                ASSIGN new label = next_label
```

```
            RECORD label and centroid in global pore label dictionary
            ADD {label, area, centroid} to frame_labels
            INCREMENT next_label by 1
    ELSE:
        INITIALIZE list of candidate matches (distance, existing_label, hole_index)
        FOR each pore (with index i) in holes_this_frame:
            LET (c_x, c_y) be its centroid
            FOR each existing label and its centroid (o_x, o_y) in pore label dictionary:
                COMPUTE distance d = sqrt((c_x - o_x)^2 + (c_y - o_y)^2)
                IF d < MAX_CENTROID_DIST:
                    ADD candidate match (d, label, i) to candidate list
        SORT candidate matches by distance (smallest first)
        ASSIGN labels one-to-one based on the sorted candidates (avoiding duplicates)
        UPDATE global pore label dictionary with new centroid positions for matched pores
        FOR pores not matched:
            ASSIGN new label = next_label, update dictionary, and add to frame_labels
    ADD text labels to the plot at each pore's centroid
    STORE frame data (time, all pore areas, and labeled pores) in the JSON data list
    SET plot limits based on the min and max of x and y (with a margin equal to r)
    SAVE plot image to file, record the filename, and close the plot
WRITE the collected JSON pore data to a file
COMPILE all saved images into an animated GIF and save it
```

Variation of yarn lengths (simulated laces)

The purpose of this code was to read JSON data files containing the lengths of yarns and then producing plots with the relative variation of length grouped by the yarn type. The `plot_lengths` starts by reading the JSON data. Each entry is a dictionary with:

- `time`, the frame of the simulation;
- `yarns`, a list of structures containing:
  - `mol`, the yarn identifier;
  - `type`, the yarn type, identifying groups of yarns that are active (the ones that actuate) and passive;
  - `length`, the absolute length of the yarn at the above time

The baseline value considered for the initial length was their value in the first instant $L_0 = L(t = 0)$. The relative variation is given by:

$$l(t) = \frac{L(t) - L_0}{L_0} \tag{S26}$$

If L_0 is below a threshold `filter_L0`, which was set as half of the simulation box width, then the yarn is skipped. This analysis considers the variation of yarns, and smaller yarns, for instance yarns close to the border, are likely to have most of their path constricted of moving, which introduces information that has little relevance to this analysis.

Yarns with the same type are then grouped, and the plots and JSON files are saved. Fig.

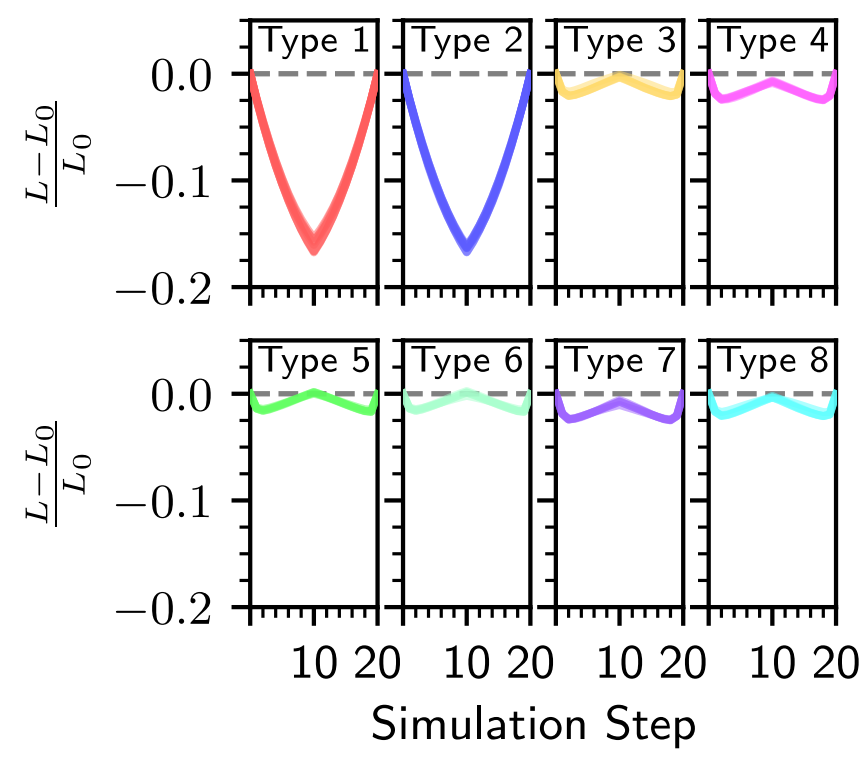

Figure S17: **Example of an image produced by `plot_lengths` function.** The 8 subplots show the variation of the relative length of yarns grouped by types. In this case, it can be easily assessed what happened in a simulation where yarns with types 1 and 2 were actuated.

```
FUNCTION plot_lengths(json_file, pattern_folder, dump_file):
    OPEN file at json_file FOR reading
    PARSE JSON data INTO variable data
    INITIALIZE dictionary yarn_dict
    INITIALIZE empty dictionary type_groups
    FOR EACH key = (mol_val, typ_val) IN yarn_dict DO:
        CONVERT yarn_dict[key]["times"] TO array times_array
        CONVERT yarn_dict[key]["lengths"] TO array lengths_array
        SET L0 = first element of lengths_array
        IF L0 < FILTER_L0 THEN:
            CONTINUE to next key
        SET relvar = (lengths_array - L0) / L0
        APPEND { "times": times_array, "relvar": relvar, "mol": mol_val } TO type_groups[typ_val]

    CREATE figure WITH 2 rows and 4 columns of subplots
    ASSIGN subplots TO variable axes
    SAVE figure
    WRITE the collected JSON pore data to a file
```

Pattern areal variation (physical and simulated laces)

The script used to measure the dynamics of the areal data handles both `.json` files from physical and simulated data, but the analysis is branched into two different workflows. For physical samples, the pore detection algorithm does not track individual pores by ID. Therefore, the code treats each frame as an independent distribution of areas, converts those areas to $\log_{10}(A+1)$, fits a weighted Epanechnikov kernel density estimate, with weights proportional to area. For the most cases, the biggest peaks in the density distribution was followed, and in some relevant cases where that peak split into two, then the peaks were tracked separately. Each peak value is converted to square-millimeter units, and its relative change is plotted. The workflow produces two different types of histogram distribution dynamics, (1) a magma-colored contour

map of density of time (not shown in this work) and (2) waterfall-like plot with color coded-histograms that show of the dynamics of the peaks shift over time, and (3) the relative-change curve(s) with the last point annotated. For example, for the lace structure Rose-12 it can be obtained:

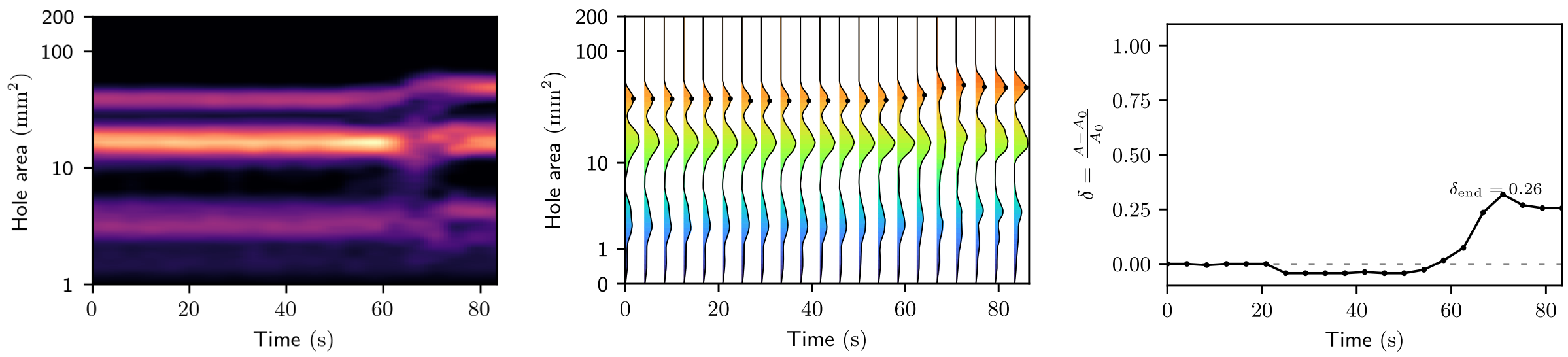

Figure S18: **Example of plots produced by `workflow_physical` function**

For simulated samples, both untagged and tagged pores are recorded, allowing the code to extract a complete time series for each individual pore. Each pore's area at step 0 serves as its baseline, and the algorithm then computes the per-label relative change $(A_t - A_0)/A_0$. So, the simulated workflow tracks how every individual area grows or shrinks, with both negative and positive contributions included in the overall areal change. Note, however, that pores sharing the same area but differing in shape register no difference in this analysis. Even so, this approach provides a first, quantifiable measure of a pattern's overall dynamics. Besides the pore metrics, the algorithm also calculates the overall, baseline-weighted curve that combines all per-pore changes into a singular trend, a separate trajectory (optional) for a subset of larger pores (for comparison with the main trend), and a loss-fraction line, defined at each step as the difference between the frame's total number of areas and the portion still assigned to tracked label, since some labels are dropped due to become too small or hard to track, normalized by the total. Together, these quantities help with the dynamics of the pore tracking.

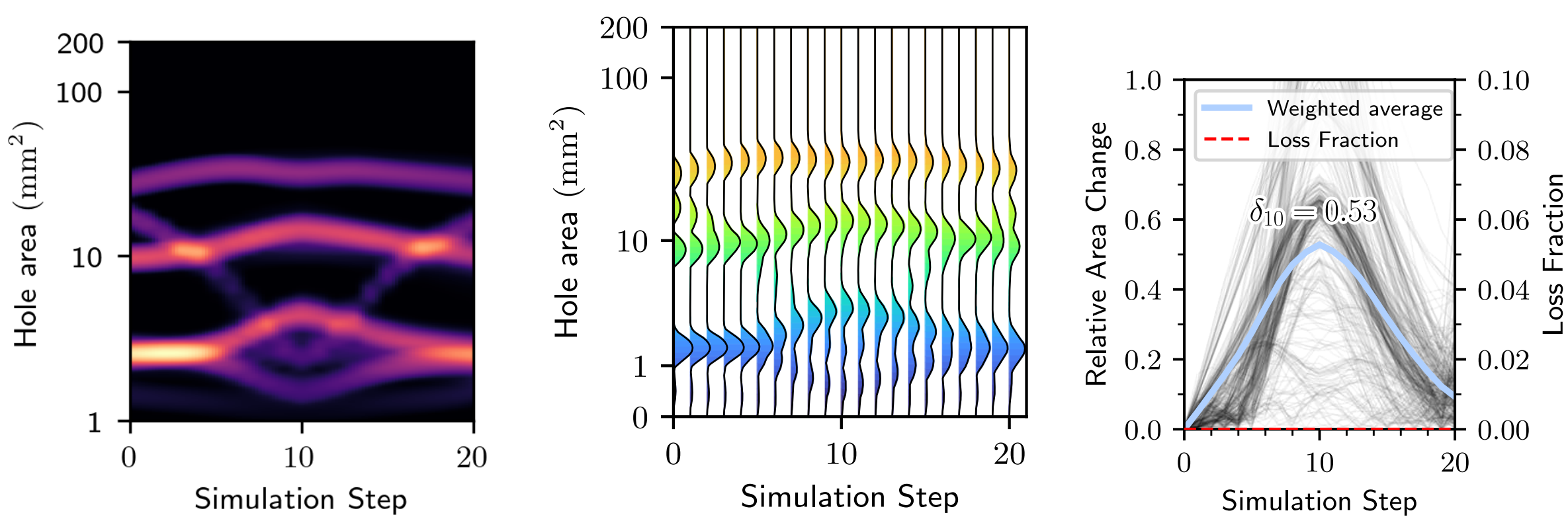

Figure S19: **Example of plots produced by `workflow_simulated` function.**

The examples provided for the two different workflows showcase how physical and simulated samples are processed differently. Physical samples' actuation is time-dependent, non-reversible, and smaller areas are not easily detected due to experimental constraints. Therefore, for the physical samples, we focus on the largest areas where the variation is clearly identified and is representative in most cases of the dynamics of a

given pattern. In simulation, by contrast, every pore is tracked individually, so even small pores that would tend to blur or vanish in physical tests are not lost for the analysis. Finally, the physical experiments are time-dependent, and the activation of the fabrics is not homogeneous in some real-life cases. In the case of the simulations, the activation is controlled by steps of yarns shrinking rather than time activation.

```
FUNCTION plot_from_json(json_path, pattern_dir, label, split=False, bw=0.1):

    READ holes_data FROM json_path
    CREATE plots_dir IF it does not exist
    IF analysing simulated sample THEN:
        CALL workflow_simulated
    ELSE:
        CALL workflow_physical

FUNCTION workflow_physical(holes_data, split=False, bw=0.1):

    FOR EACH step_index, step IN holes_data DO:
        areas = step['areas'] WHERE area >= min_area
        LOG_TRANSFORM  proc = log10(areas + 1)
        FIT weighted Epanechnikov KDE to proc USING weights = areas
        STORE density_x[step_index], density_y[step_index]
        FIND peaks IN density_y
            IF split==True  THEN keep two most-prominent peaks
            ELSE                 keep last (largest) peak only
        CONVERT chosen peak(s) back to mm² scale
        SAVE to series  peak_area_small[t], peak_area_large[t]  (or one series)

    IF split==True:
        delta_small[t]  = (peak_area_small[t] - peak_area_large[0]) / peak_area_large[0]
        delta_large[t]  = (peak_area_large[t] - peak_area_large[0]) / peak_area_large[0]
    ELSE:
        delta[t]        = (peak_area[t] - peak_area[0]) / peak_area[0]

    SAVE Plots

FUNCTION workflow_simulated(holes_data, ...):

    DEFINE big_hole_threshold = user-supplied value
    SET big_labels = { l | baseline[l] >= big_hole_threshold }
    INITIALISE dict areas_by_label
    FOR EACH step IN holes_data DO:
        FOR EACH (label, area) IN step['tagged_areas']:
            APPEND area TO areas_by_label[label]

    SET baseline[label] = areas_by_label[label][0]    FOR EVERY tracked label
    FOR EACH t IN simulation_steps DO:
        FOR EACH label:
            rel[label][t] = (areas_by_label[label][t] - baseline[label]) / baseline[label]
```

```
    overall_rel[t] = numerator / denominator

    SET big_num   = Sum( baseline[l] * rel[l][t] )   FOR l in big_labels
    SET big_den   = Sum( baseline[l] )               FOR l in big_labels
    big_rel[t]    = big_num / big_den

    total_area_frame   = step_total_area[t]
    tracked_area_frame = Sum( areas_by_label[l][t] ) FOR all labels l
    IF total_area_frame > 0 THEN
        loss_fraction[t] = (total_area_frame - tracked_area_frame) / total_area_frame
    ELSE
        loss_fraction[t] = 0

SAVE Plots
```

Supplementary Results

Experimental results

Analysis of lace deformation over time are visualized in four panels:

- Contraction of textile's unit-cell scheme: A drawn schematic diagram of the yarn paths in the structure. The diagram shows one repeating unit or, if it is not square-shaped, two units. The position of active yarns (pemotex) is marked in red, and passive ones (cotton) are marked in gray. Fig. S20
- Time-lapse visualization of the textile structure under deformation: Photographs from the beginning and end of the video taken of the activation, as well as three intermediate phase images. The images are color-coded to indicate the pores between the yarns identified by image analysis. The largest pores are marked in red, and the color gradient shifts towards blue as the pores size decrease. Sec.
- Evolution of the distribution of pore areas over time: The waterfall graph shows how the number of pores of different sizes varies in the activated lace over time. The color gradient corresponds to the color coding of the pores marked in the photographs. Normalized pore area change of the largest areas is marked on the graph with a black dot. Sec.
- Normalized pore area change of the largest areas: The normalized plot, which serves as a summary of the data presented in the previous graph, indicates the direction of change in the size of the pores. Sec.

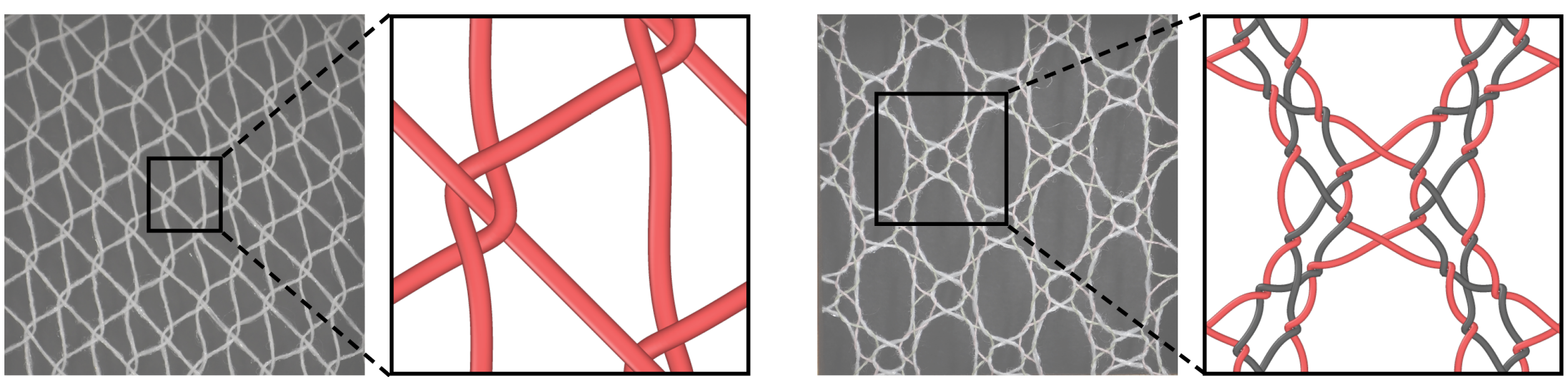

Figure S20: Unit-cell is a square shaped repeating part of the pattern. Two examples of parts selection. Shrinking yarns are presented with red color and passive yarns are gray.

Sample	Cross	Twist	Moves (C+T)	Active threads (%)	Straight threads (%)	Area change
Diamond-1	8	16	24	100	100	0.05
Diamond-2	8	48	56	100	0	-0.04
Diamond-3	32	32	64	50	0	0.19
Diamond-4	32	32	64	50	0	0.11
Diamond-5	32	32	64	50	0	0.59
Diamond-6	72	72	144	50	0	0.02
Diamond-7	16	16	32	100	33	-0.01
Hexa-1	20	16	36	50	50	0.62
Rose-1	24	28	52	12.5	0	0.35
Rose-1	24	28	52	25	0	0.53
Rose-1	24	28	52	25	0	0.62
Rose-1	24	28	52	25	0	0.63
Rose-1	24	28	52	50	0	0.75
Rose-1	24	28	52	25	0	0.79
Rose-1	24	28	52	50	0	0.89
Rose-1	24	28	52	50	0	0.9
Rose-1	24	28	52	25	0	0.9
Rose-1	24	28	52	50	0	0.95
Rose-1	24	28	52	50	0	0.95
Rose-1	24	28	52	100	0	0.97
Rose-10	40	40	80	50	50	0.43
Rose-11	12	48	60	50	0	-0.02
Rose-11	12	48	60	50	0	0.13
Rose-12	8	20	28	25	75	-0.16
Rose-12	8	20	28	25	0	0.26
Rose-12	8	20	28	100	0	0.35
Rose-13	12	28	40	100	50	0.32
Rose-2	32	36	68	100	0	0.44
Rose-3	20	40	60	100	0	0.3
Rose-4	24	48	72	12.5	0	0.22
Rose-4	24	48	72	100	0	0.27
Rose-4	24	48	72	25	0	0.31
Rose-4	24	48	72	50	0	0.43
Rose-4	24	48	72	50	0	0.45
Rose-5	40	80	120	100	0	0.32
Rose-6	20	32	52	50	50	0.68
Rose-7	24	24	48	50	0	0.63
Rose-8	12	24	36	50	25	0.13
Rose-8	12	24	36	50	25	0.13
Rose-9	14	28	42	50	0	0.07
Rose-9	14	28	42	50	0	0.07
Rose-9	14	28	42	50	0	0.1
Rose-9	14	28	42	50	0	0.13
Spruce-1	14	28	42	50	0	0.37
Square-1	64	64	128	100	0	0.09
Square-2	36	48	84	100	50	-0.04

Table S1: Textile Sample Data

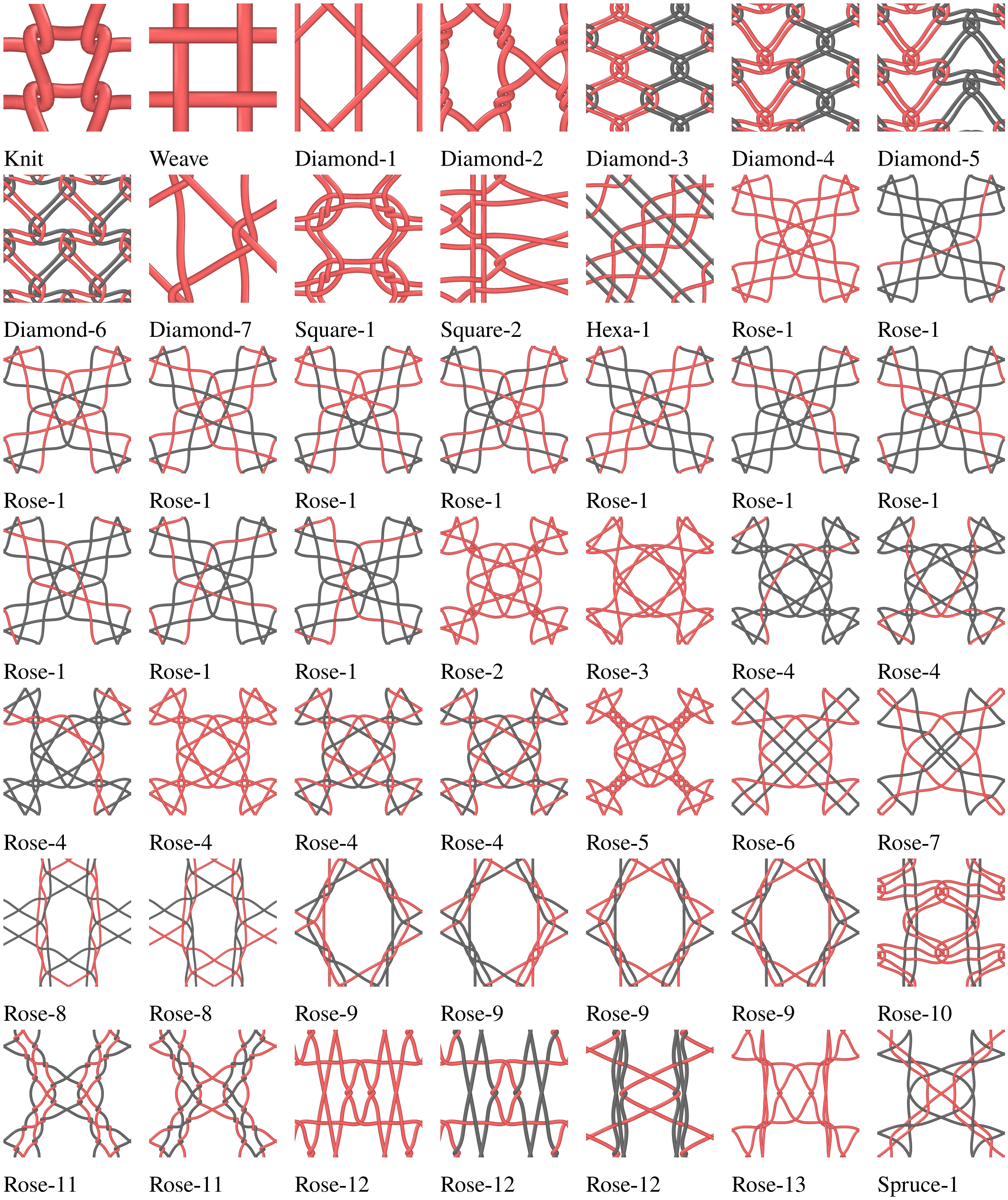

Figure S21: All schematics os textile structures. The position of active yarns (pemotex) is marked in red, and passive ones (cotton) are marked in gray.

Knit

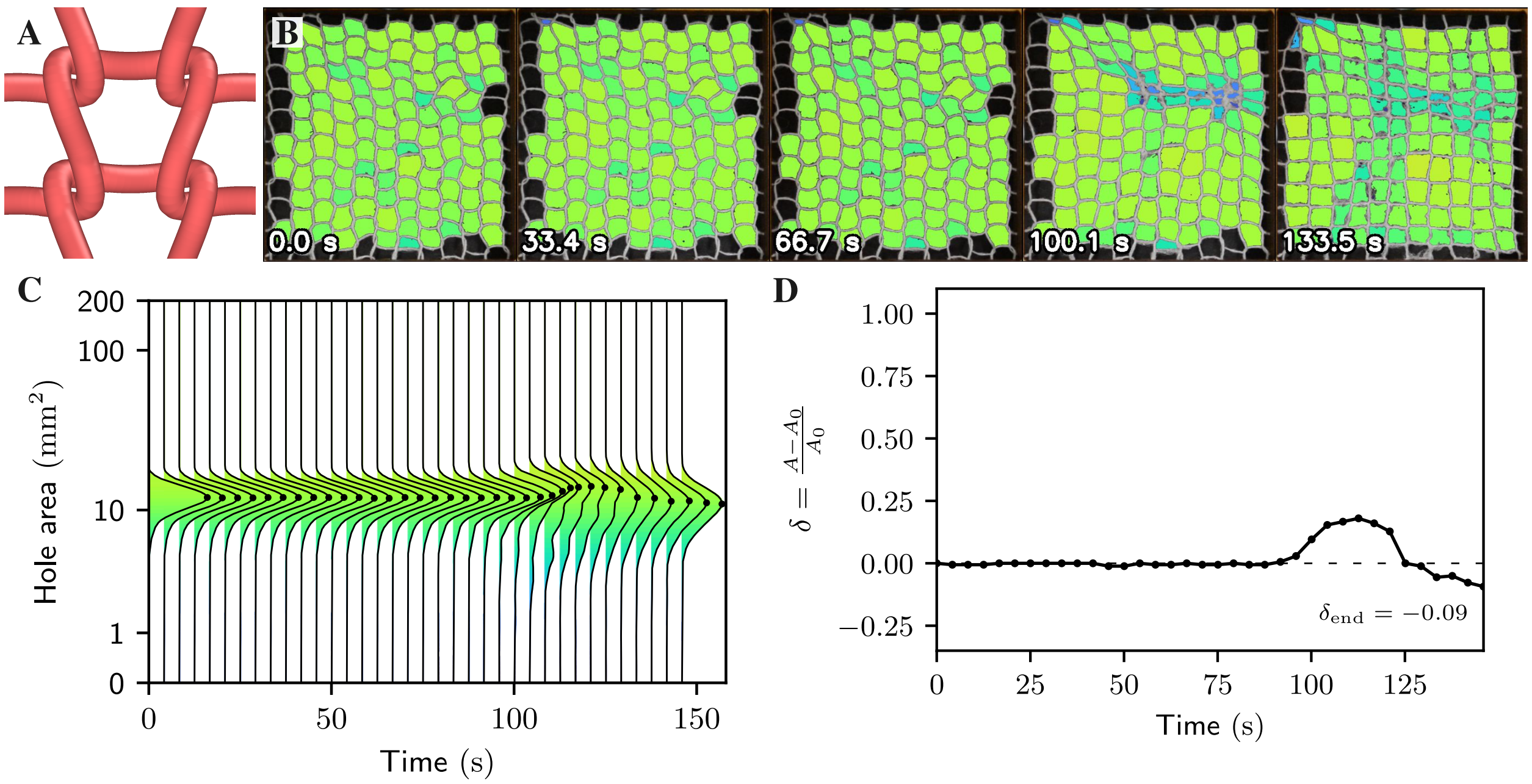

Figure S22: **Analysis of a knit structure deformation over time. a** Contraction of textile's unit-cell scheme (red yarns shrink). **b** Time-lapse visualization of the textile structure under deformation. **c** Evolution of the distribution of pore areas over time. **d** Normalized pore area change of the largest areas.

Weave

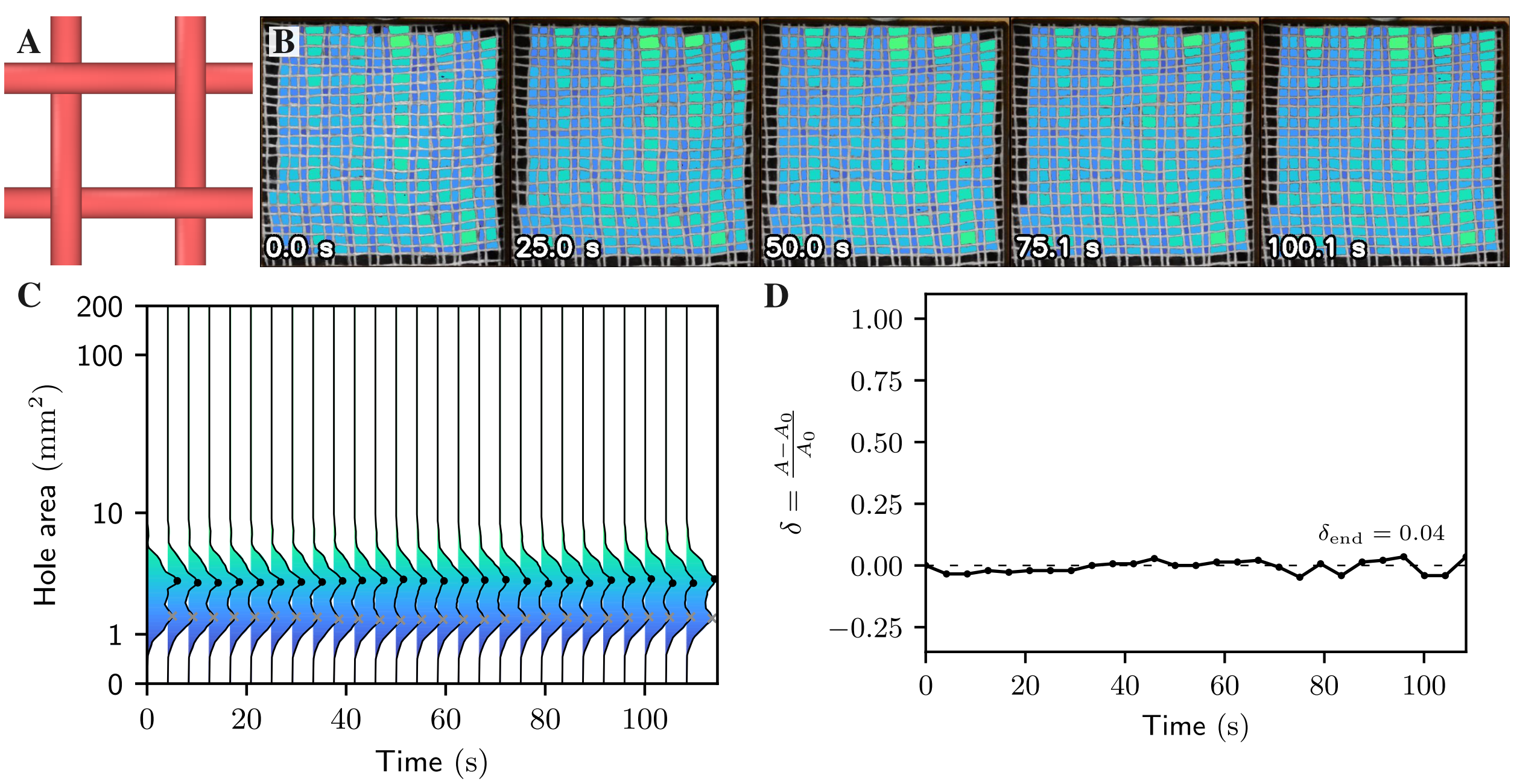

Figure S23: **Analysis of a plain weave structure deformation over time. a** Contraction of textile's unit-cell scheme (red yarns shrink). **b** Time-lapse visualization of the textile structure under deformation. **c** Evolution of the distribution of pore areas over time. **d** Normalized pore area change of the largest areas.

Diamond-1

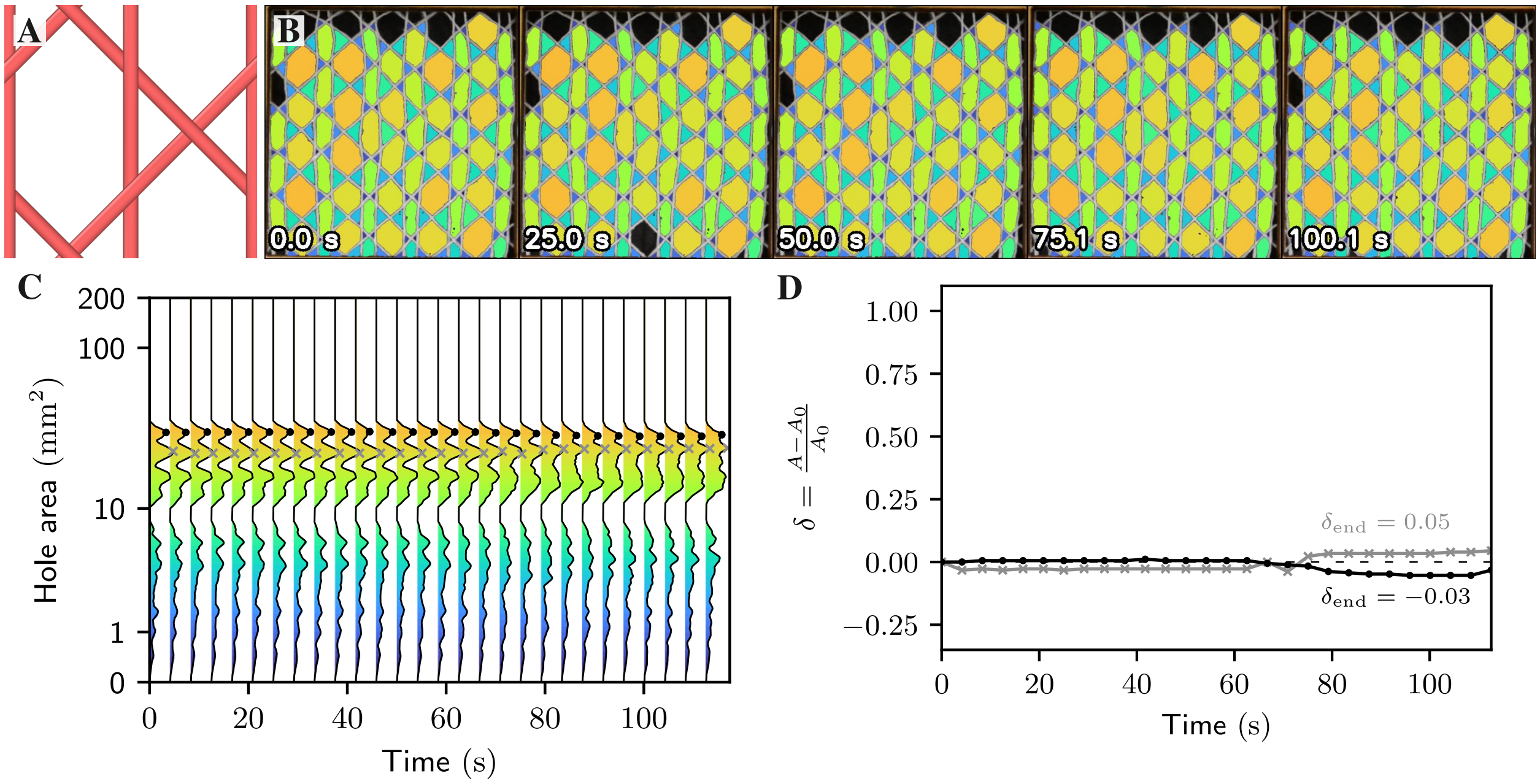

Figure S24: **Analysis of lace structure Diamond-1 deformation over time. A** Contraction of textile's unit-cell scheme (red yarns shrink). **B** Time-lapse visualization of the textile structure under deformation. **C** Evolution of the distribution of pore areas over time. **D** Normalized pore area change of the largest areas.

Diamond-2

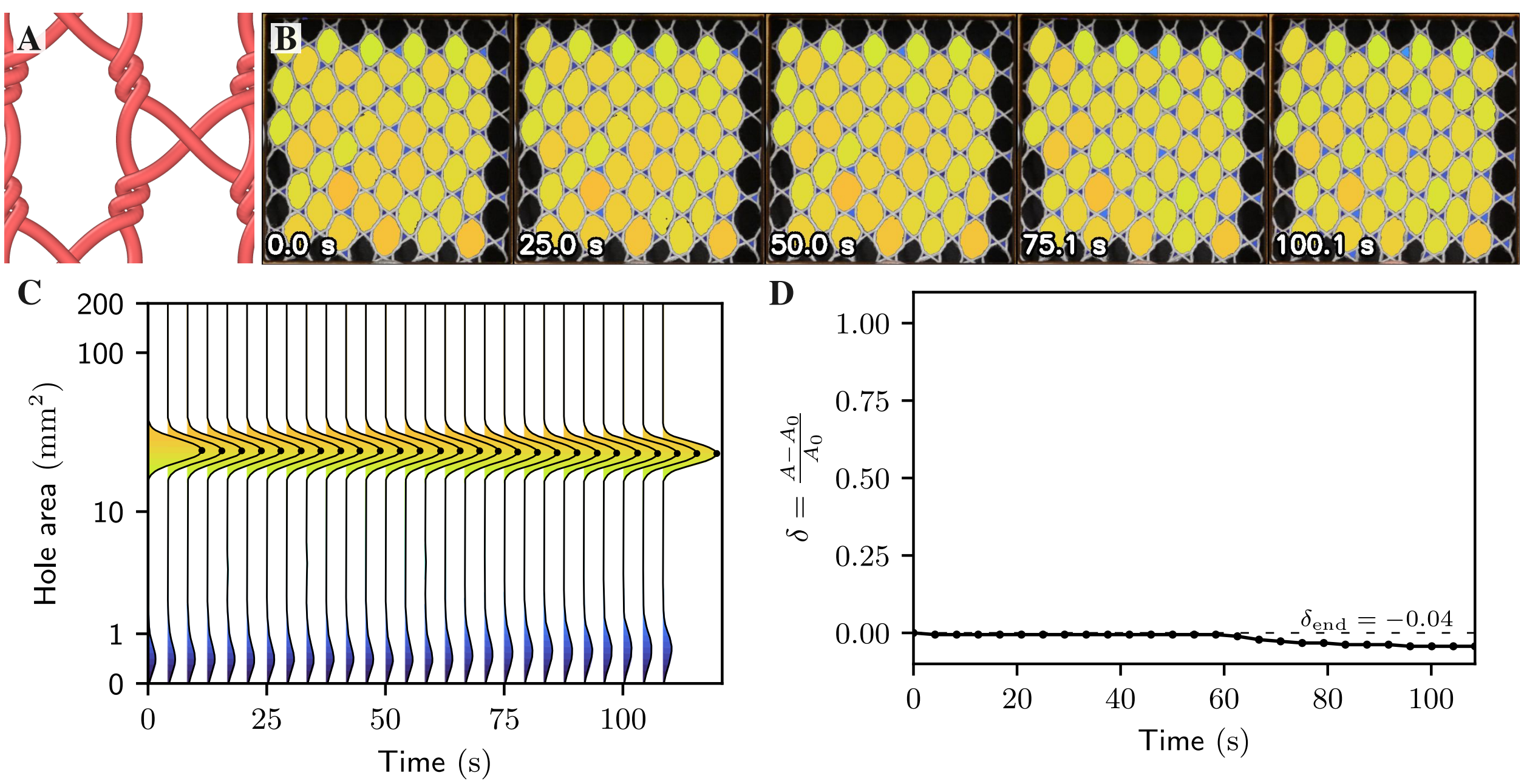

Figure S25: **Analysis of lace structure Diamond-2 deformation over time. A** Contraction of textile's unit-cell scheme (red yarns shrink). **B** Time-lapse visualization of the textile structure under deformation. **C** Evolution of the distribution of pore areas over time. **D** Normalized pore area change of the largest areas.

Diamond-3

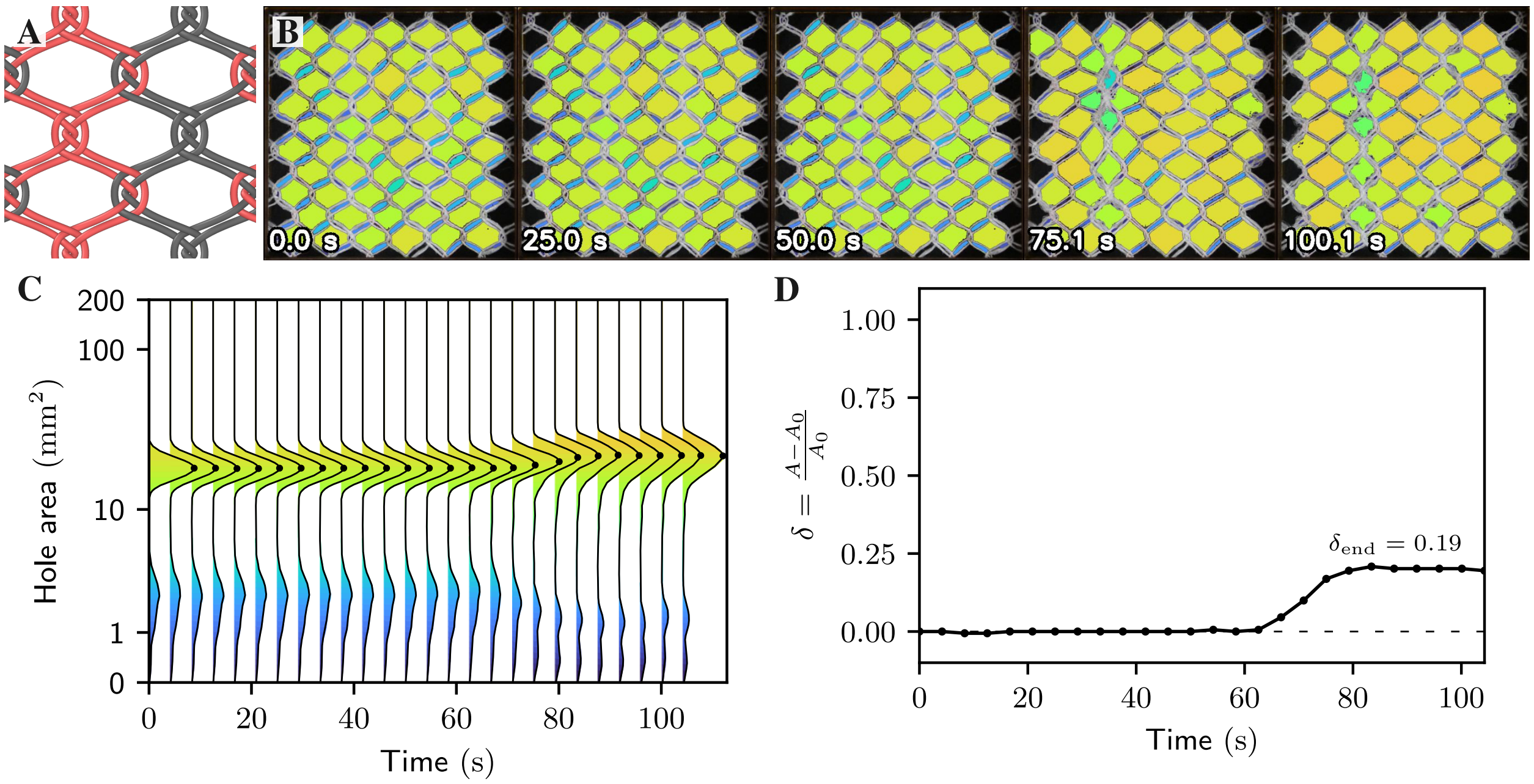

Figure S26: **Analysis of lace structure Diamond-3 deformation over time. A** Contraction of textile's unit-cell scheme (red yarns shrink). **B** Time-lapse visualization of the textile structure under deformation. **C** Evolution of the distribution of pore areas over time. **D** Normalized pore area change of the largest areas.

Diamond-4

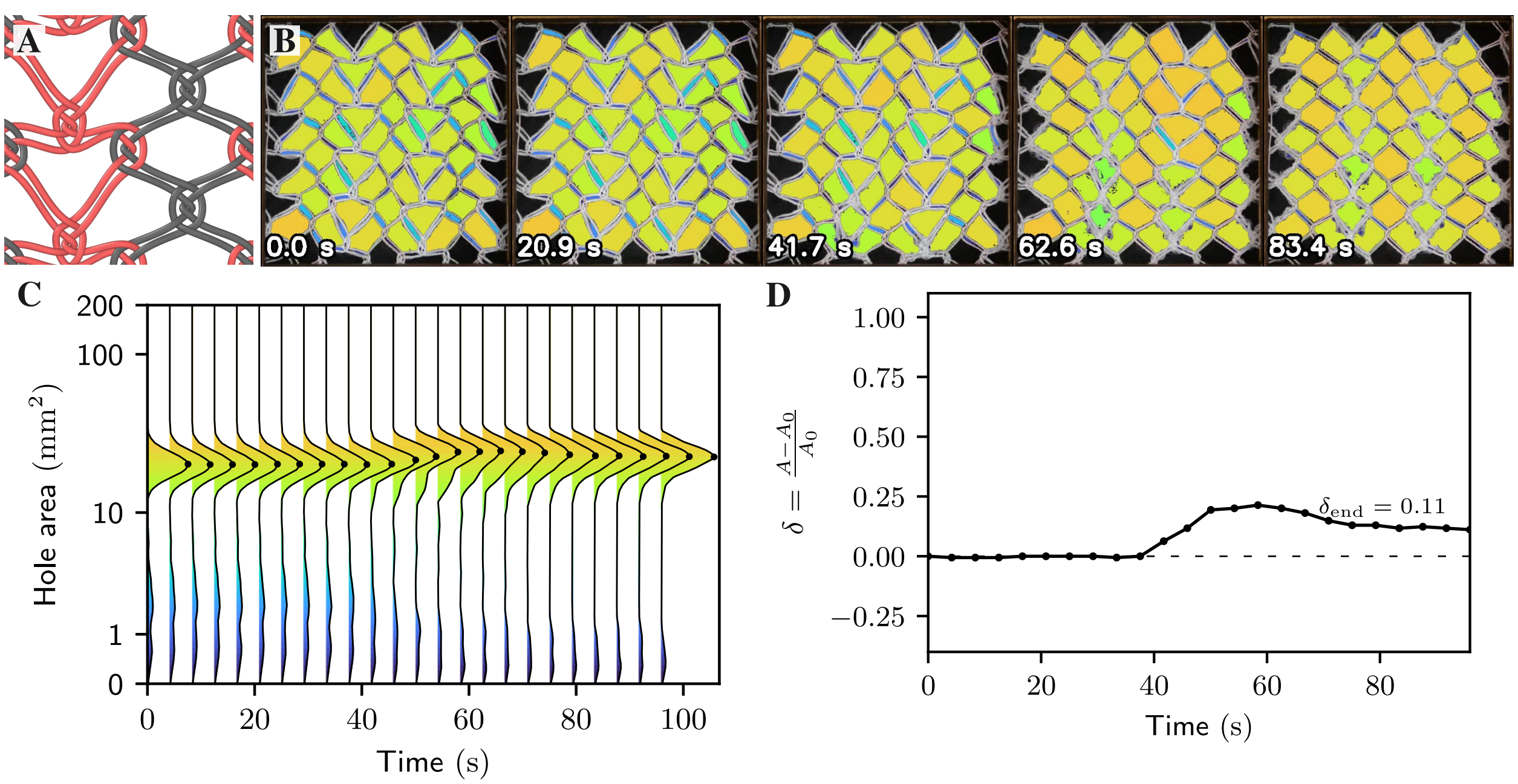

Figure S27: **Analysis of lace structure Diamond-4 deformation over time. A** Contraction of textile's unit-cell scheme (red yarns shrink). **B** Time-lapse visualization of the textile structure under deformation. **C** Evolution of the distribution of pore areas over time. **D** Normalized pore area change of the largest areas.

Diamond-5

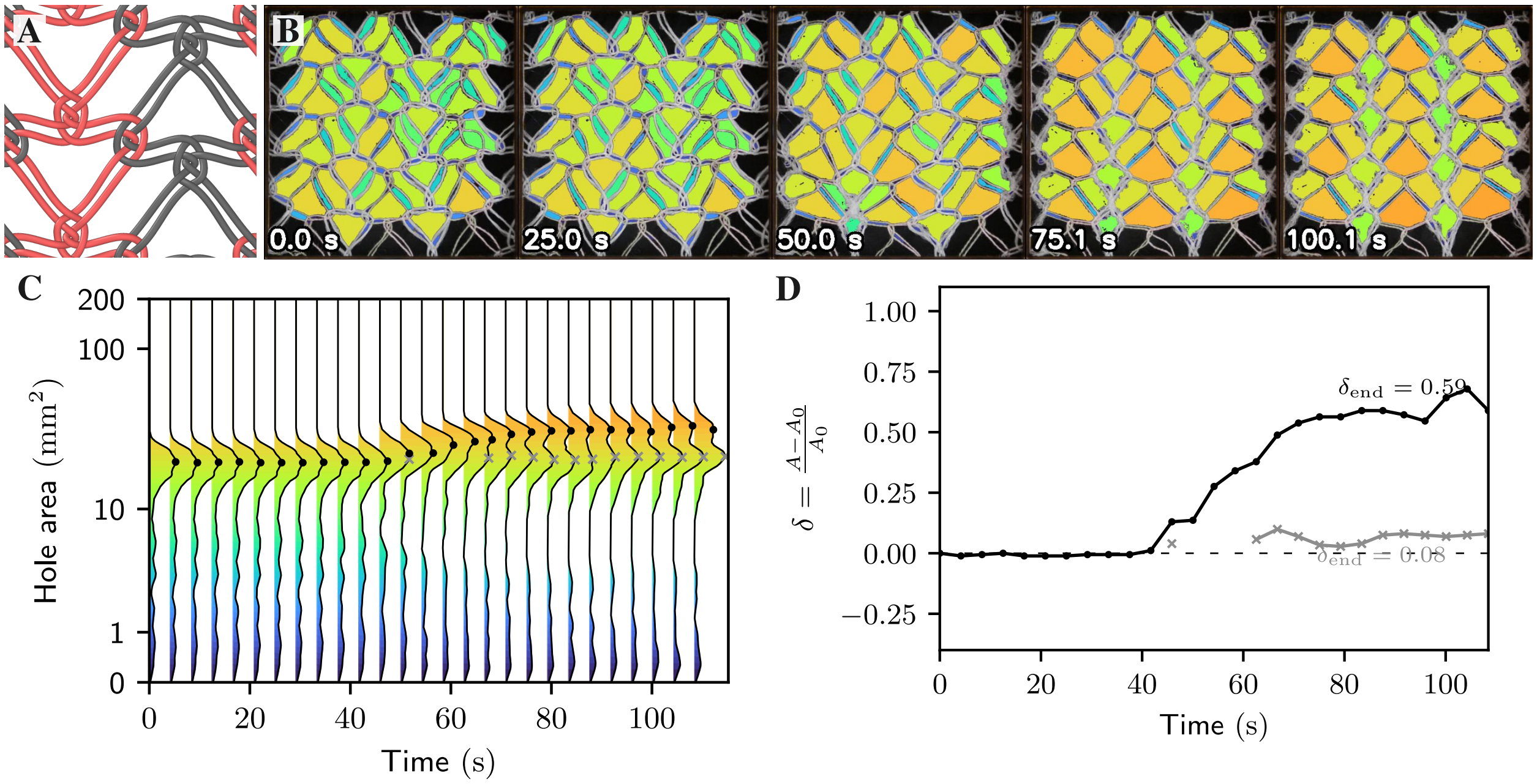

Figure S28: **Analysis of lace structure Diamond-5 deformation over time. A** Contraction of textile's unit-cell scheme (red yarns shrink). **B** Time-lapse visualization of the textile structure under deformation. **C** Evolution of the distribution of pore areas over time. **D** Normalized pore area change of the largest areas.

Diamond-6

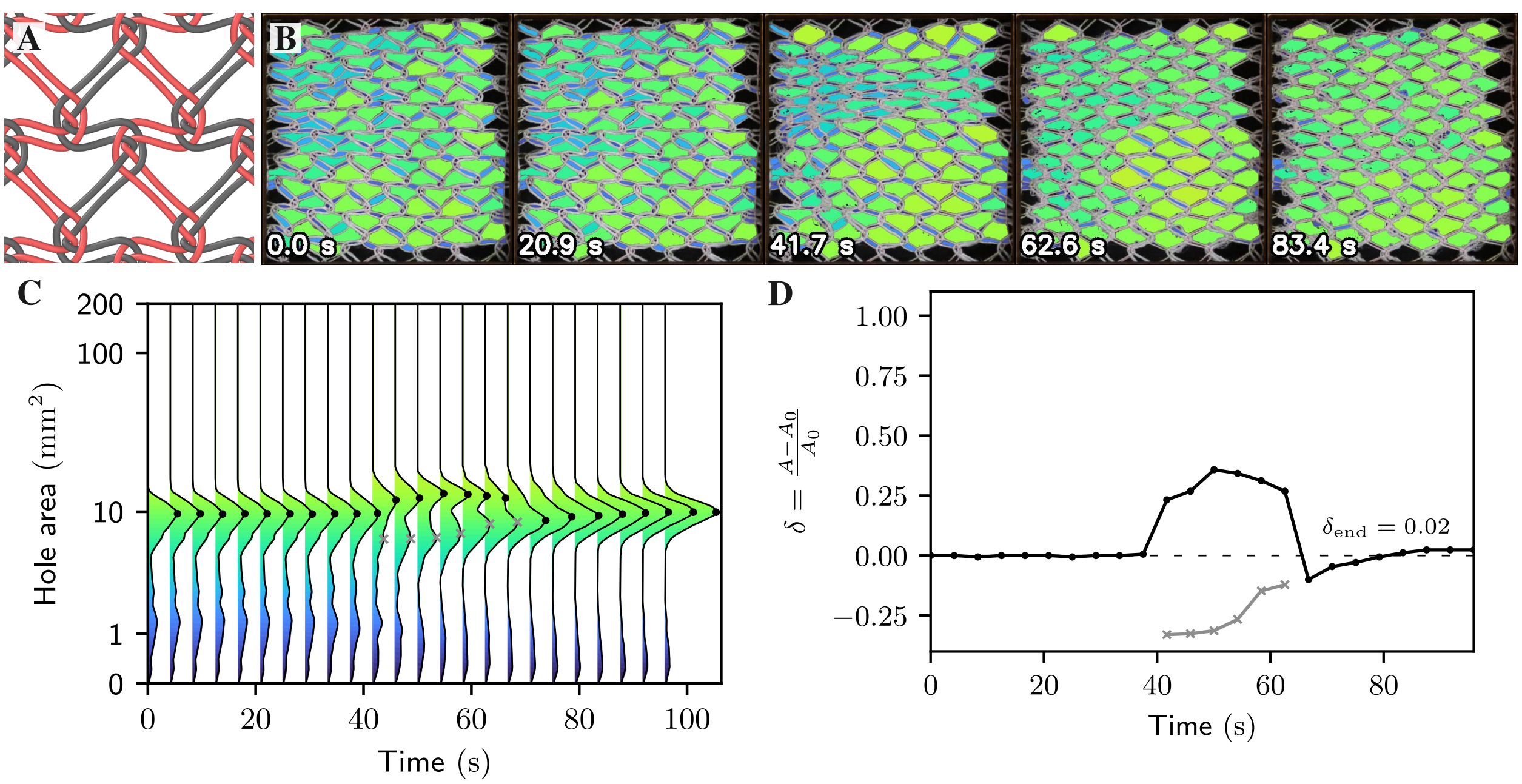

Figure S29: **Analysis of lace structure Diamond-6 deformation over time. A** Contraction of textile's unit-cell scheme (red yarns shrink). **B** Time-lapse visualization of the textile structure under deformation. **C** Evolution of the distribution of pore areas over time. **D** Normalized pore area change of the largest areas.

Diamond-7

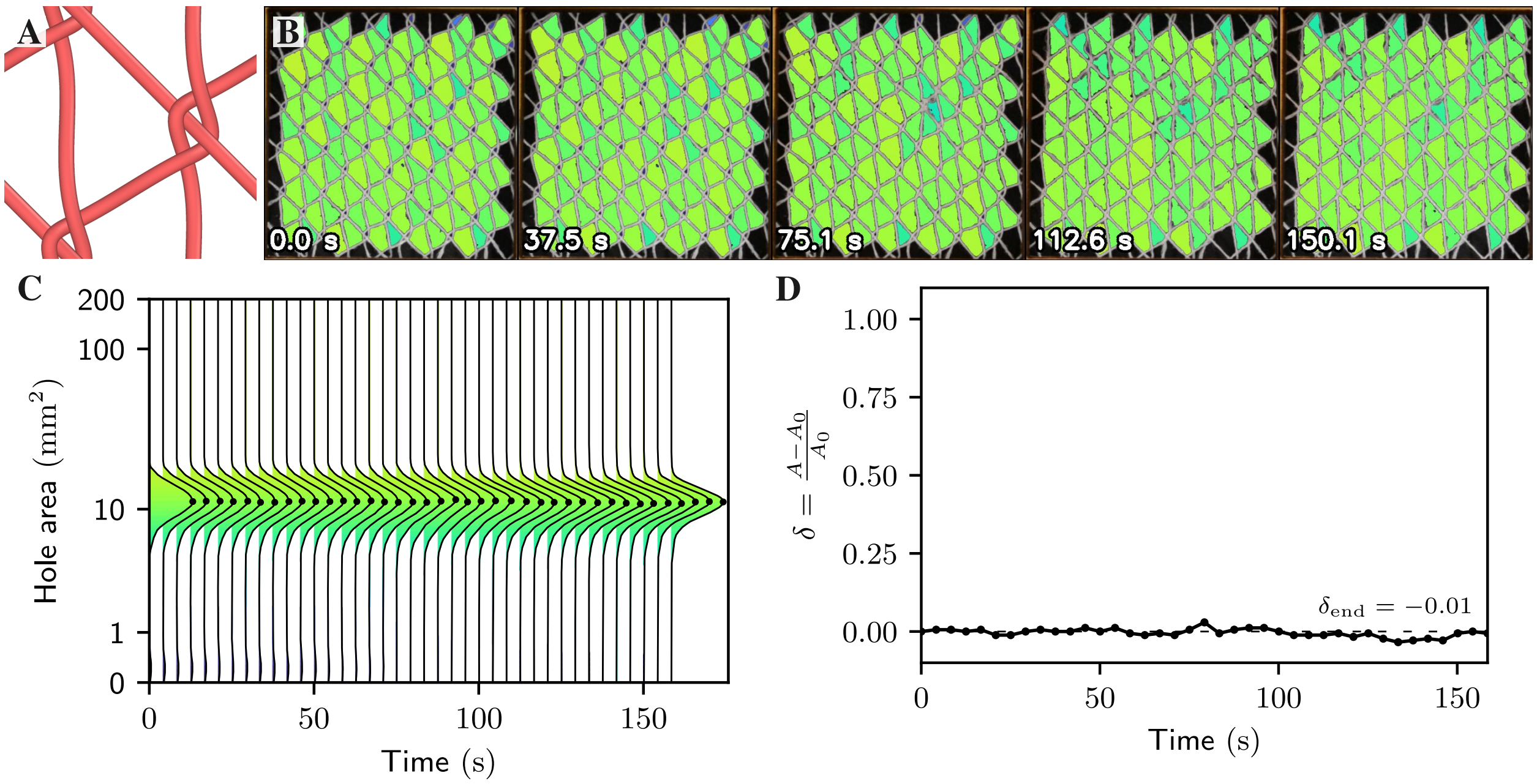

Figure S30: **Analysis of lace structure Diamond-7 deformation over time. A** Contraction of textile's unit-cell scheme (red yarns shrink). **B** Time-lapse visualization of the textile structure under deformation. **C** Evolution of the distribution of pore areas over time. **D** Normalized pore area change of the largest areas.

Square-1

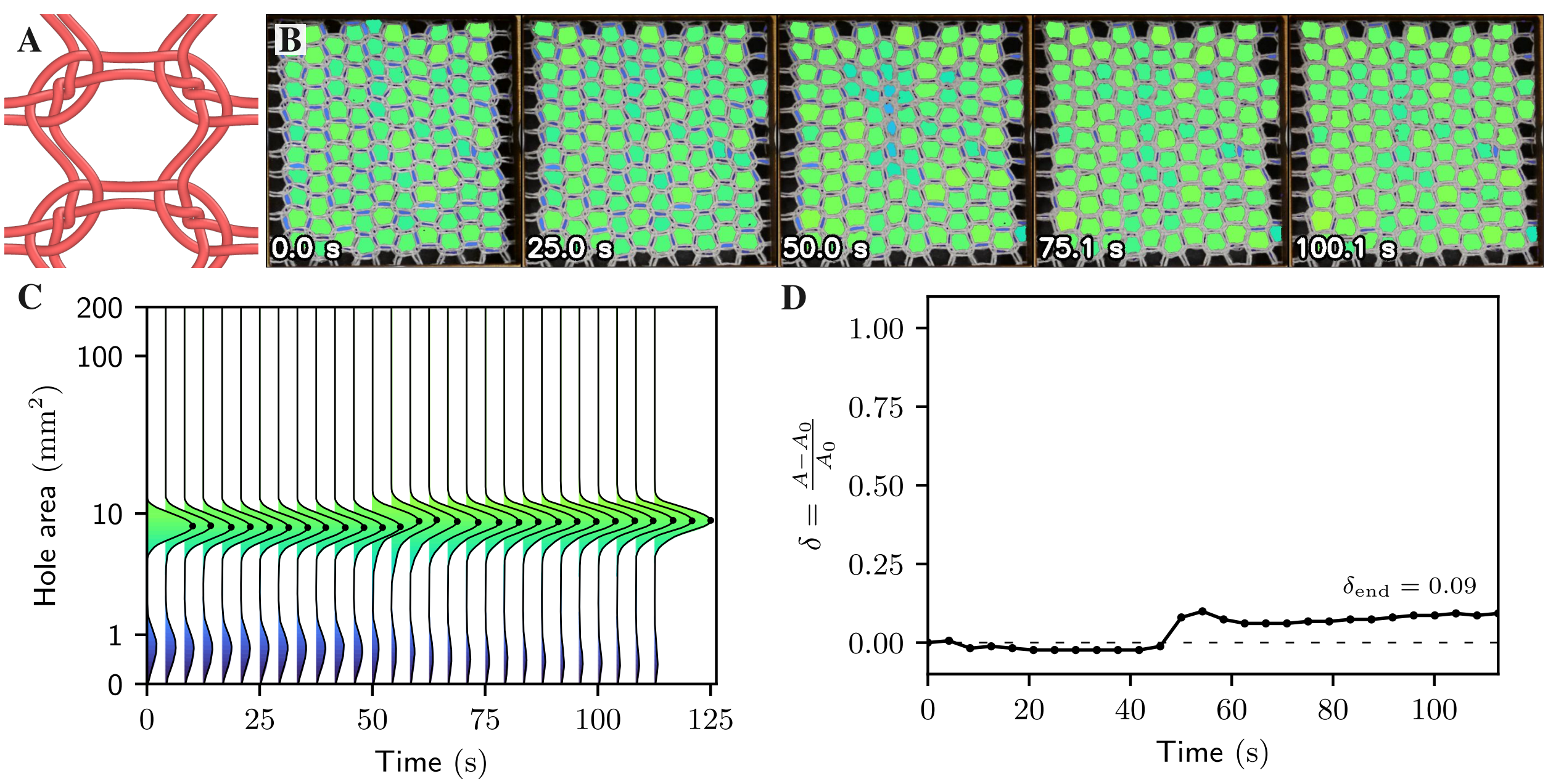

Figure S31: **Analysis of lace structure Square-1 deformation over time. A** Contraction of textile's unit-cell scheme (red yarns shrink). **B** Time-lapse visualization of the textile structure under deformation. **C** Evolution of the distribution of pore areas over time. **D** Normalized pore area change of the largest areas.

Square-2

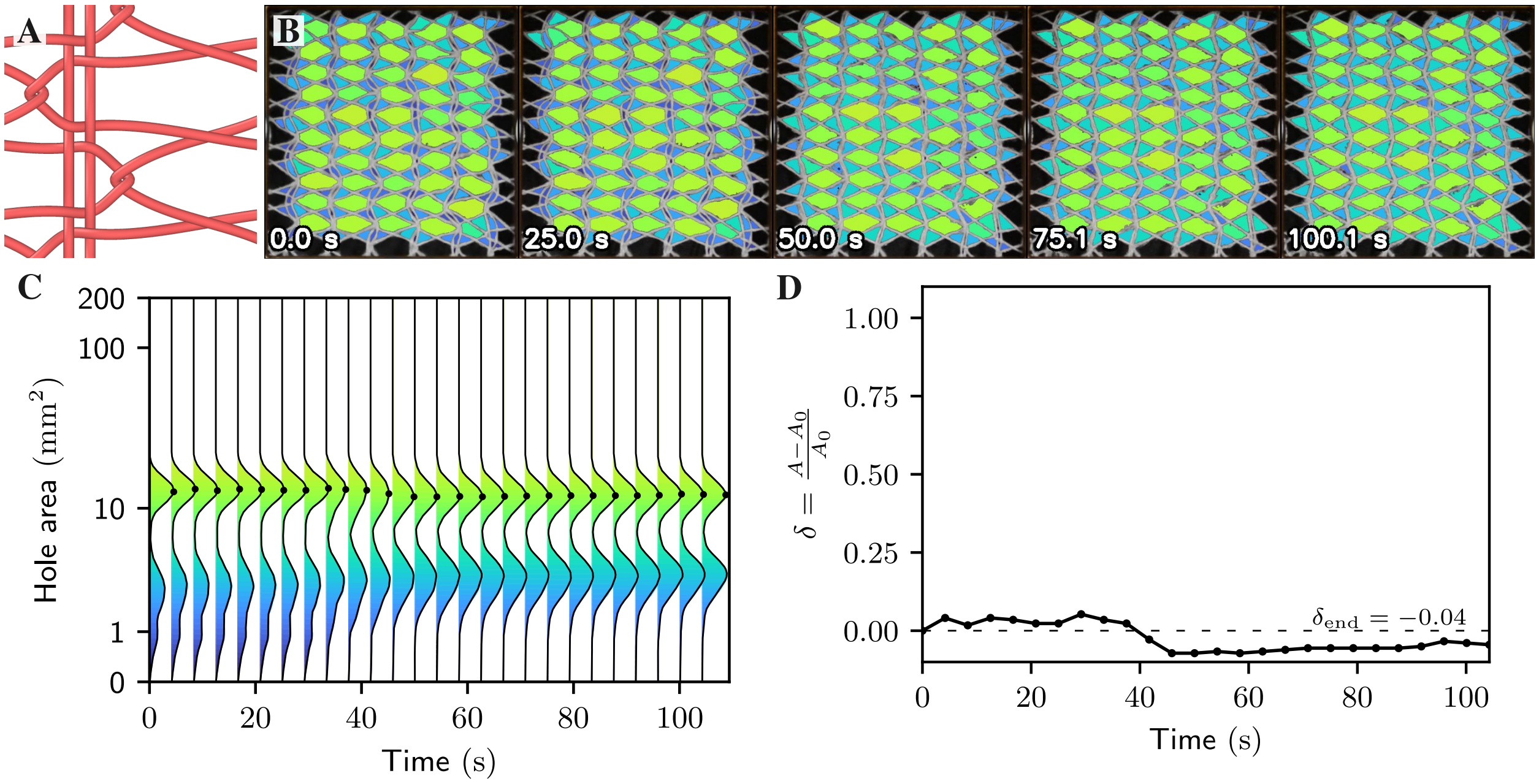

Figure S32: **Analysis of lace structure Square-2 deformation over time. A** Contraction of textile's unit-cell scheme (red yarns shrink). **B** Time-lapse visualization of the textile structure under deformation. **C** Evolution of the distribution of pore areas over time. **D** Normalized pore area change of the largest areas.

Hexa-1

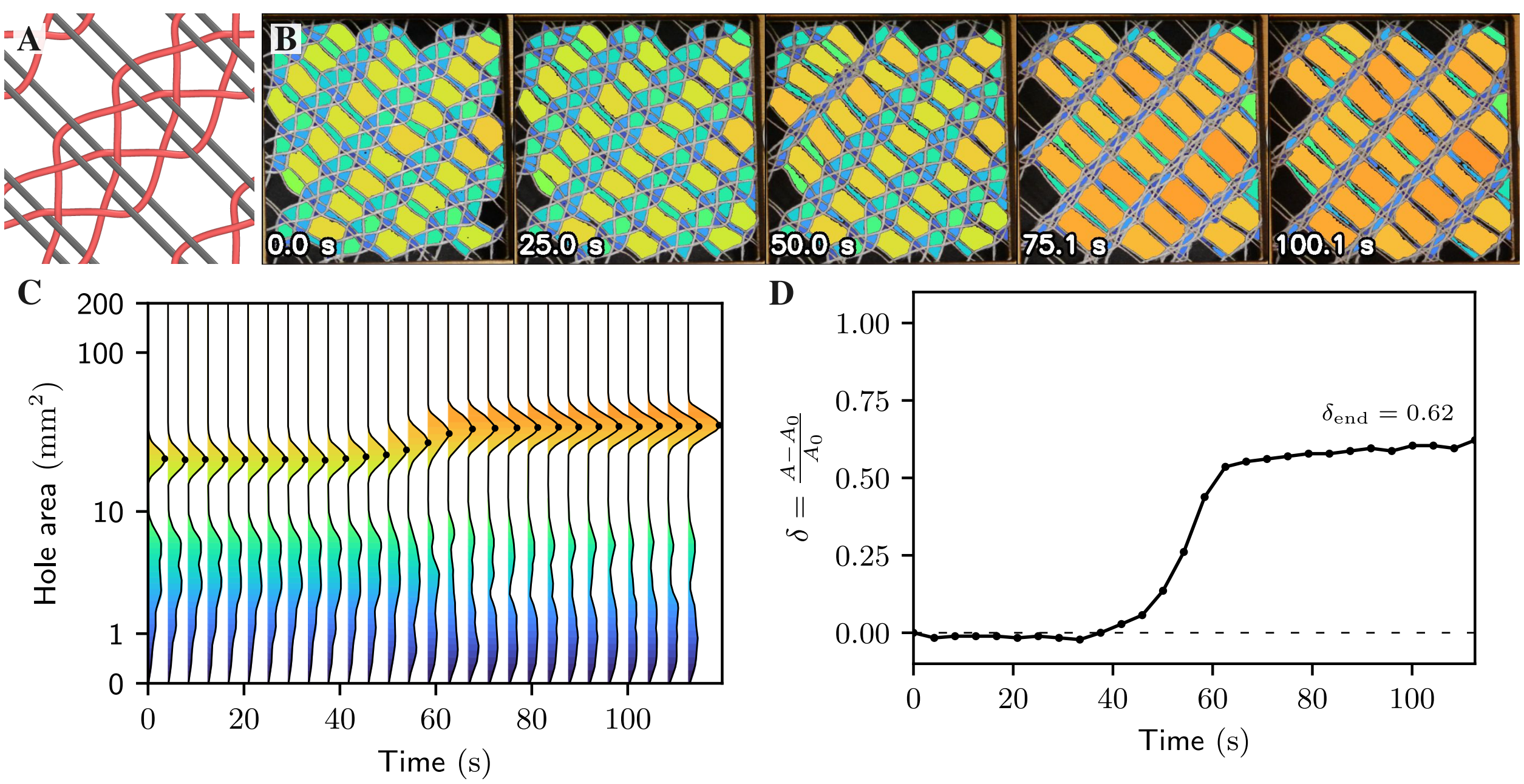

Figure S33: **Analysis of lace structure Hexa-1 deformation over time. A** Contraction of textile's unit-cell scheme (red yarns shrink). **B** Time-lapse visualization of the textile structure under deformation. **C** Evolution of the distribution of pore areas over time. **D** Normalized pore area change of the largest areas.

Rose-1

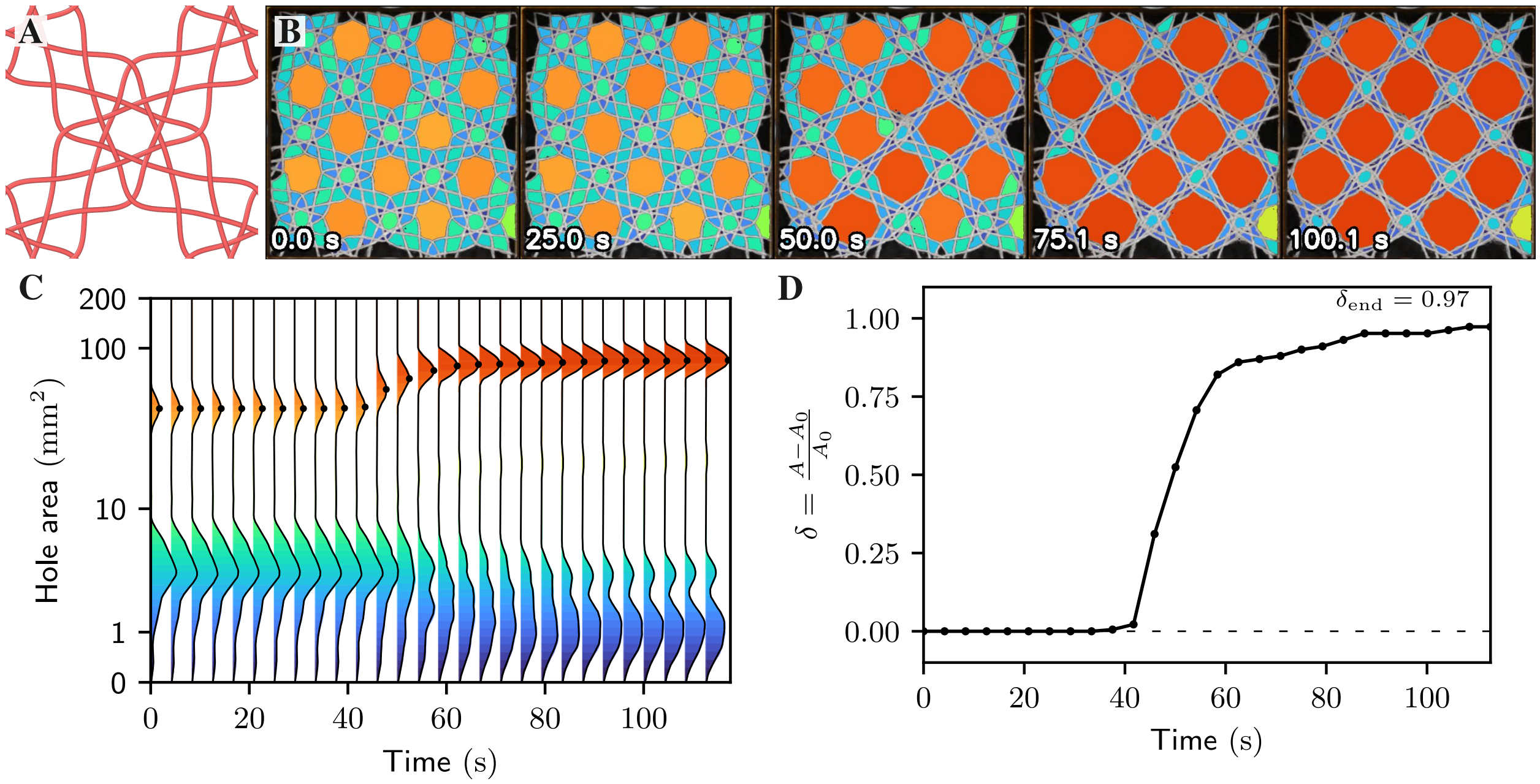

Figure S34: **Analysis of lace structure Rose-1 deformation over time. A** Contraction of textile's unit-cell scheme (red yarns shrink). **B** Time-lapse visualization of the textile structure under deformation. **C** Evolution of the distribution of pore areas over time. **D** Normalized pore area change of the largest areas.

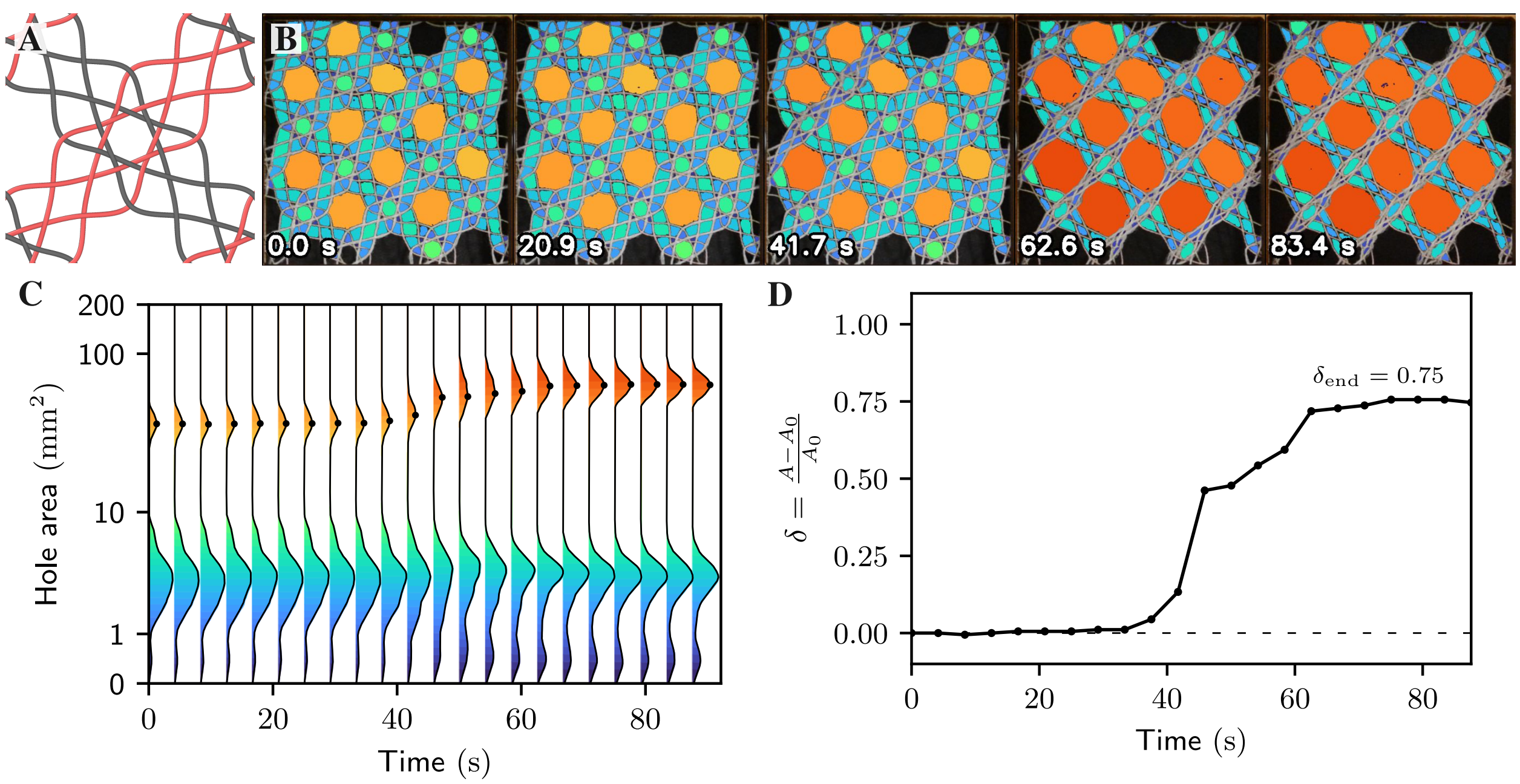

Figure S35: **Analysis of lace structure Rose-1 deformation over time. A** Contraction of textile's unit-cell scheme (red yarns shrink). **B** Time-lapse visualization of the textile structure under deformation. **C** Evolution of the distribution of pore areas over time. **D** Normalized pore area change of the largest areas.

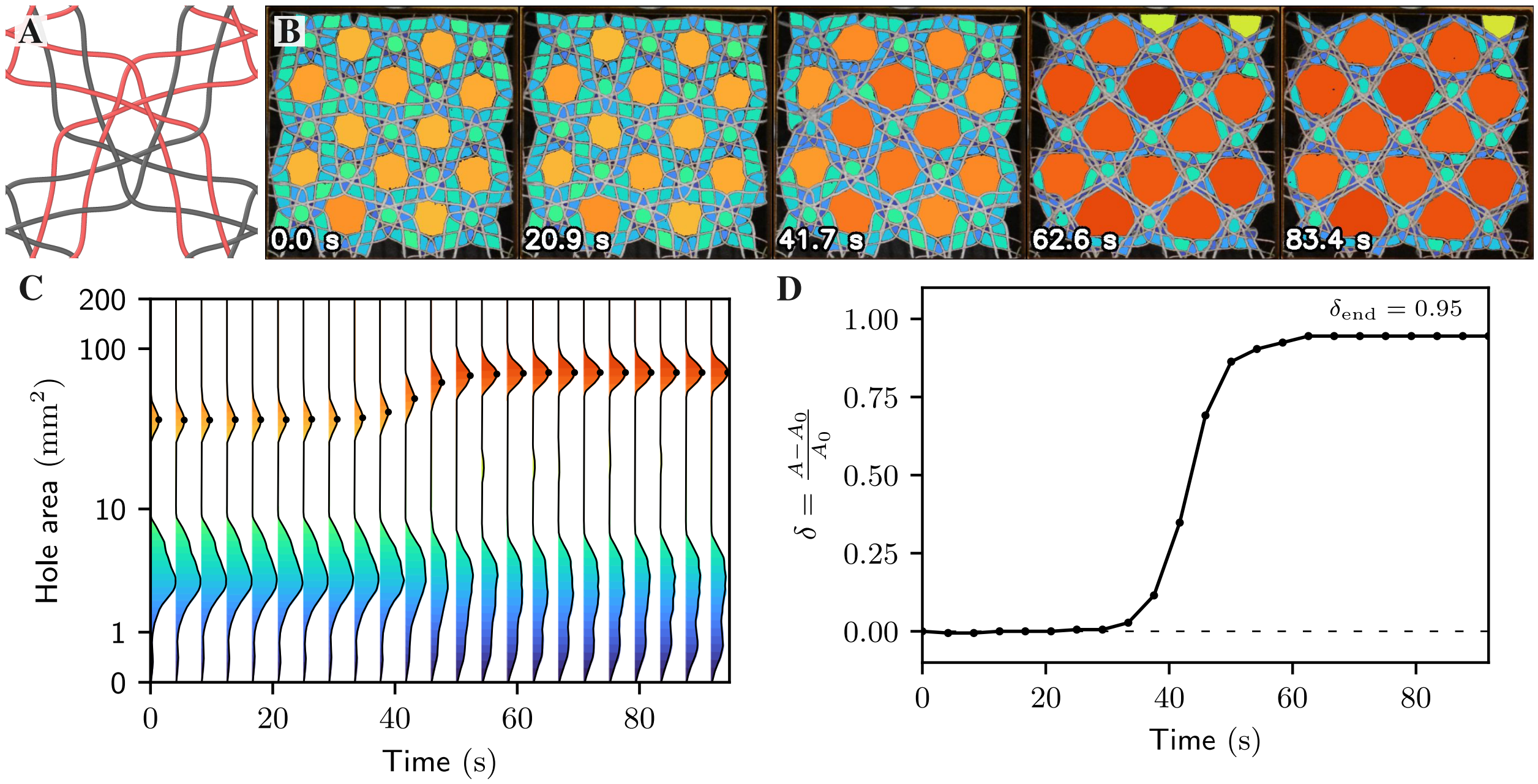

Figure S36: **Analysis of lace structure Rose-1 deformation over time. A** Contraction of textile's unit-cell scheme (red yarns shrink). **B** Time-lapse visualization of the textile structure under deformation. **C** Evolution of the distribution of pore areas over time. **D** Normalized pore area change of the largest areas.

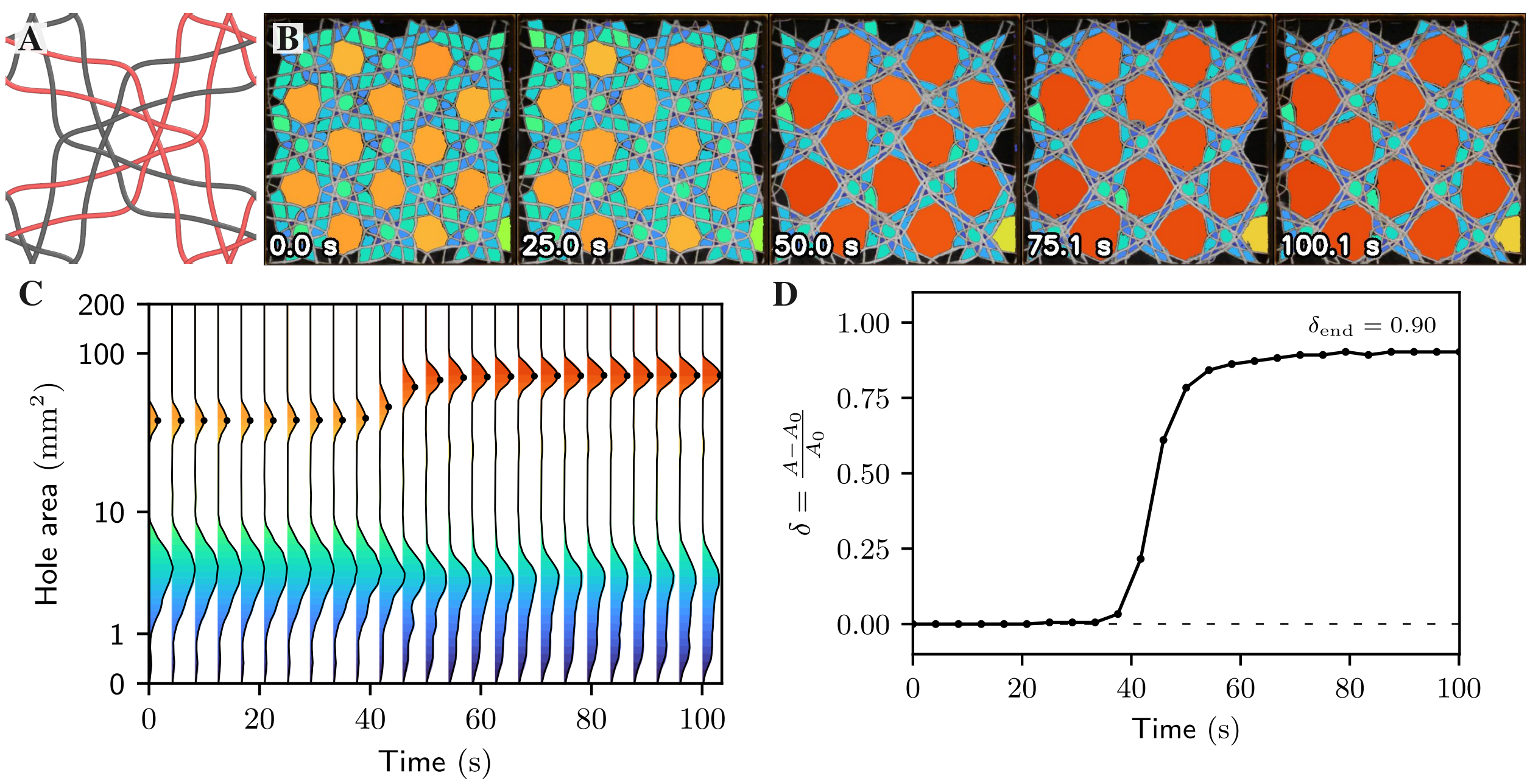

Figure S37: **Analysis of lace structure Rose-1 deformation over time. A** Contraction of textile's unit-cell scheme (red yarns shrink). **B** Time-lapse visualization of the textile structure under deformation. **C** Evolution of the distribution of pore areas over time. **D** Normalized pore area change of the largest areas.

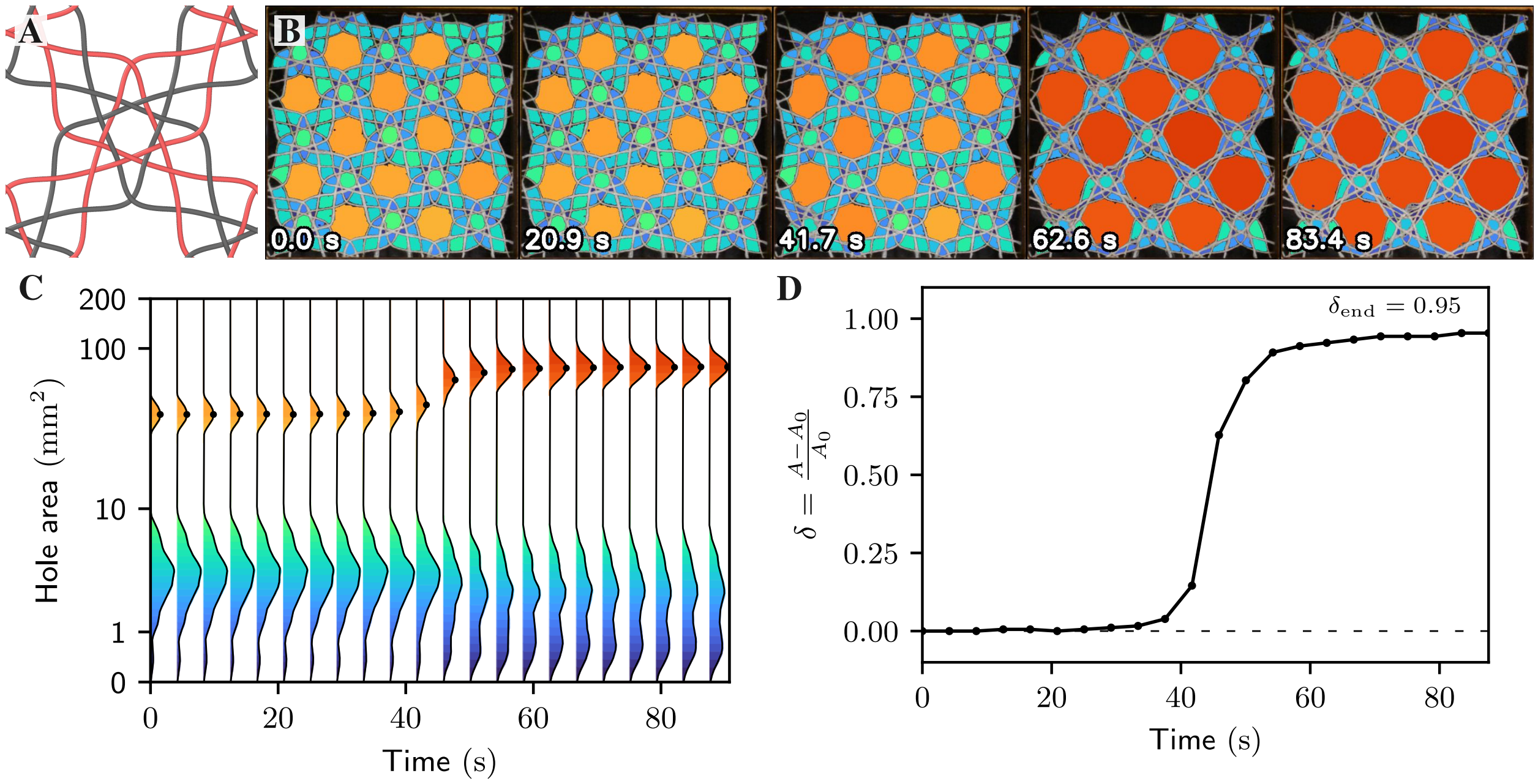

Figure S38: **Analysis of lace structure Rose-1 deformation over time. A** Contraction of textile's unit-cell scheme (red yarns shrink). **B** Time-lapse visualization of the textile structure under deformation. **C** Evolution of the distribution of pore areas over time. **D** Normalized pore area change of the largest areas.

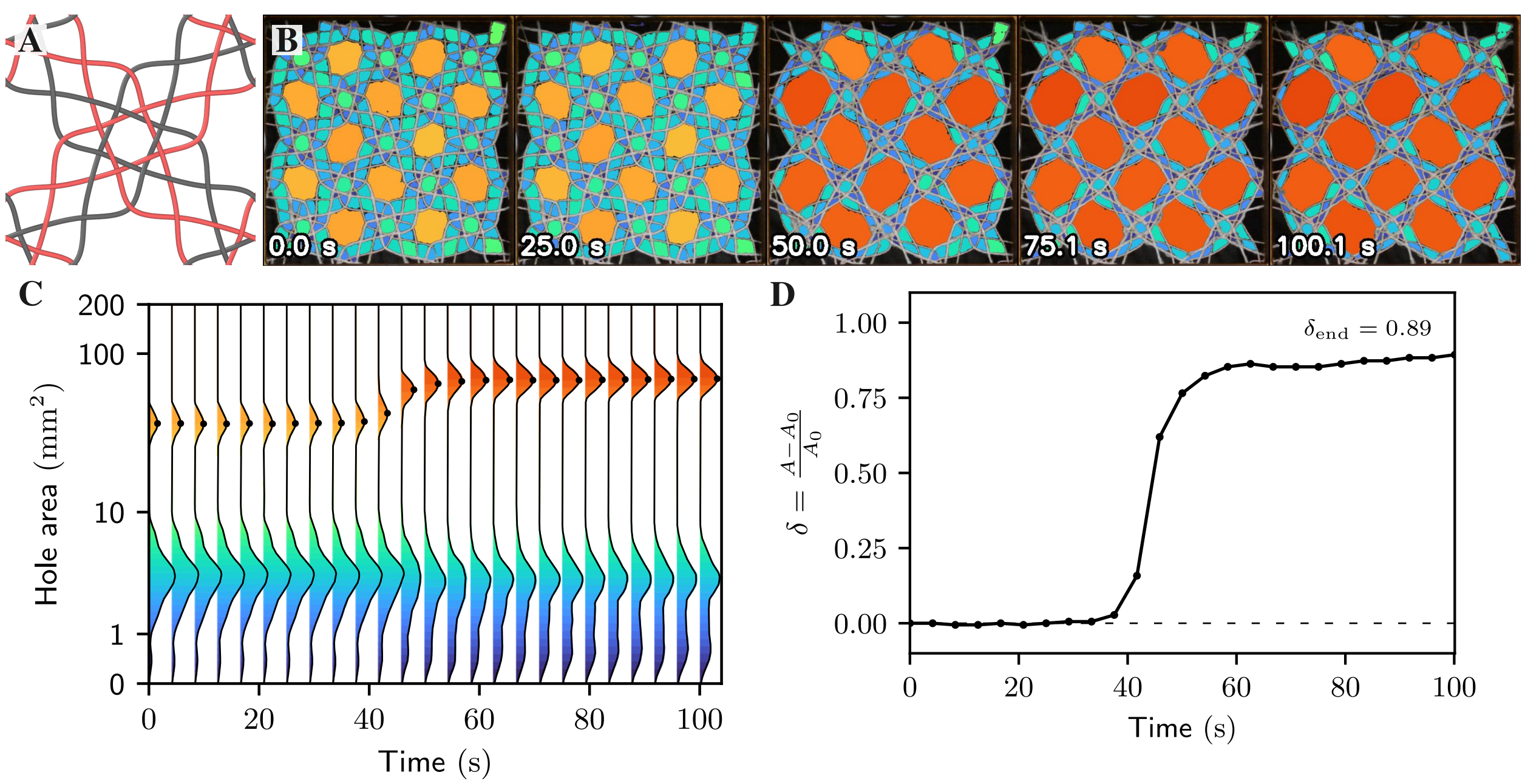

Figure S39: **Analysis of lace structure Rose-1 deformation over time. A** Contraction of textile's unit-cell scheme (red yarns shrink). **B** Time-lapse visualization of the textile structure under deformation. **C** Evolution of the distribution of pore areas over time. **D** Normalized pore area change of the largest areas.

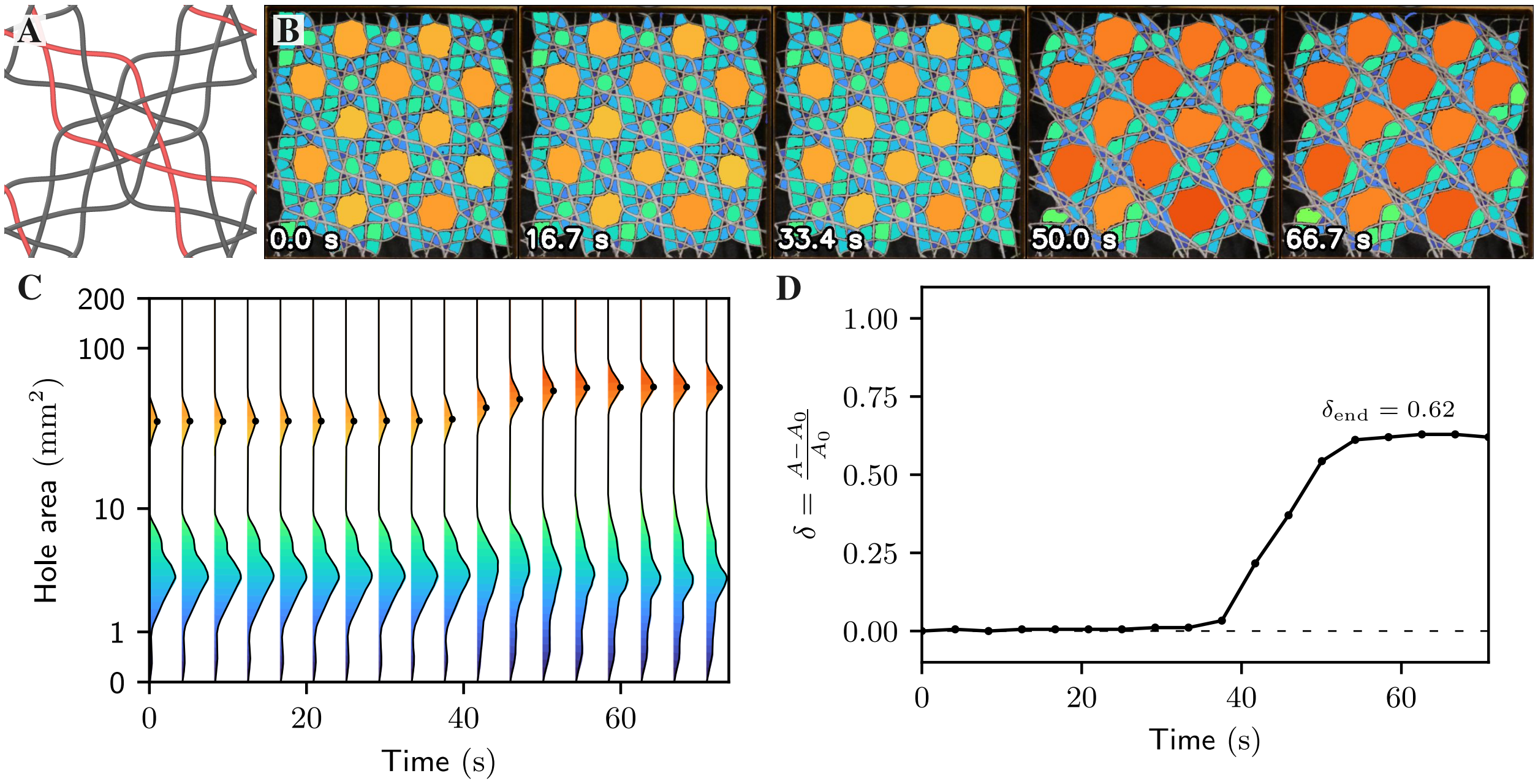

Figure S40: **Analysis of lace structure Rose-1 deformation over time. A** Contraction of textile's unit-cell scheme (red yarns shrink). **B** Time-lapse visualization of the textile structure under deformation. **C** Evolution of the distribution of pore areas over time. **D** Normalized pore area change of the largest areas.

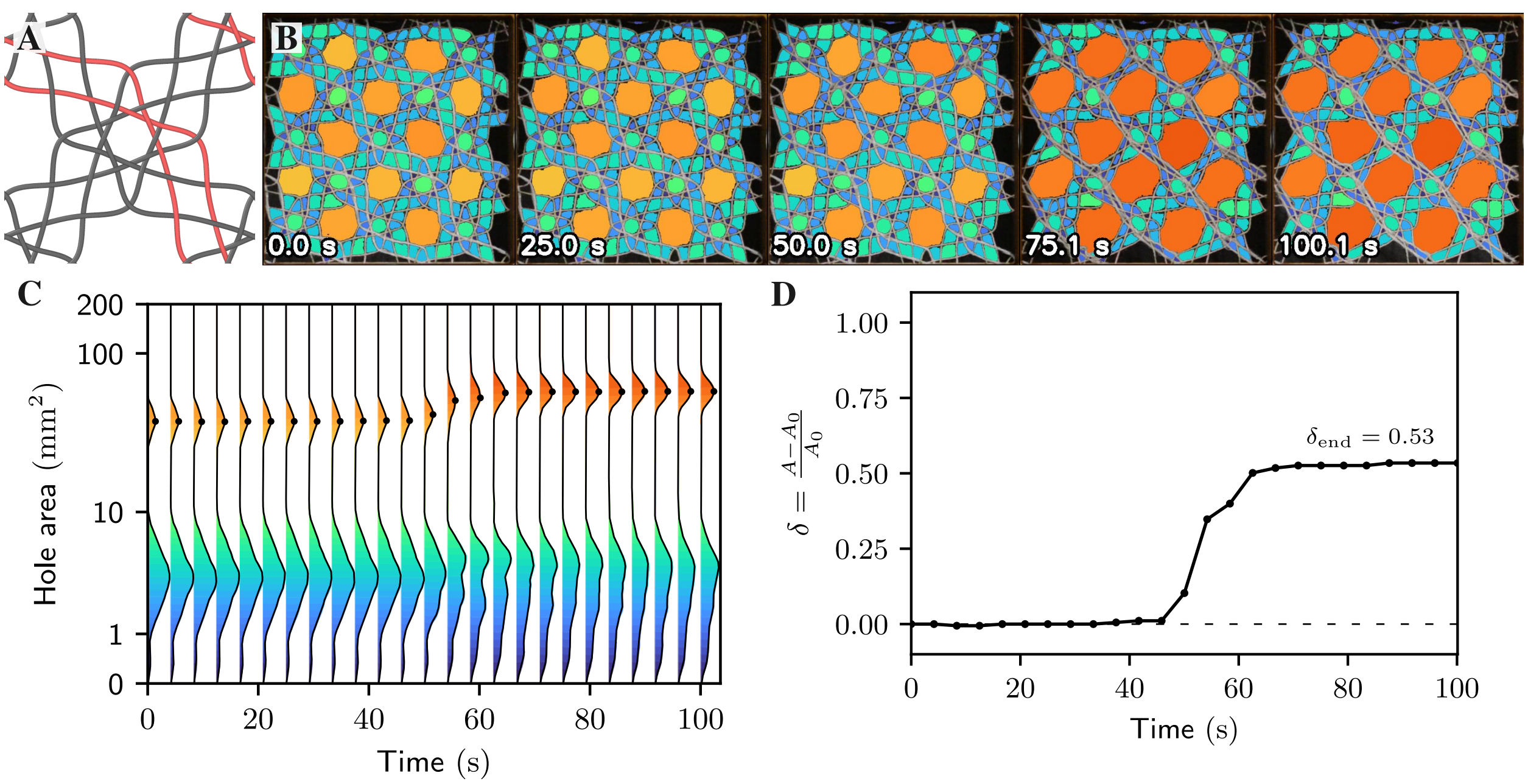

Figure S41: **Analysis of lace structure Rose-1 deformation over time. A** Contraction of textile's unit-cell scheme (red yarns shrink). **B** Time-lapse visualization of the textile structure under deformation. **C** Evolution of the distribution of pore areas over time. **D** Normalized pore area change of the largest areas.

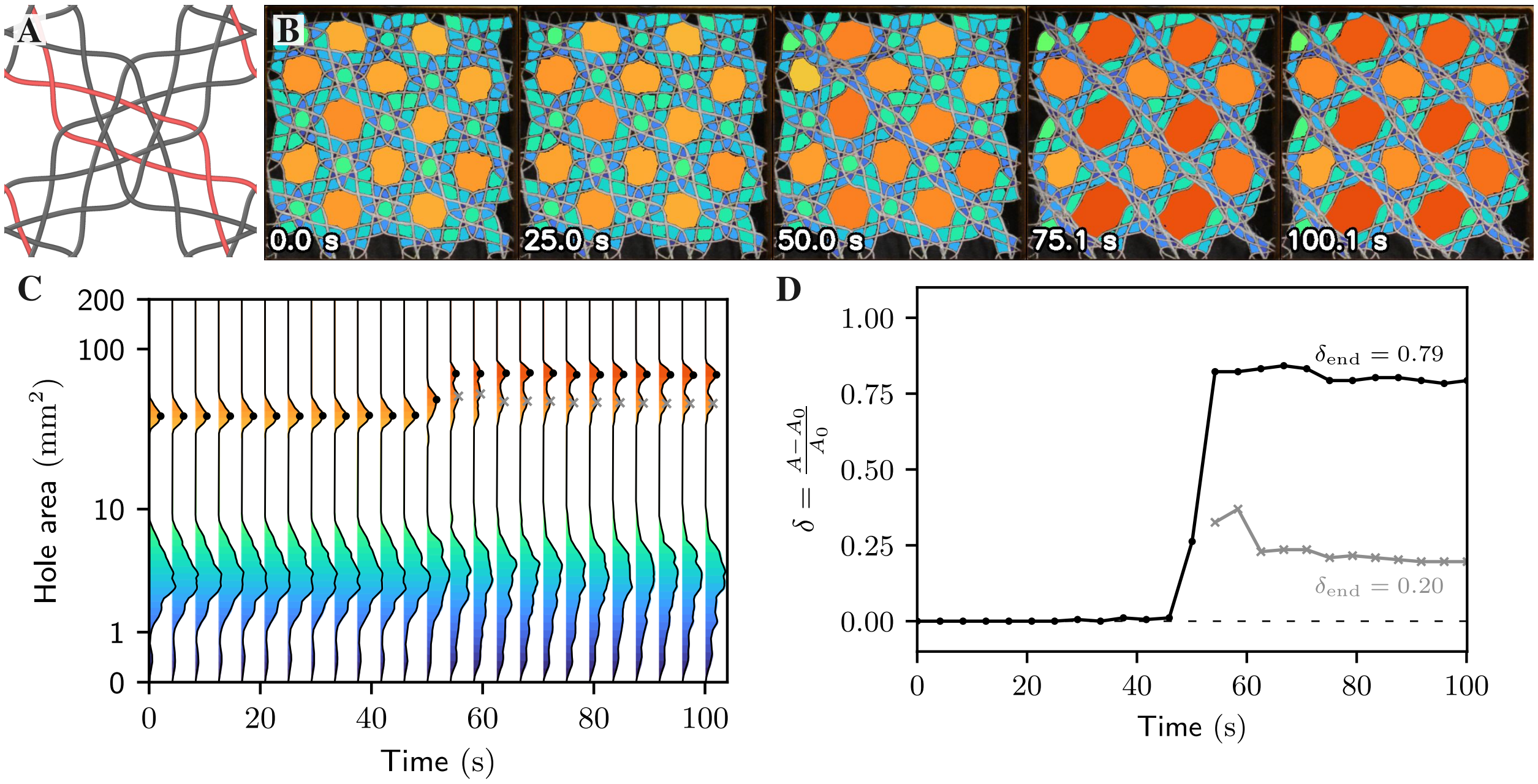

Figure S42: **Analysis of lace structure Rose-1 deformation over time. A** Contraction of textile's unit-cell scheme (red yarns shrink). **B** Time-lapse visualization of the textile structure under deformation. **C** Evolution of the distribution of pore areas over time. **D** Normalized pore area change of the largest areas.

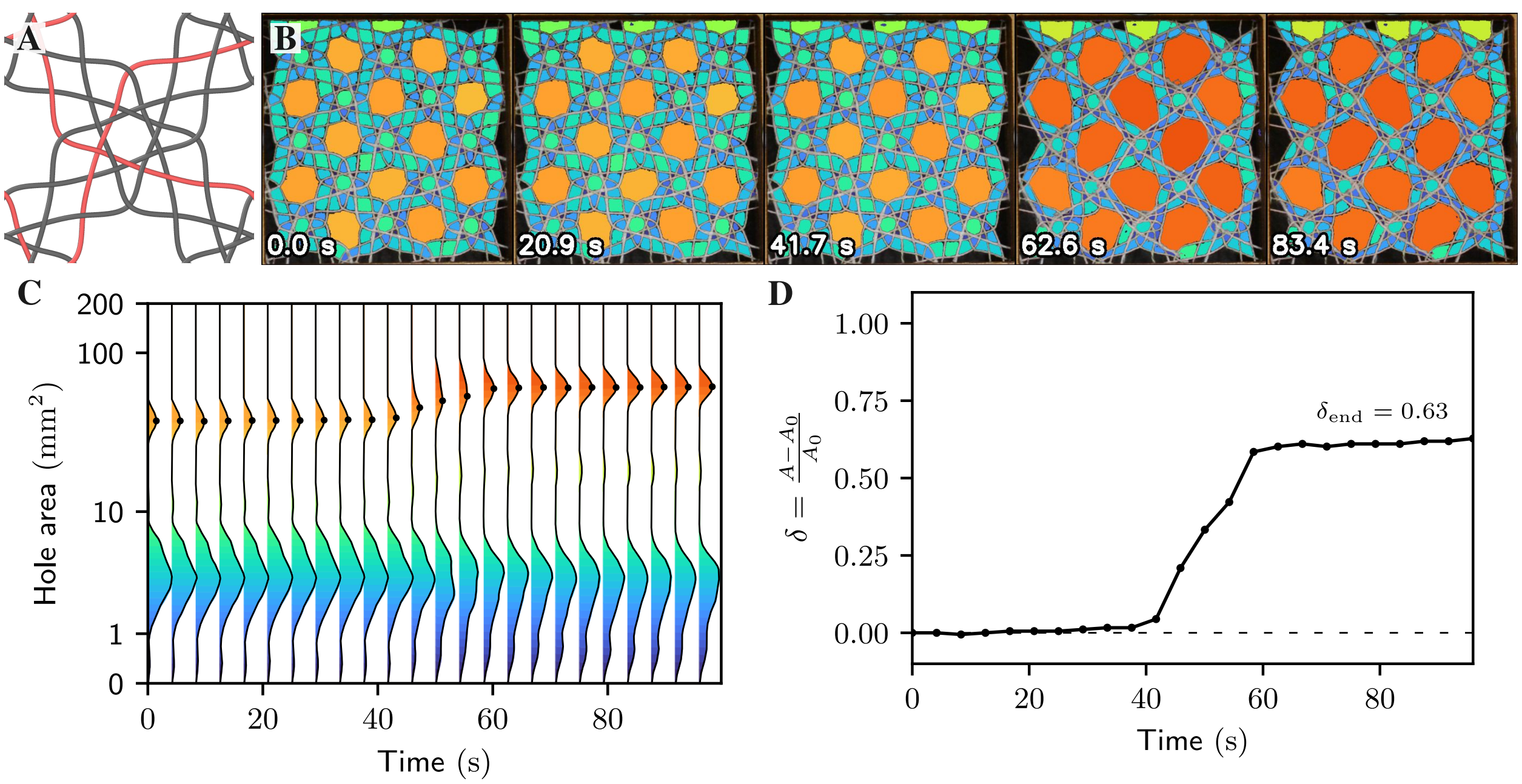

Figure S43: **Analysis of lace structure Rose-1 deformation over time. A** Contraction of textile's unit-cell scheme (red yarns shrink). **B** Time-lapse visualization of the textile structure under deformation. **C** Evolution of the distribution of pore areas over time. **D** Normalized pore area change of the largest areas.

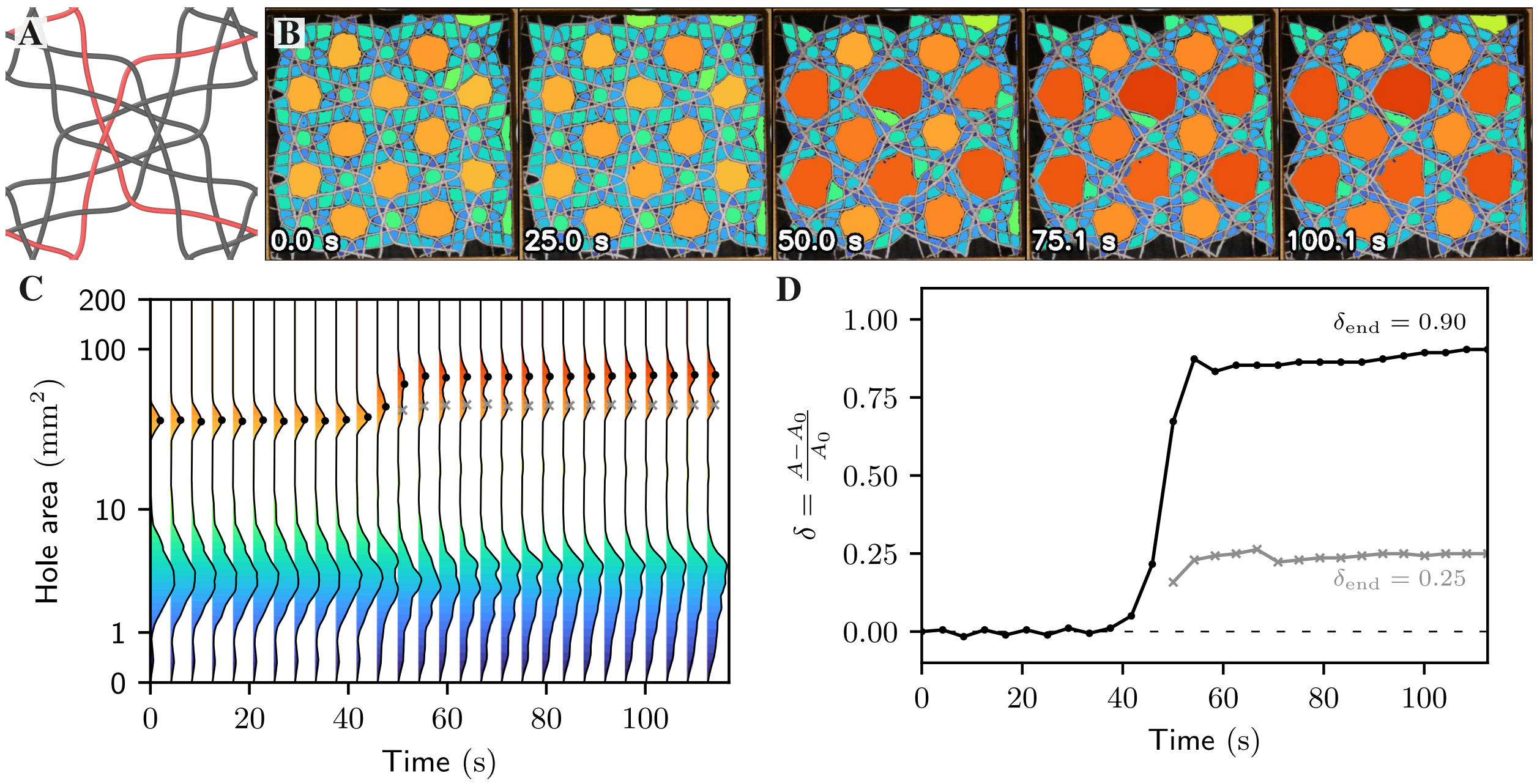

Figure S44: **Analysis of lace structure Rose-1 deformation over time. A** Contraction of textile's unit-cell scheme (red yarns shrink). **B** Time-lapse visualization of the textile structure under deformation. **C** Evolution of the distribution of pore areas over time. **D** Normalized pore area change of the largest areas.

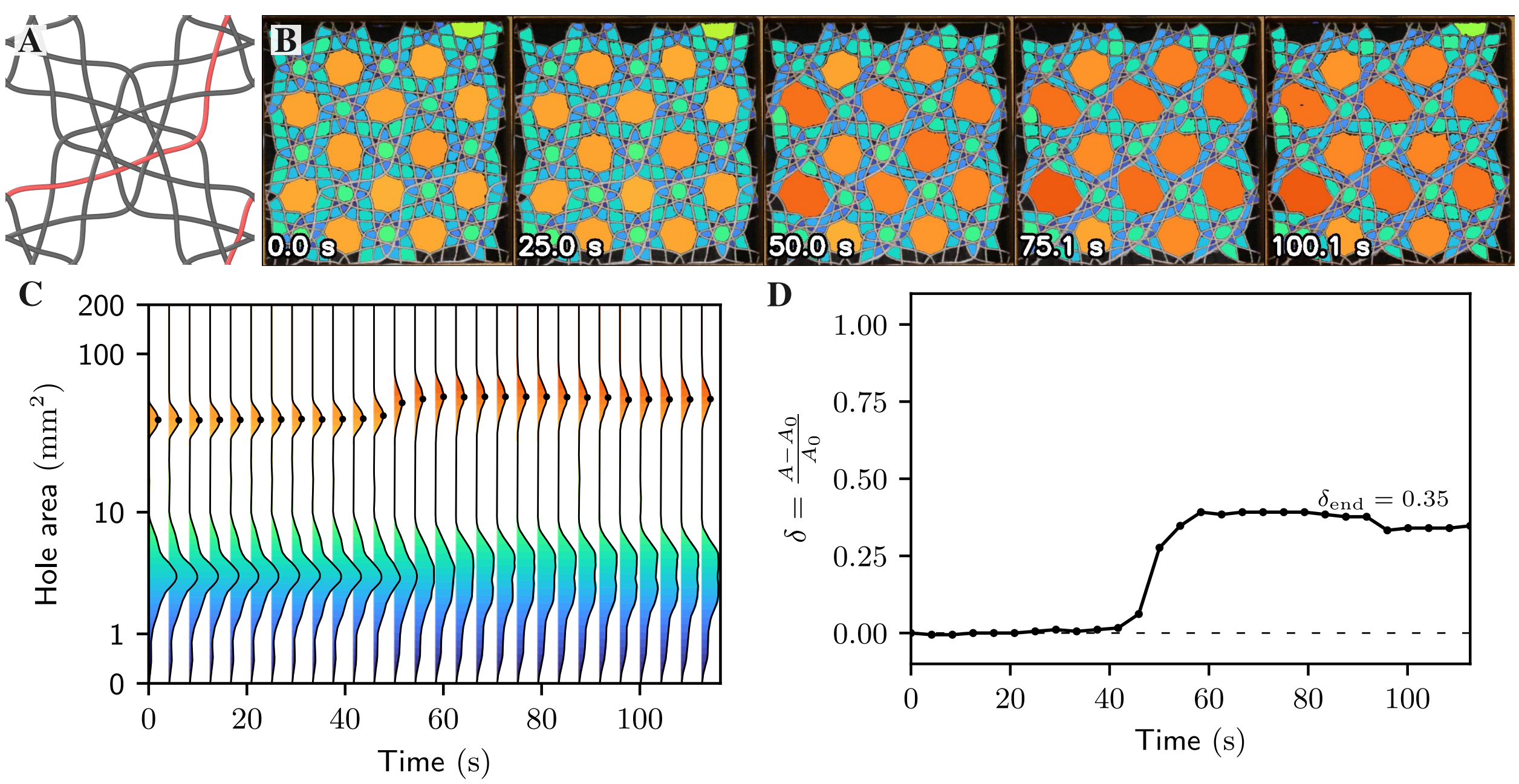

Figure S45: **Analysis of lace structure Rose-1 deformation over time. A** Contraction of textile's unit-cell scheme (red yarns shrink). **B** Time-lapse visualization of the textile structure under deformation. **C** Evolution of the distribution of pore areas over time. **D** Normalized pore area change of the largest areas.

Rose-2

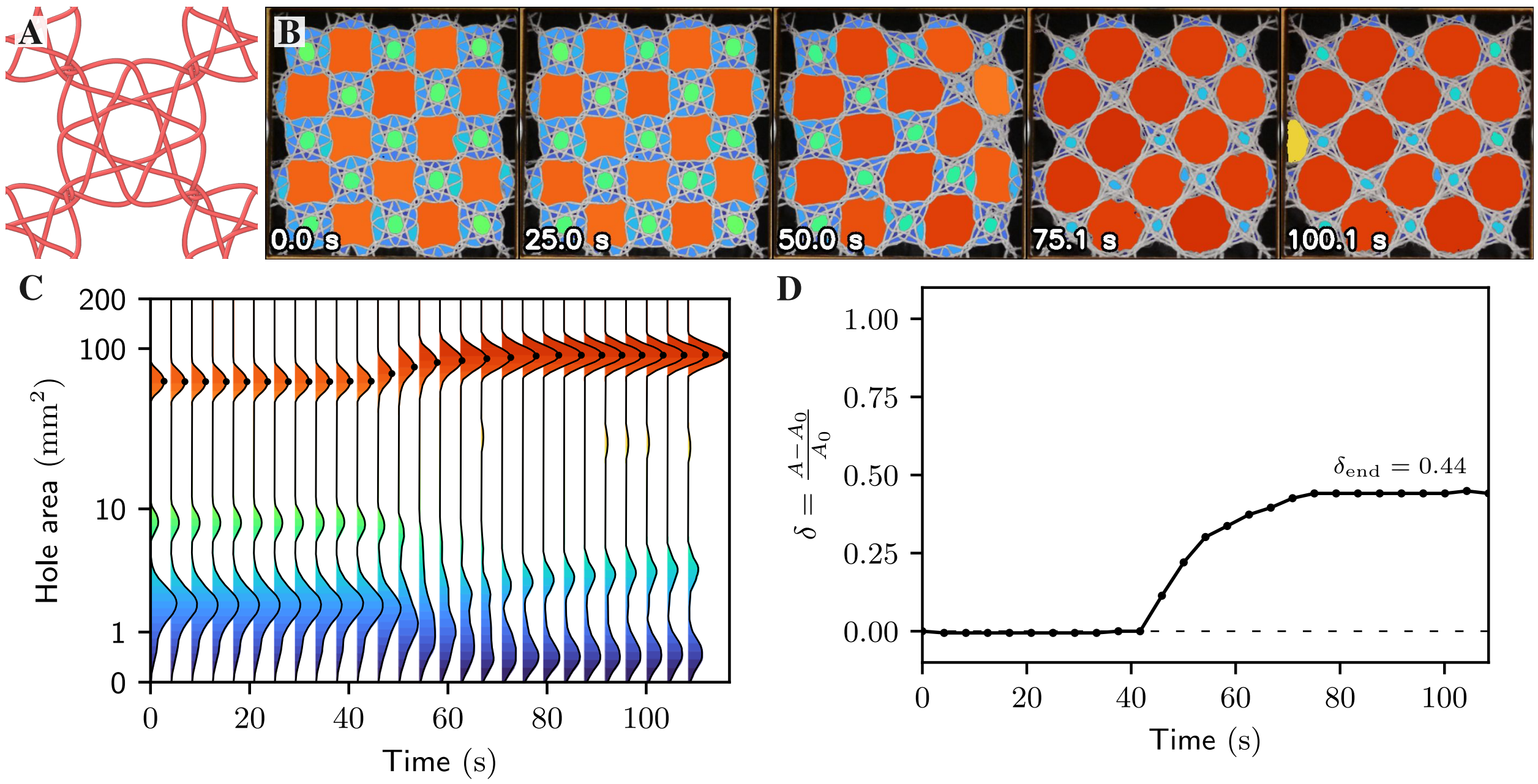

Figure S46: **Analysis of lace structure Rose-2 deformation over time. A** Contraction of textile's unit-cell scheme (red yarns shrink). **B** Time-lapse visualization of the textile structure under deformation. **C** Evolution of the distribution of pore areas over time. **D** Normalized pore area change of the largest areas.

Rose-3

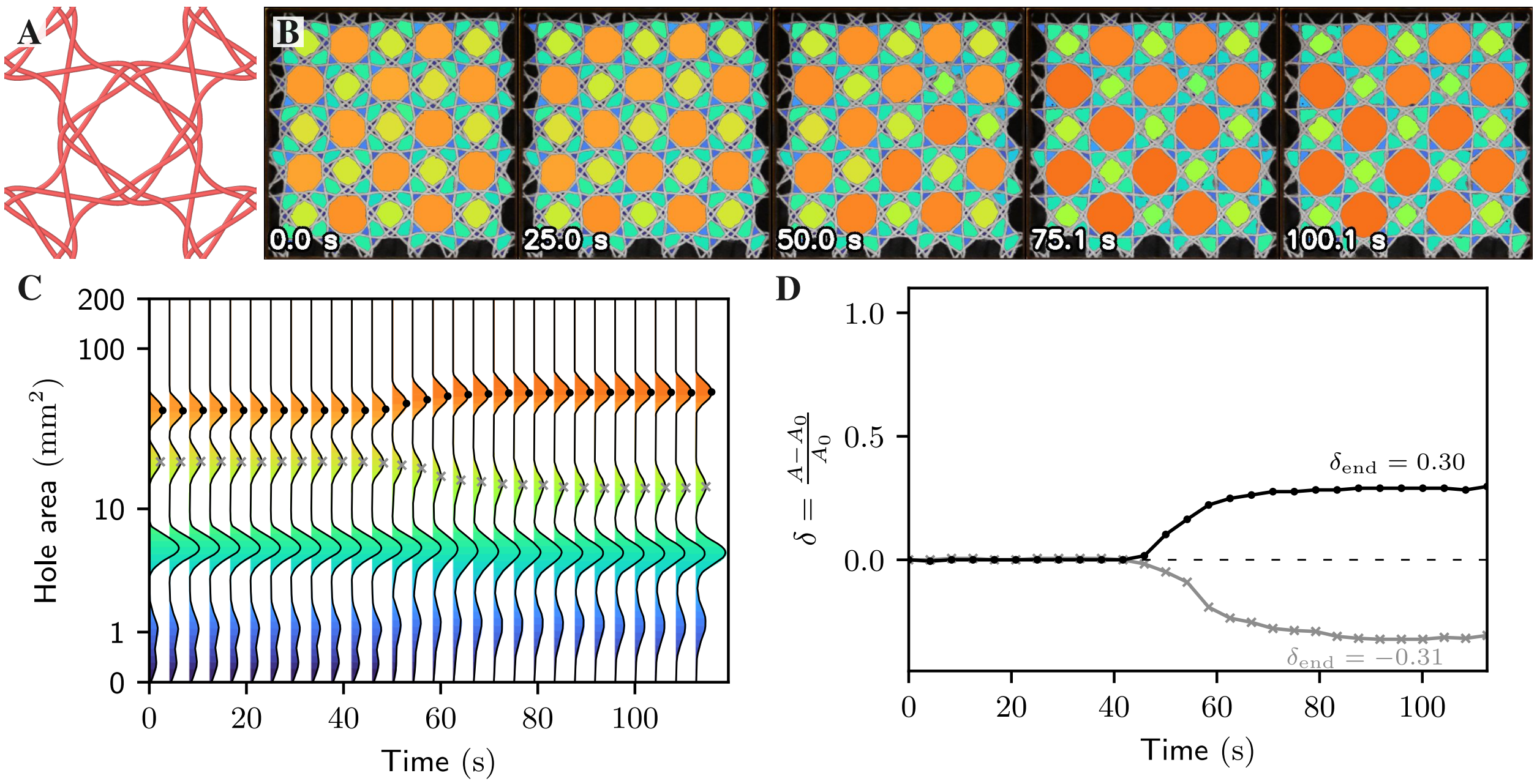

Figure S47: **Analysis of lace structure Rose-3 deformation over time. A** Contraction of textile's unit-cell scheme (red yarns shrink). **B** Time-lapse visualization of the textile structure under deformation. **C** Evolution of the distribution of pore areas over time. **D** Normalized pore area change of the largest areas.

Rose-4

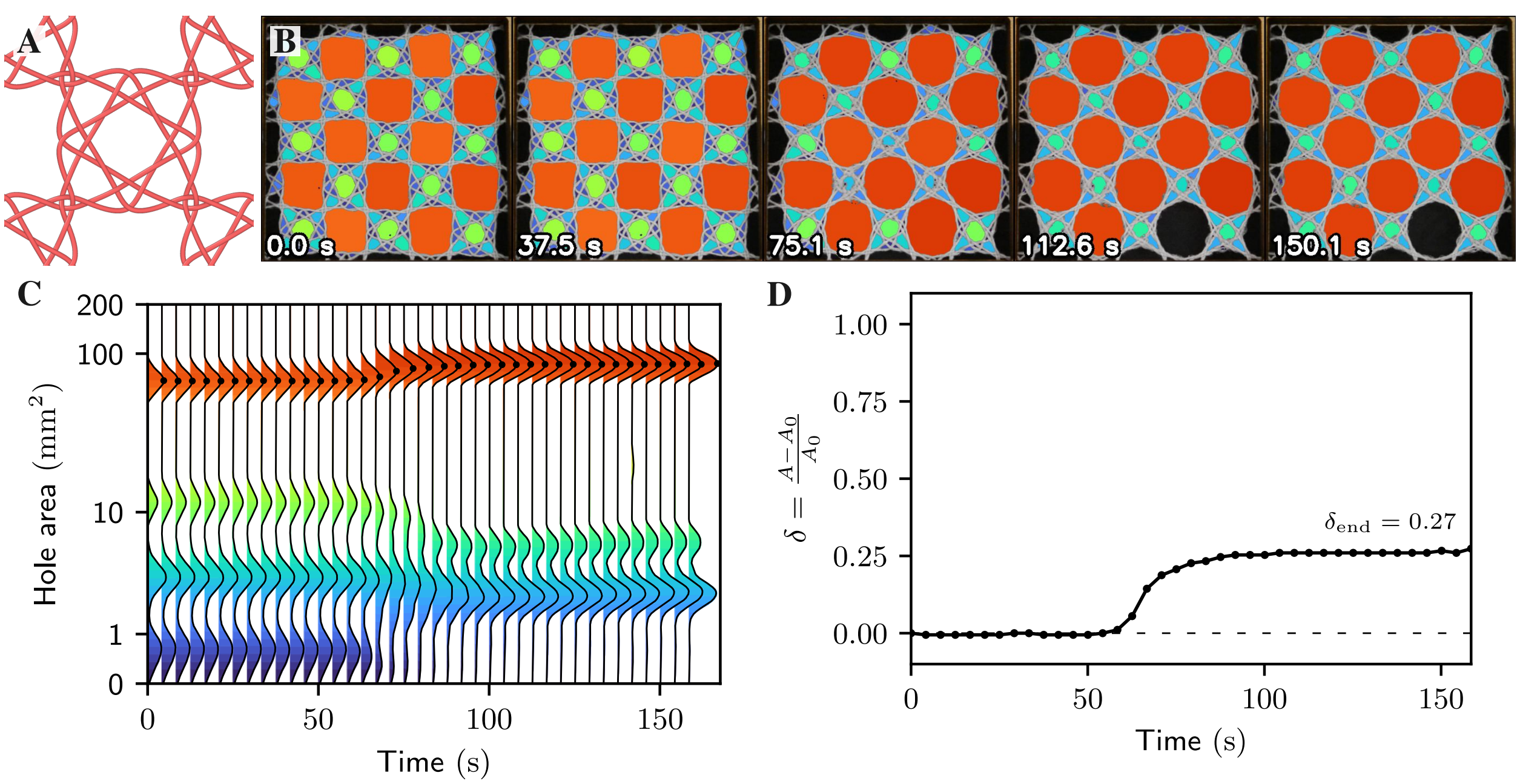

Figure S48: **Analysis of textile structure Rose-4 deformation over time. A** Contraction of textile's unit-cell scheme (red yarns shrink). **B** Time-lapse visualization of the textile structure under deformation. **C** Evolution of the distribution of pore areas over time. **D** Normalized pore area change of the largest areas.

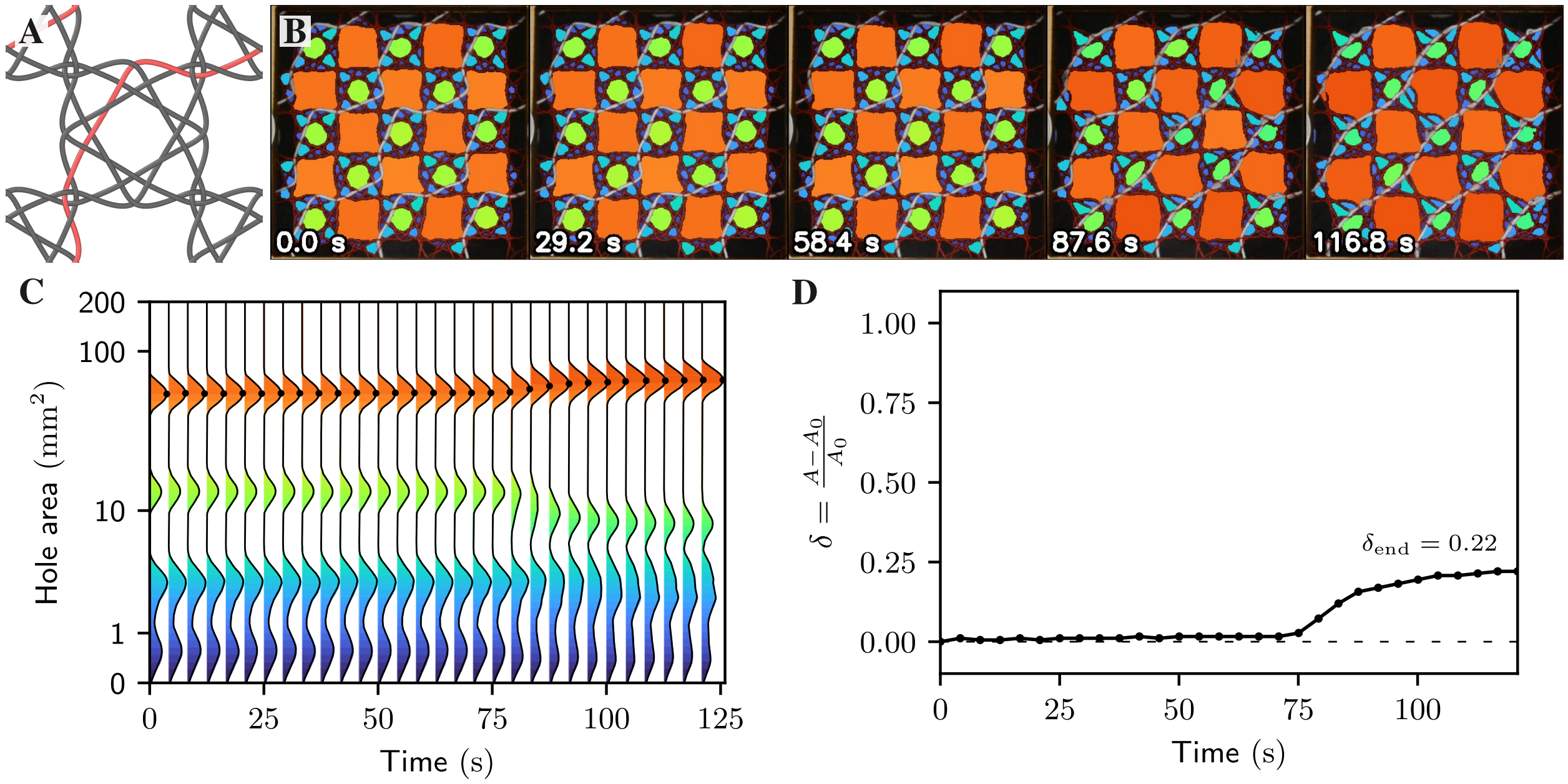

Figure S49: **Analysis of textile structure Rose-4 deformation over time. A** Contraction of textile's unit-cell scheme (red yarns shrink). **B** Time-lapse visualization of the textile structure under deformation. **C** Evolution of the distribution of pore areas over time. **D** Normalized pore area change of the largest areas.

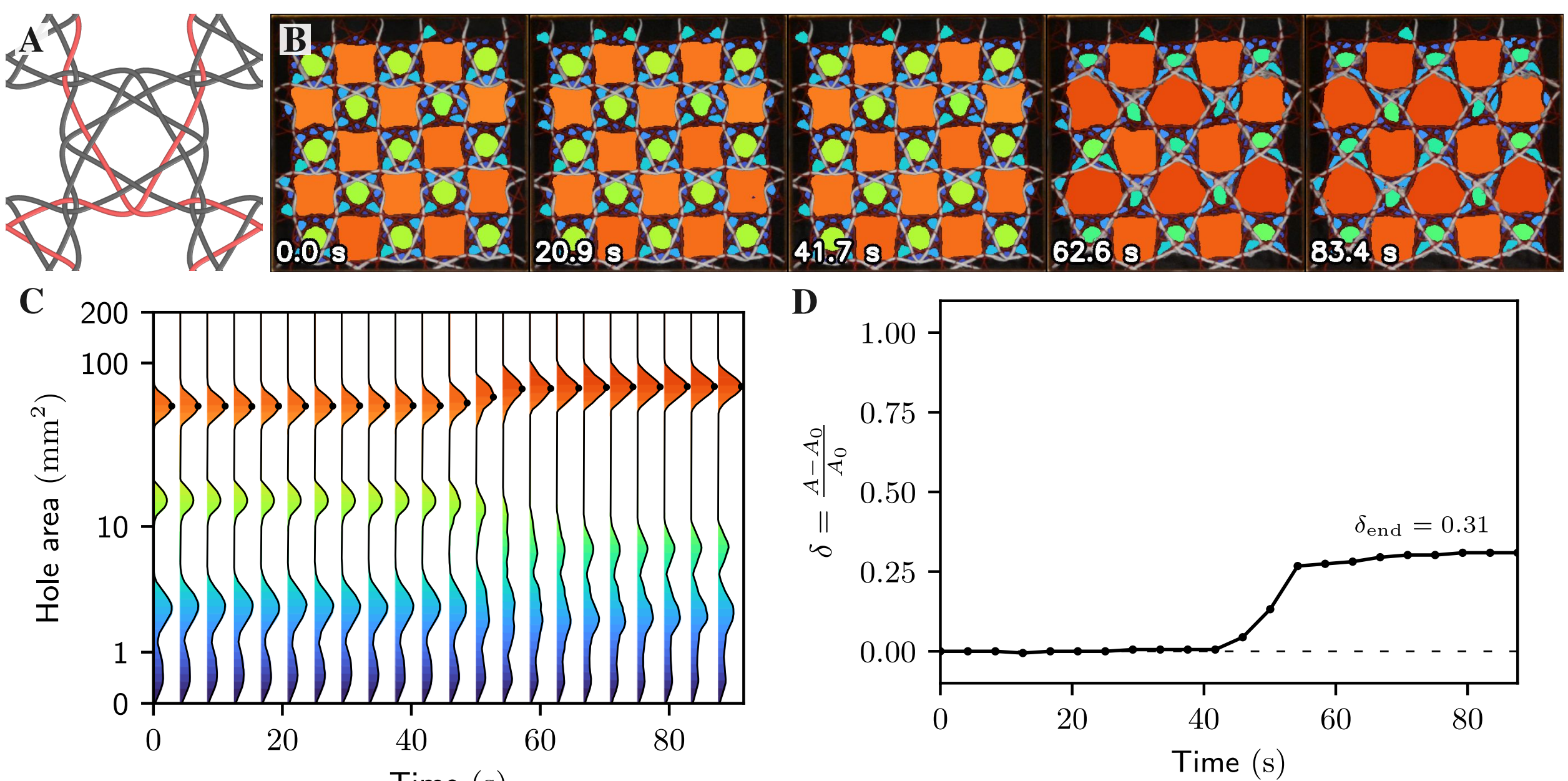

Figure S50: **Analysis of textile structure Rose-4 deformation over time. A** Contraction of textile's unit-cell scheme (red yarns shrink). **B** Time-lapse visualization of the textile structure under deformation. **C** Evolution of the distribution of pore areas over time. **D** Normalized pore area change of the largest areas.

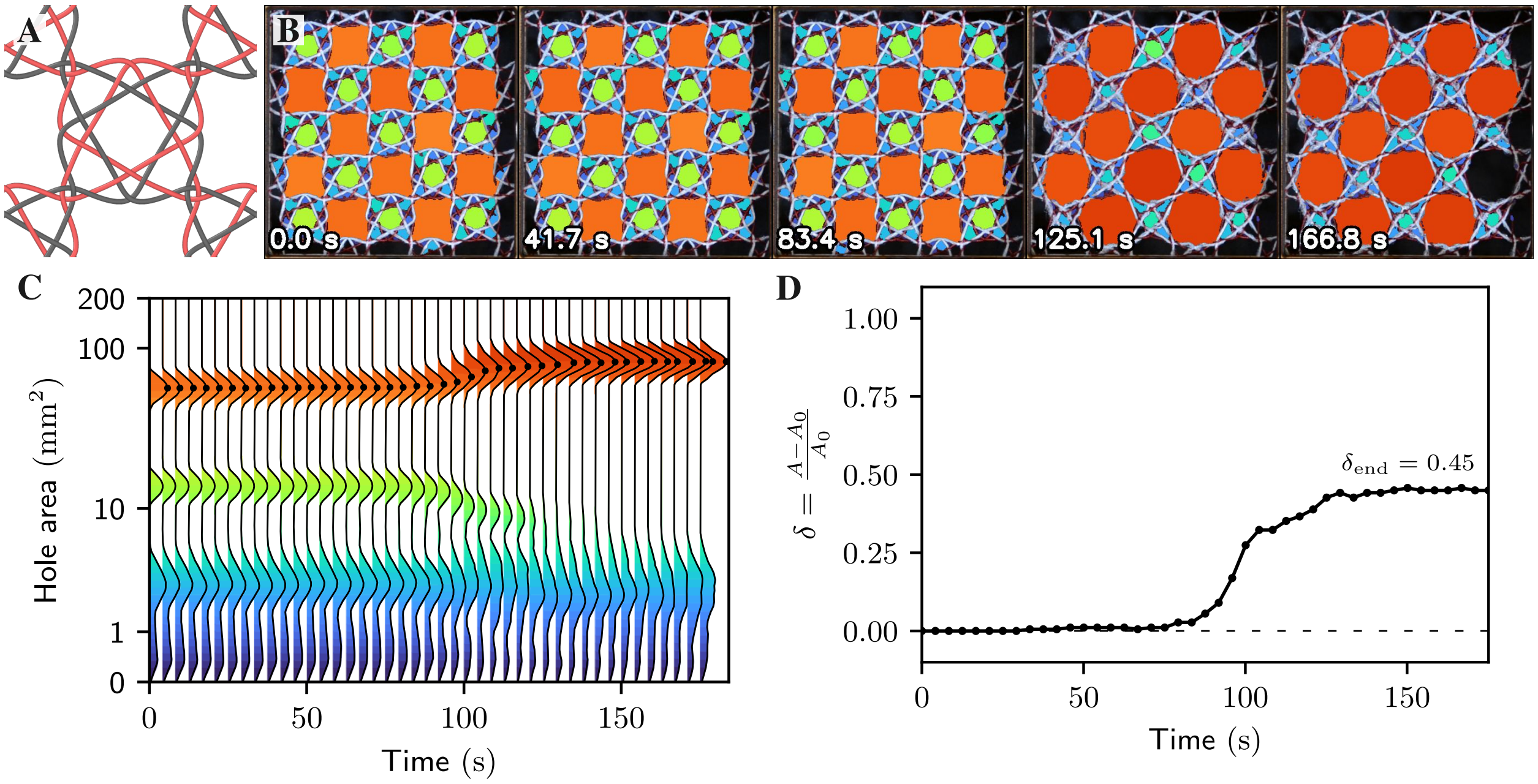

Figure S51: **Analysis of textile structure Rose-4 deformation over time. A** Contraction of textile's unit-cell scheme (red yarns shrink). **B** Time-lapse visualization of the textile structure under deformation. **C** Evolution of the distribution of pore areas over time. **D** Normalized pore area change of the largest areas.

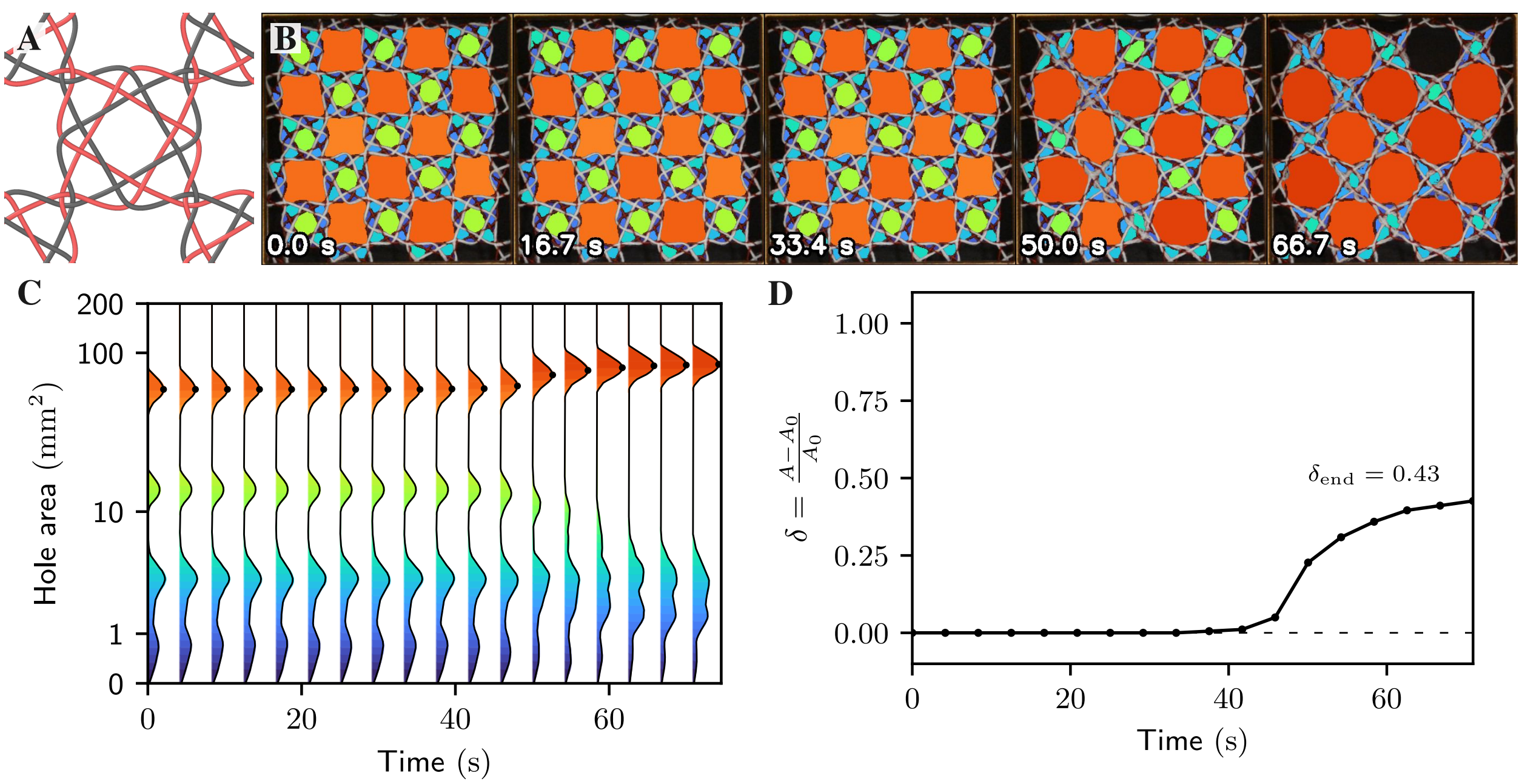

Figure S52: **Analysis of textile structure Rose-4 deformation over time. A** Contraction of textile's unit-cell scheme (red yarns shrink). **B** Time-lapse visualization of the textile structure under deformation. **C** Evolution of the distribution of pore areas over time. **D** Normalized pore area change of the largest areas.

Rose-5

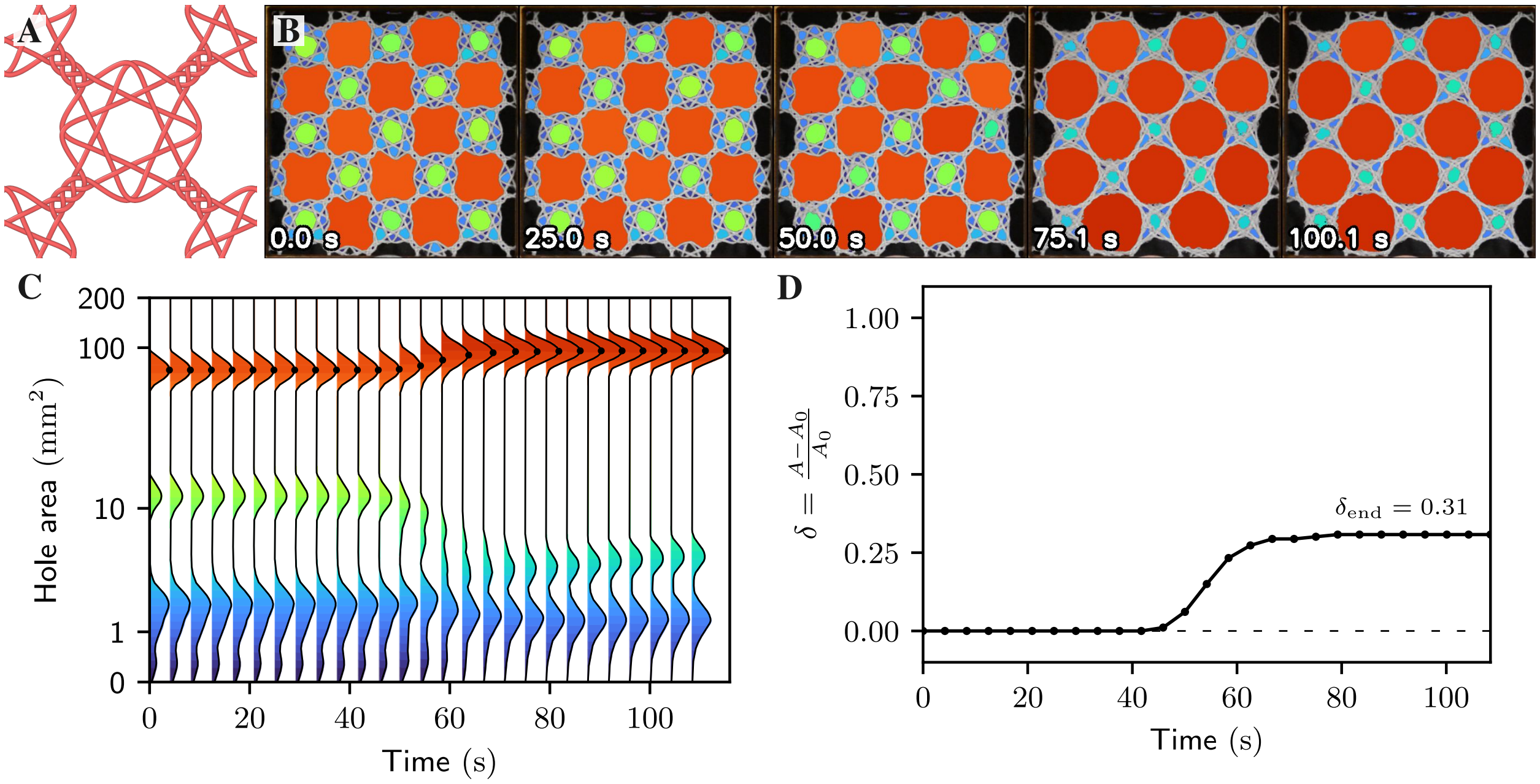

Figure S53: **Analysis of lace structure Rose-5 deformation over time. A** Contraction of textile's unit-cell scheme (red yarns shrink). **B** Time-lapse visualization of the textile structure under deformation. **C** Evolution of the distribution of pore areas over time. **D** Normalized pore area change of the largest areas.

Rose-6

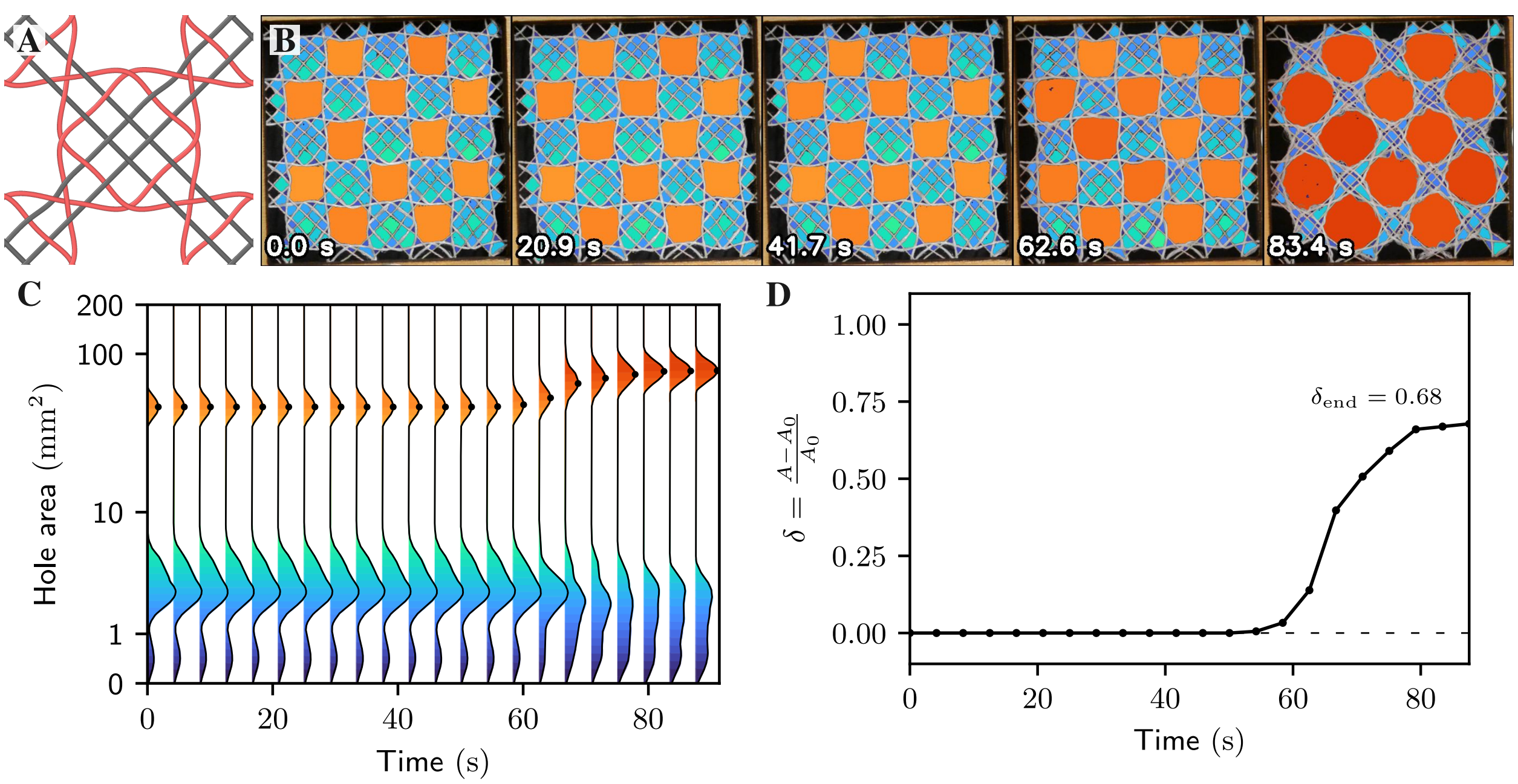

Figure S54: **Analysis of lace structure Rose-6 deformation over time. A** Contraction of textile's unit-cell scheme (red yarns shrink). **B** Time-lapse visualization of the textile structure under deformation. **C** Evolution of the distribution of pore areas over time. **D** Normalized pore area change of the largest areas.

Rose-7

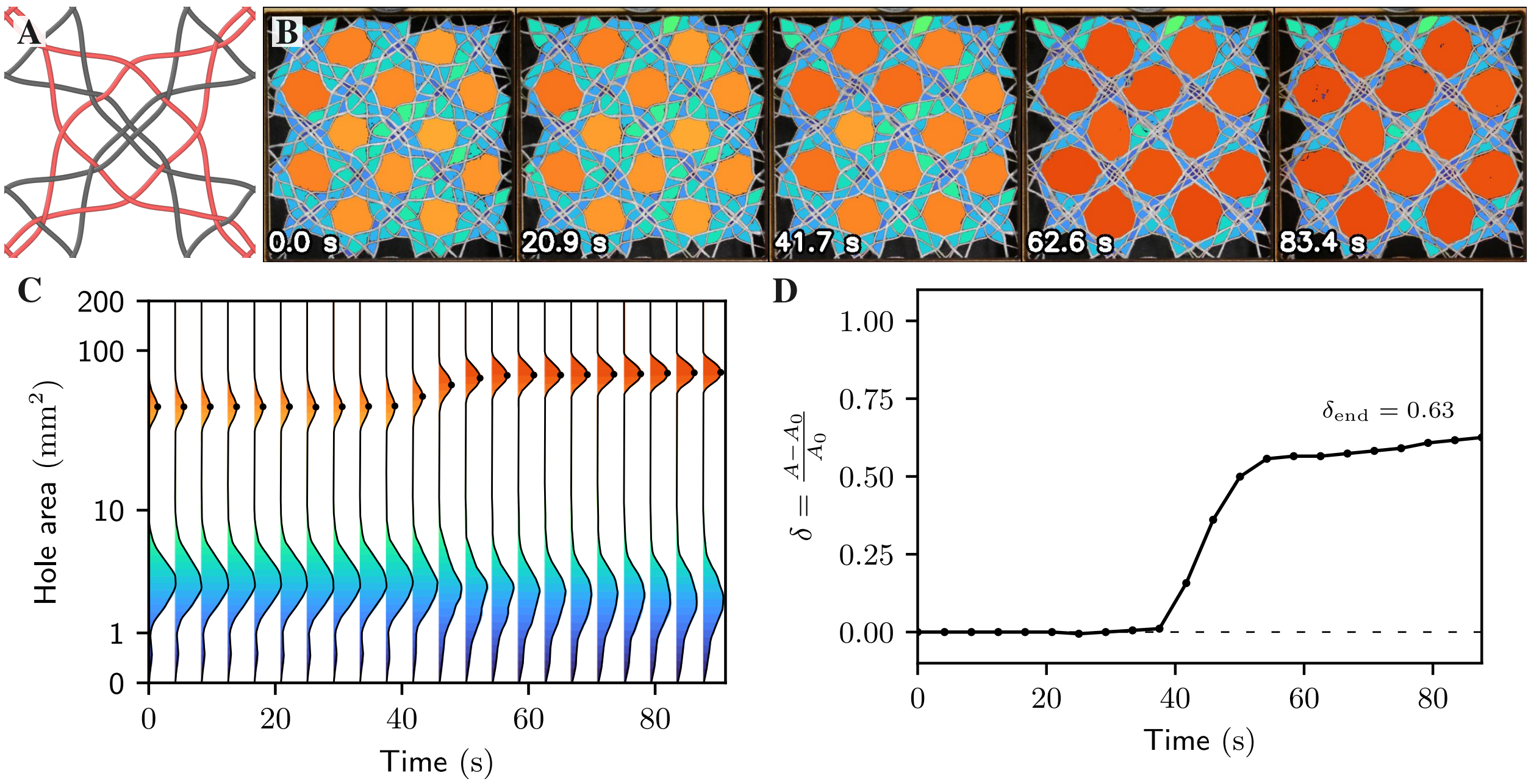

Figure S55: **Analysis of lace structure Rose-7 deformation over time. A** Contraction of textile's unit-cell scheme (red yarns shrink). **B** Time-lapse visualization of the textile structure under deformation. **C** Evolution of the distribution of pore areas over time. **D** Normalized pore area change of the largest areas.

Rose-8

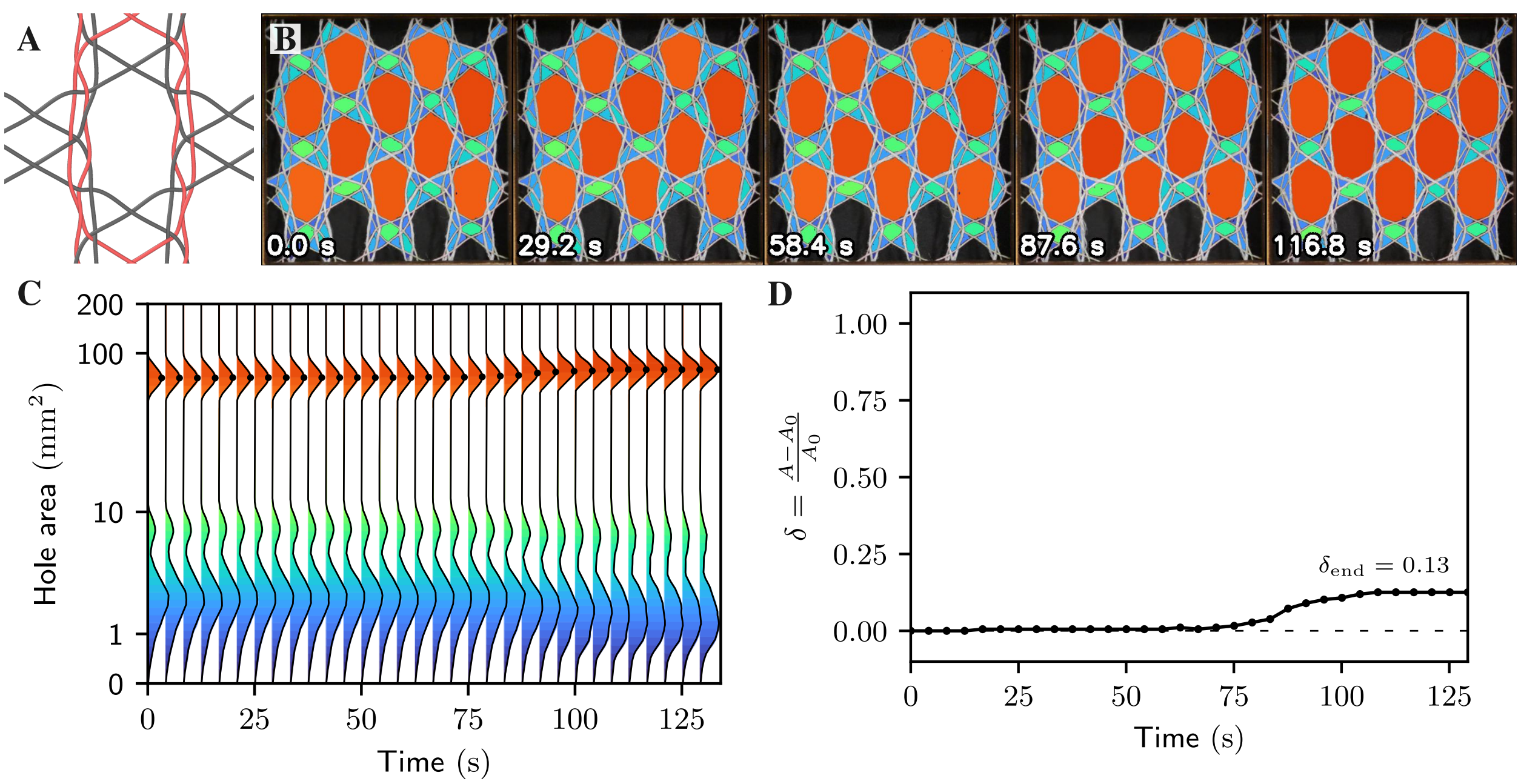

Figure S56: **Analysis of lace structure Rose-8 deformation over time. A** Contraction of textile's unit-cell scheme (red yarns shrink). **B** Time-lapse visualization of the textile structure under deformation. **C** Evolution of the distribution of pore areas over time. **D** Normalized pore area change of the largest areas.

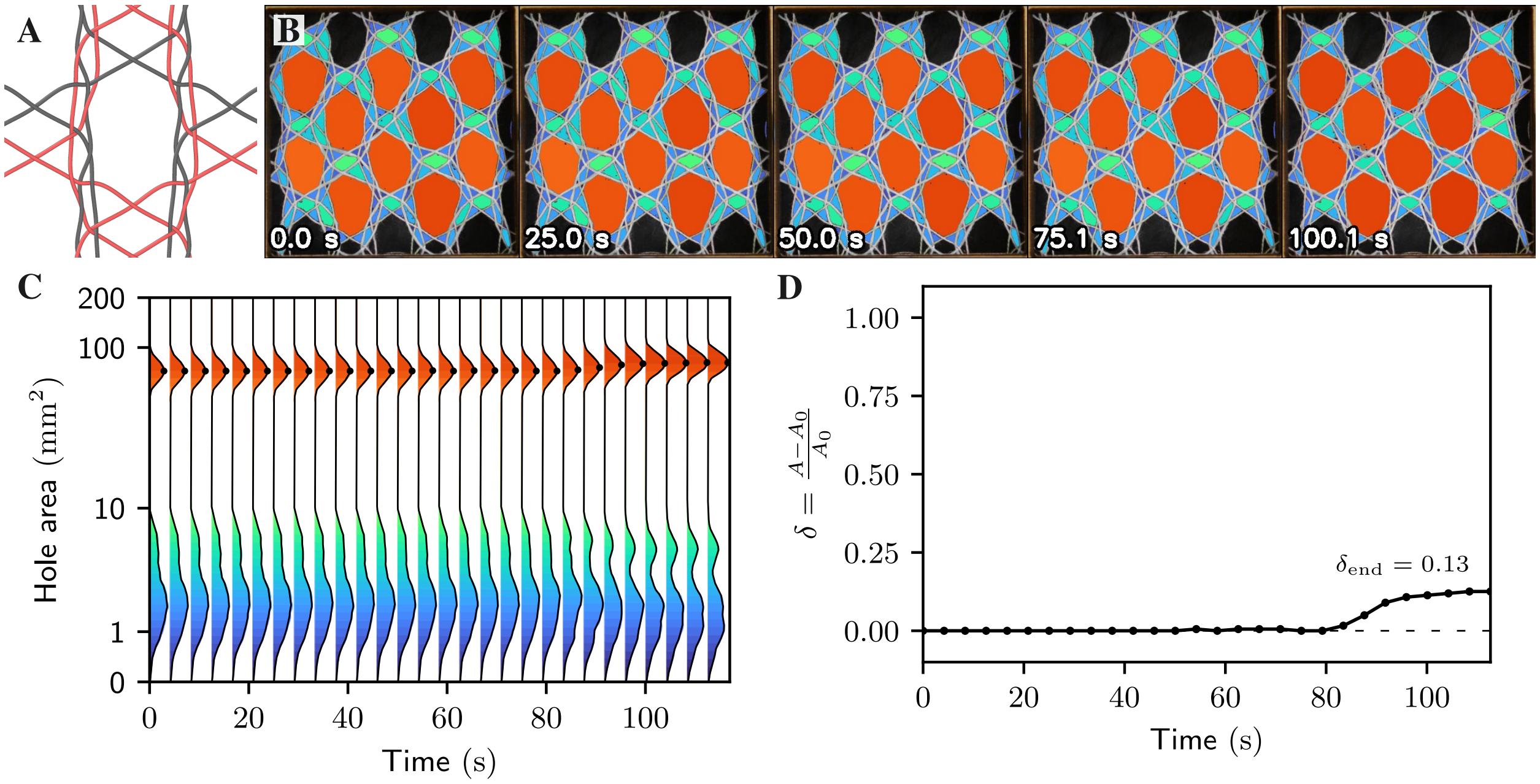

Figure S57: **Analysis of lace structure Rose-8 deformation over time. A** Contraction of textile's unit-cell scheme (red yarns shrink). **B** Time-lapse visualization of the textile structure under deformation. **C** Evolution of the distribution of pore areas over time. **D** Normalized pore area change of the largest areas.

Rose-9

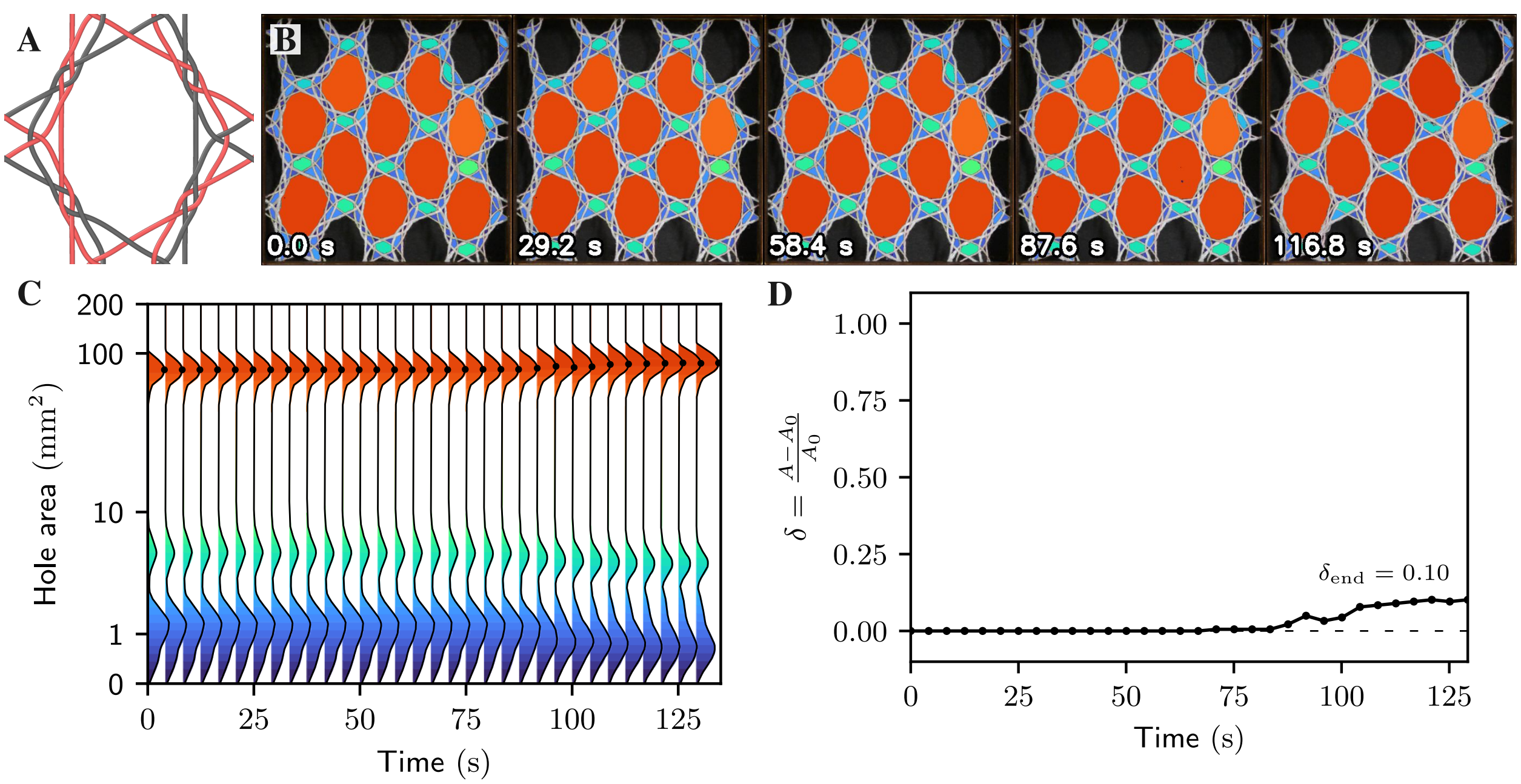

Figure S58: **Analysis of lace structure Rose-9 deformation over time. A** Contraction of textile's unit-cell scheme (red yarns shrink). **B** Time-lapse visualization of the textile structure under deformation. **C** Evolution of the distribution of pore areas over time. **D** Normalized pore area change of the largest areas.

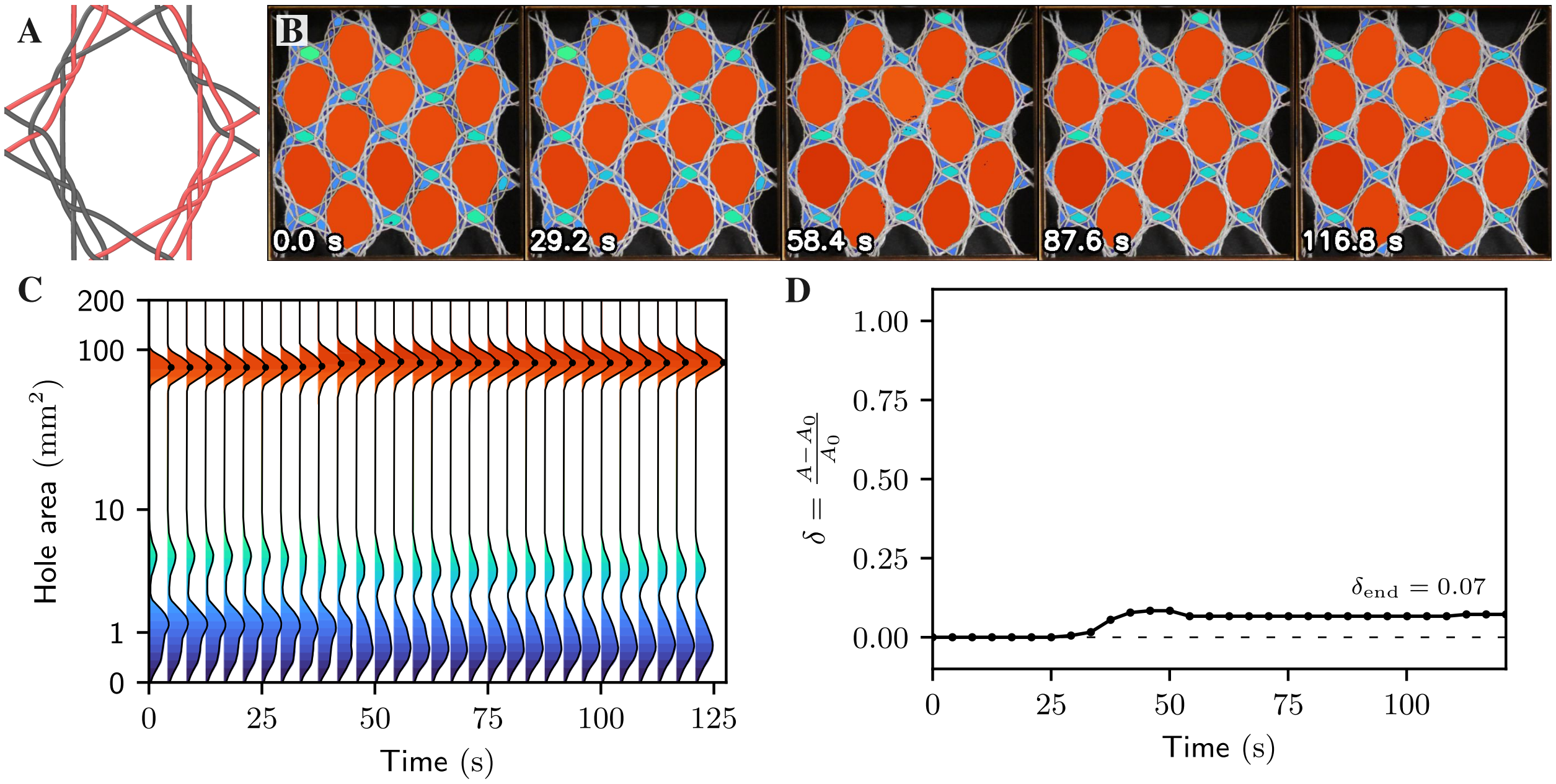

Figure S59: **Analysis of lace structure Rose-9 deformation over time. A** Contraction of textile's unit-cell scheme (red yarns shrink). **B** Time-lapse visualization of the textile structure under deformation. **C** Evolution of the distribution of pore areas over time. **D** Normalized pore area change of the largest areas.

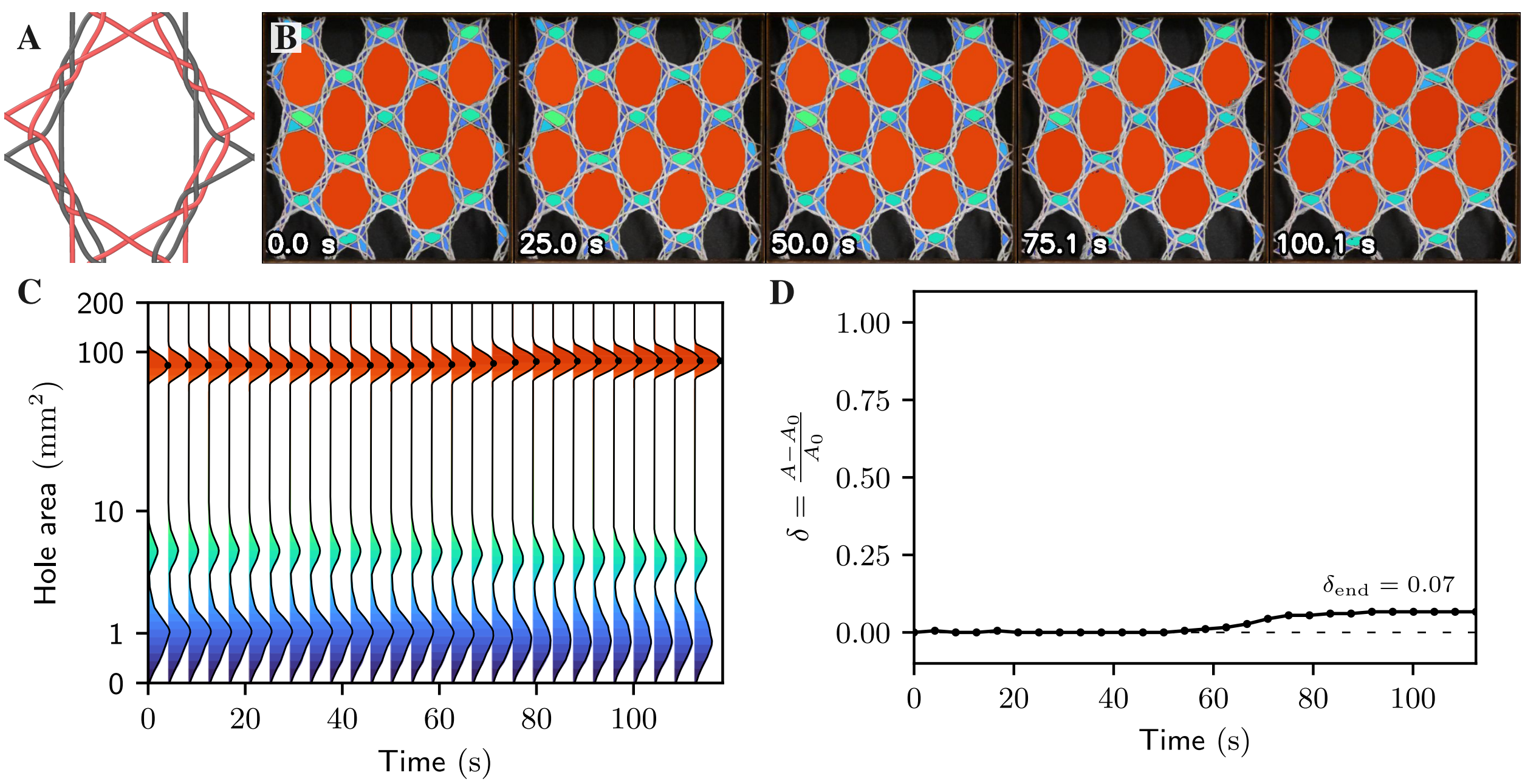

Figure S60: **Analysis of lace structure Rose-9 deformation over time. A** Contraction of textile's unit-cell scheme (red yarns shrink). **B** Time-lapse visualization of the textile structure under deformation. **C** Evolution of the distribution of pore areas over time. **D** Normalized pore area change of the largest areas.

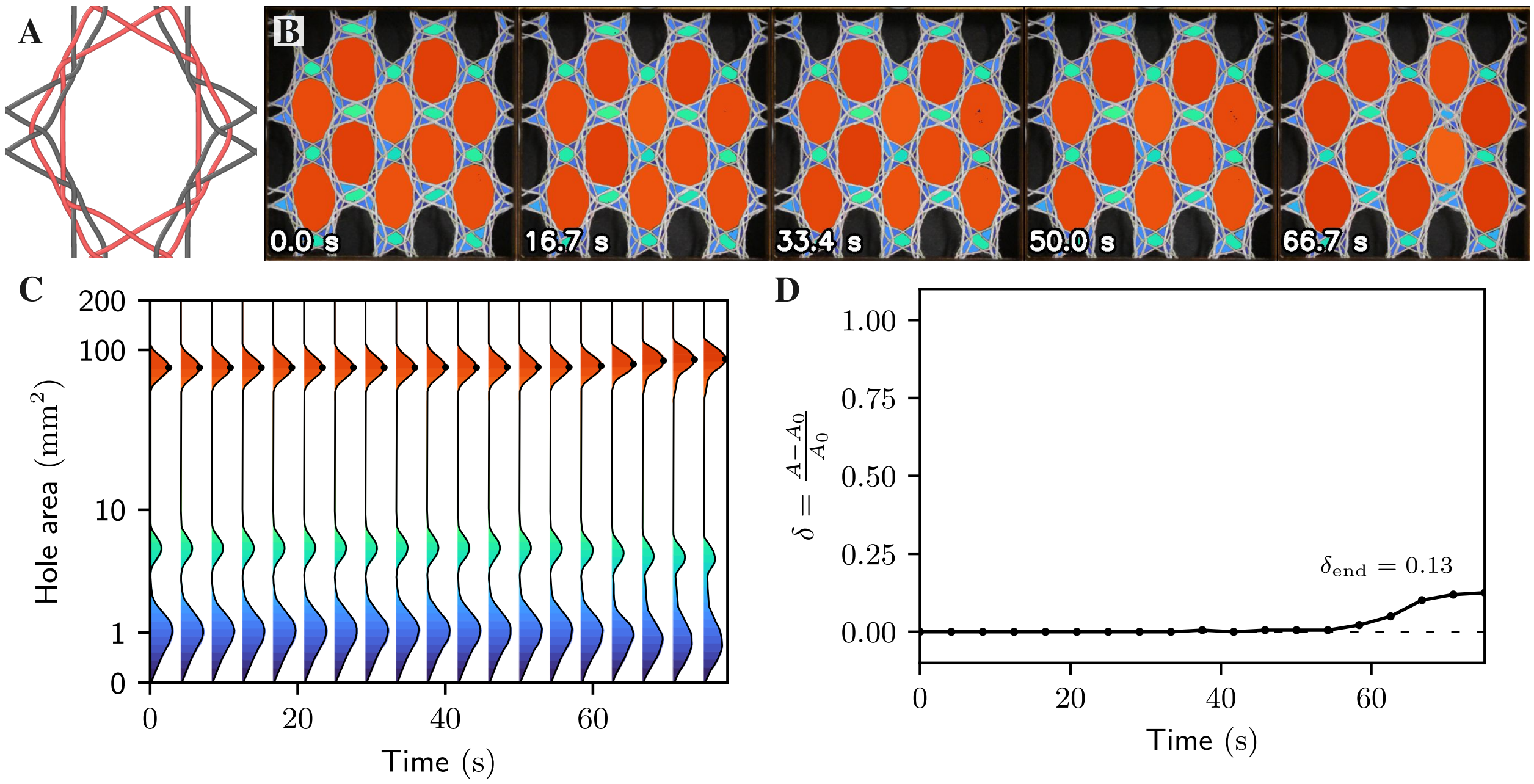

Figure S61: **Analysis of lace structure Rose-9 deformation over time. A** Contraction of textile's unit-cell scheme (red yarns shrink). **B** Time-lapse visualization of the textile structure under deformation. **C** Evolution of the distribution of pore areas over time. **D** Normalized pore area change of the largest areas.

Rose-10

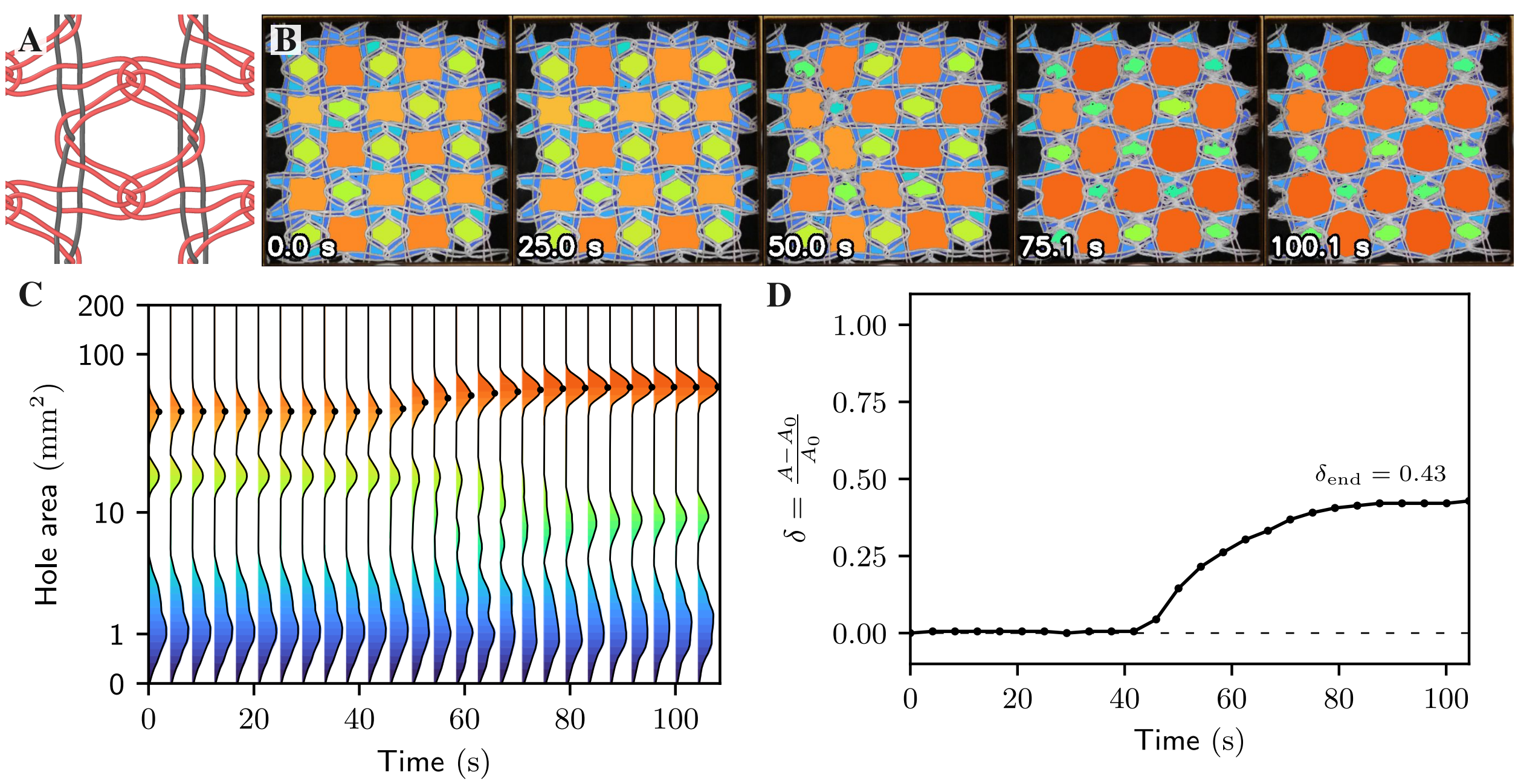

Figure S62: **Analysis of lace structure Rose-10 deformation over time. A** Contraction of textile's unit-cell scheme (red yarns shrink). **B** Time-lapse visualization of the textile structure under deformation. **C** Evolution of the distribution of pore areas over time. **D** Normalized pore area change of the largest areas.

Rose-11

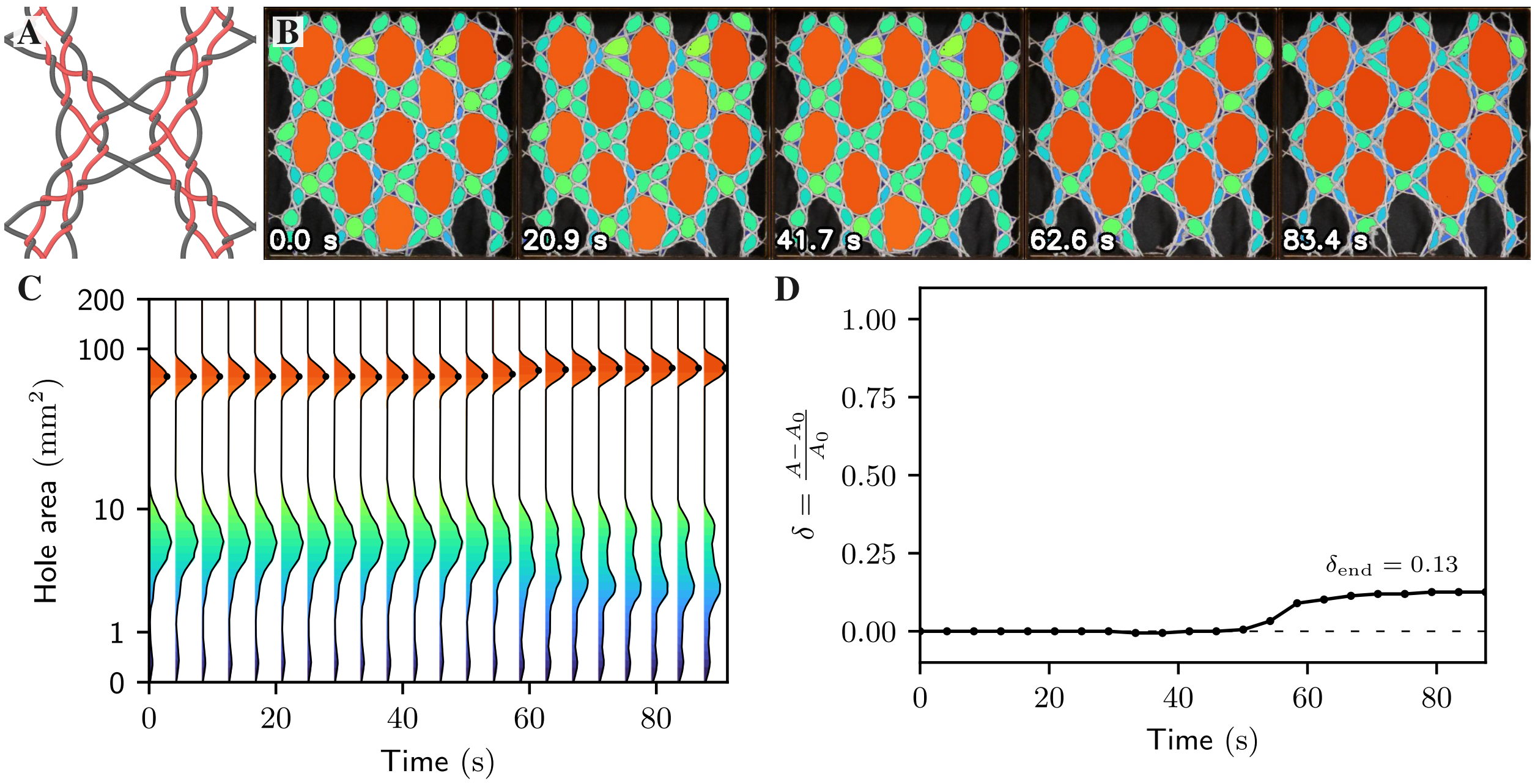

Figure S63: **Analysis of lace structure Rose-11 deformation over time. A** Contraction of textile's unit-cell scheme (red yarns shrink). **B** Time-lapse visualization of the textile structure under deformation. **C** Evolution of the distribution of pore areas over time. **D** Normalized pore area change of the largest areas.

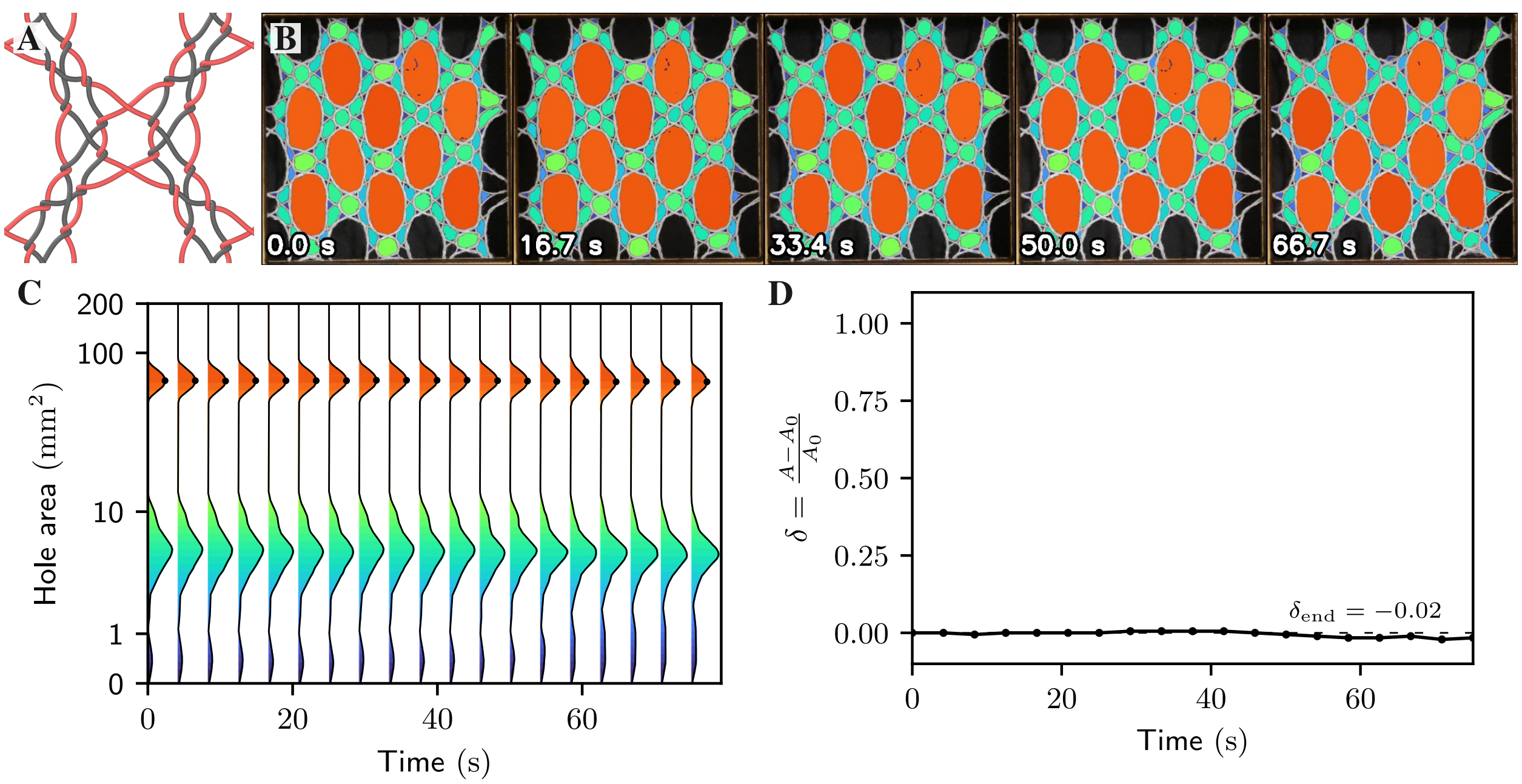

Figure S64: **Analysis of lace structure Rose-11 deformation over time. A** Contraction of textile's unit-cell scheme (red yarns shrink). **B** Time-lapse visualization of the textile structure under deformation. **C** Evolution of the distribution of pore areas over time. **D** Normalized pore area change of the largest areas.

Rose-12

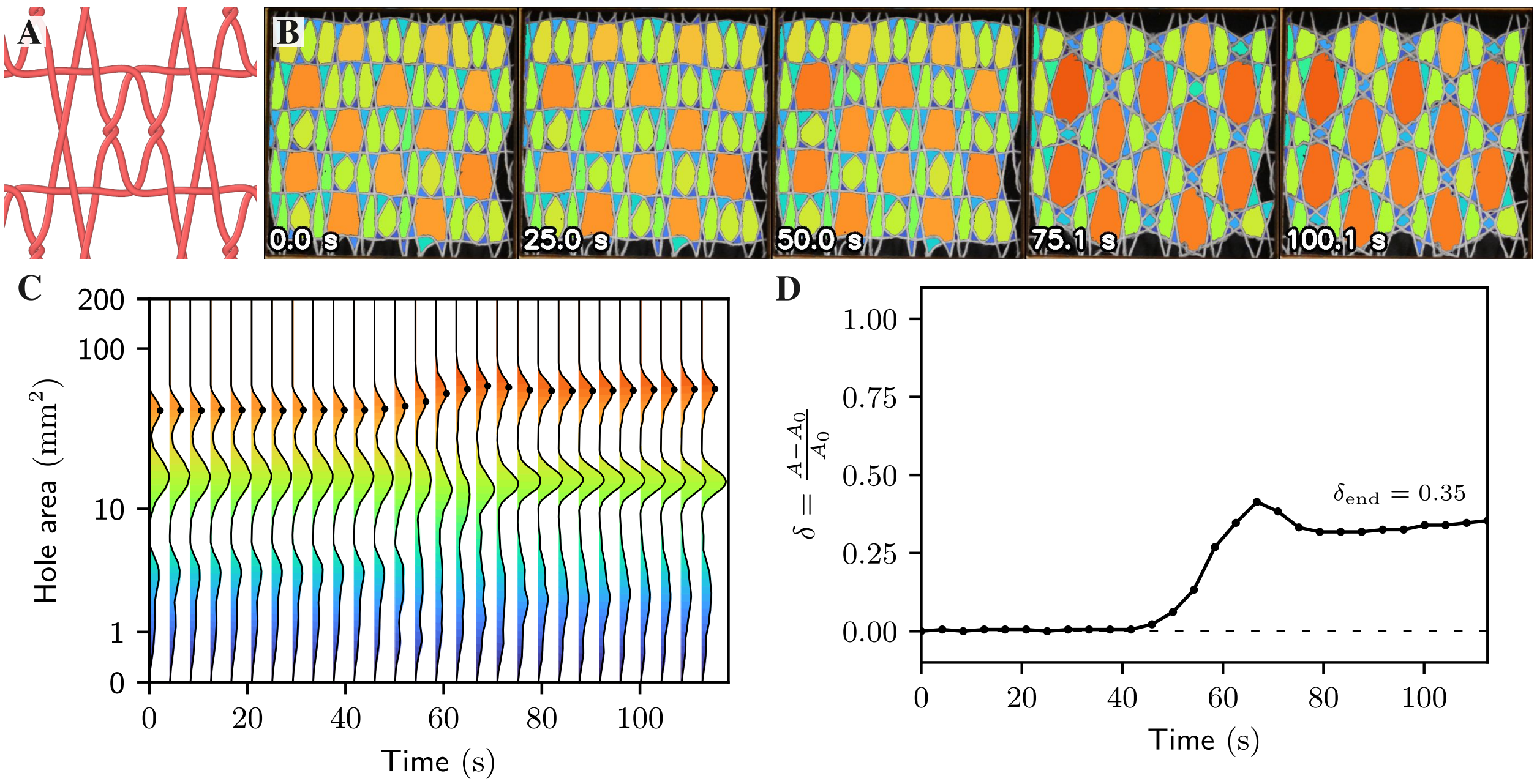

Figure S65: **Analysis of lace structure Rose-12 deformation over time. A** Contraction of textile's unit-cell scheme (red yarns shrink). **B** Time-lapse visualization of the textile structure under deformation. **C** Evolution of the distribution of pore areas over time. **D** Normalized pore area change of the largest areas.

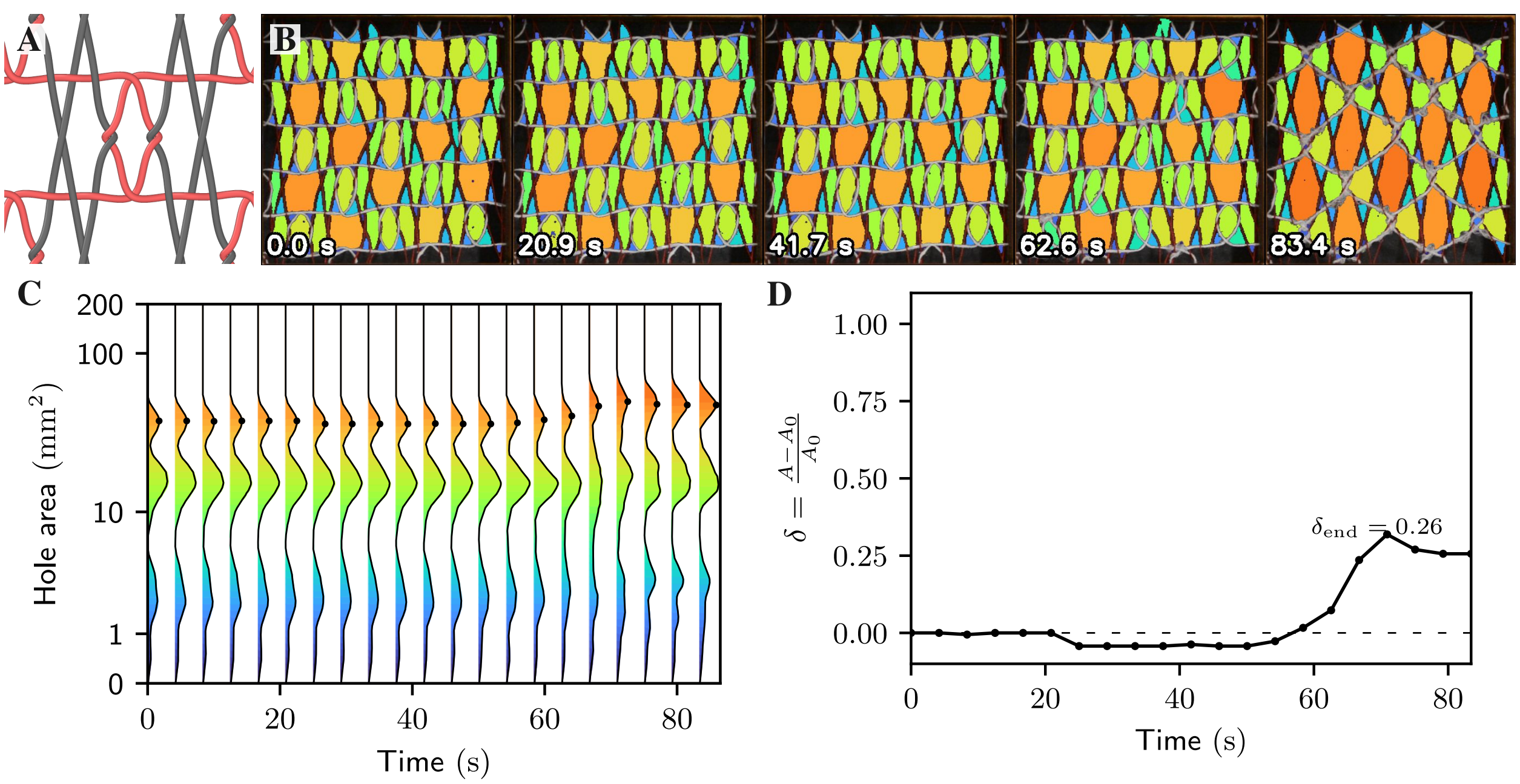

Figure S66: **Analysis of lace structure Rose-12 deformation over time. A** Contraction of textile's unit-cell scheme (red yarns shrink). **B** Time-lapse visualization of the textile structure under deformation. **C** Evolution of the distribution of pore areas over time. **D** Normalized pore area change of the largest areas.

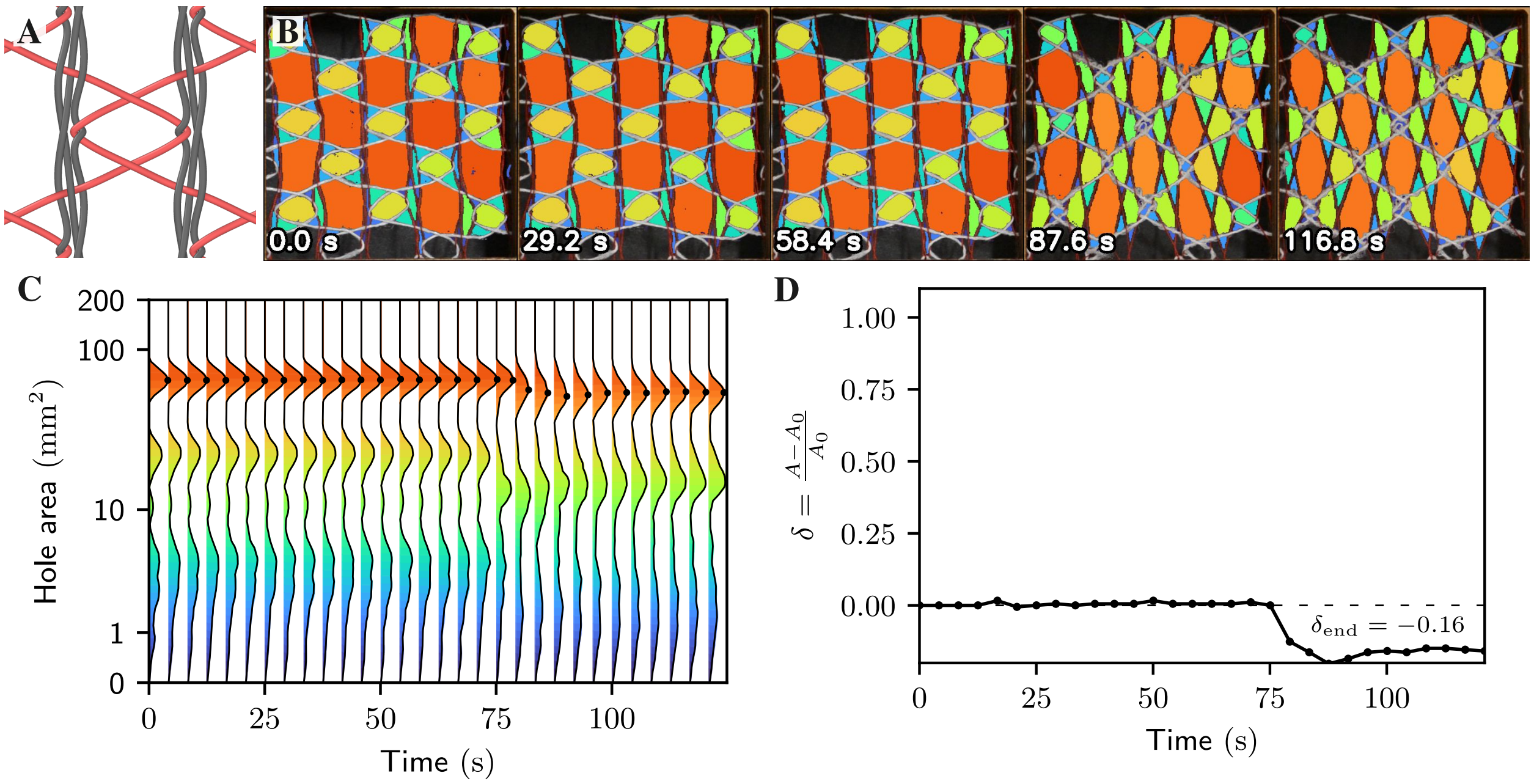

Figure S67: **Analysis of lace structure Rose-12 deformation over time. A** Contraction of textile's unit-cell scheme (red yarns shrink). **B** Time-lapse visualization of the textile structure under deformation. **C** Evolution of the distribution of pore areas over time. **D** Normalized pore area change of the largest areas.

Rose-13

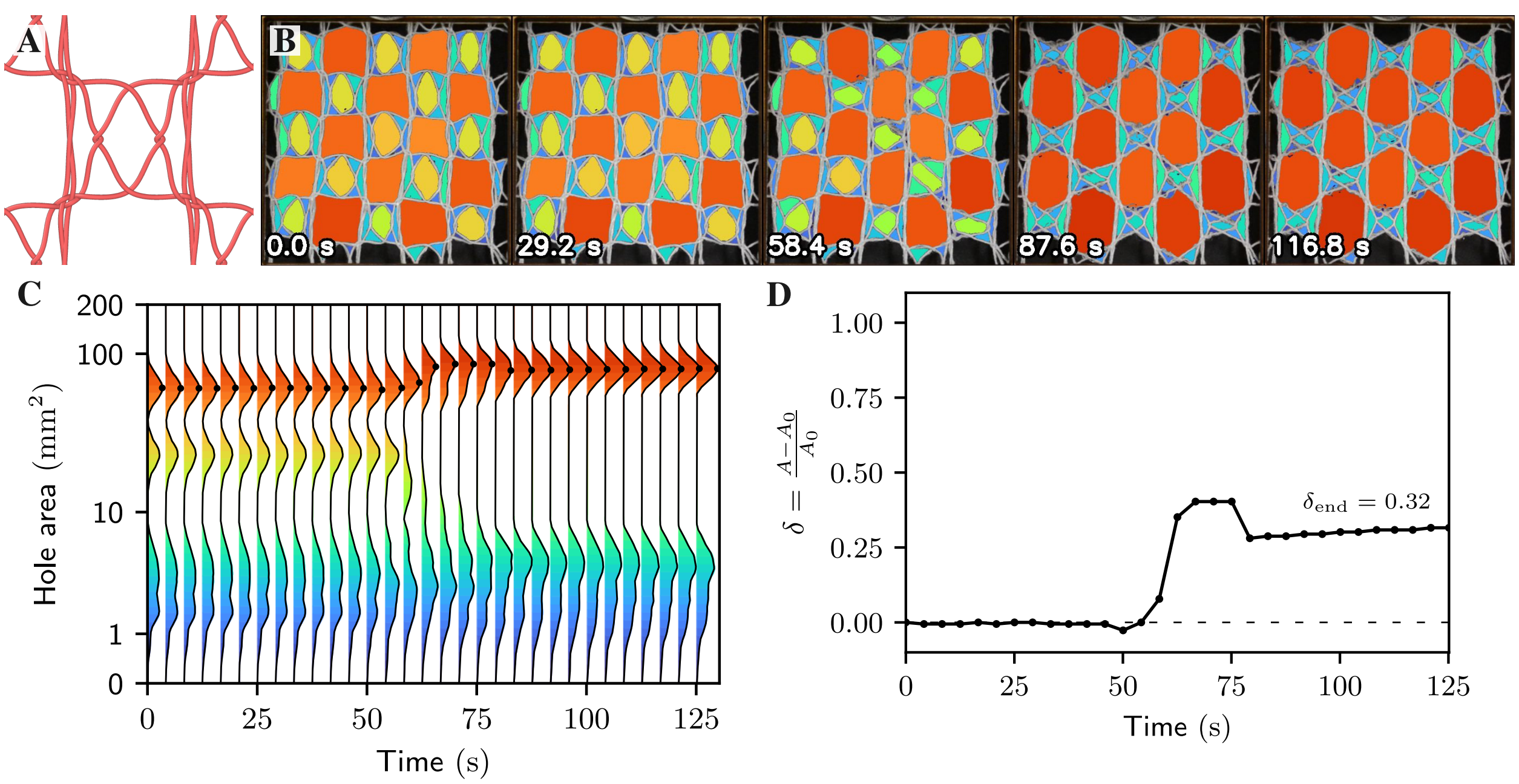

Figure S68: **Analysis of lace structure Rose-13 deformation over time. A** Contraction of textile's unit-cell scheme (red yarns shrink). **B** Time-lapse visualization of the textile structure under deformation. **C** Evolution of the distribution of pore areas over time. **D** Normalized pore area change of the largest areas.

Spruce-1

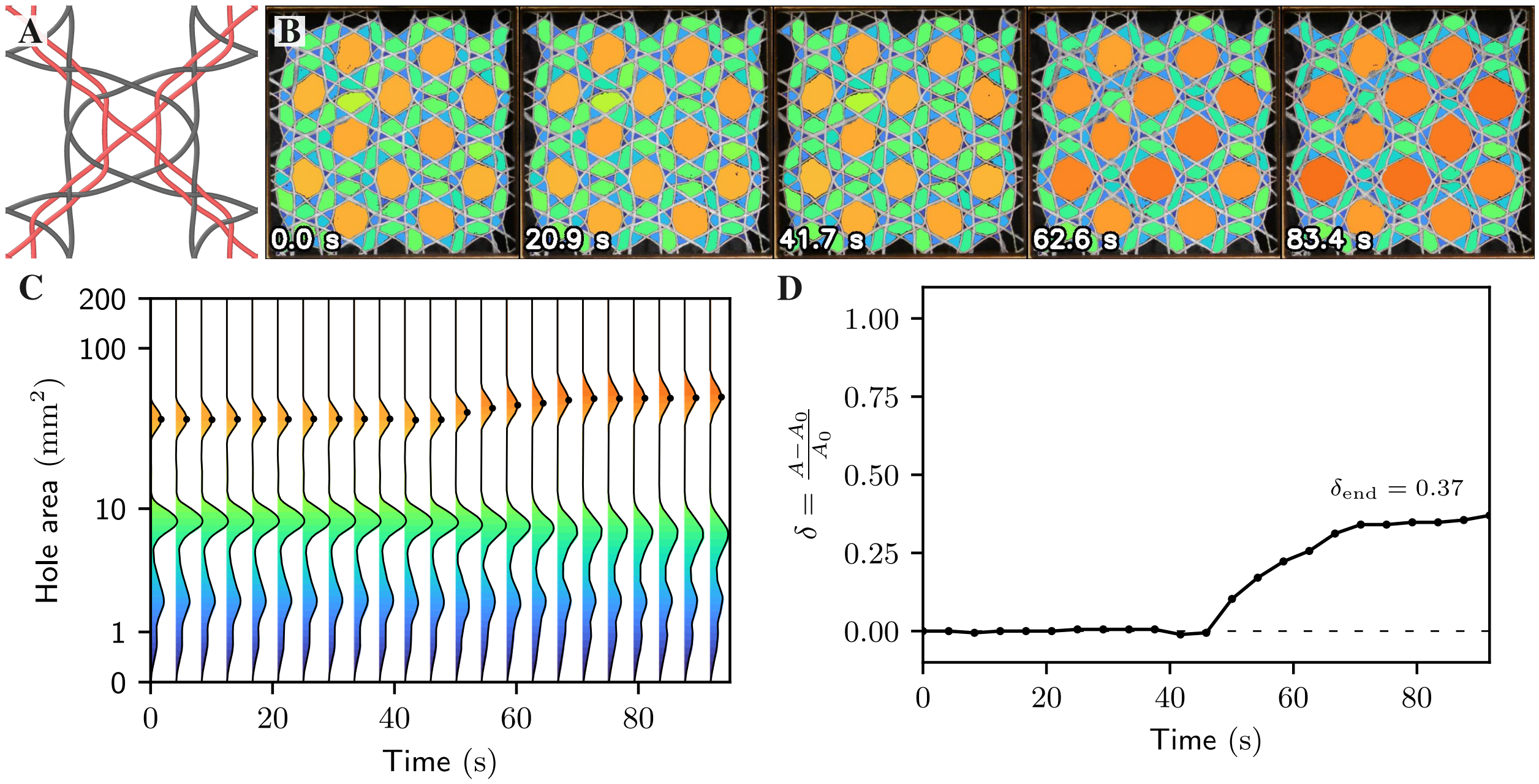

Figure S69: **Analysis of lace structure Spruce-1 deformation over time. A** Contraction of textile's unit-cell scheme (red yarns shrink). **B** Time-lapse visualization of the textile structure under deformation. **C** Evolution of the distribution of pore areas over time. **D** Normalized pore area change of the largest areas.

Simulations of lace structures

- Unit-cell schematic: A drawn schematic diagram of the yarn paths in the structure. The diagram shows one repeating unit or, if it is not square-shaped, two units. Each unique yarn path is marked with its own color. Sec. and Fig. S70

- Snapshots at steps 0, 10, and 20, showing the initial structure, maximum deformation (active bonds shrink by 25%), and subsequent relaxation: Picture of the simulated structures on step 0, 10 and 20. The images are color-coded to indicate the pores between the yarns identified by image analysis. The largest pores are marked in red, and the color gradient shifts towards blue as the pores size decrease. Sec.

- Variation of the relative length of yarns, grouped by type: Graph of the change in yarn length as a function of time. The colors correspond to the colors in the ”Unit-cell schematic” image. Sec.

- Evolution of the distribution of pore areas over time: The waterfall graph shows how the number of pores of different sizes varies in the activated lace over time. The color gradient corresponds to the color coding of the pores marked in the photographs. Sec.

- Relative area change of individual pores: The plot of relative area change, which serves as a summary of the data presented in the previous graph, indicates the direction of change in the size of the pores. Sec.

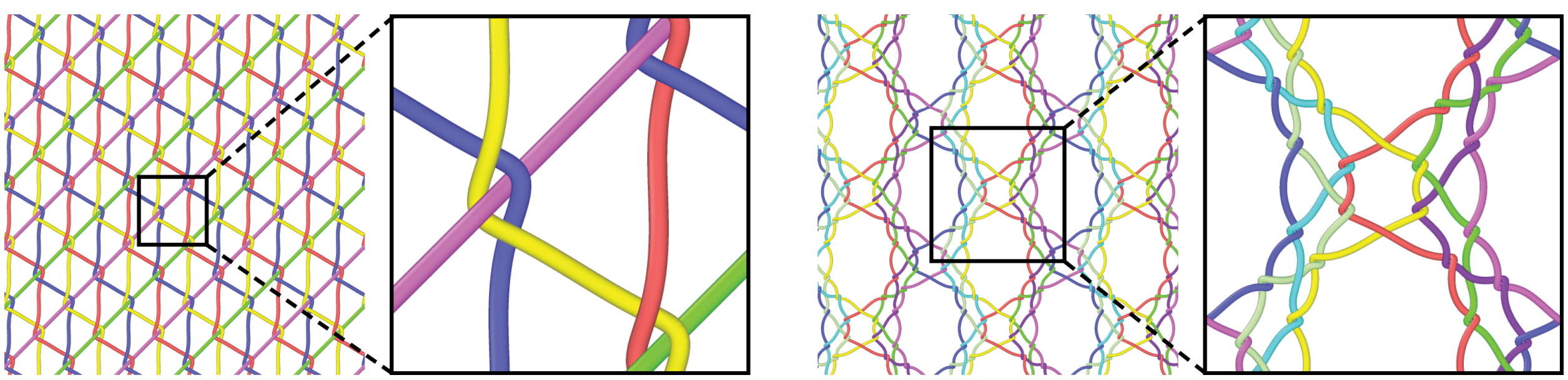

Figure S70: Unit-cell is a square shaped repeating part of the pattern. Each unique yarn path is marked with its own color. Two examples of unit-cells selection.

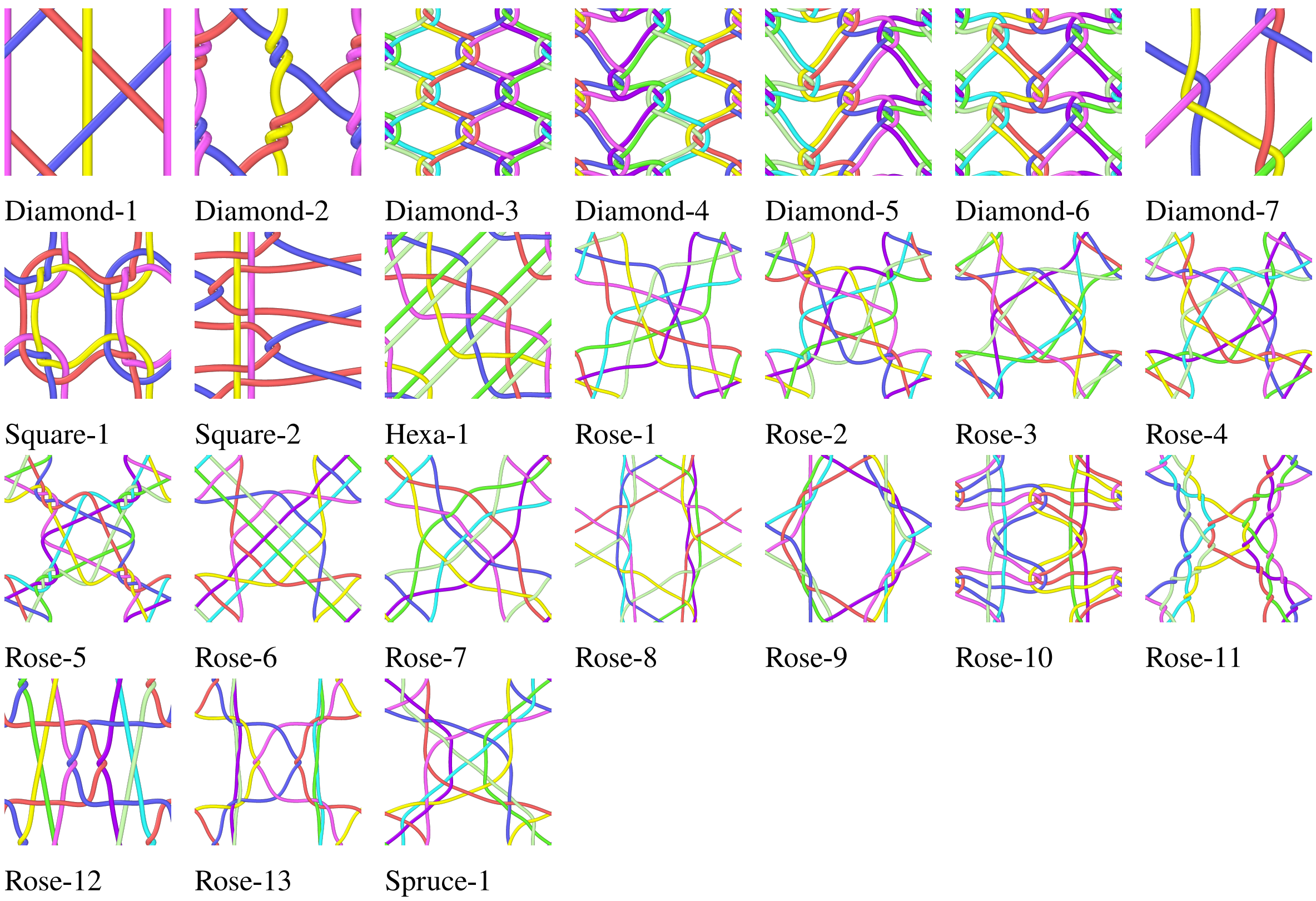

Figure S71: All simulated structures.

Diamond-1

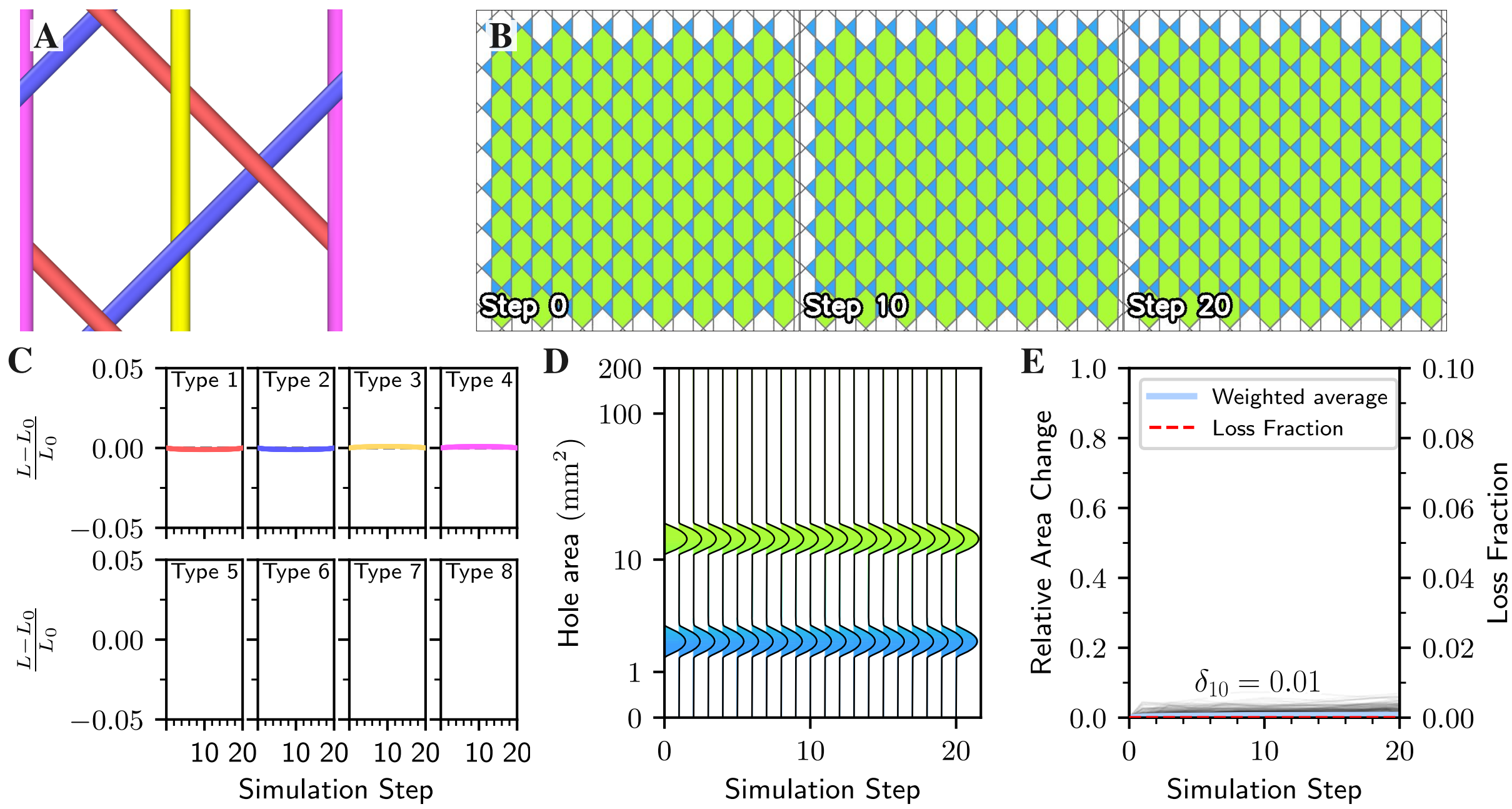

Figure S72: **Simulation of lace structure Diamond-1. A** Unit-cell schematic (active bond types {1, 2, 3, 4}). **B** Snapshots at steps 0, 10, and 20, showing the initial structure, maximum deformation (active bonds shrink by 25%), and subsequent relaxation. **C** Variation of the relative length of yarns, grouped by type. **D** Evolution of the distribution of pore areas over time. **E** Relative area change of individual pores.

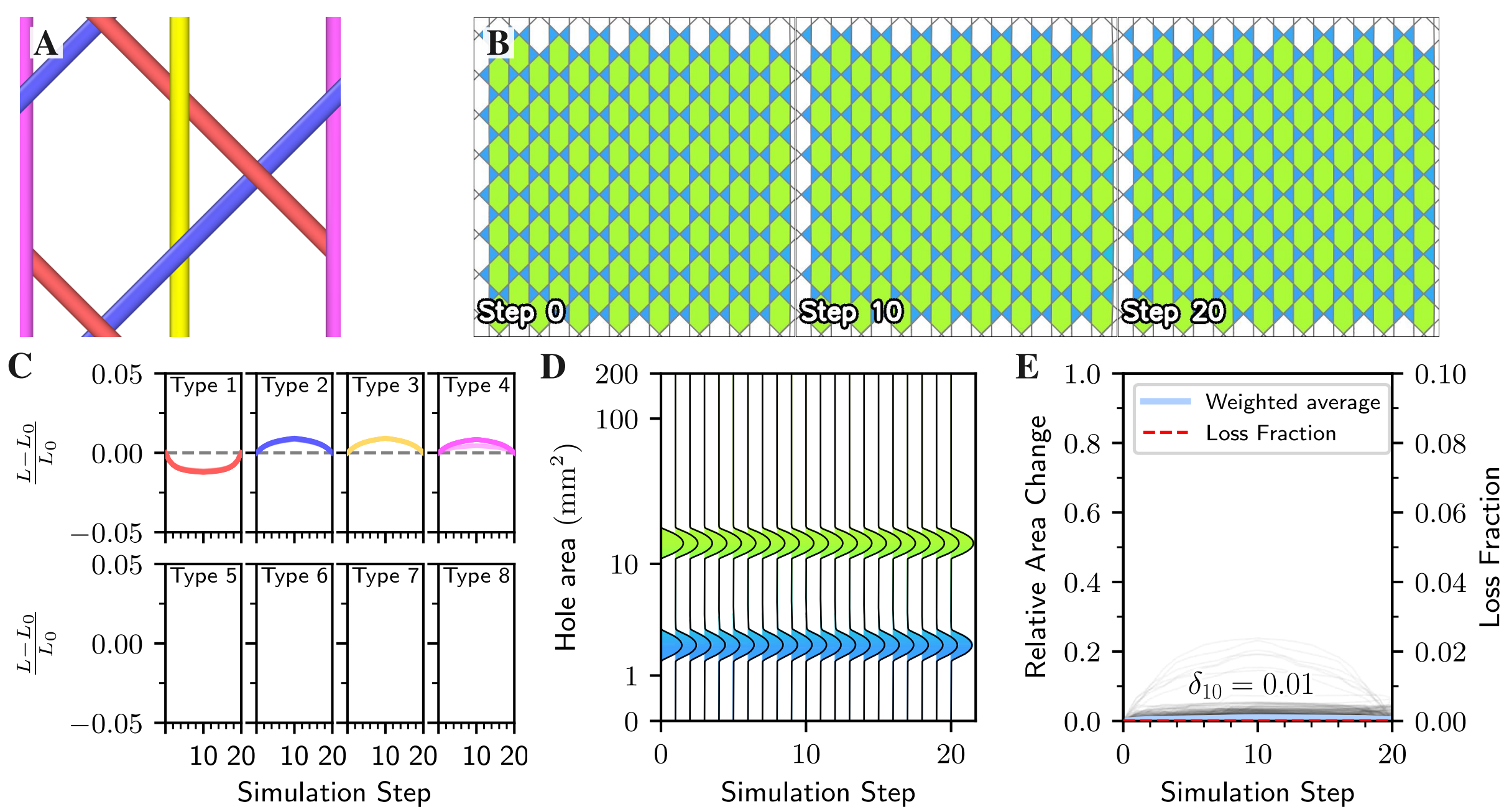

Figure S73: **Simulation of lace structure Diamond-1. A** Unit-cell schematic (active bond types {1}). **B** Snapshots at steps 0, 10, and 20, showing the initial structure, maximum deformation (active bonds shrink by 25%), and subsequent relaxation. **C** Variation of the relative length of yarns, grouped by type. **D** Evolution of the distribution of pore areas over time. **E** Relative area change of individual pores.

Diamond-2

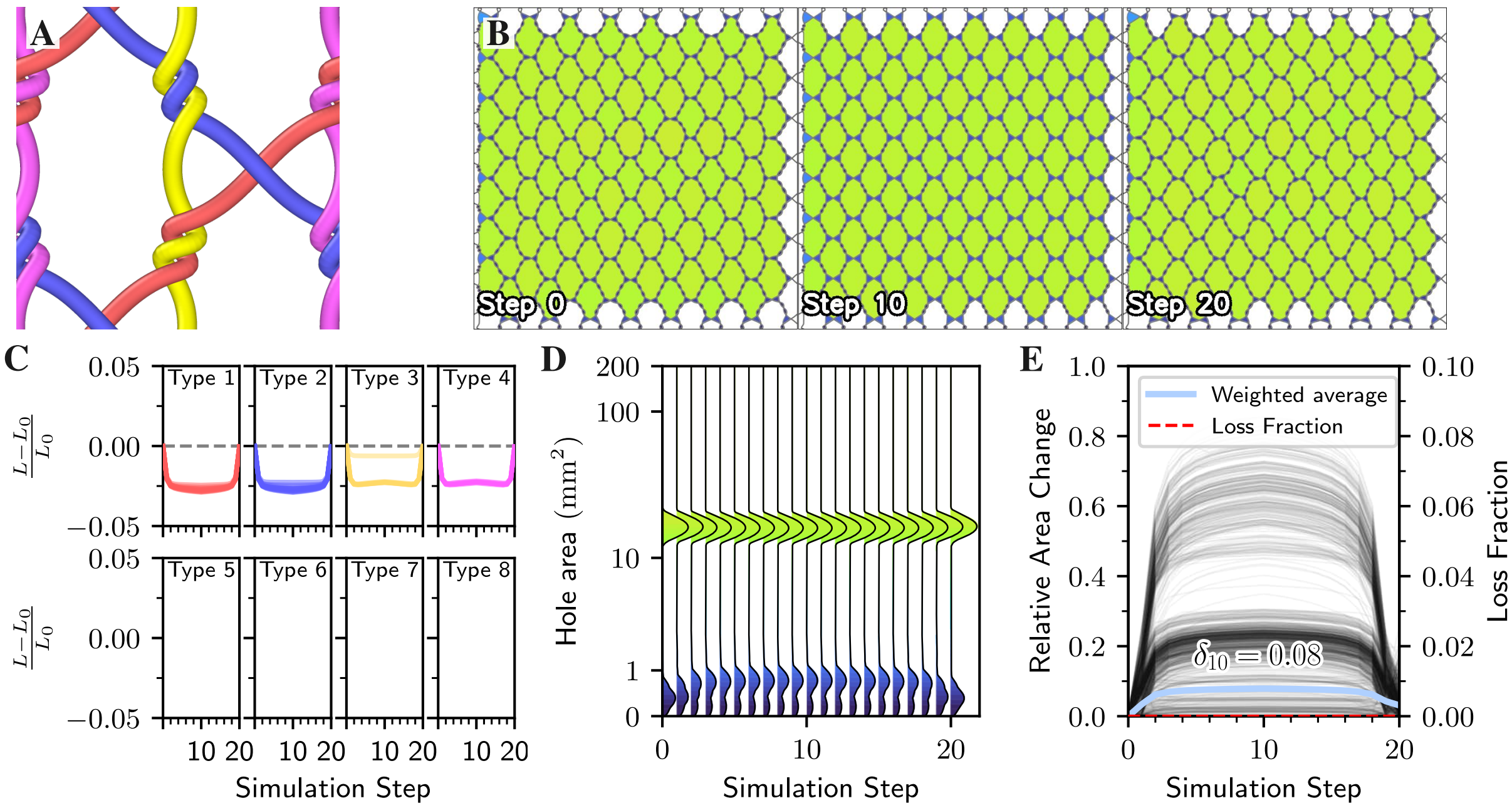

Figure S74: **Simulation of lace structure Diamond-2. A** Unit-cell schematic (active bond types {1, 2, 3, 4}). **B** Snapshots at steps 0, 10, and 20, showing the initial structure, maximum deformation (active bonds shrink by 25%), and subsequent relaxation. **C** Variation of the relative length of yarns, grouped by type. **D** Evolution of the distribution of pore areas over time. **E** Relative area change of individual pores.

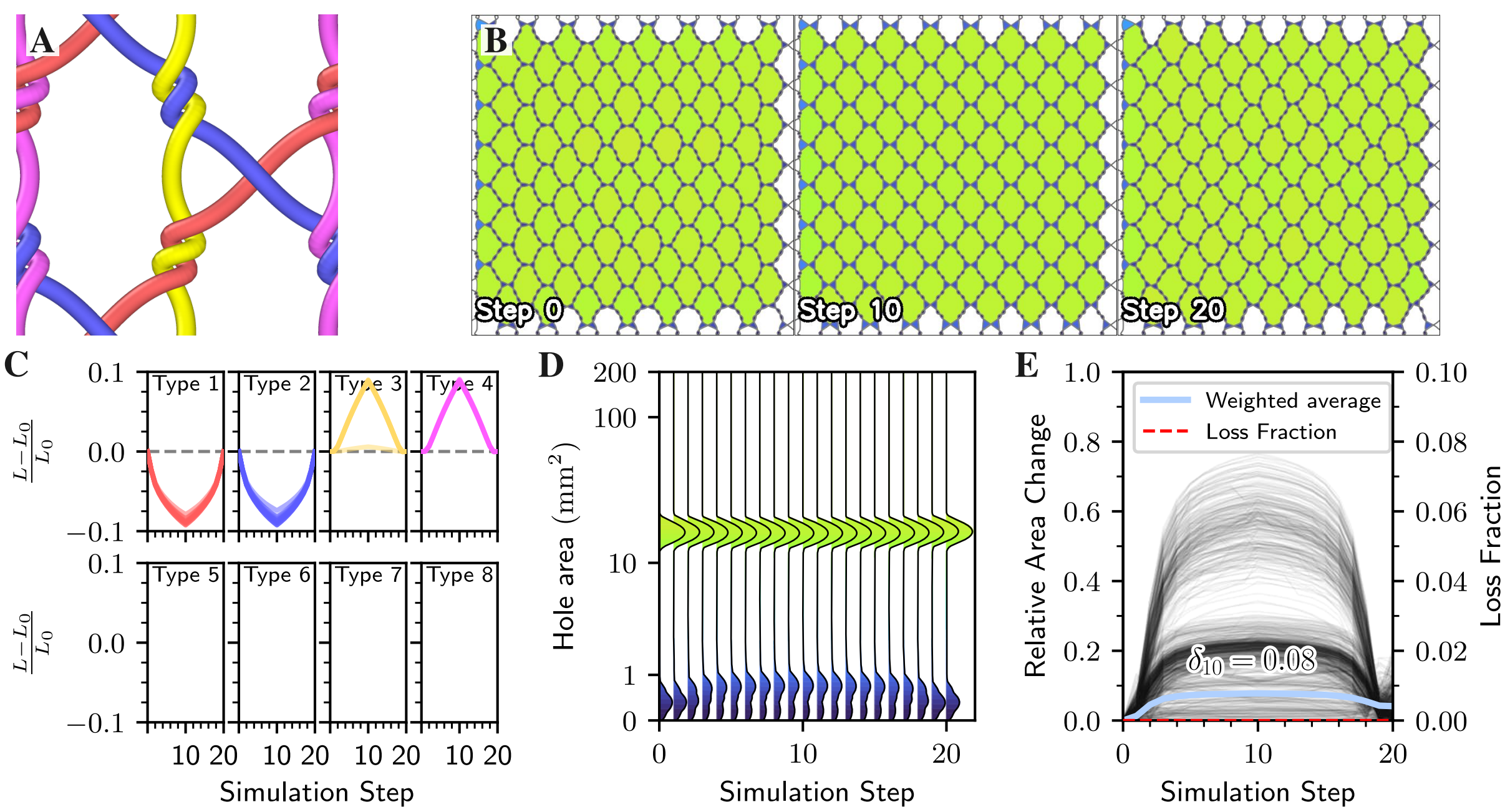

Figure S75: **Simulation of lace structure Diamond-2. A** Unit-cell schematic (active bond types {1, 2}). **B** Snapshots at steps 0, 10, and 20, showing the initial structure, maximum deformation (active bonds shrink by 25%), and subsequent relaxation. **C** Variation of the relative length of yarns, grouped by type. **D** Evolution of the distribution of pore areas over time. **E** Relative area change of individual pores.

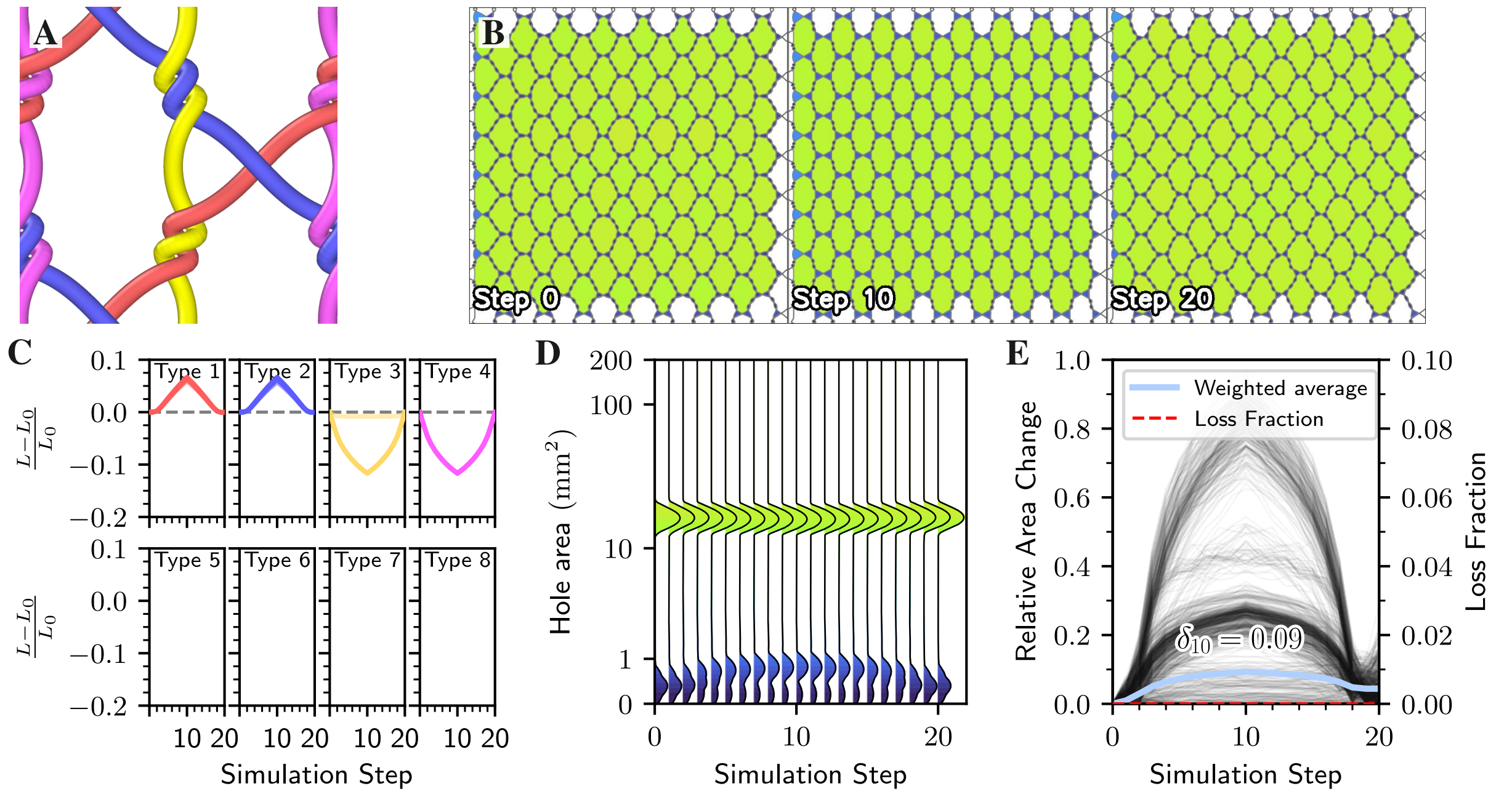

Figure S76: **Simulation of lace structure Diamond-2. A** Unit-cell schematic (active bond types {3, 4}). **B** Snapshots at steps 0, 10, and 20, showing the initial structure, maximum deformation (active bonds shrink by 25%), and subsequent relaxation. **C** Variation of the relative length of yarns, grouped by type. **D** Evolution of the distribution of pore areas over time. **E** Relative area change of individual pores.

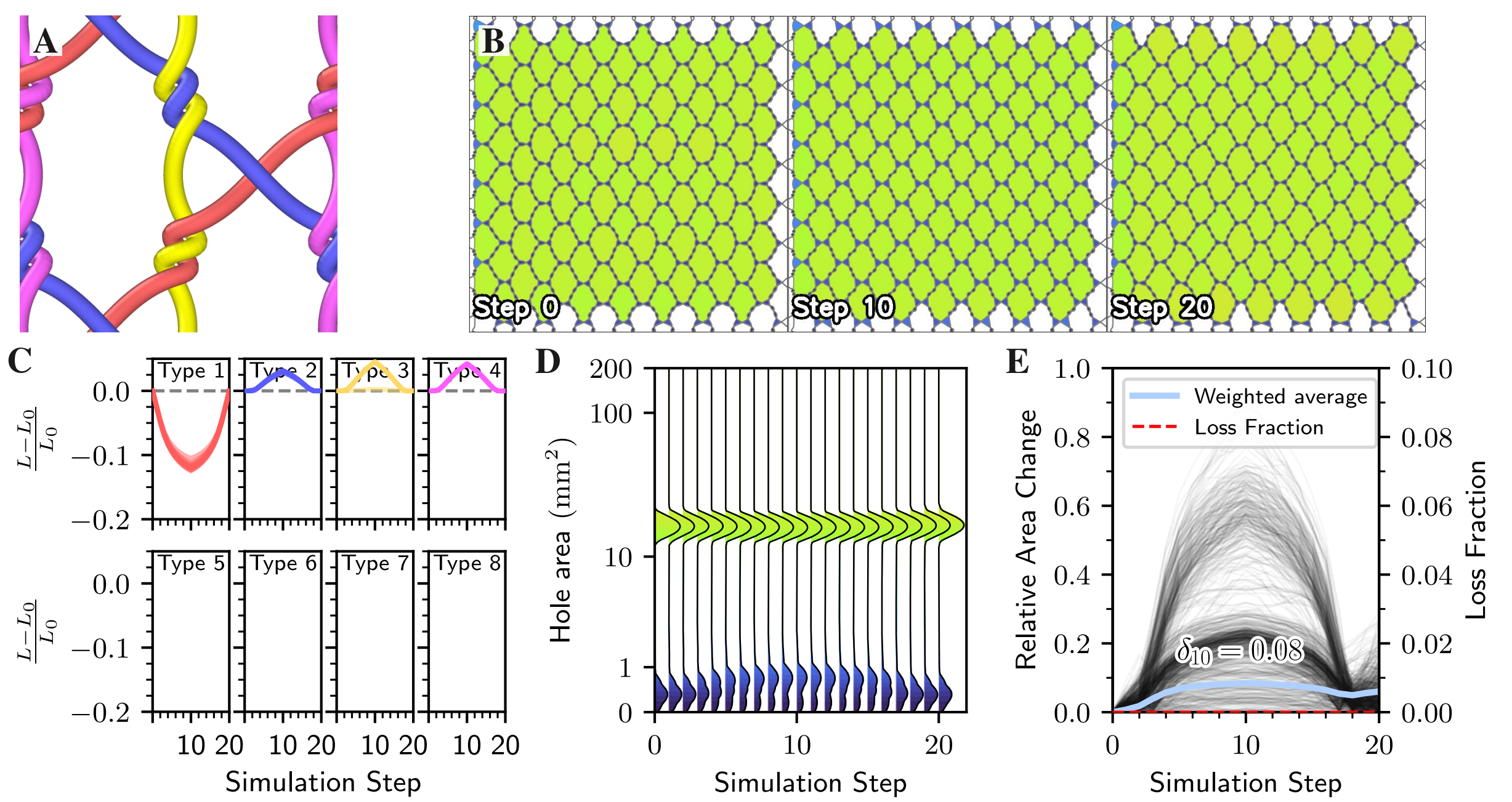

Figure S77: **Simulation of lace structure Diamond-2. A** Unit-cell schematic (active bond types {1}). **B** Snapshots at steps 0, 10, and 20, showing the initial structure, maximum deformation (active bonds shrink by 25%), and subsequent relaxation. **C** Variation of the relative length of yarns, grouped by type. **D** Evolution of the distribution of pore areas over time. **E** Relative area change of individual pores.

Diamond-3

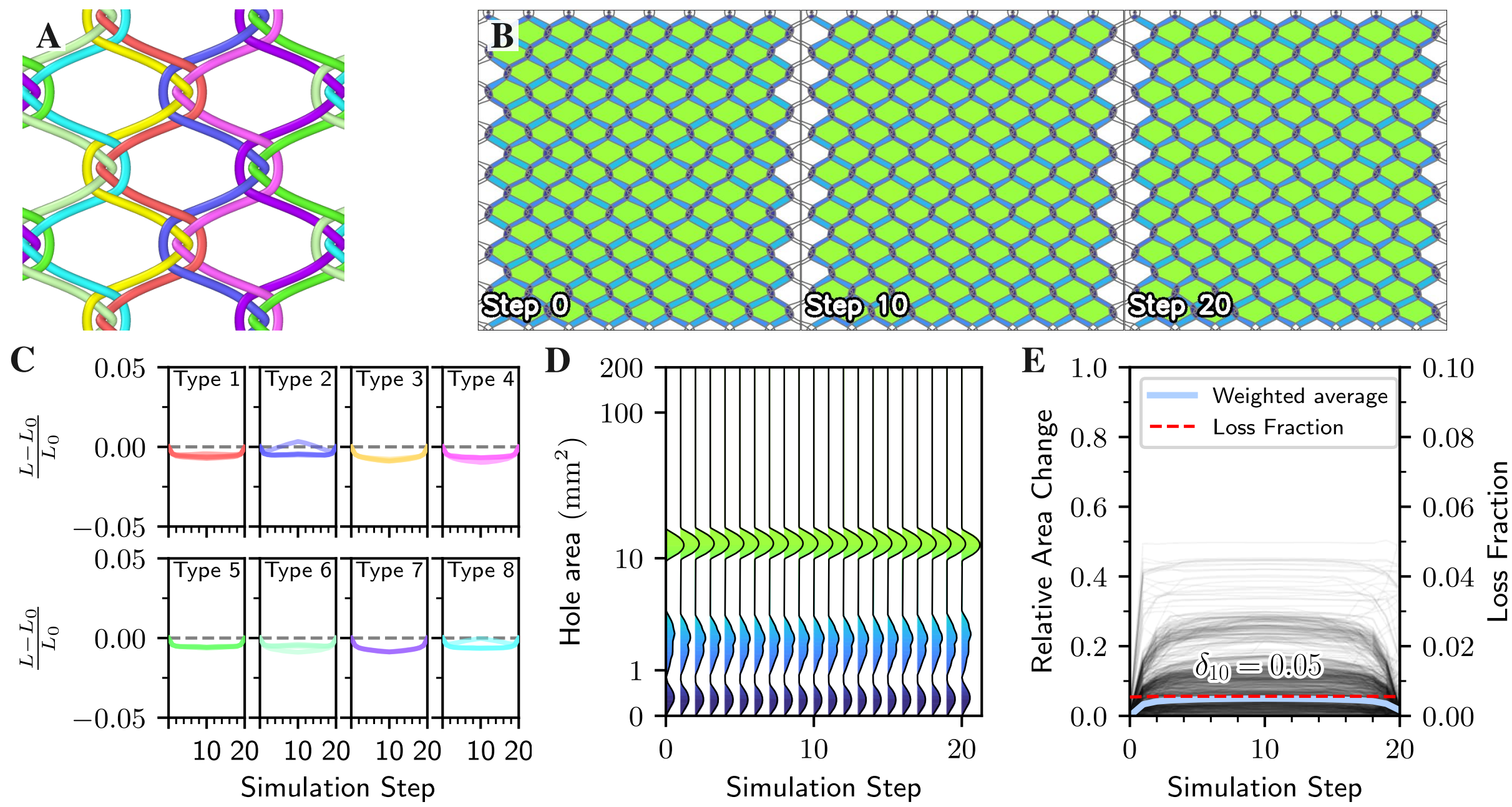

Figure S78: **Simulation of lace structure Diamond-3. A** Unit-cell schematic (active bond types {1, 2, 3, 4, 5, 6, 7, 8}). **B** Snapshots at steps 0, 10, and 20, showing the initial structure, maximum deformation (active bonds shrink by 25%), and subsequent relaxation. **C** Variation of the relative length of yarns, grouped by type. **D** Evolution of the distribution of pore areas over time. **E** Relative area change of individual pores.

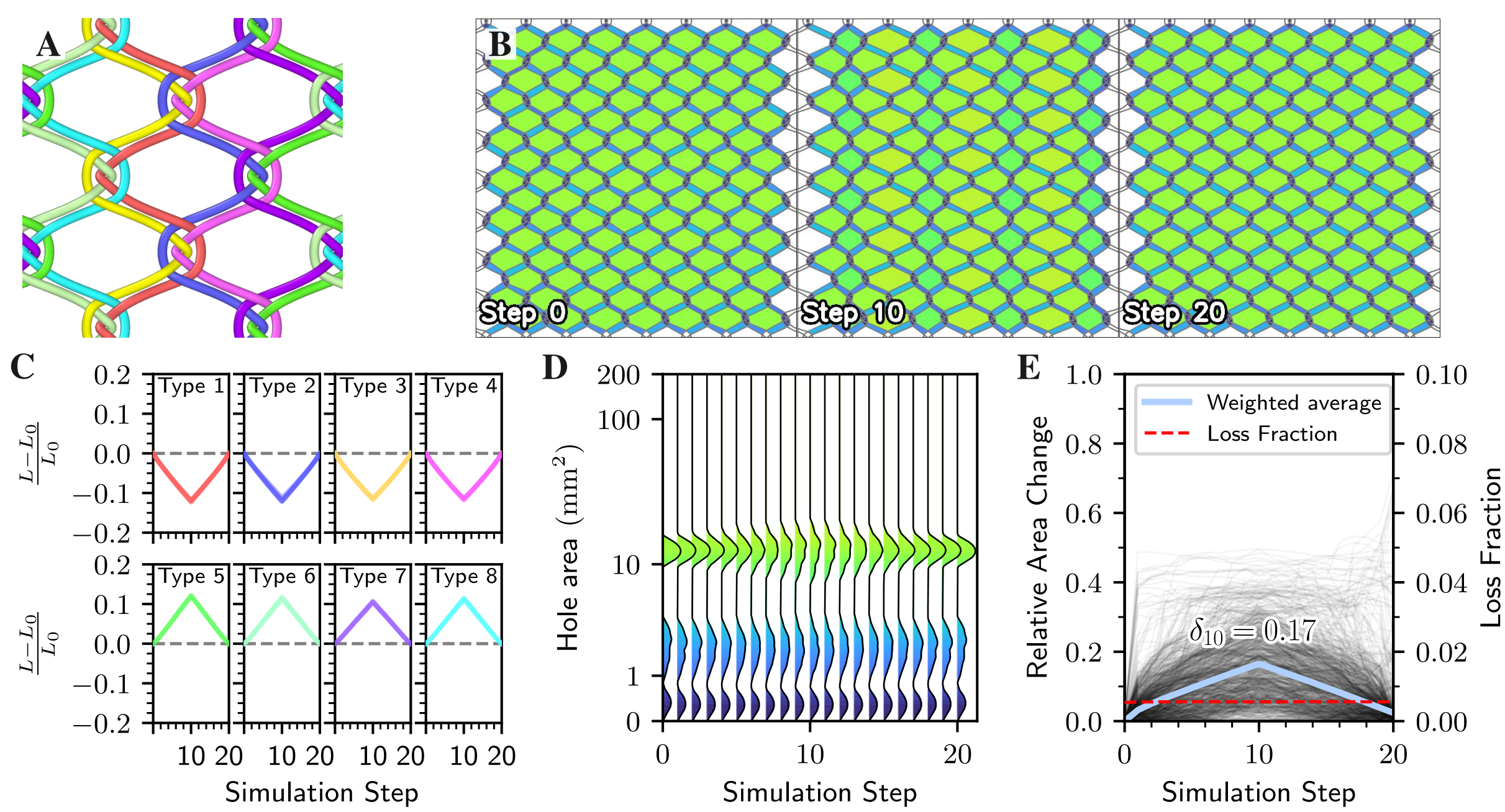

Figure S79: **Simulation of lace structure Diamond-3. A** Unit-cell schematic (active bond types {1, 2, 3, 4}). **B** Snapshots at steps 0, 10, and 20, showing the initial structure, maximum deformation (active bonds shrink by 25%), and subsequent relaxation. **C** Variation of the relative length of yarns, grouped by type. **D** Evolution of the distribution of pore areas over time. **E** Relative area change of individual pores.

Diamond-4

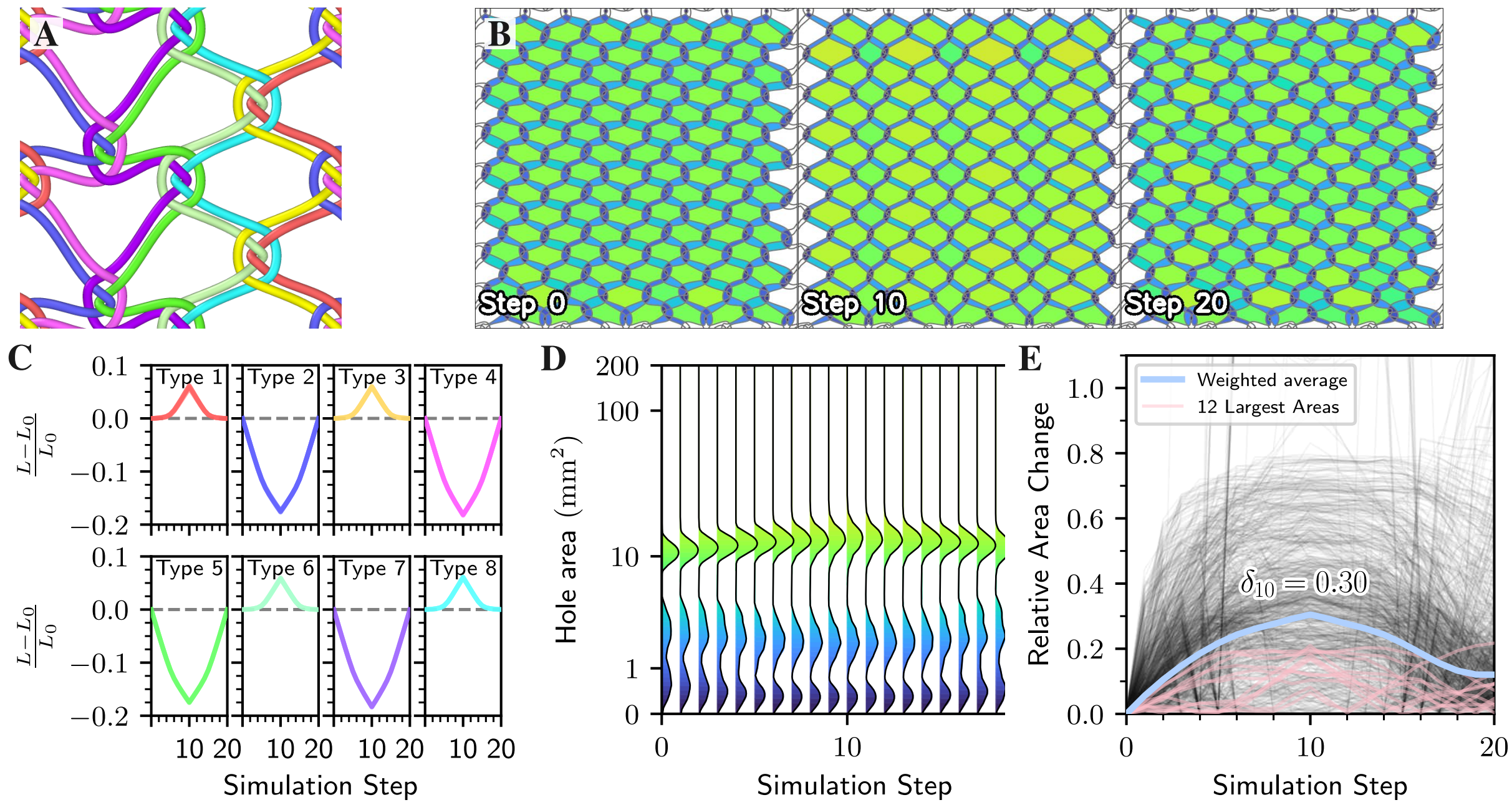

Figure S80: **Simulation of lace structure Diamond-4. A** Unit-cell schematic (active bond types {2, 4, 5, 7}). **B** Snapshots at steps 0, 10, and 20, showing the initial structure, maximum deformation (active bonds shrink by 25%), and subsequent relaxation. **C** Variation of the relative length of yarns, grouped by type. **D** Evolution of the distribution of pore areas over time. **E** Relative area change of individual pores.

Diamond-5

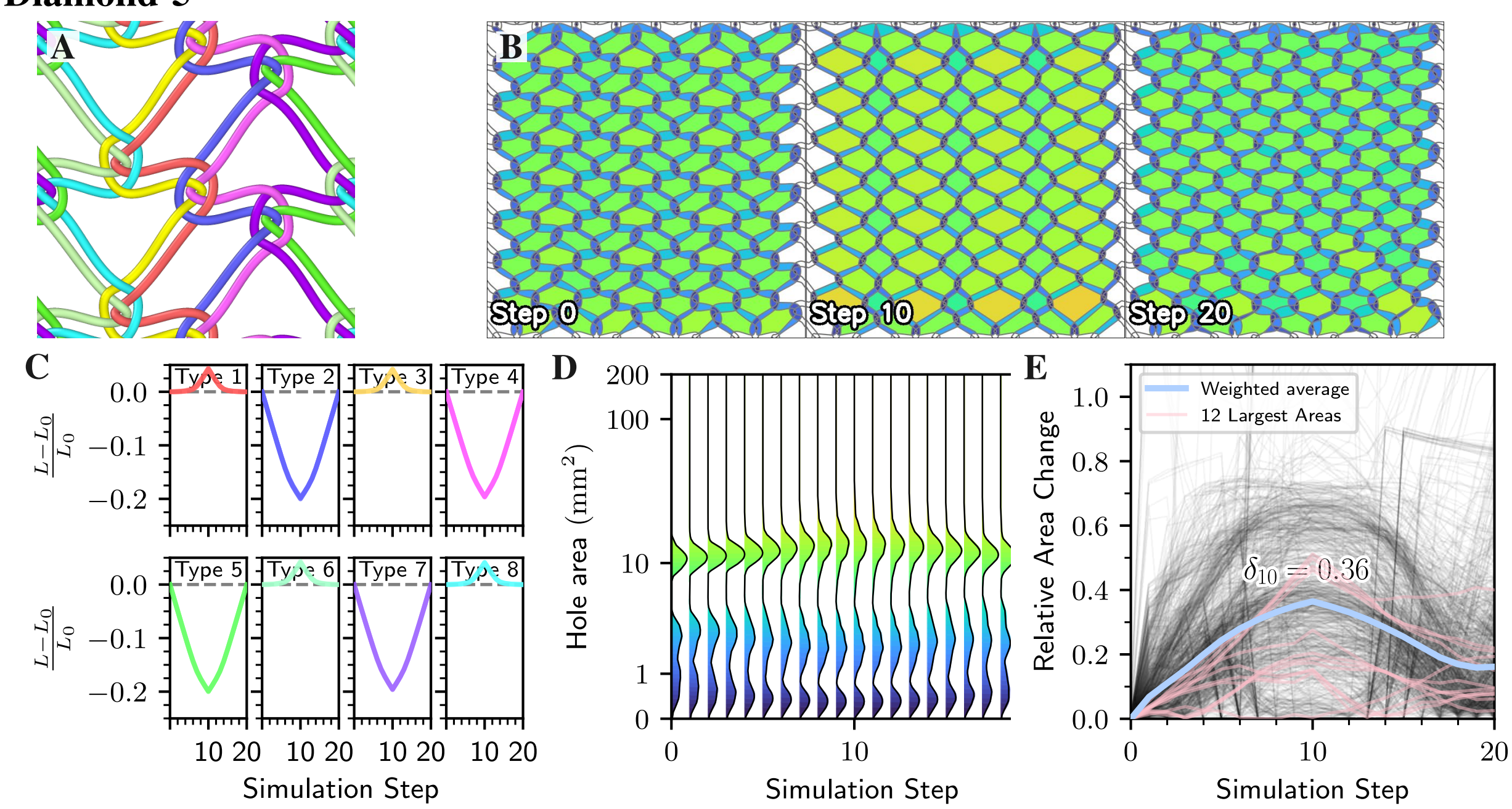

Figure S81: **Simulation of lace structure Diamond-5. A** Unit-cell schematic (active bond types {2, 4, 5, 7}). **B** Snapshots at steps 0, 10, and 20, showing the initial structure, maximum deformation (active bonds shrink by 25%), and subsequent relaxation. **C** Variation of the relative length of yarns, grouped by type. **D** Evolution of the distribution of pore areas over time. **E** Relative area change of individual pores.

Diamond-6

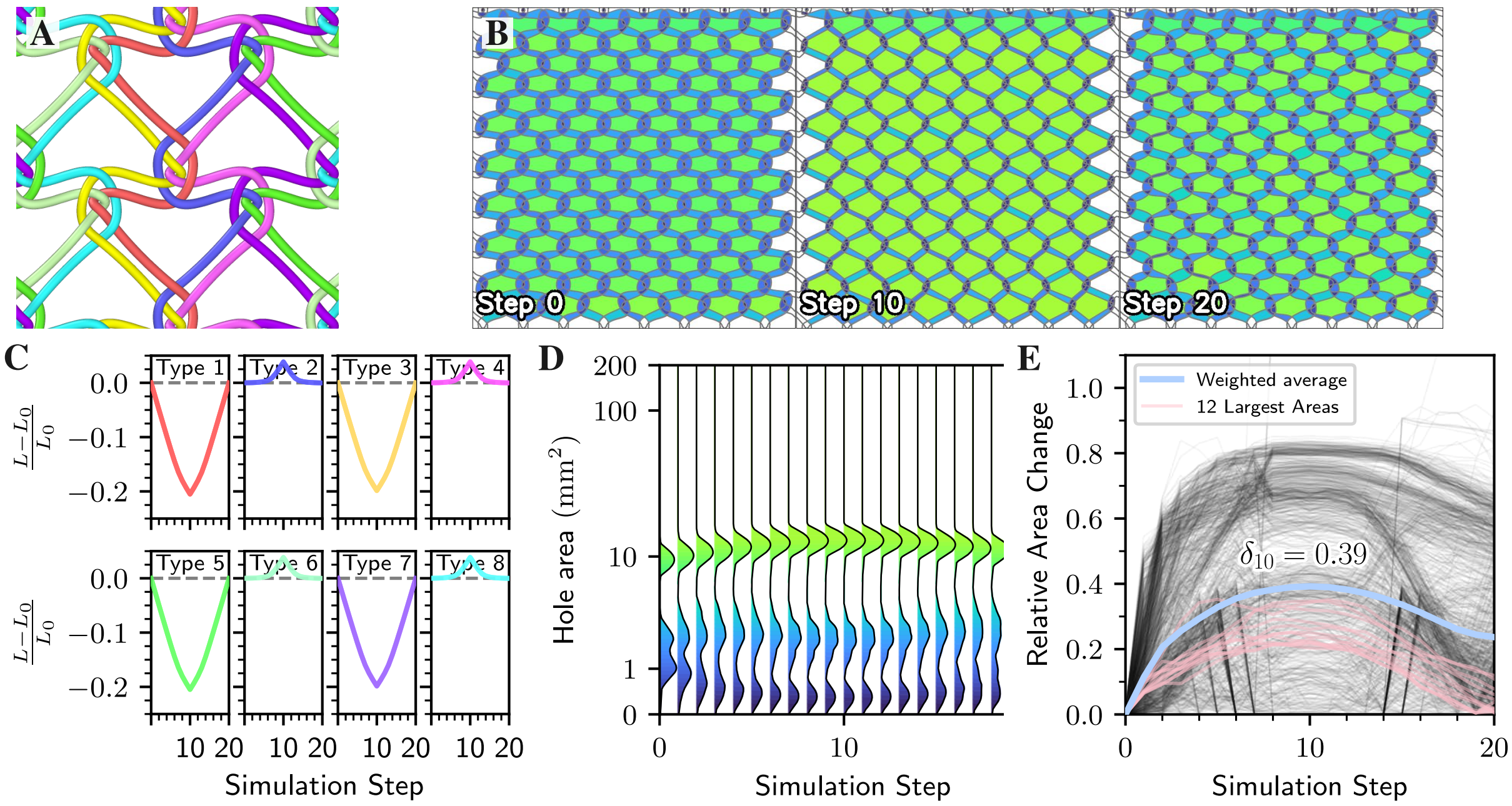

Figure S82: **Simulation of lace structure Diamond-6. A** Unit-cell schematic (active bond types {1, 3, 5, 7}). **B** Snapshots at steps 0, 10, and 20, showing the initial structure, maximum deformation (active bonds shrink by 25%), and subsequent relaxation. **C** Variation of the relative length of yarns, grouped by type. **D** Evolution of the distribution of pore areas over time. **E** Relative area change of individual pores.

Diamond-7

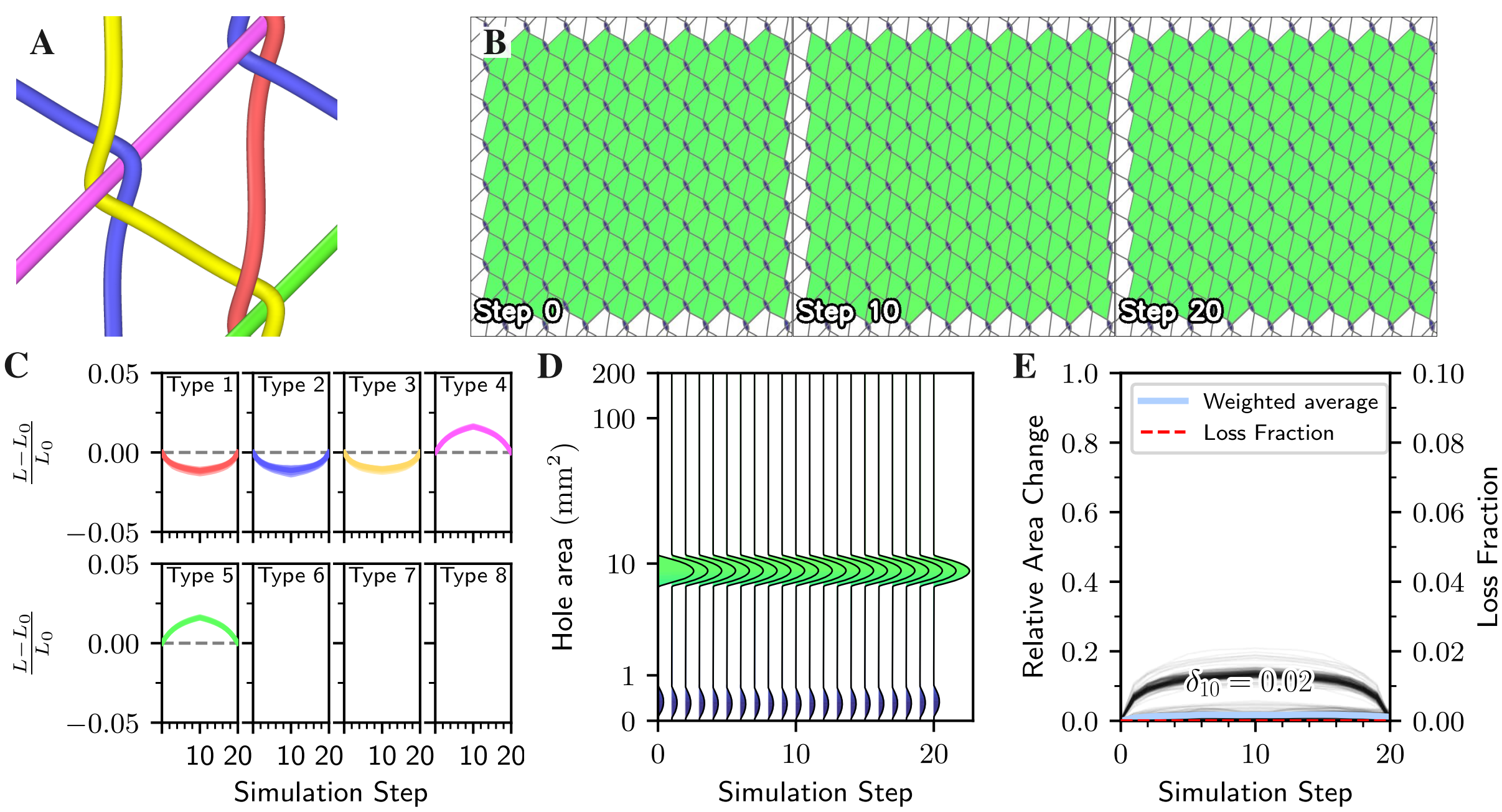

Figure S83: **Simulation of lace structure Diamond-7. A** Unit-cell schematic (active bond types {1, 2, 3, 4, 5}). **B** Snapshots at steps 0, 10, and 20, showing the initial structure, maximum deformation (active bonds shrink by 25%), and subsequent relaxation. **C** Variation of the relative length of yarns, grouped by type. **D** Evolution of the distribution of pore areas over time. **E** Relative area change of individual pores.

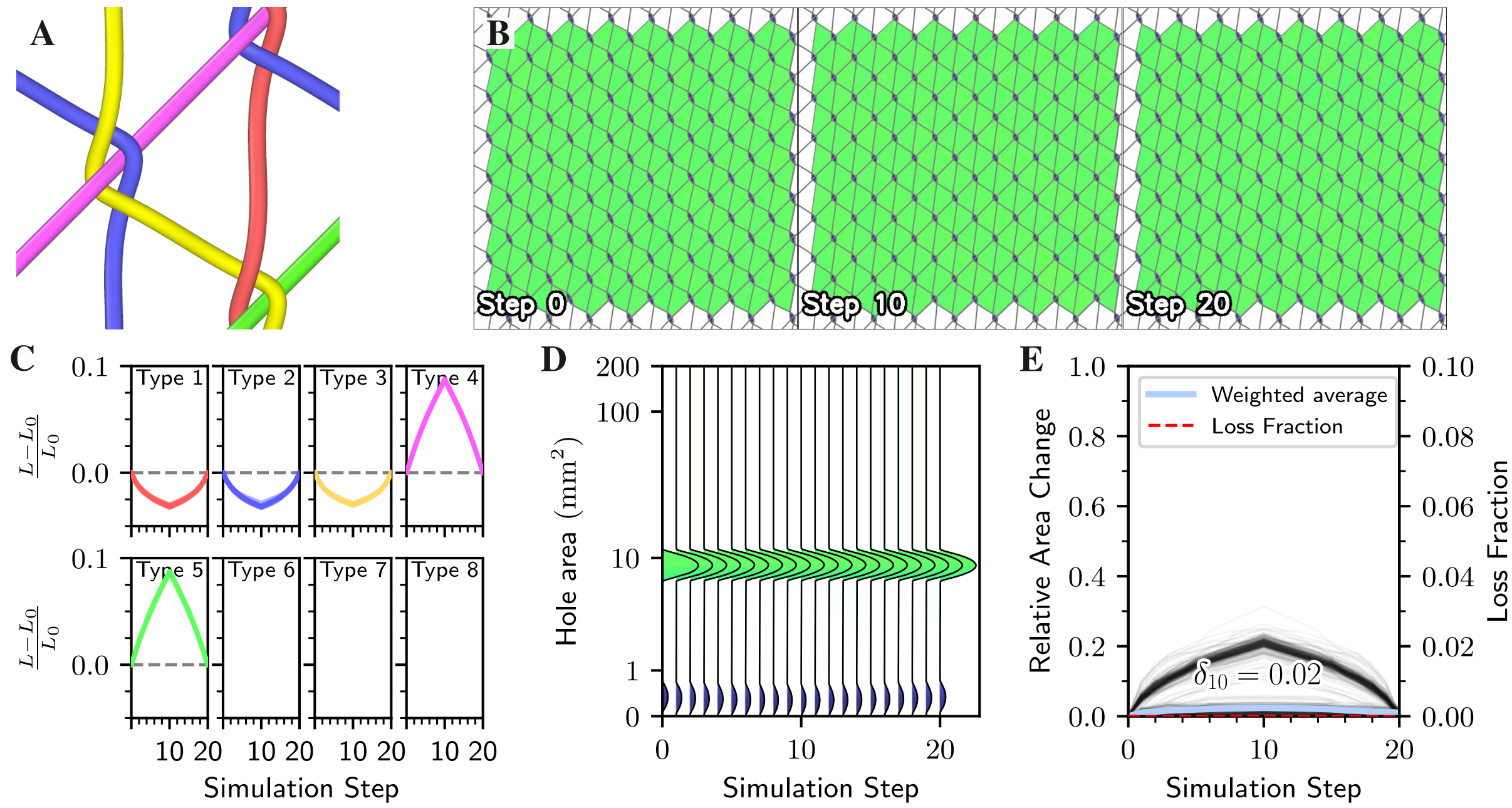

Figure S84: **Simulation of lace structure Diamond-7. A** Unit-cell schematic (active bond types {1, 2, 3}). **B** Snapshots at steps 0, 10, and 20, showing the initial structure, maximum deformation (active bonds shrink by 25%), and subsequent relaxation. **C** Variation of the relative length of yarns, grouped by type. **D** Evolution of the distribution of pore areas over time. **E** Relative area change of individual pores.

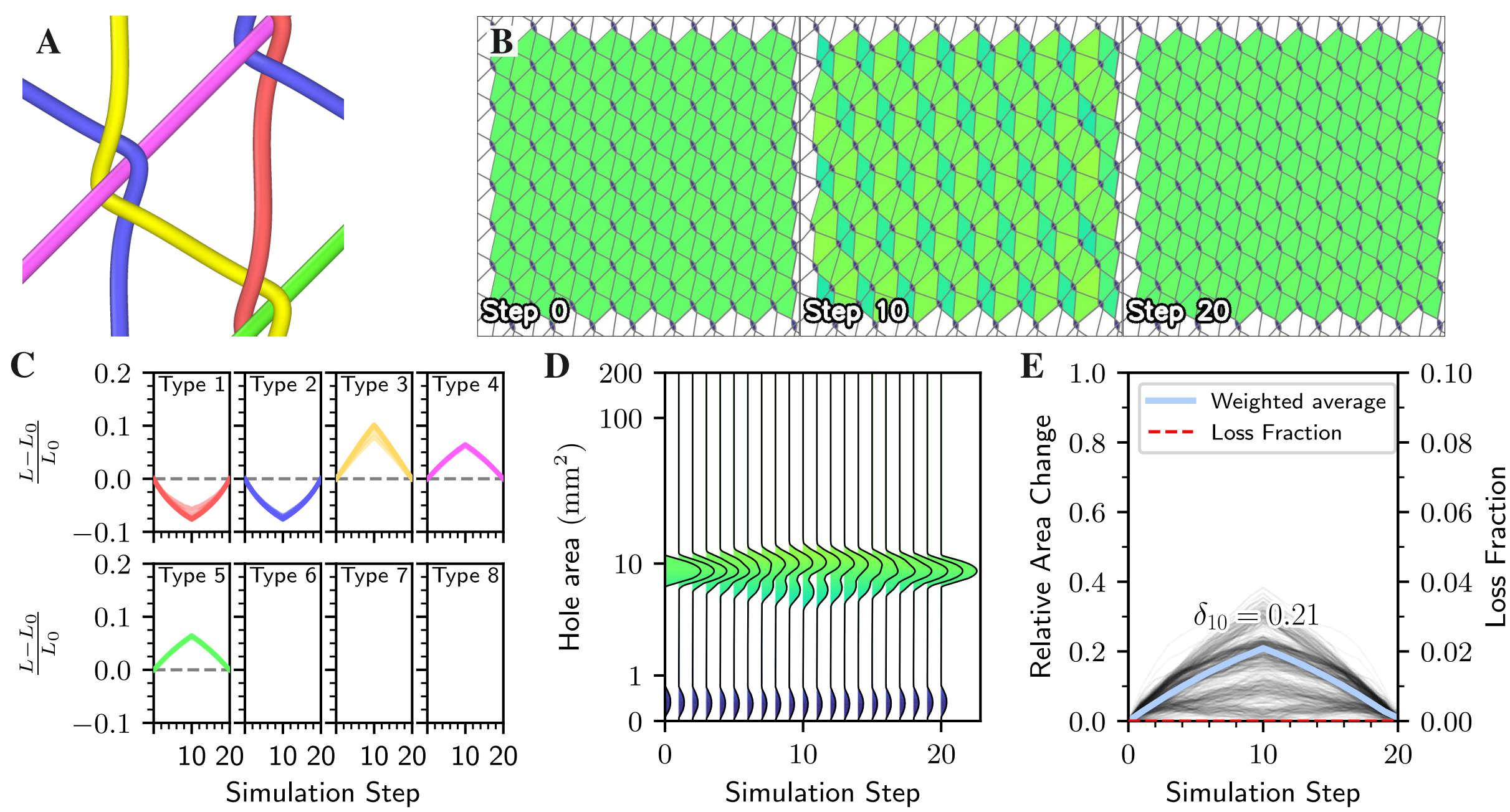

Figure S85: **Simulation of lace structure Diamond-7. A** Unit-cell schematic (active bond types {1, 2}). **B** Snapshots at steps 0, 10, and 20, showing the initial structure, maximum deformation (active bonds shrink by 25%), and subsequent relaxation. **C** Variation of the relative length of yarns, grouped by type. **D** Evolution of the distribution of pore areas over time. **E** Relative area change of individual pores.

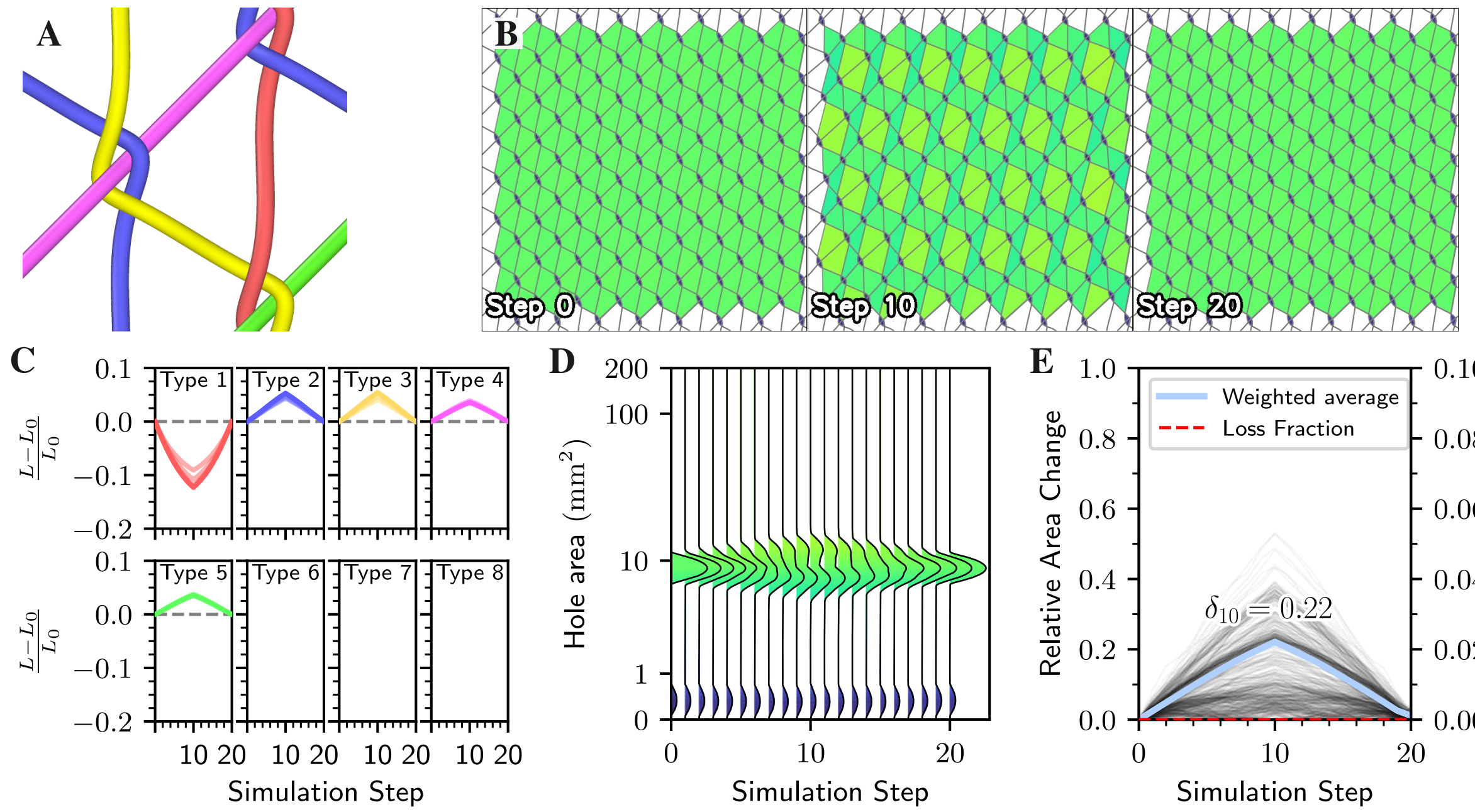

Figure S86: **Simulation of lace structure Diamond-7.** **A** Unit-cell schematic (active bond types {1}). **B** Snapshots at steps 0, 10, and 20, showing the initial structure, maximum deformation (active bonds shrink by 25%), and subsequent relaxation. **C** Variation of the relative length of yarns, grouped by type. **D** Evolution of the distribution of pore areas over time. **E** Relative area change of individual pores.

Square-1

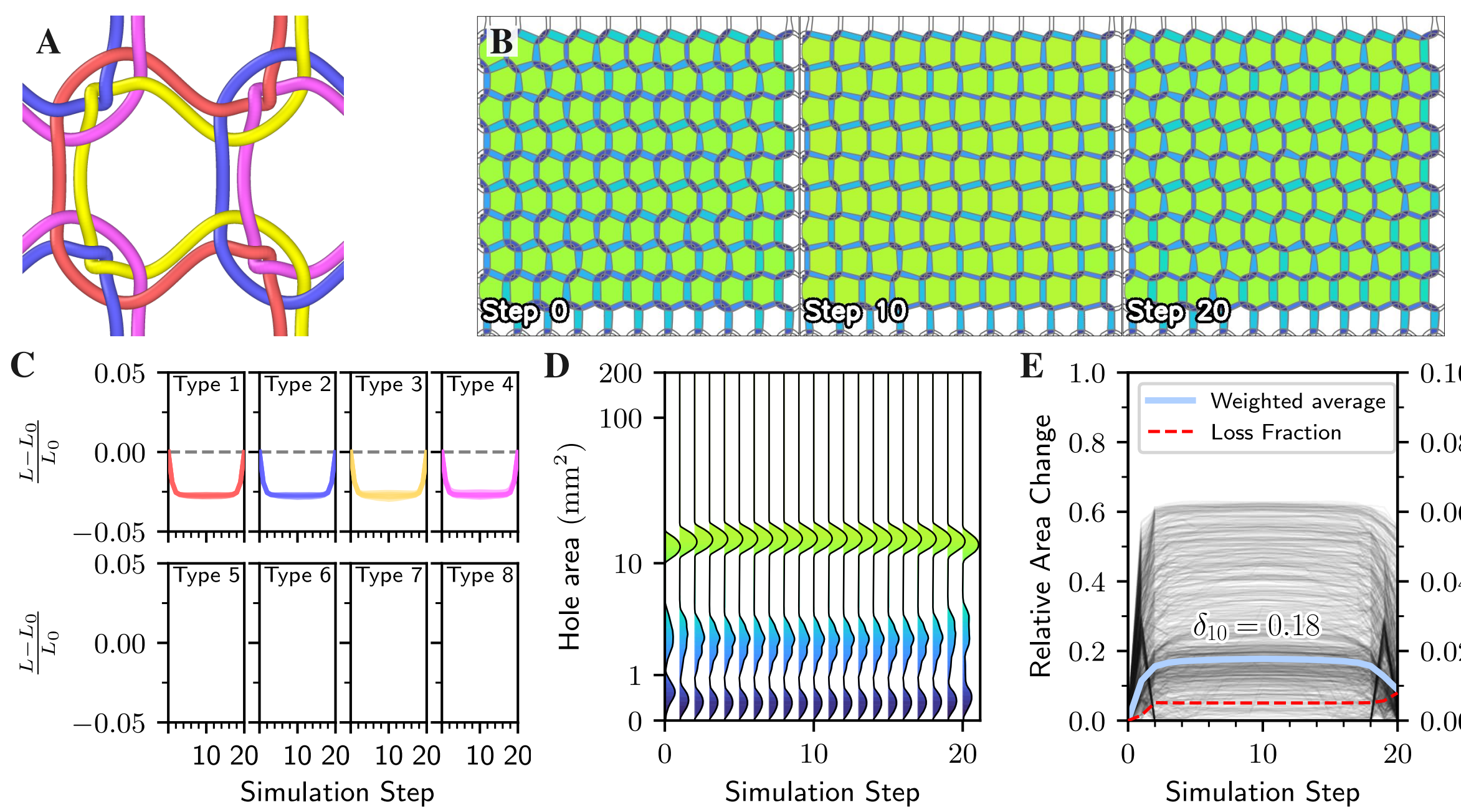

Figure S87: **Simulation of lace structure Square-1.** **A** Unit-cell schematic (active bond types {1, 2, 3, 4}). **B** Snapshots at steps 0, 10, and 20, showing the initial structure, maximum deformation (active bonds shrink by 25%), and subsequent relaxation. **C** Variation of the relative length of yarns, grouped by type. **D** Evolution of the distribution of pore areas over time. **E** Relative area change of individual pores.

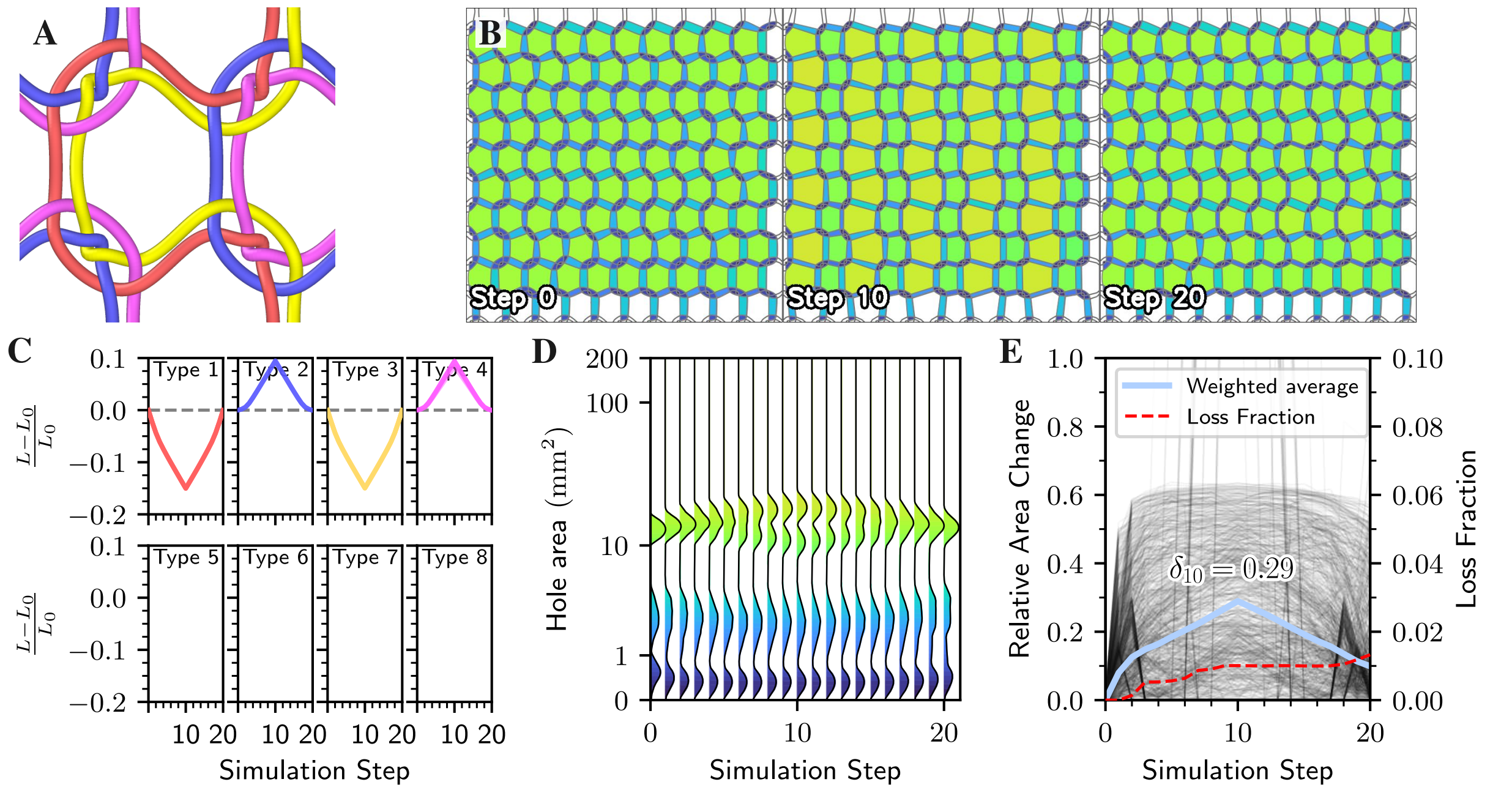

Figure S88: **Simulation of lace structure Square-1.** **A** Unit-cell schematic (active bond types {1, 3}). **B** Snapshots at steps 0, 10, and 20, showing the initial structure, maximum deformation (active bonds shrink by 25%), and subsequent relaxation. **C** Variation of the relative length of yarns, grouped by type. **D** Evolution of the distribution of pore areas over time. **E** Relative area change of individual pores.

Square-2

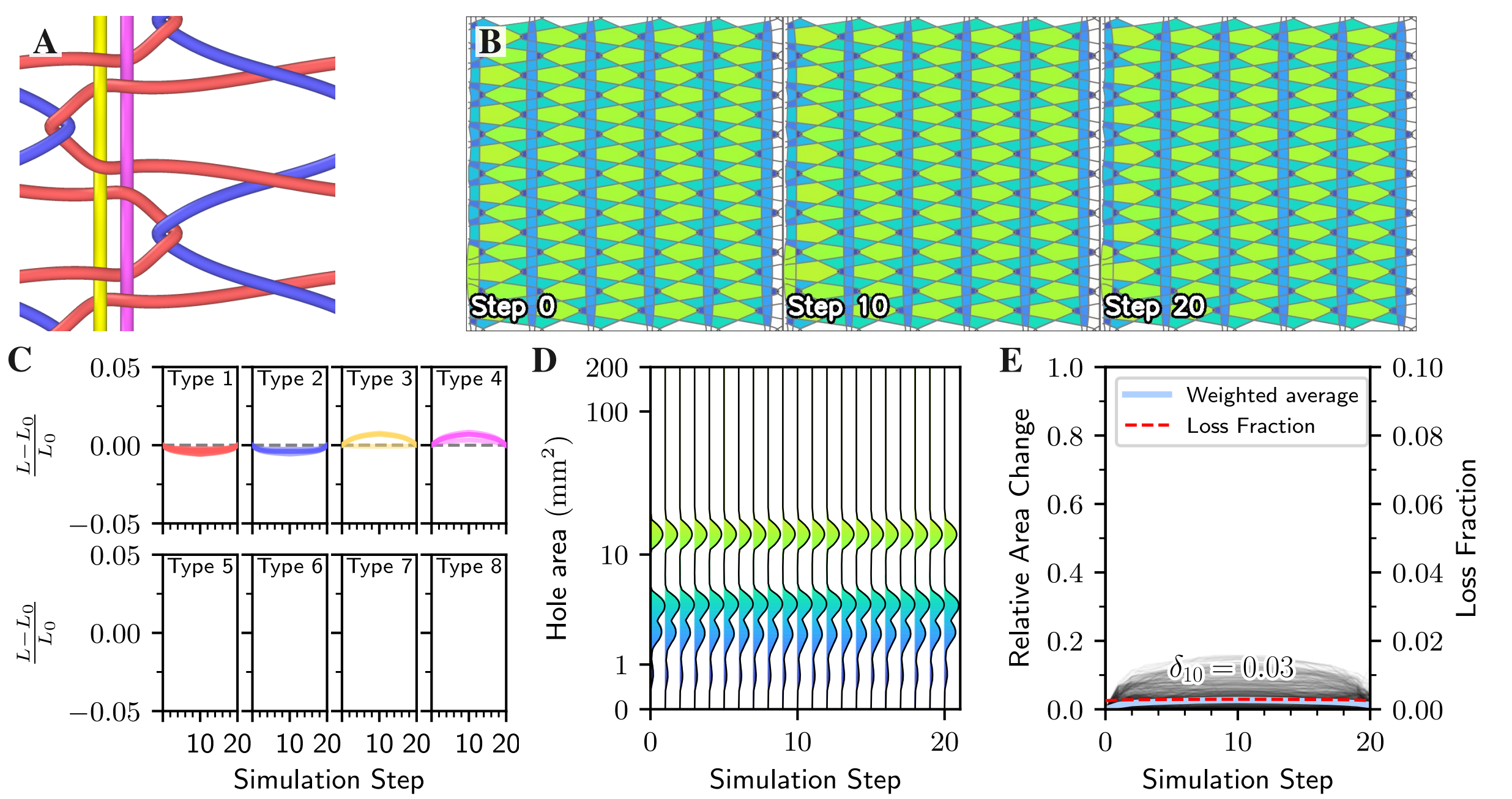

Figure S89: **Simulation of lace structure Square-2.** **A** Unit-cell schematic (active bond types {1, 2, 3, 4}). **B** Snapshots at steps 0, 10, and 20, showing the initial structure, maximum deformation (active bonds shrink by 25%), and subsequent relaxation. **C** Variation of the relative length of yarns, grouped by type. **D** Evolution of the distribution of pore areas over time. **E** Relative area change of individual pores.

Hexa-1

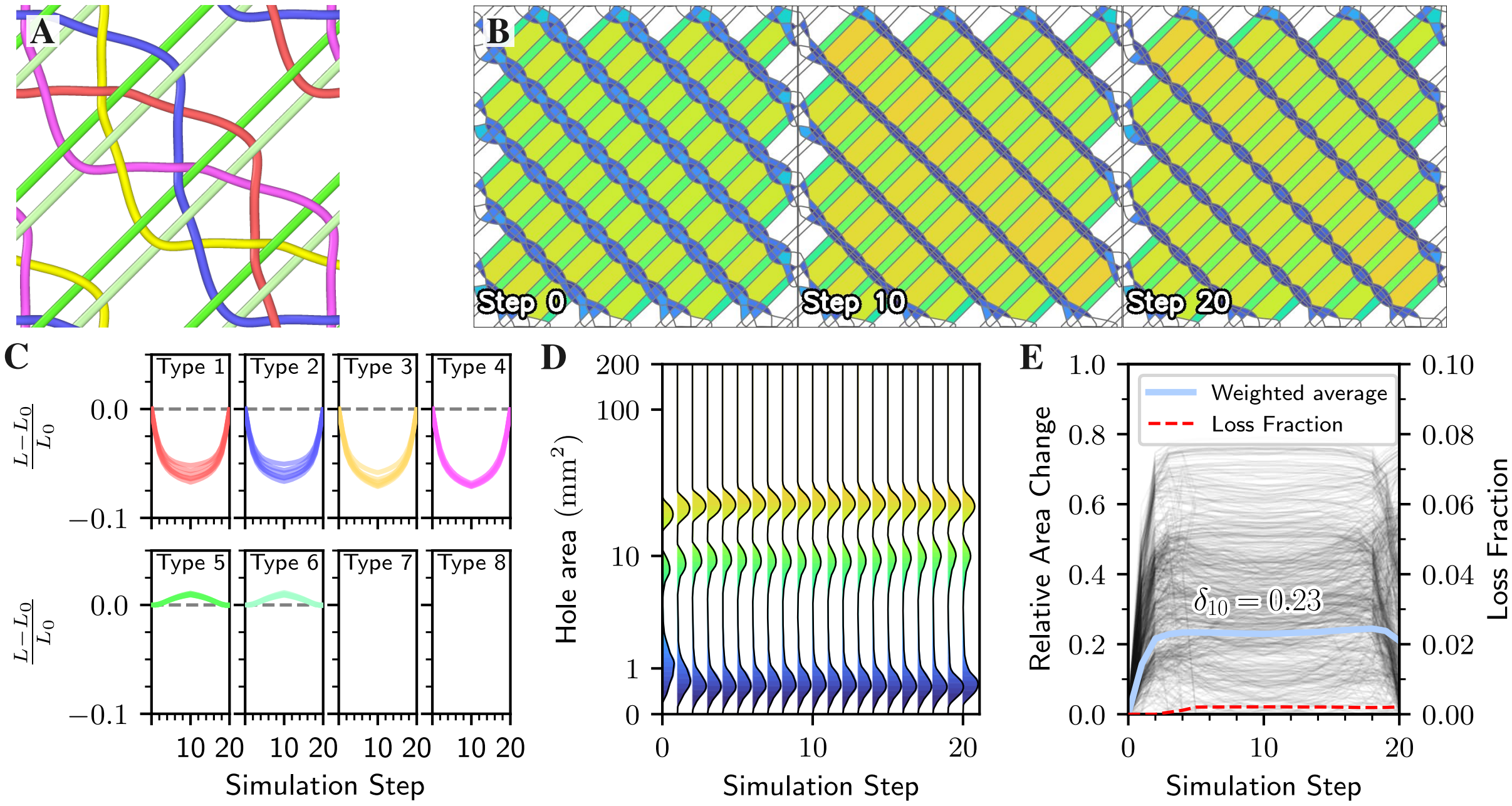

Figure S90: **Simulation of lace structure Hexa-1.** **A** Unit-cell schematic (active bond types {1, 2, 3, 4, 5, 6}). **B** Snapshots at steps 0, 10, and 20, showing the initial structure, maximum deformation (active bonds shrink by 25%), and subsequent relaxation. **C** Variation of the relative length of yarns, grouped by type. **D** Evolution of the distribution of pore areas over time. **E** Relative area change of individual pores.

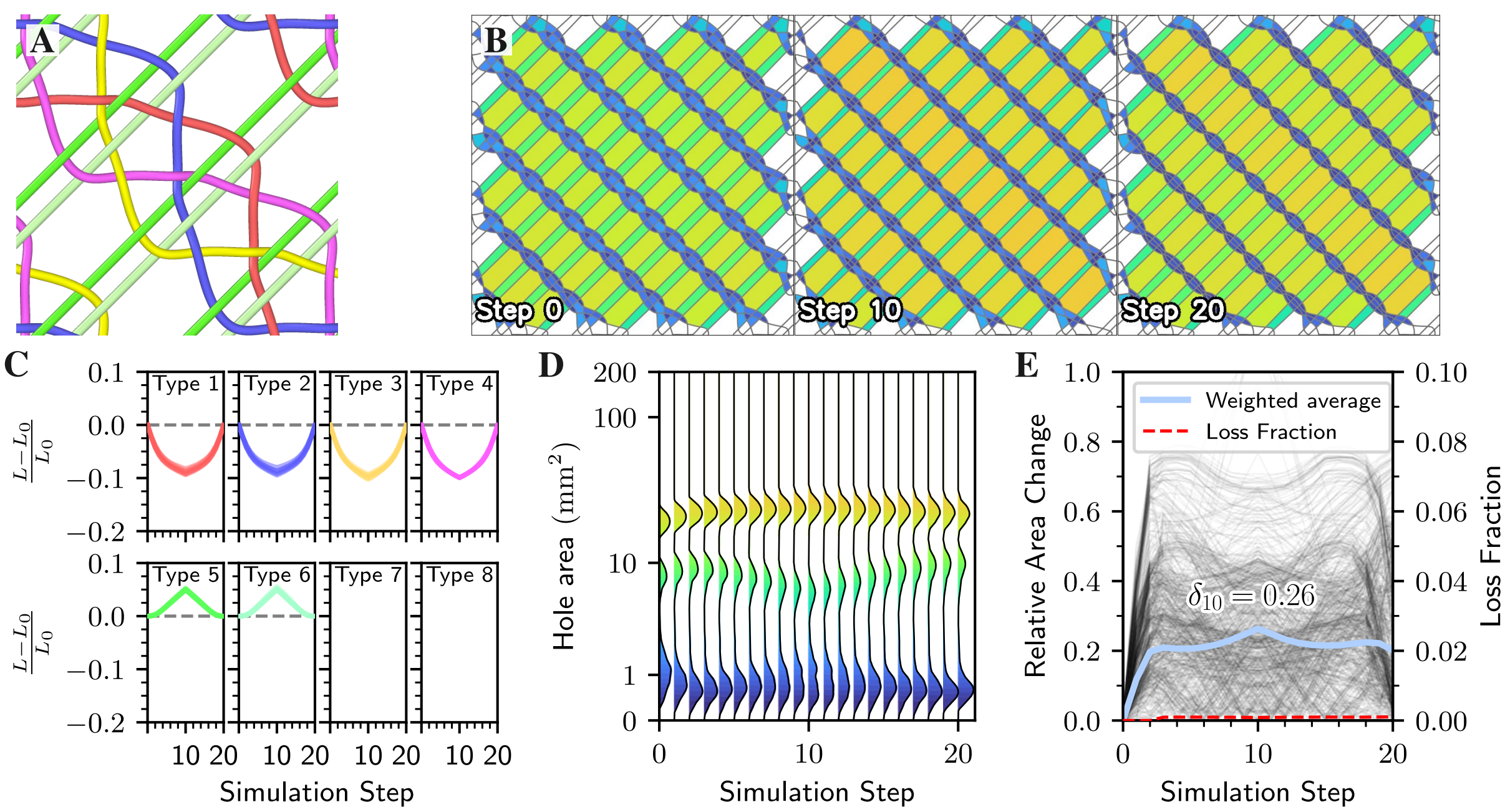

Figure S91: **Simulation of lace structure Hexa-1.** **A** Unit-cell schematic (active bond types {1, 2, 3, 4}). **B** Snapshots at steps 0, 10, and 20, showing the initial structure, maximum deformation (active bonds shrink by 25%), and subsequent relaxation. **C** Variation of the relative length of yarns, grouped by type. **D** Evolution of the distribution of pore areas over time. **E** Relative area change of individual pores.

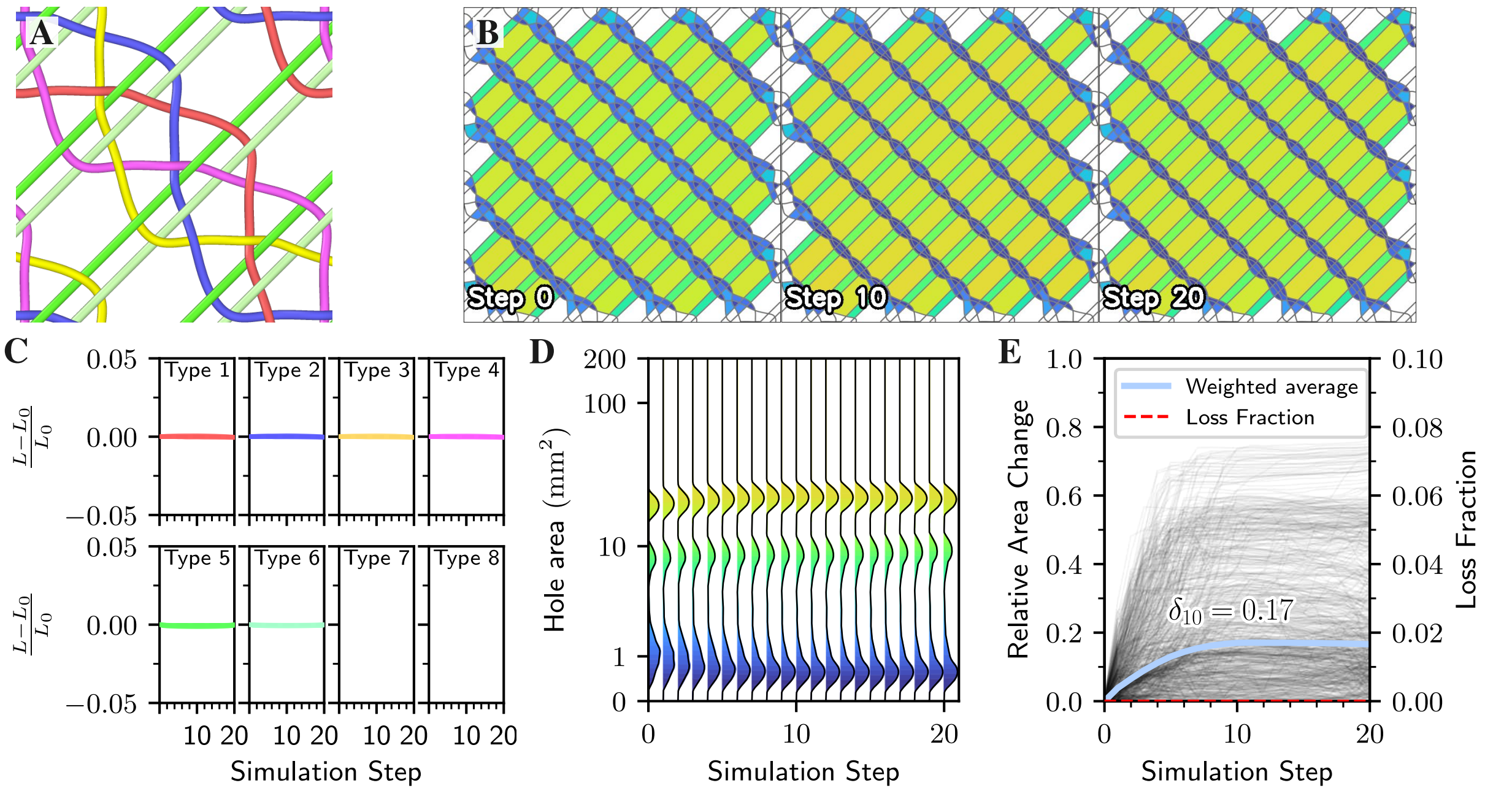

Figure S92: **Simulation of lace structure Hexa-1. A** Unit-cell schematic (active bond types {5, 6}). **B** Snapshots at steps 0, 10, and 20, showing the initial structure, maximum deformation (active bonds shrink by 25%), and subsequent relaxation. **C** Variation of the relative length of yarns, grouped by type. **D** Evolution of the distribution of pore areas over time. **E** Relative area change of individual pores.

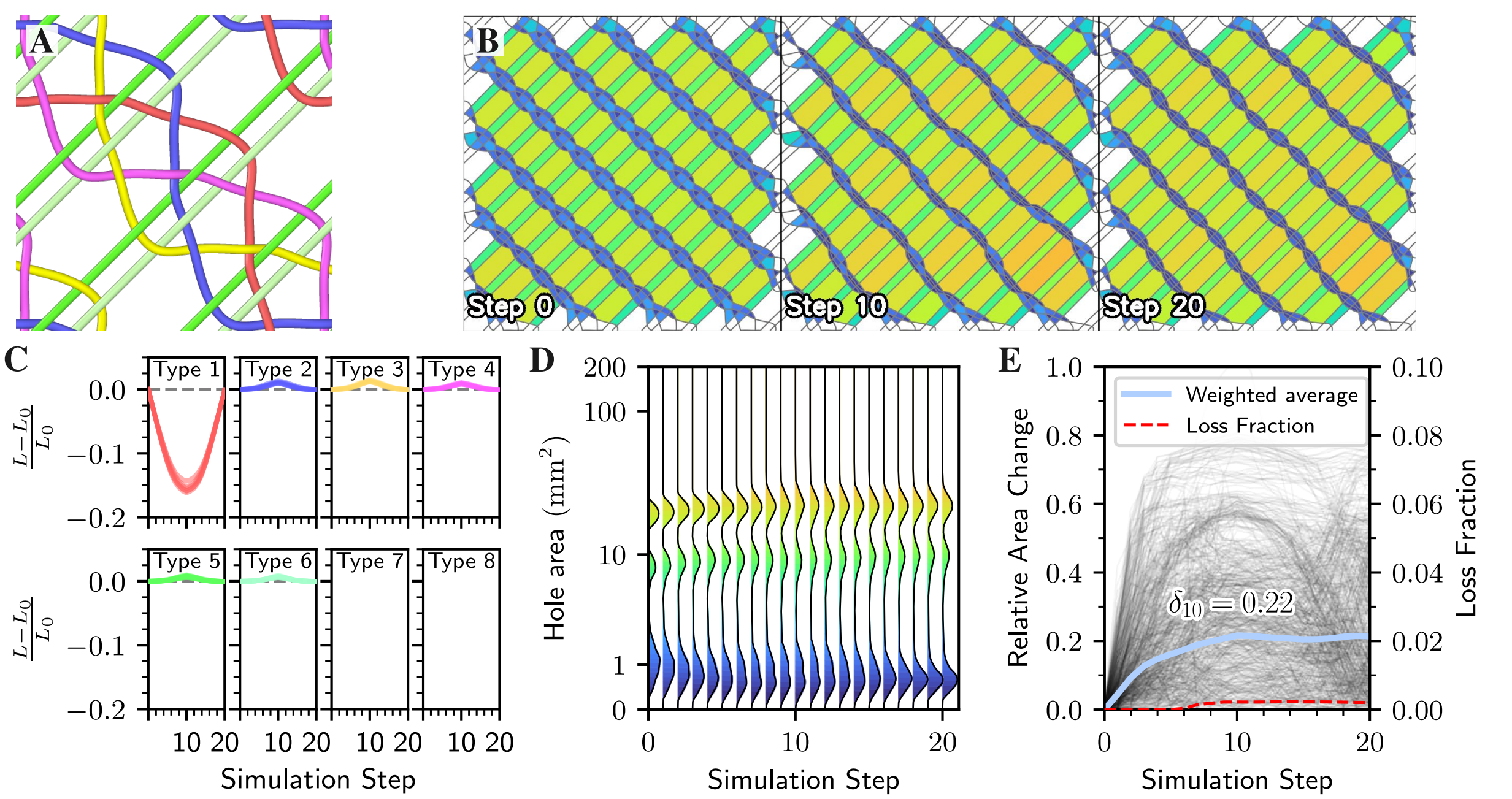

Figure S93: **Simulation of lace structure Hexa-1. A** Unit-cell schematic (active bond types {1}). **B** Snapshots at steps 0, 10, and 20, showing the initial structure, maximum deformation (active bonds shrink by 25%), and subsequent relaxation. **C** Variation of the relative length of yarns, grouped by type. **D** Evolution of the distribution of pore areas over time. **E** Relative area change of individual pores.

Rose-1

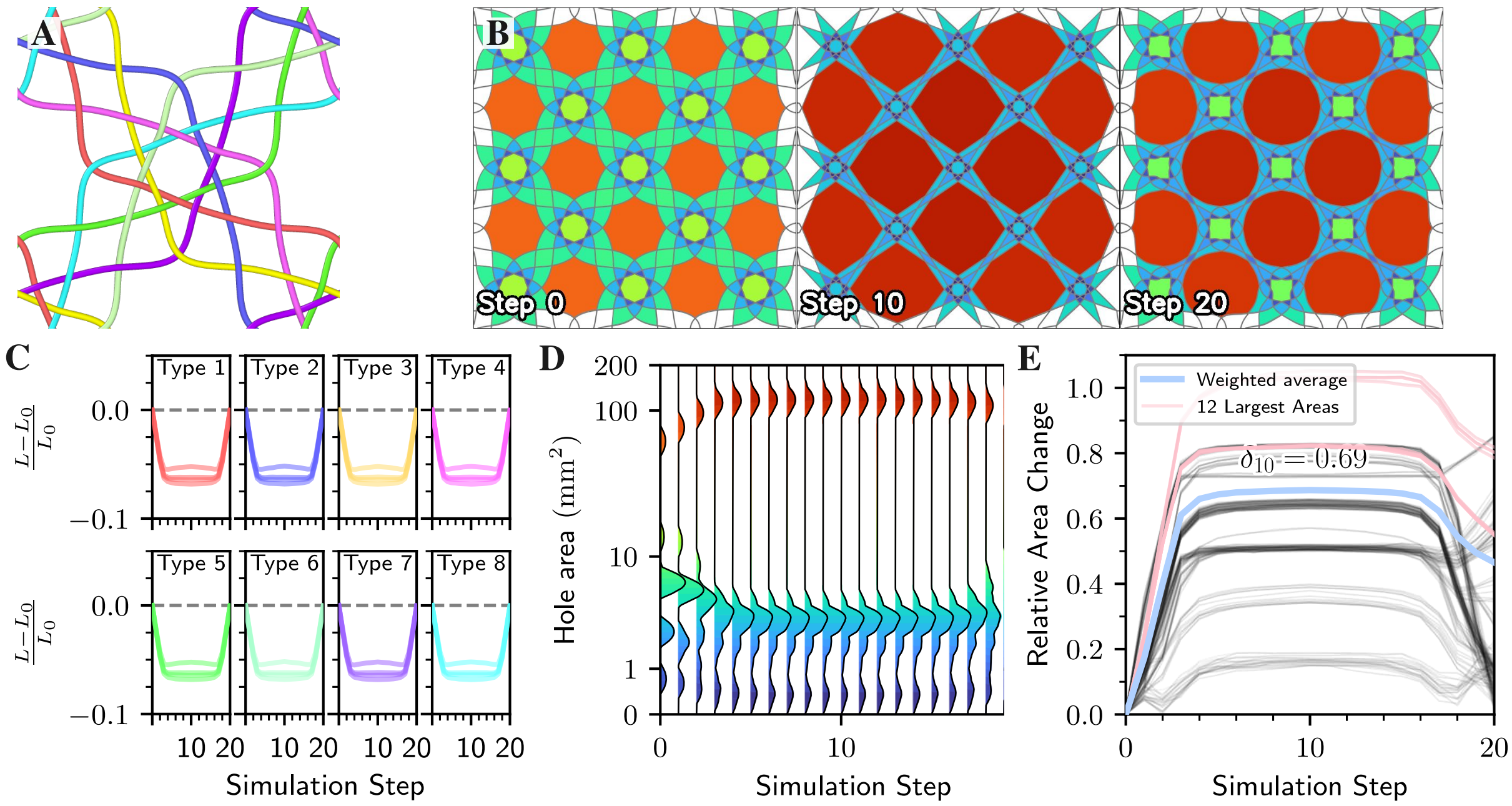

Figure S94: **Simulation of lace structure Rose-1. A** Unit-cell schematic (active bond types {1, 2, 3, 4, 5, 6, 7, 8}). **B** Snapshots at steps 0, 10, and 20, showing the initial structure, maximum deformation (active bonds shrink by 25%), and subsequent relaxation. **C** Variation of the relative length of yarns, grouped by type. **D** Evolution of the distribution of pore areas over time. **E** Relative area change of individual pores.

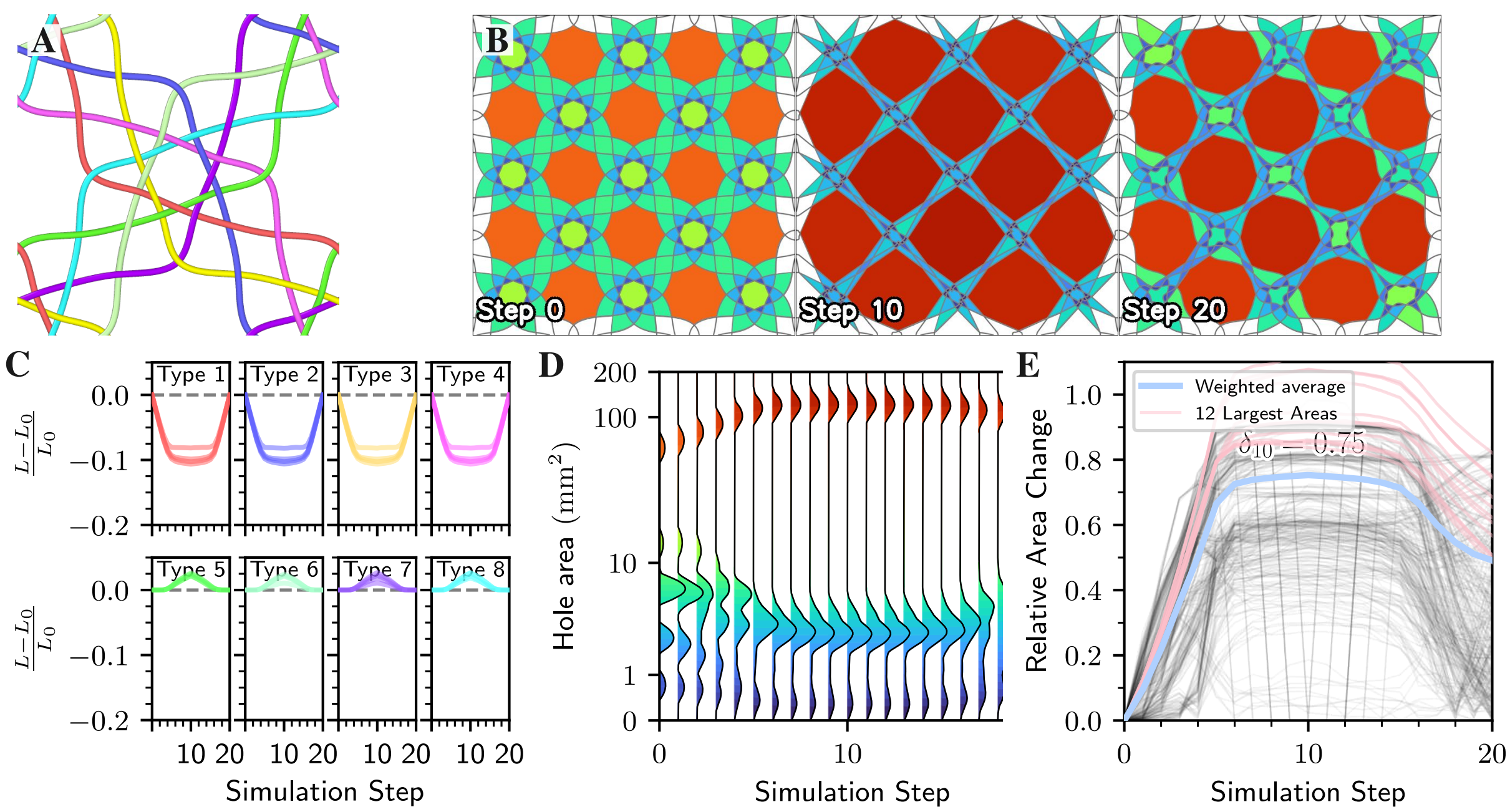

Figure S95: **Simulation of lace structure Rose-1. A** Unit-cell schematic (active bond types {1, 2, 3, 4}). **B** Snapshots at steps 0, 10, and 20, showing the initial structure, maximum deformation (active bonds shrink by 25%), and subsequent relaxation. **C** Variation of the relative length of yarns, grouped by type. **D** Evolution of the distribution of pore areas over time. **E** Relative area change of individual pores.

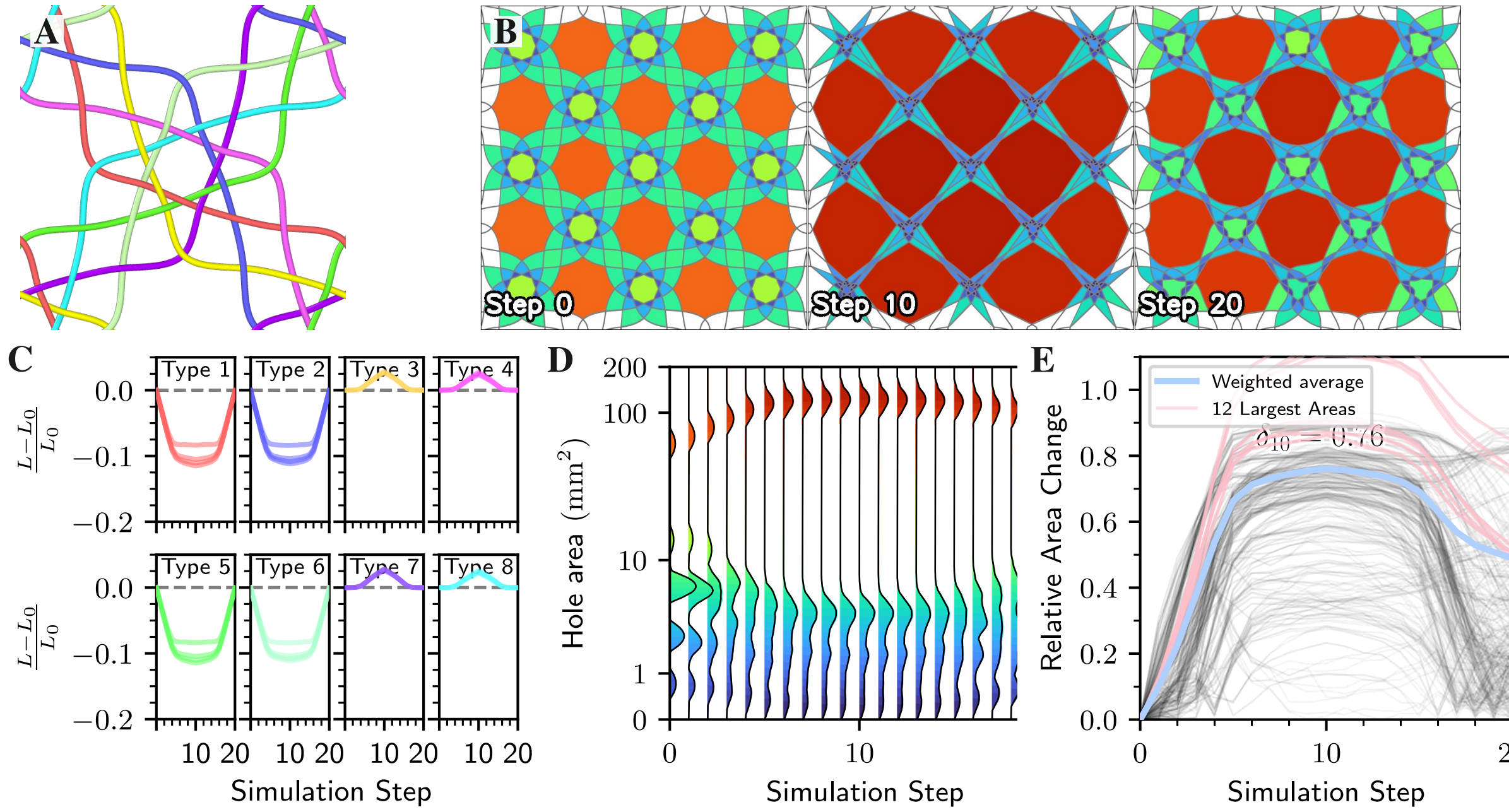

Figure S96: **Simulation of lace structure Rose-1.** **A** Unit-cell schematic (active bond types {1, 2, 5, 6}). **B** Snapshots at steps 0, 10, and 20, showing the initial structure, maximum deformation (active bonds shrink by 25%), and subsequent relaxation. **C** Variation of the relative length of yarns, grouped by type. **D** Evolution of the distribution of pore areas over time. **E** Relative area change of individual pores.

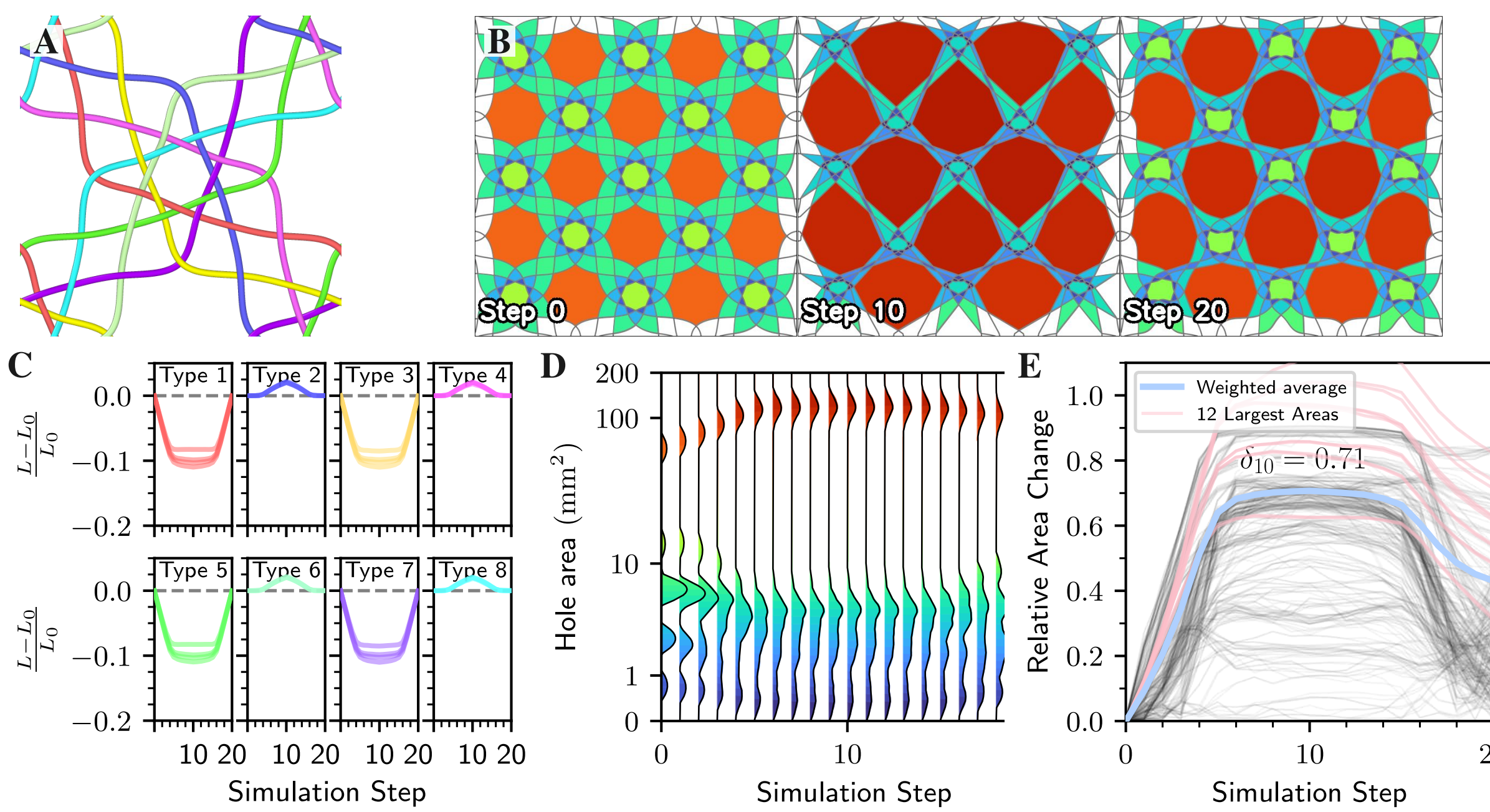

Figure S97: **Simulation of lace structure Rose-1.** **A** Unit-cell schematic (active bond types {1, 3, 5, 7}). **B** Snapshots at steps 0, 10, and 20, showing the initial structure, maximum deformation (active bonds shrink by 25%), and subsequent relaxation. **C** Variation of the relative length of yarns, grouped by type. **D** Evolution of the distribution of pore areas over time. **E** Relative area change of individual pores.

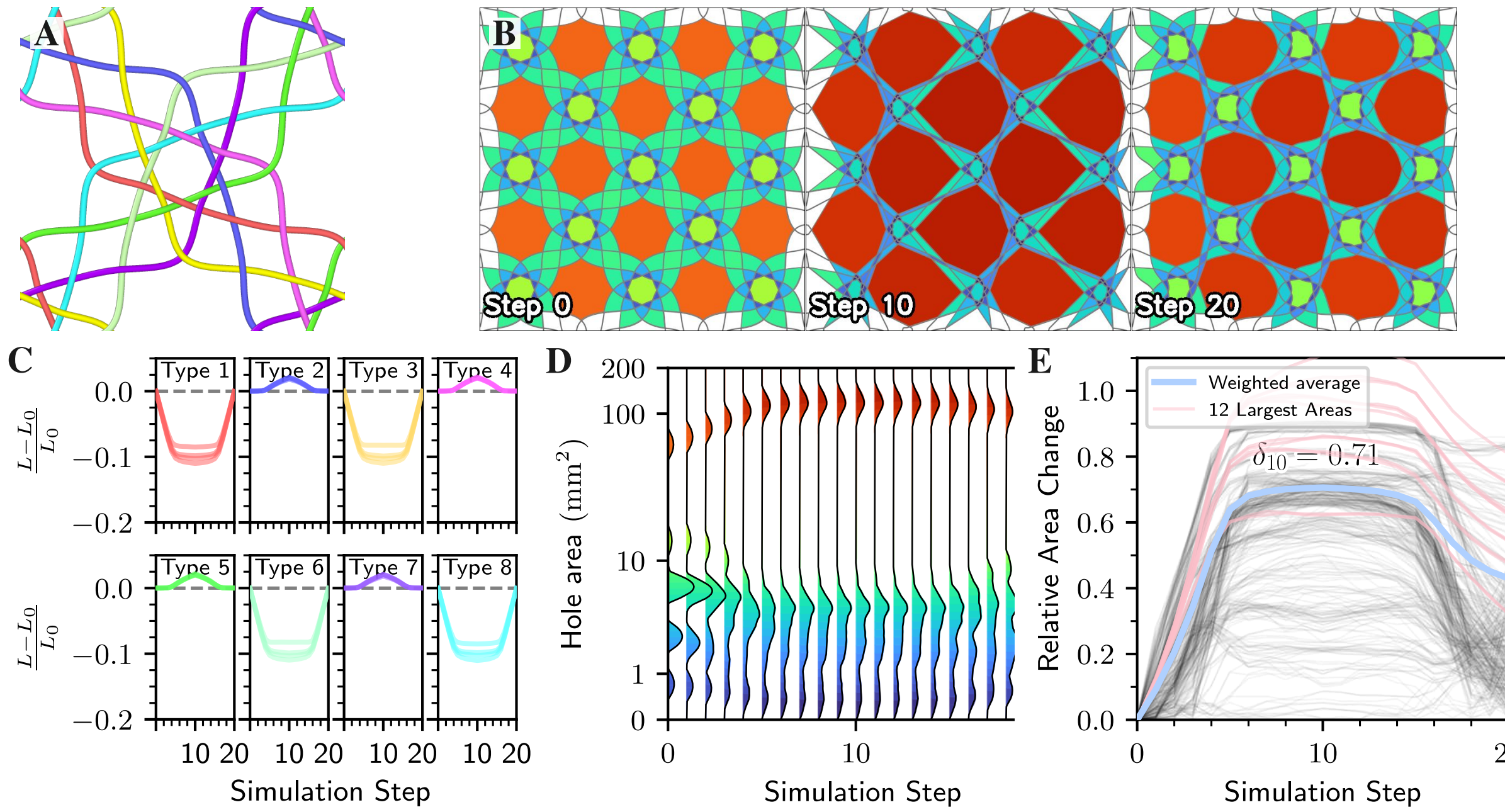

Figure S98: **Simulation of lace structure Rose-1. A** Unit-cell schematic (active bond types {1, 3, 6, 8}). **B** Snapshots at steps 0, 10, and 20, showing the initial structure, maximum deformation (active bonds shrink by 25%), and subsequent relaxation. **C** Variation of the relative length of yarns, grouped by type. **D** Evolution of the distribution of pore areas over time. **E** Relative area change of individual pores.

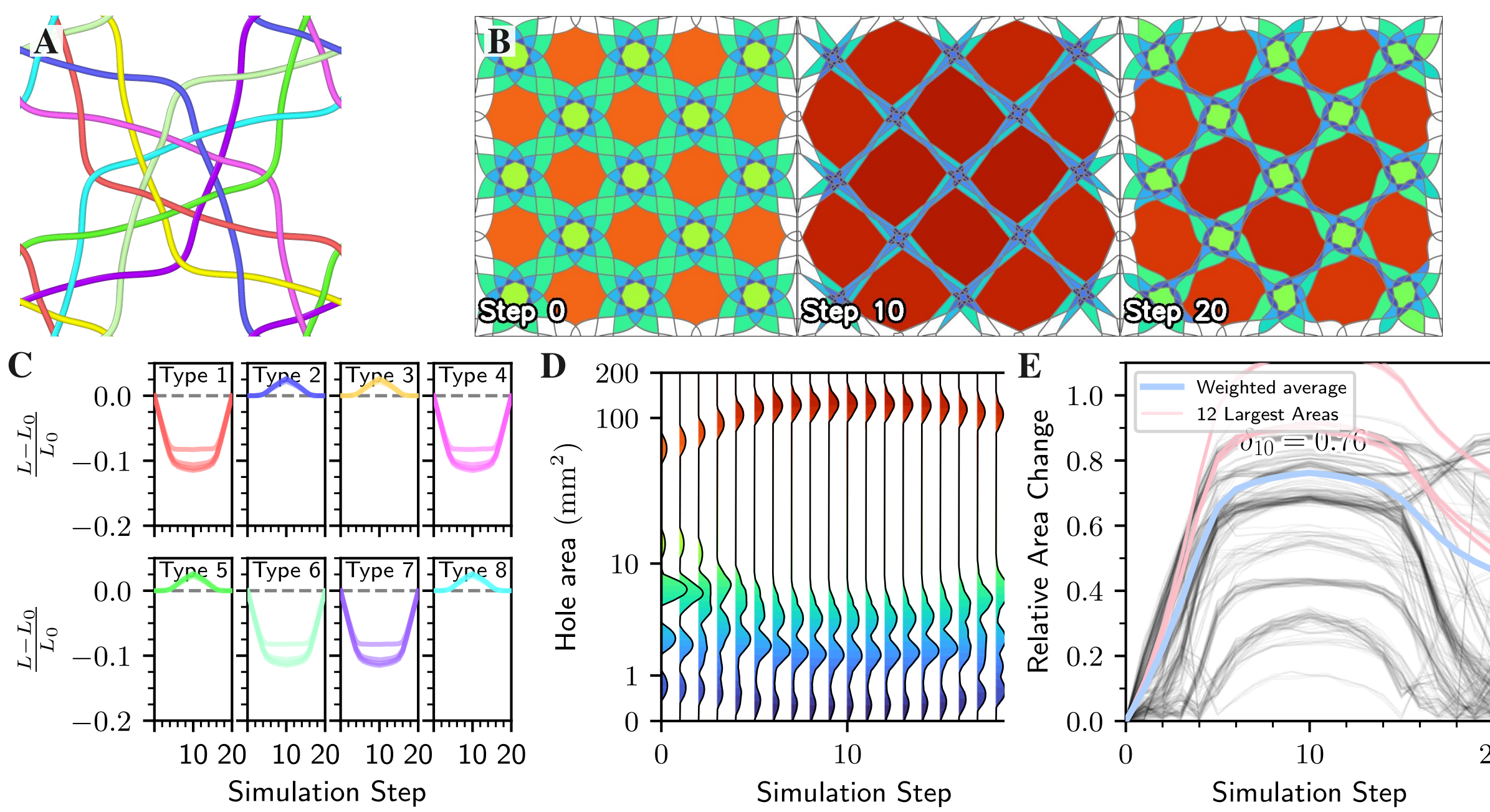

Figure S99: **Simulation of lace structure Rose-1. A** Unit-cell schematic (active bond types {1, 4, 6, 7}). **B** Snapshots at steps 0, 10, and 20, showing the initial structure, maximum deformation (active bonds shrink by 25%), and subsequent relaxation. **C** Variation of the relative length of yarns, grouped by type. **D** Evolution of the distribution of pore areas over time. **E** Relative area change of individual pores.

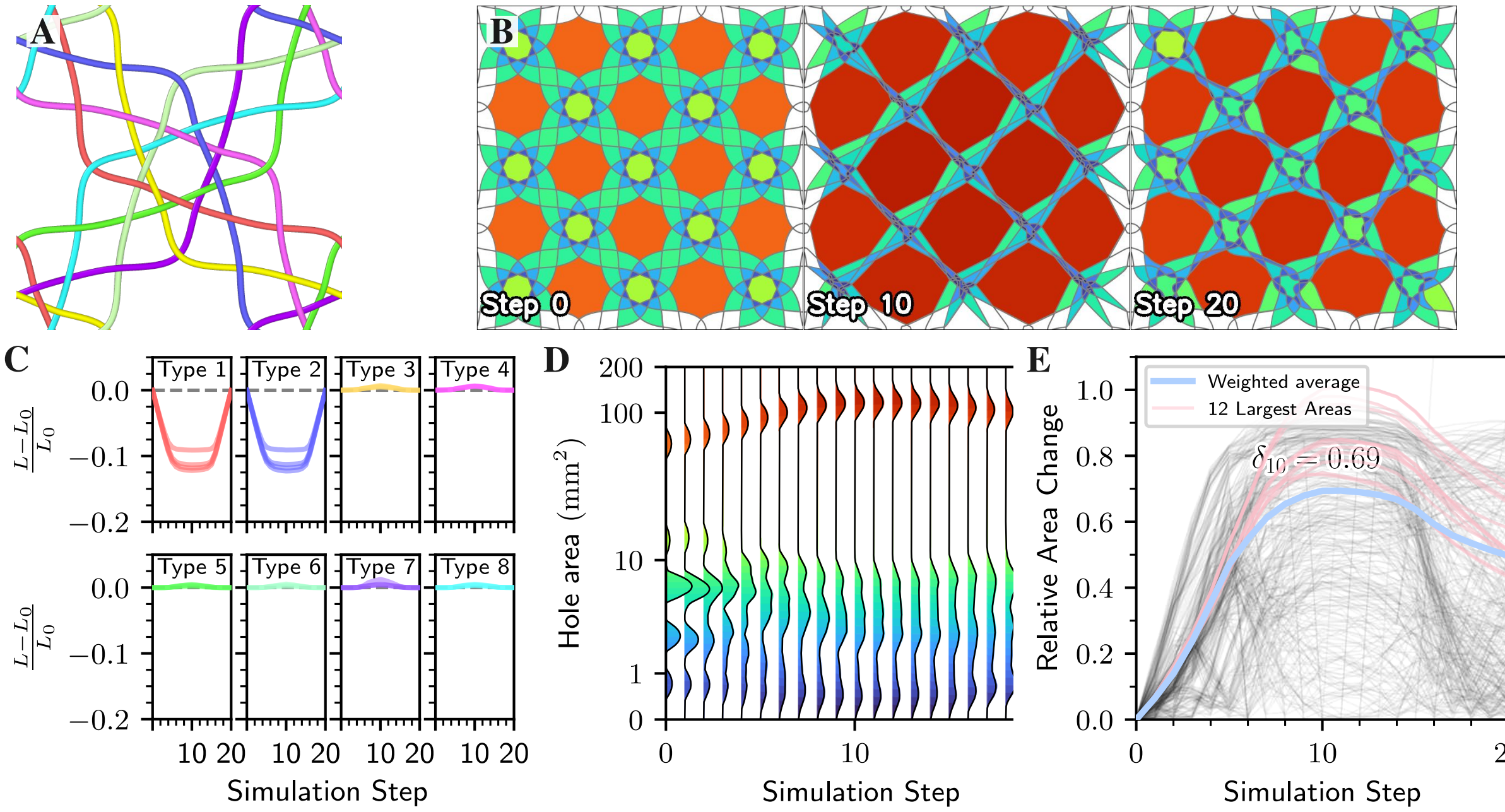

Figure S100: **Simulation of lace structure Rose-1. A** Unit-cell schematic (active bond types {1, 2}). **B** Snapshots at steps 0, 10, and 20, showing the initial structure, maximum deformation (active bonds shrink by 25%), and subsequent relaxation. **C** Variation of the relative length of yarns, grouped by type. **D** Evolution of the distribution of pore areas over time. **E** Relative area change of individual pores.

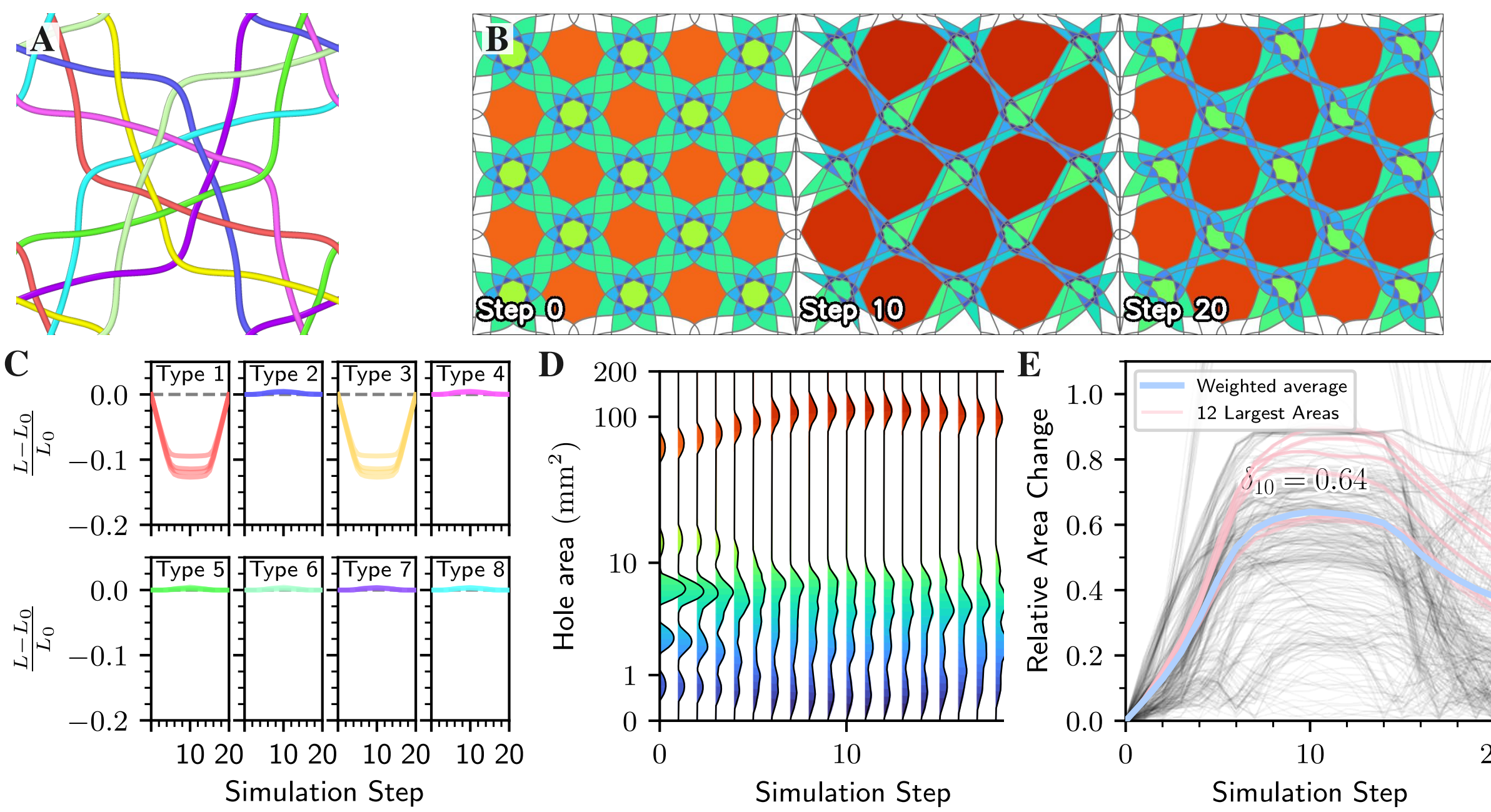

Figure S101: **Simulation of lace structure Rose-1. A** Unit-cell schematic (active bond types {1, 3}). **B** Snapshots at steps 0, 10, and 20, showing the initial structure, maximum deformation (active bonds shrink by 25%), and subsequent relaxation. **C** Variation of the relative length of yarns, grouped by type. **D** Evolution of the distribution of pore areas over time. **E** Relative area change of individual pores.

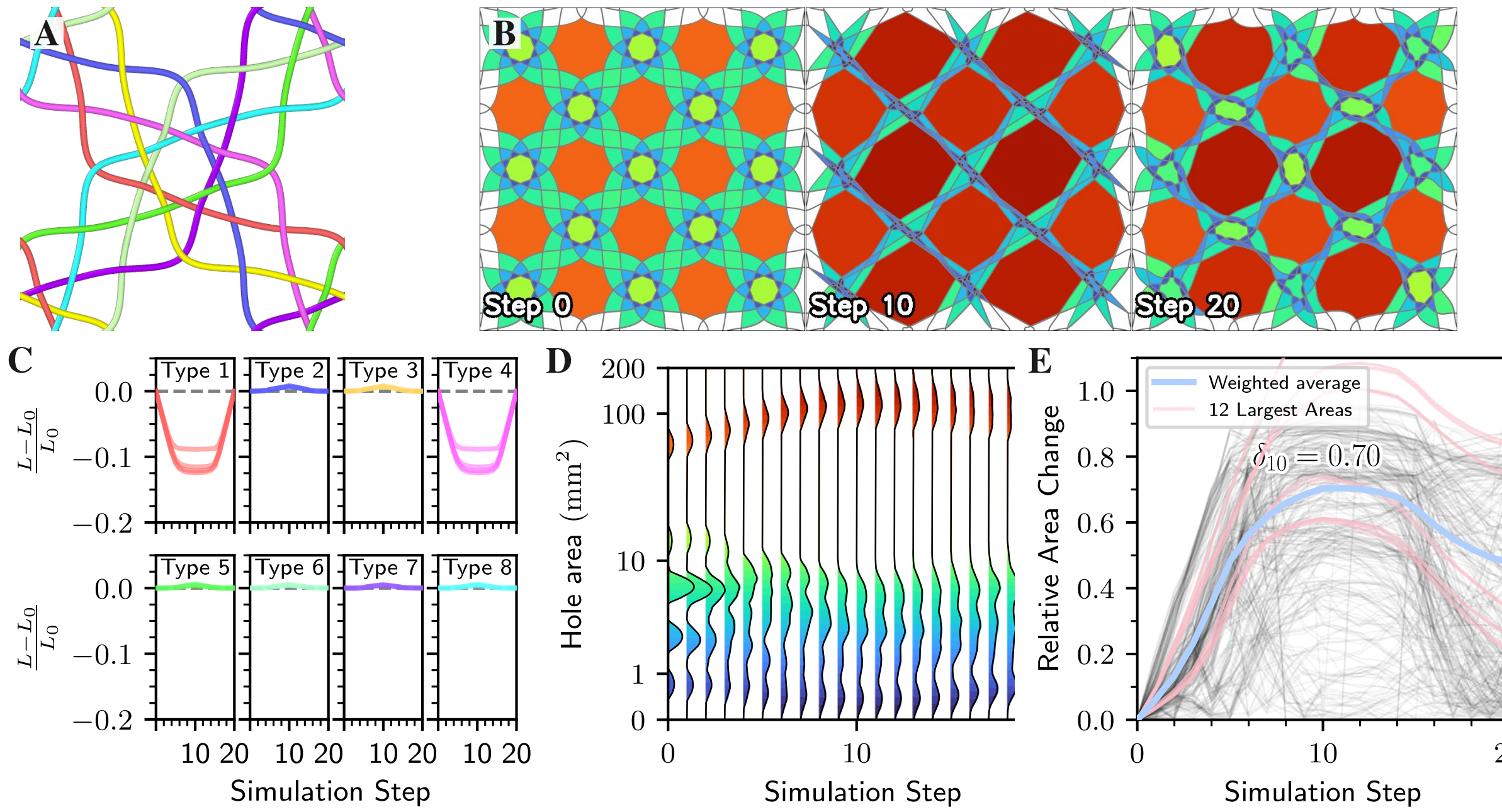

Figure S102: **Simulation of lace structure Rose-1.** **A** Unit-cell schematic (active bond types {1, 4}). **B** Snapshots at steps 0, 10, and 20, showing the initial structure, maximum deformation (active bonds shrink by 25%), and subsequent relaxation. **C** Variation of the relative length of yarns, grouped by type. **D** Evolution of the distribution of pore areas over time. **E** Relative area change of individual pores.

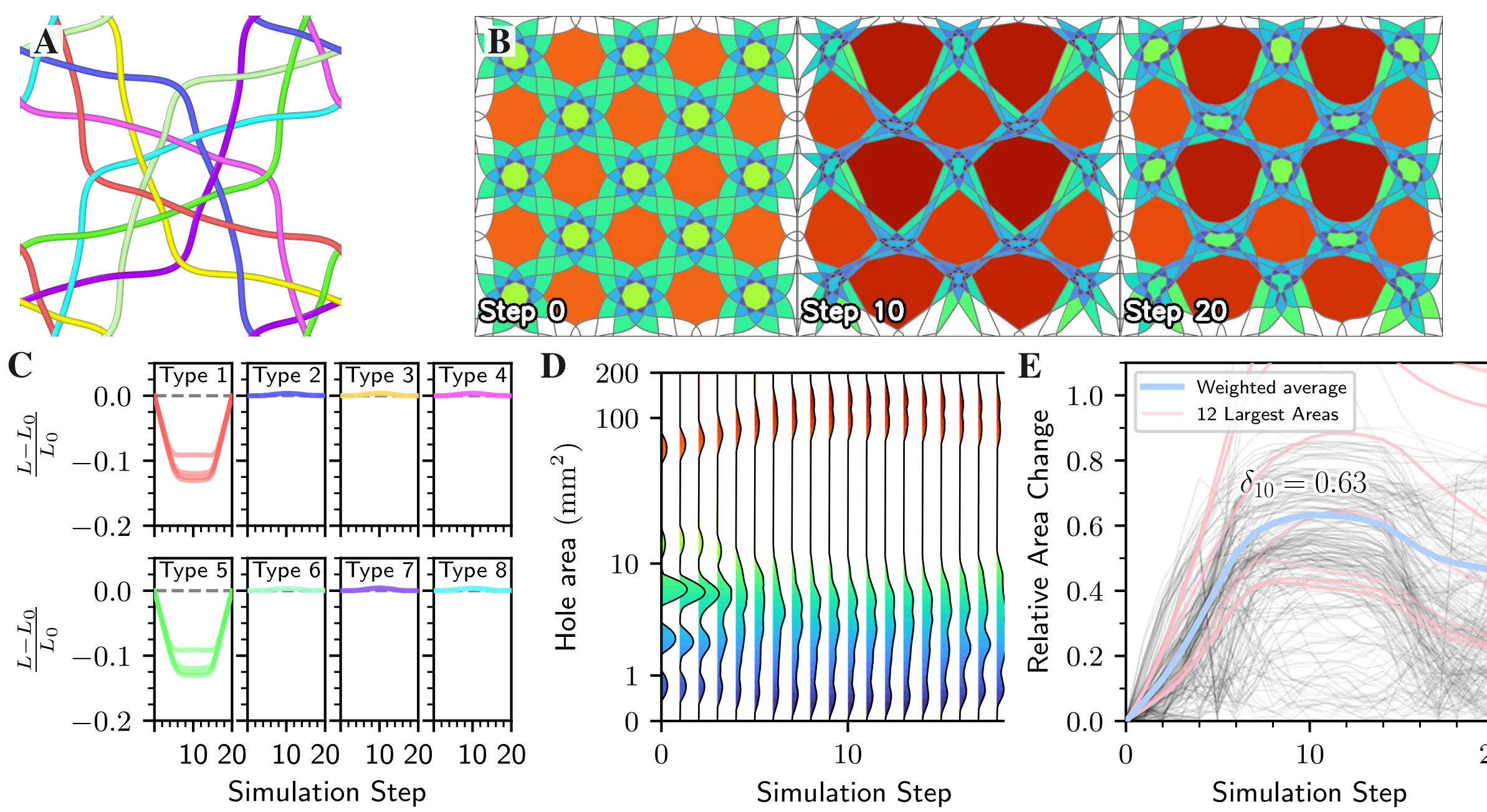

Figure S103: **Simulation of lace structure Rose-1.** **A** Unit-cell schematic (active bond types {1, 5}). **B** Snapshots at steps 0, 10, and 20, showing the initial structure, maximum deformation (active bonds shrink by 25%), and subsequent relaxation. **C** Variation of the relative length of yarns, grouped by type. **D** Evolution of the distribution of pore areas over time. **E** Relative area change of individual pores.

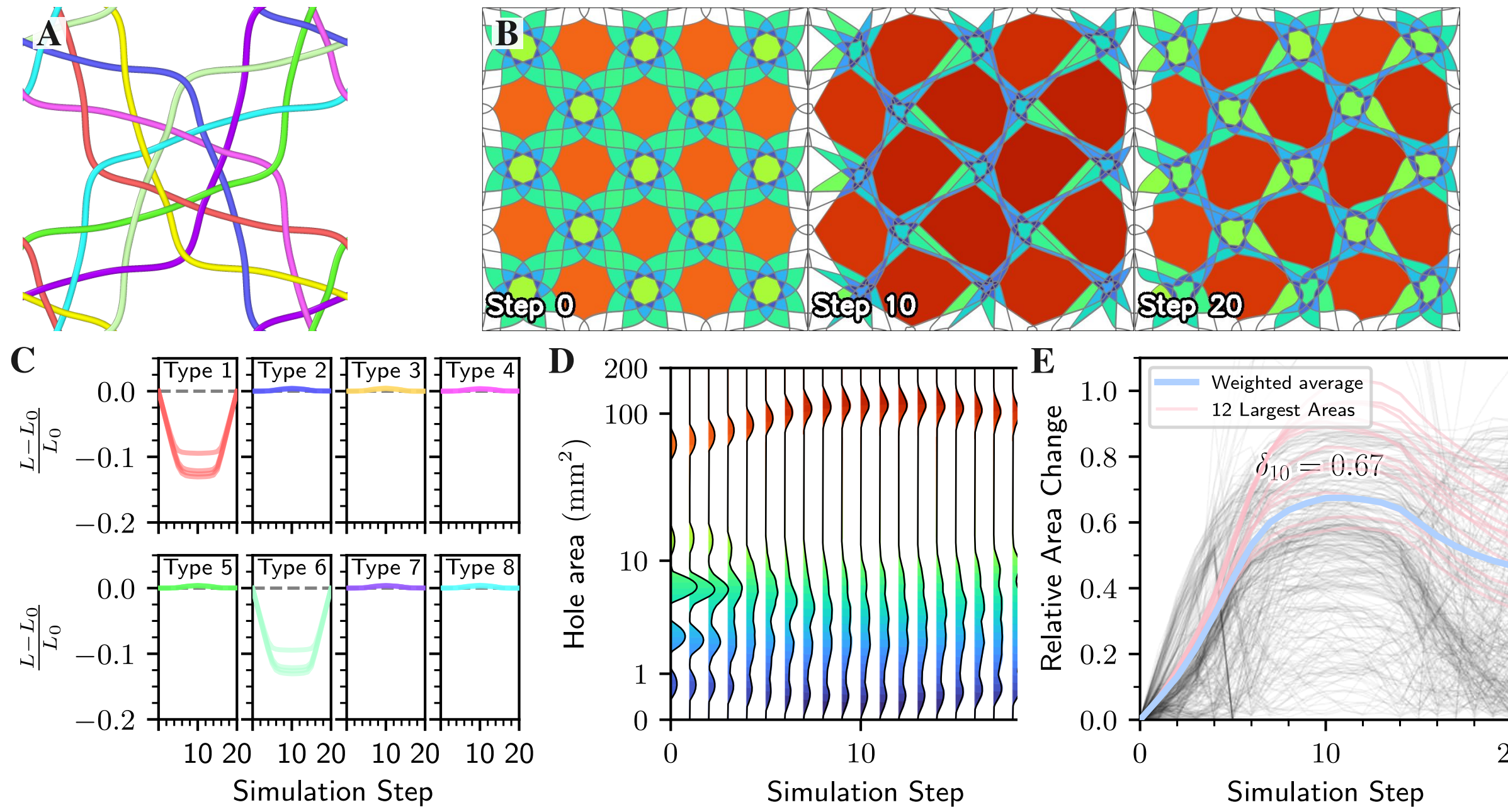

Figure S104: **Simulation of lace structure Rose-1.** **A** Unit-cell schematic (active bond types {1, 6}). **B** Snapshots at steps 0, 10, and 20, showing the initial structure, maximum deformation (active bonds shrink by 25%), and subsequent relaxation. **C** Variation of the relative length of yarns, grouped by type. **D** Evolution of the distribution of pore areas over time. **E** Relative area change of individual pores.

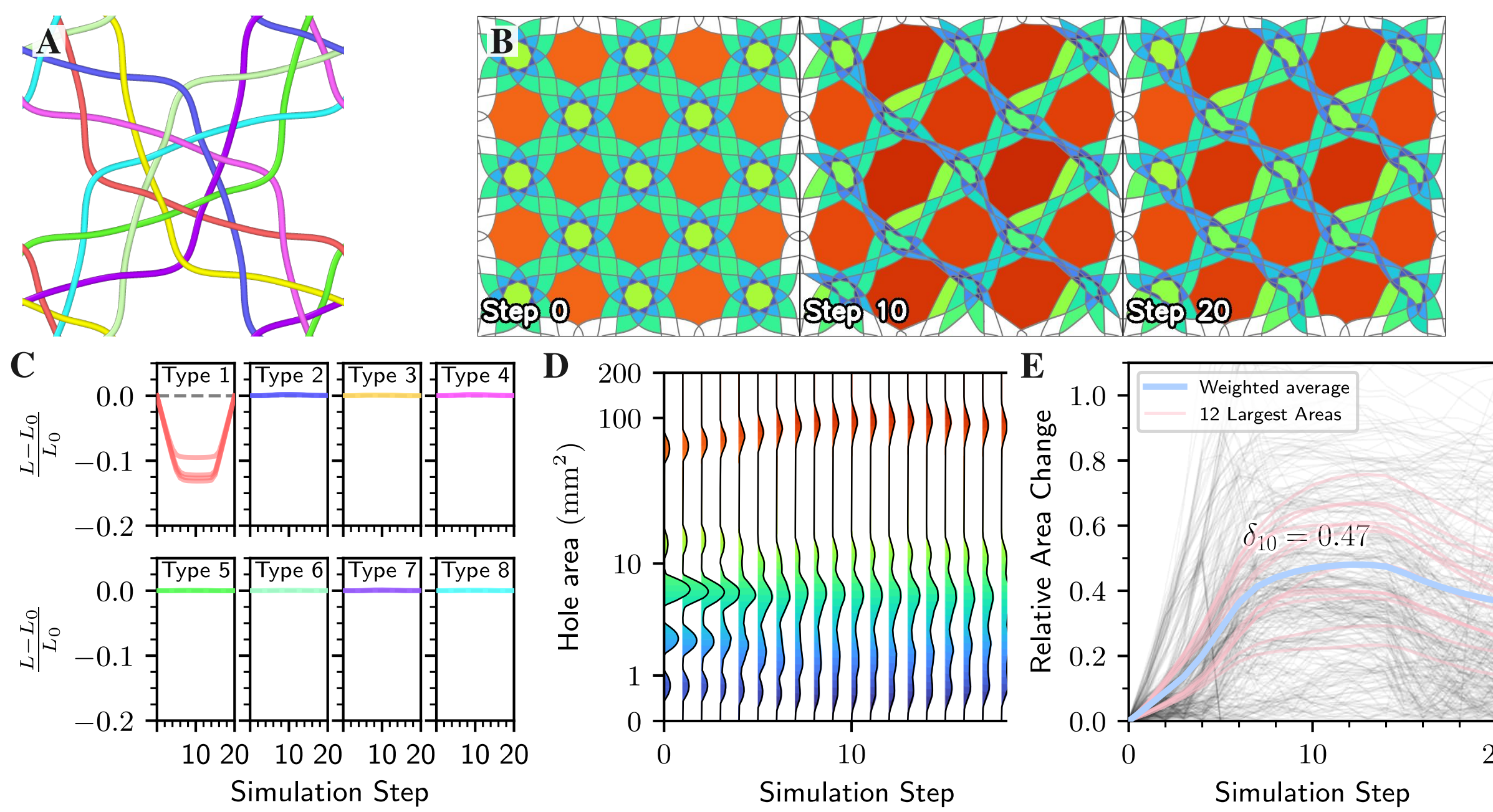

Figure S105: **Simulation of lace structure Rose-1.** **A** Unit-cell schematic (active bond types {1}). **B** Snapshots at steps 0, 10, and 20, showing the initial structure, maximum deformation (active bonds shrink by 25%), and subsequent relaxation. **C** Variation of the relative length of yarns, grouped by type. **D** Evolution of the distribution of pore areas over time. **E** Relative area change of individual pores.

Rose-2

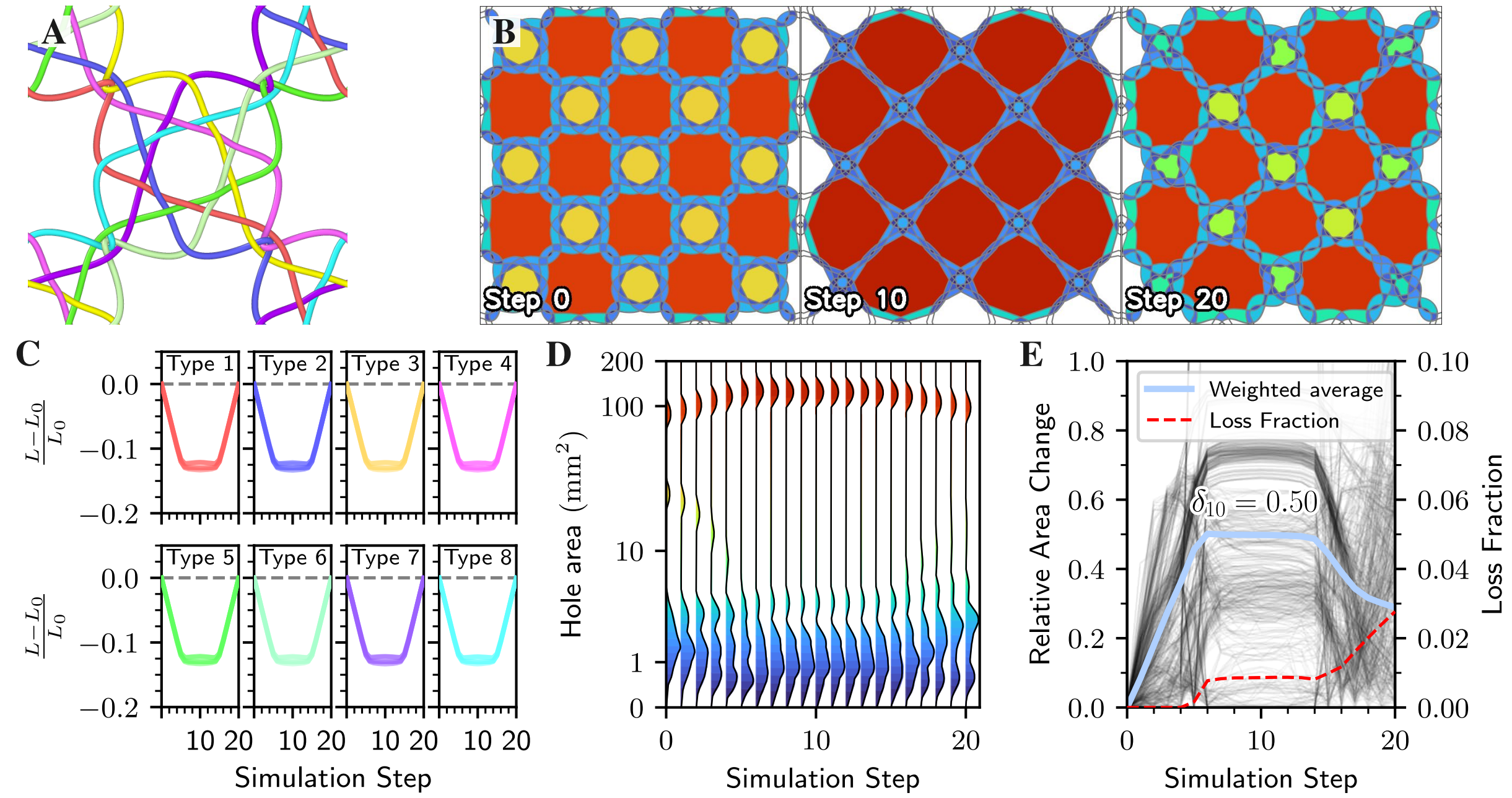

Figure S106: **Simulation of lace structure Rose-2. A** Unit-cell schematic (active bond types {1, 2, 3, 4, 5, 6, 7, 8}). **B** Snapshots at steps 0, 10, and 20, showing the initial structure, maximum deformation (active bonds shrink by 25%), and subsequent relaxation. **C** Variation of the relative length of yarns, grouped by type. **D** Evolution of the distribution of pore areas over time. **E** Relative area change of individual pores.

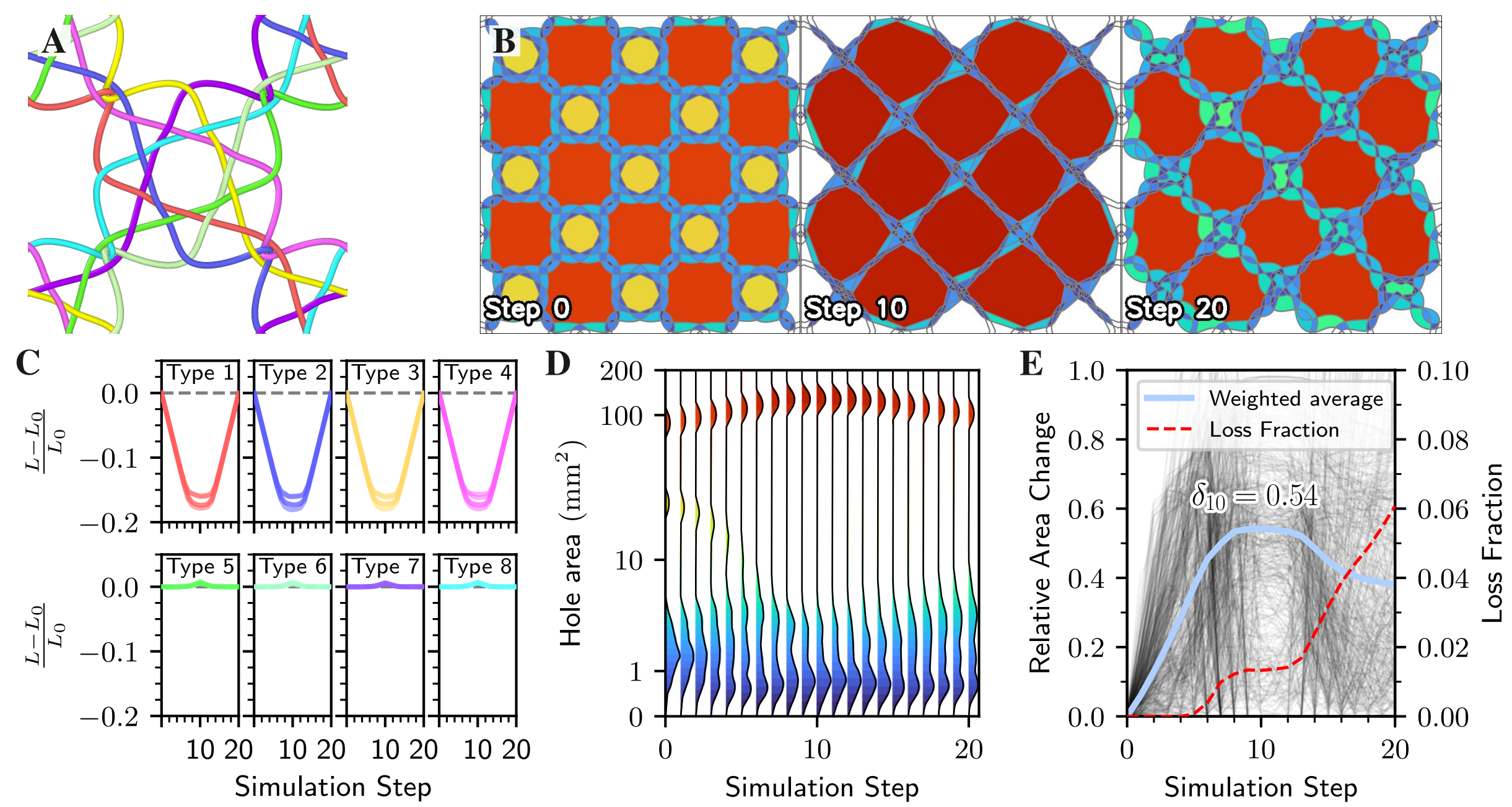

Figure S107: **Simulation of lace structure Rose-2. A** Unit-cell schematic (active bond types {1, 2, 3, 4}). **B** Snapshots at steps 0, 10, and 20, showing the initial structure, maximum deformation (active bonds shrink by 25%), and subsequent relaxation. **C** Variation of the relative length of yarns, grouped by type. **D** Evolution of the distribution of pore areas over time. **E** Relative area change of individual pores.

Rose-3

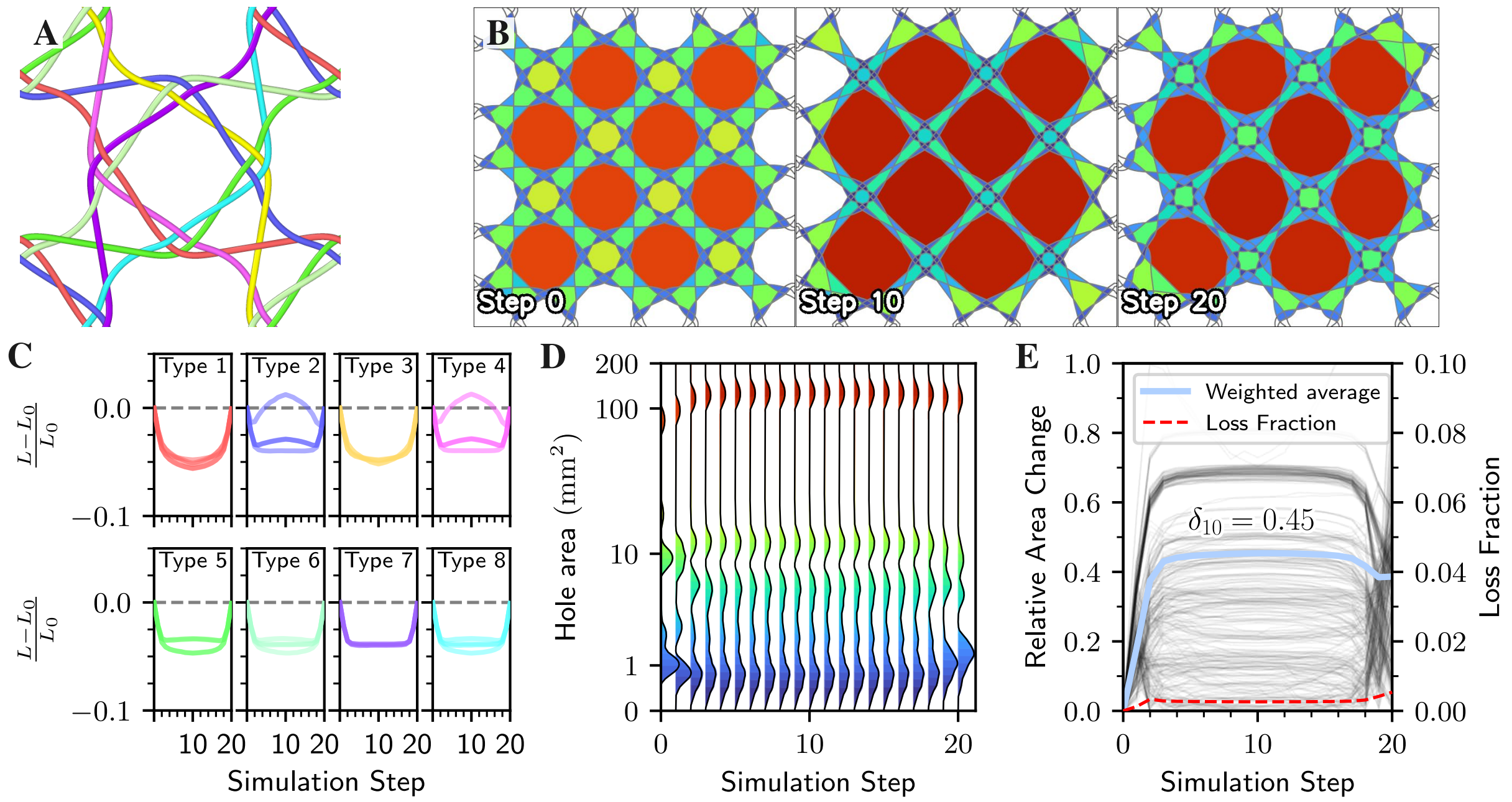

Figure S108: **Simulation of lace structure Rose-3. A** Unit-cell schematic (active bond types {1, 2, 3, 4, 5, 6, 7, 8}). **B** Snapshots at steps 0, 10, and 20, showing the initial structure, maximum deformation (active bonds shrink by 25%), and subsequent relaxation. **C** Variation of the relative length of yarns, grouped by type. **D** Evolution of the distribution of pore areas over time. **E** Relative area change of individual pores.

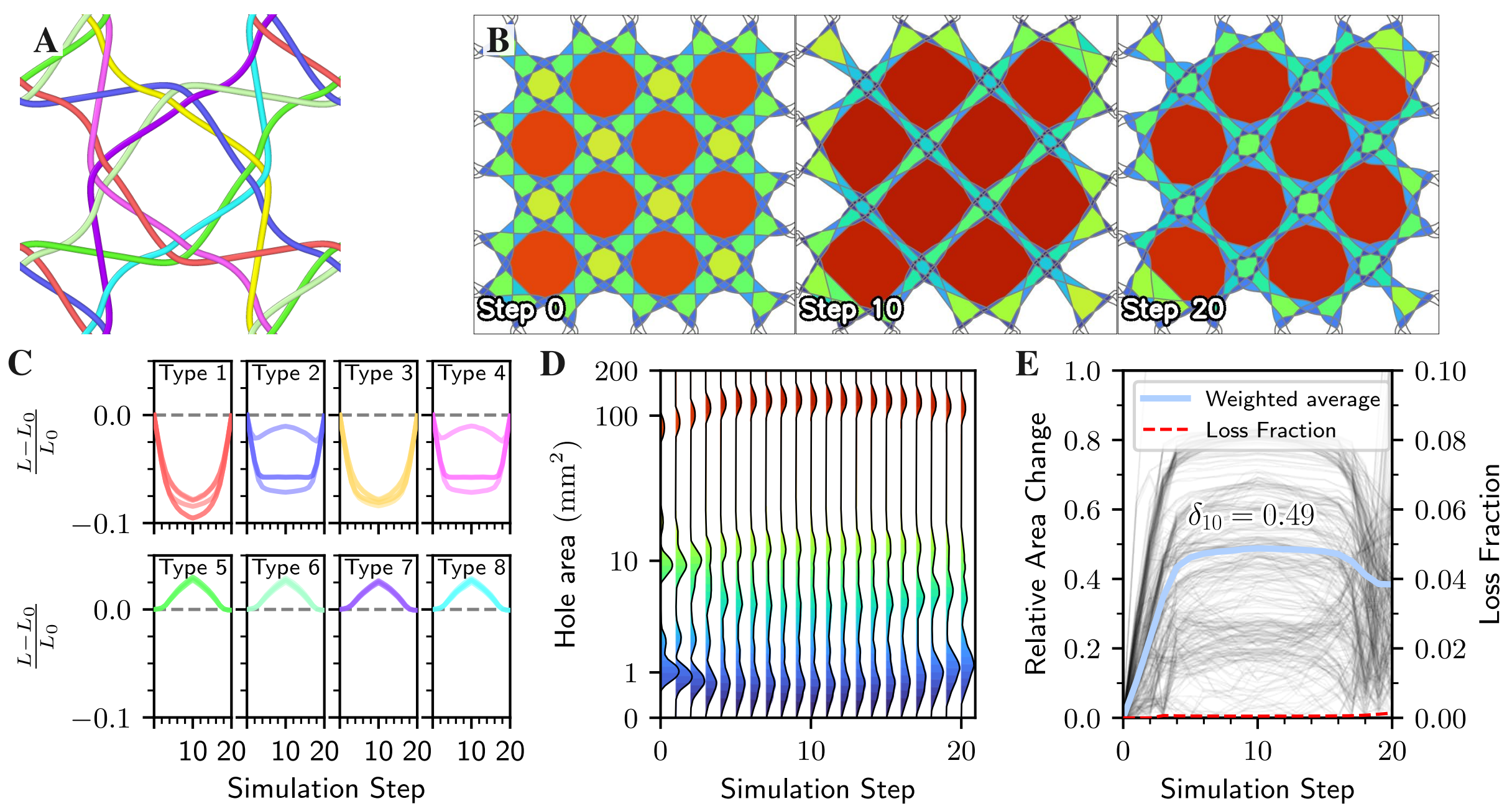

Figure S109: **Simulation of lace structure Rose-3. A** Unit-cell schematic (active bond types {1, 2, 3, 4}). **B** Snapshots at steps 0, 10, and 20, showing the initial structure, maximum deformation (active bonds shrink by 25%), and subsequent relaxation. **C** Variation of the relative length of yarns, grouped by type. **D** Evolution of the distribution of pore areas over time. **E** Relative area change of individual pores.

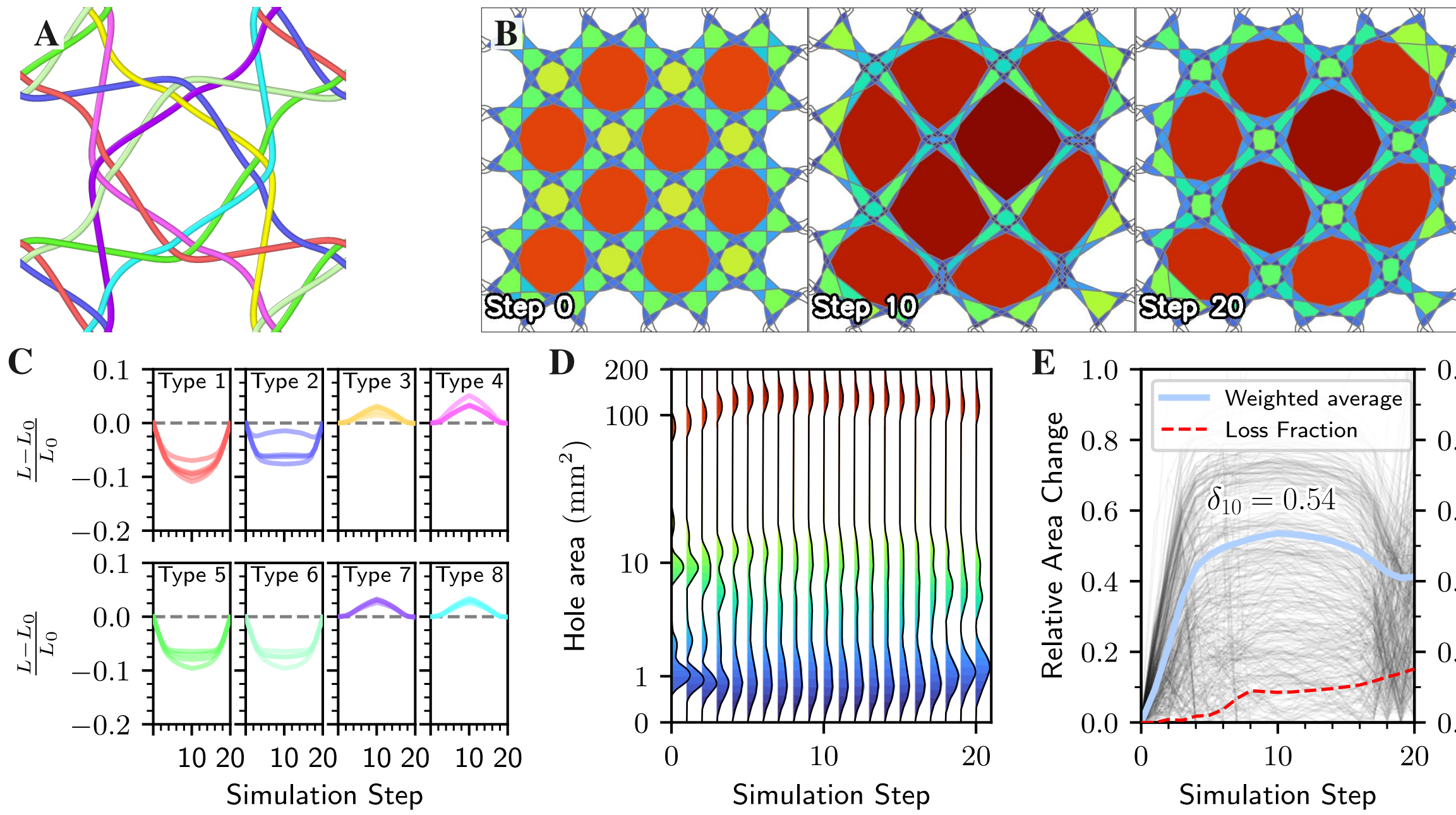

Figure S110: **Simulation of lace structure Rose-3. A** Unit-cell schematic (active bond types {1, 2, 5, 6}). **B** Snapshots at steps 0, 10, and 20, showing the initial structure, maximum deformation (active bonds shrink by 25%), and subsequent relaxation. **C** Variation of the relative length of yarns, grouped by type. **D** Evolution of the distribution of pore areas over time. **E** Relative area change of individual pores.

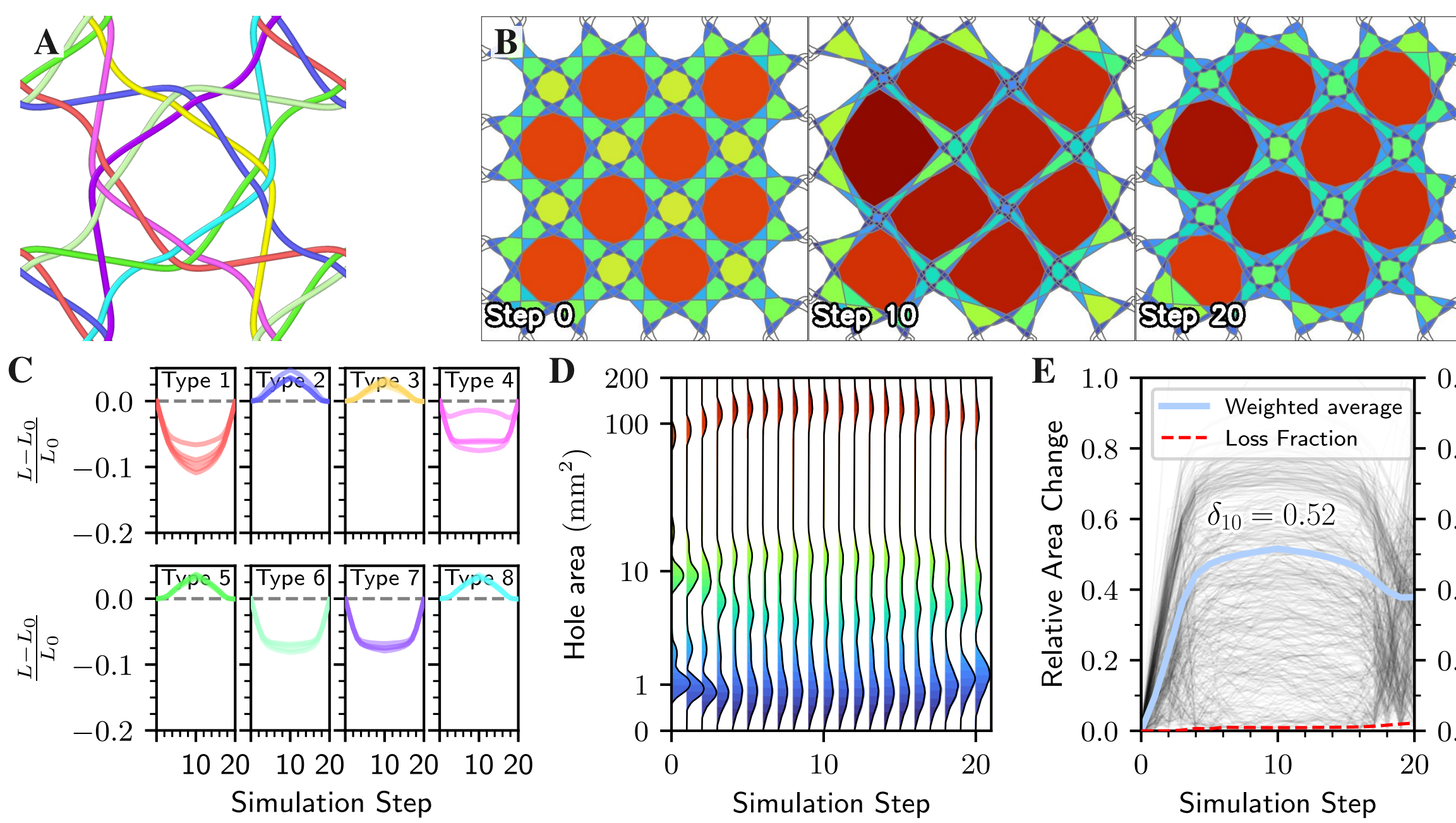

Figure S111: **Simulation of lace structure Rose-3. A** Unit-cell schematic (active bond types {1, 4, 6, 7}). **B** Snapshots at steps 0, 10, and 20, showing the initial structure, maximum deformation (active bonds shrink by 25%), and subsequent relaxation. **C** Variation of the relative length of yarns, grouped by type. **D** Evolution of the distribution of pore areas over time. **E** Relative area change of individual pores.

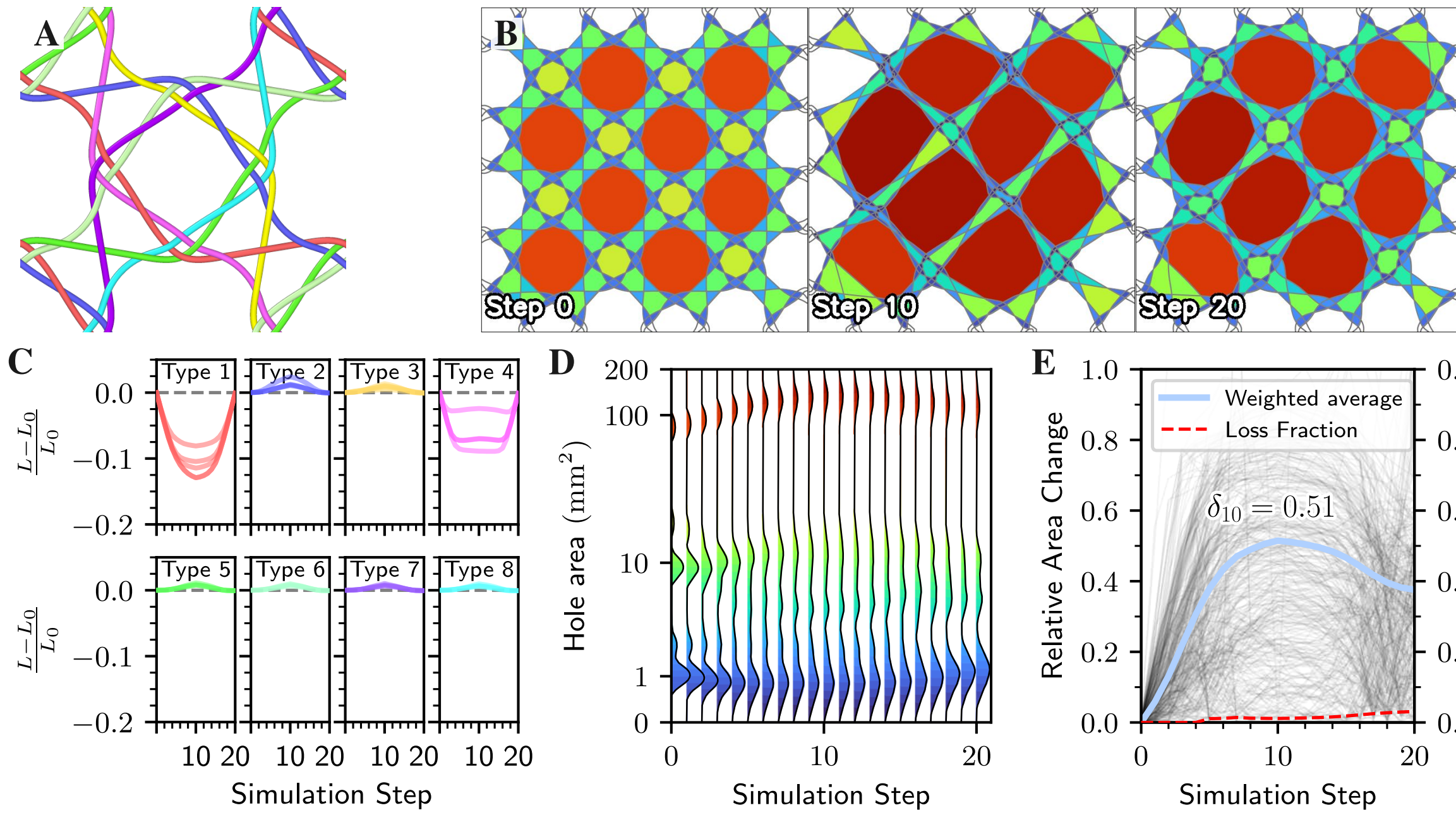

Figure S112: **Simulation of lace structure Rose-3. A** Unit-cell schematic (active bond types {1, 4}). **B** Snapshots at steps 0, 10, and 20, showing the initial structure, maximum deformation (active bonds shrink by 25%), and subsequent relaxation. **C** Variation of the relative length of yarns, grouped by type. **D** Evolution of the distribution of pore areas over time. **E** Relative area change of individual pores.

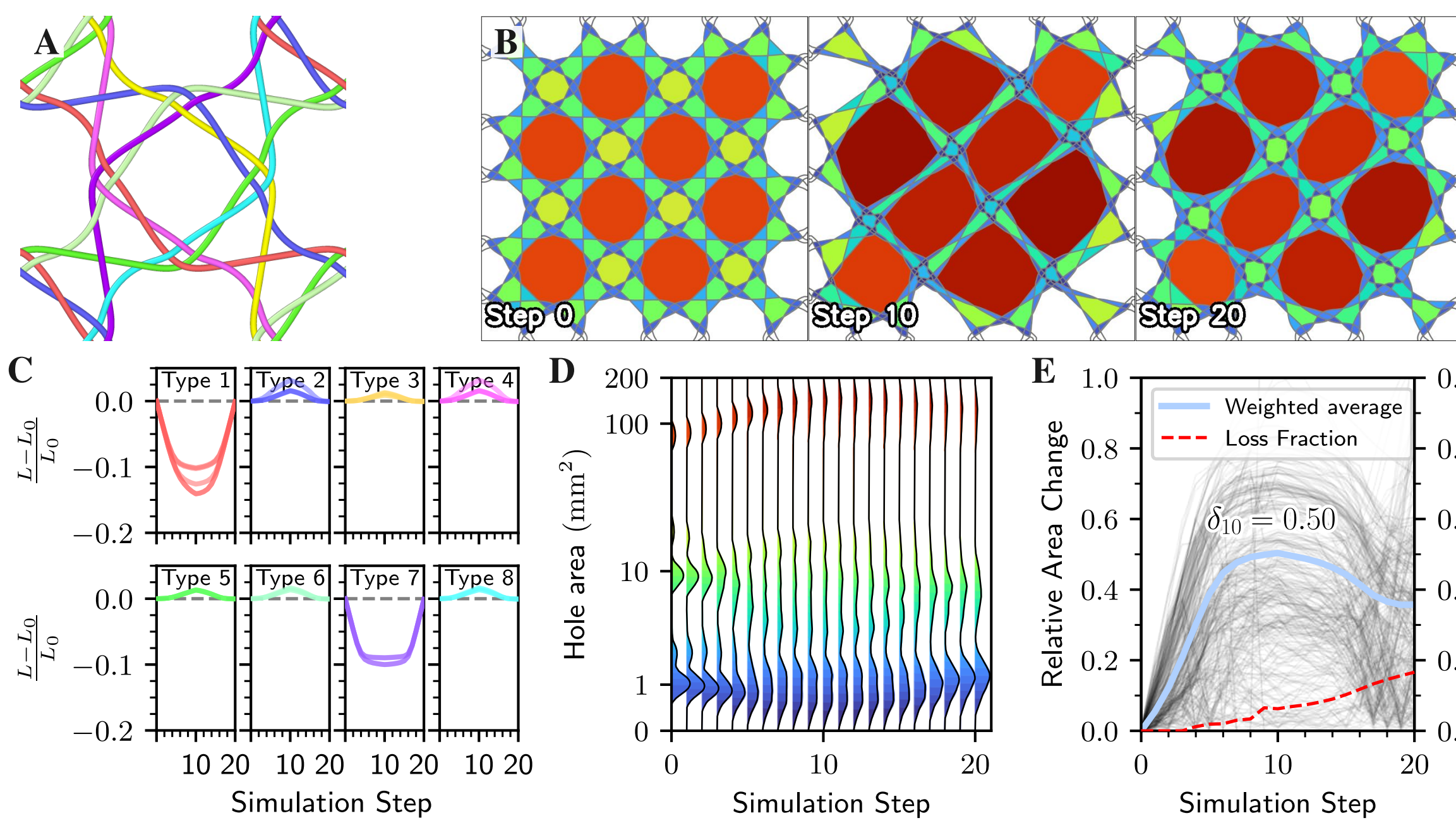

Figure S113: **Simulation of lace structure Rose-3. A** Unit-cell schematic (active bond types {1, 7}). **B** Snapshots at steps 0, 10, and 20, showing the initial structure, maximum deformation (active bonds shrink by 25%), and subsequent relaxation. **C** Variation of the relative length of yarns, grouped by type. **D** Evolution of the distribution of pore areas over time. **E** Relative area change of individual pores.

Rose-4

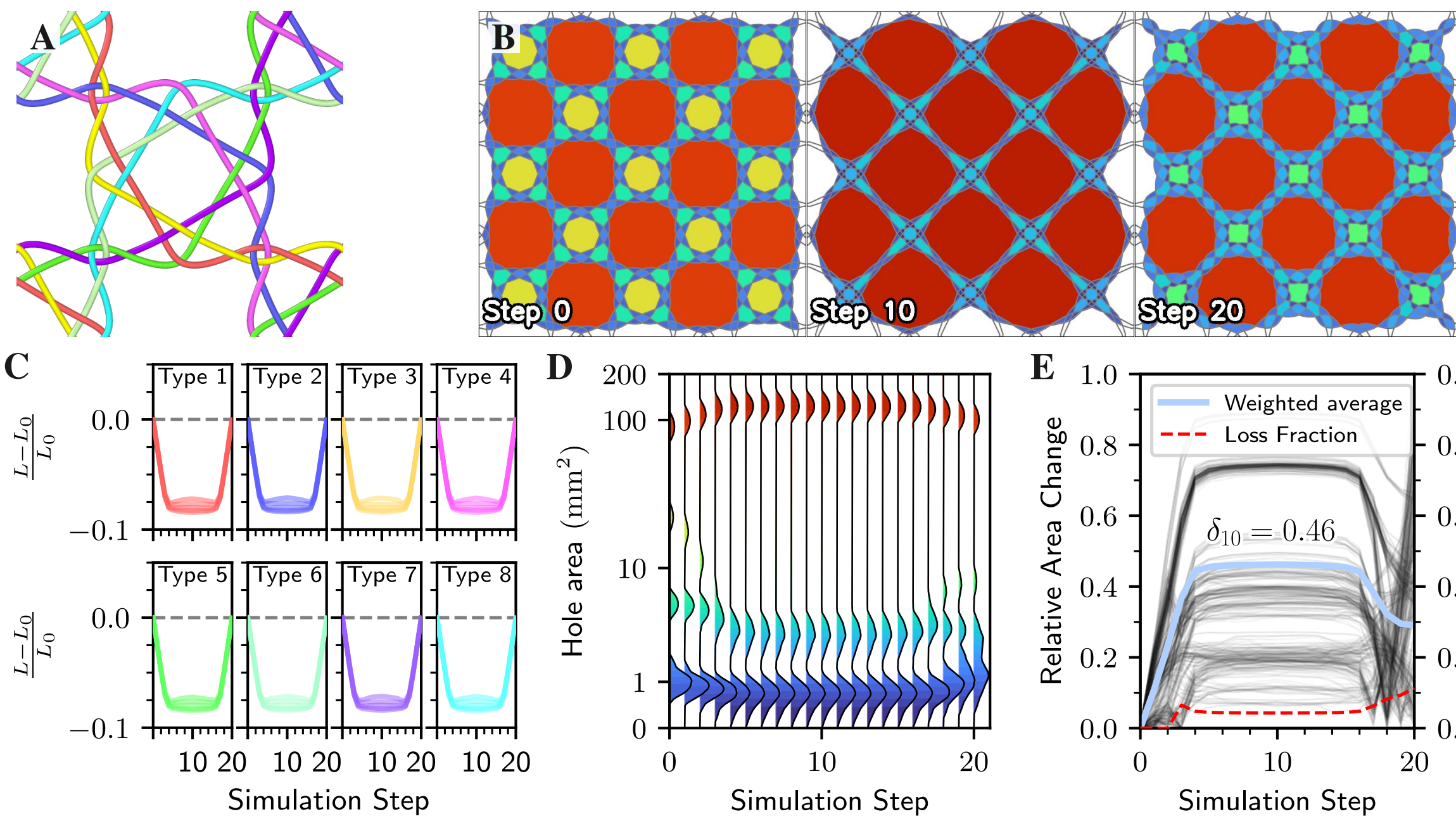

Figure S114: **Simulation of lace structure Rose-4. A** Unit-cell schematic (active bond types {1, 2, 3, 4, 5, 6, 7, 8}). **B** Snapshots at steps 0, 10, and 20, showing the initial structure, maximum deformation (active bonds shrink by 25%), and subsequent relaxation. **C** Variation of the relative length of yarns, grouped by type. **D** Evolution of the distribution of pore areas over time. **E** Relative area change of individual pores.

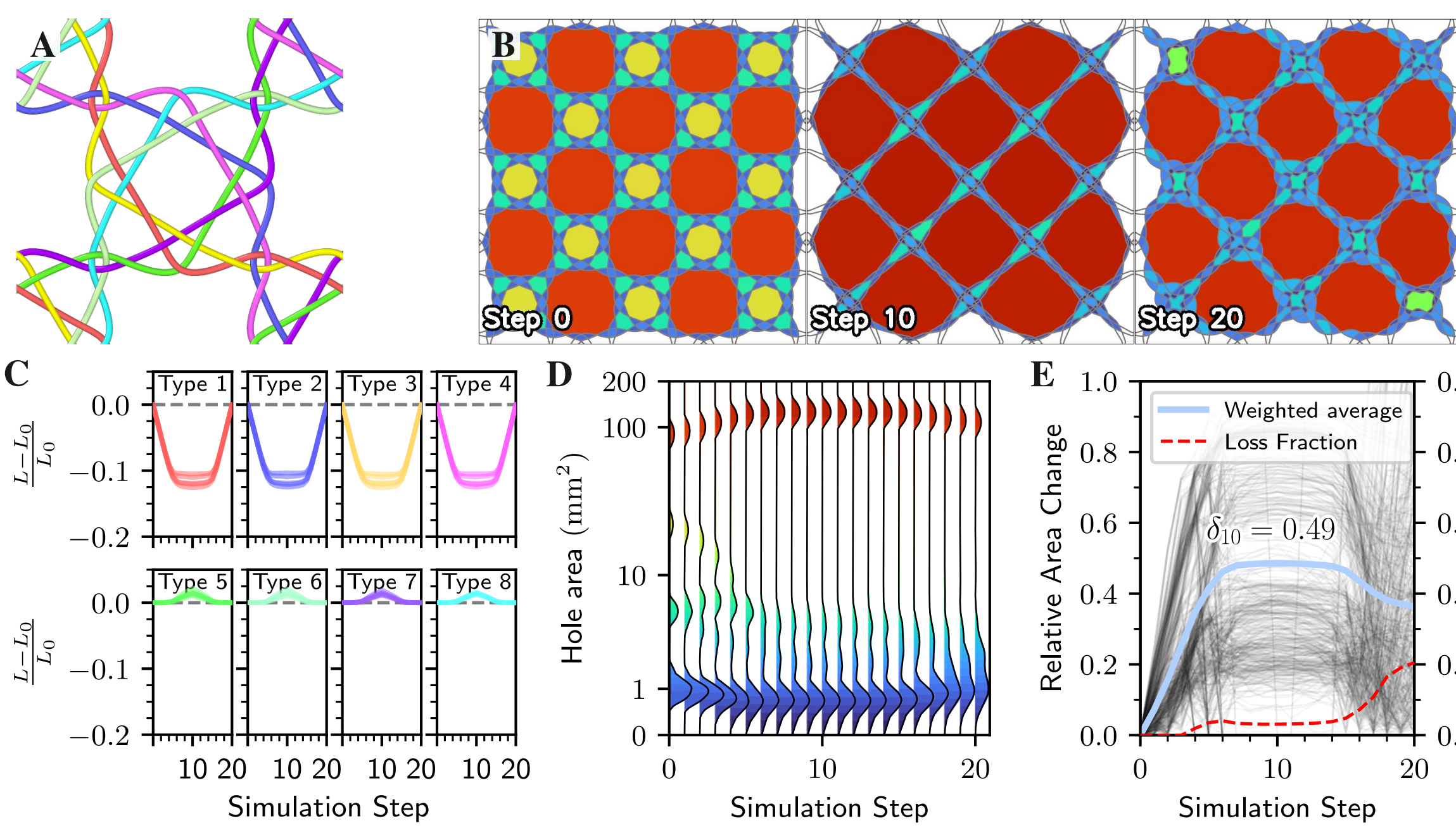

Figure S115: **Simulation of lace structure Rose-4. A** Unit-cell schematic (active bond types {1, 2, 3, 4}). **B** Snapshots at steps 0, 10, and 20, showing the initial structure, maximum deformation (active bonds shrink by 25%), and subsequent relaxation. **C** Variation of the relative length of yarns, grouped by type. **D** Evolution of the distribution of pore areas over time. **E** Relative area change of individual pores.

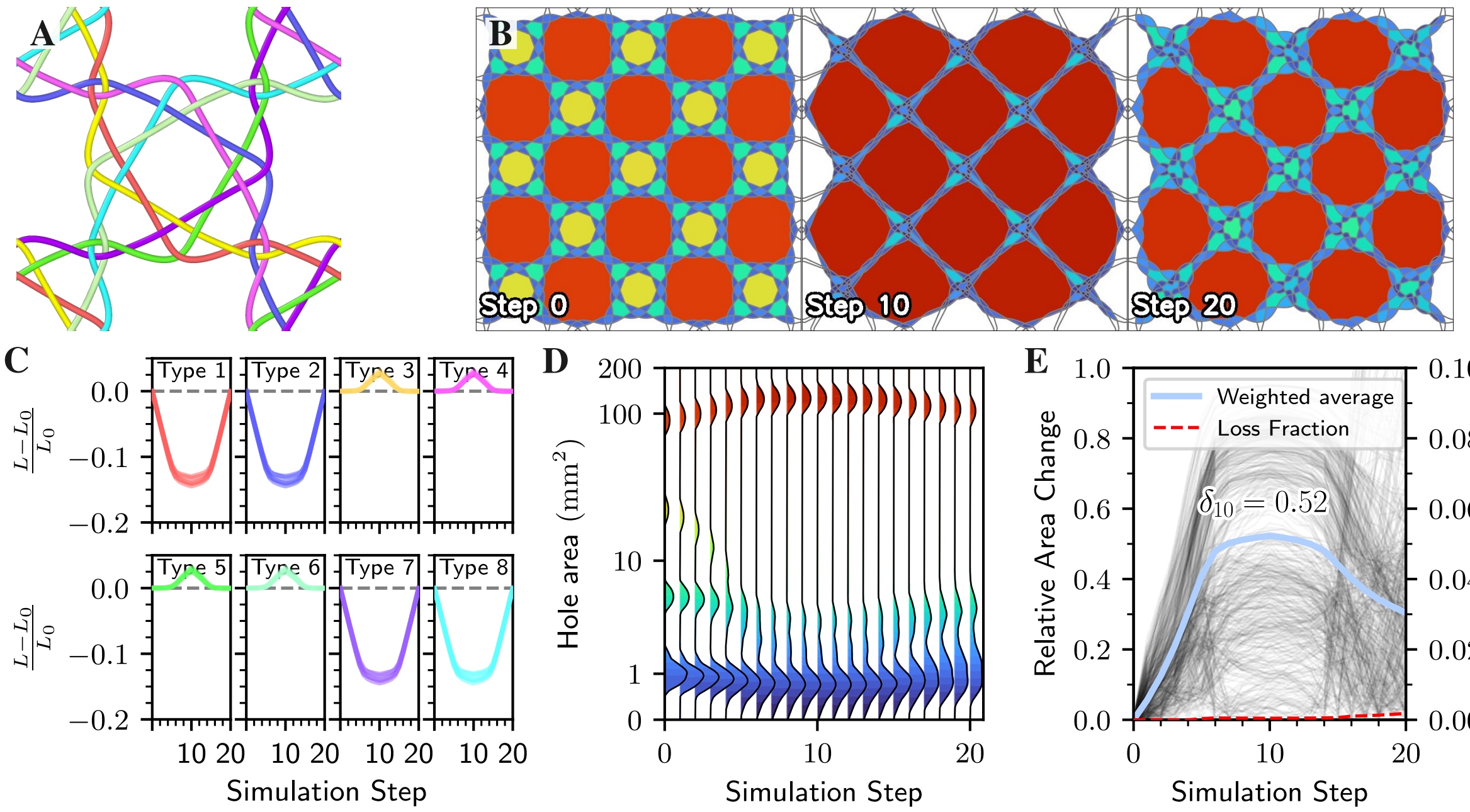

Figure S116: **Simulation of lace structure Rose-4. A** Unit-cell schematic (active bond types {1, 2, 7, 8}). **B** Snapshots at steps 0, 10, and 20, showing the initial structure, maximum deformation (active bonds shrink by 25%), and subsequent relaxation. **C** Variation of the relative length of yarns, grouped by type. **D** Evolution of the distribution of pore areas over time. **E** Relative area change of individual pores.

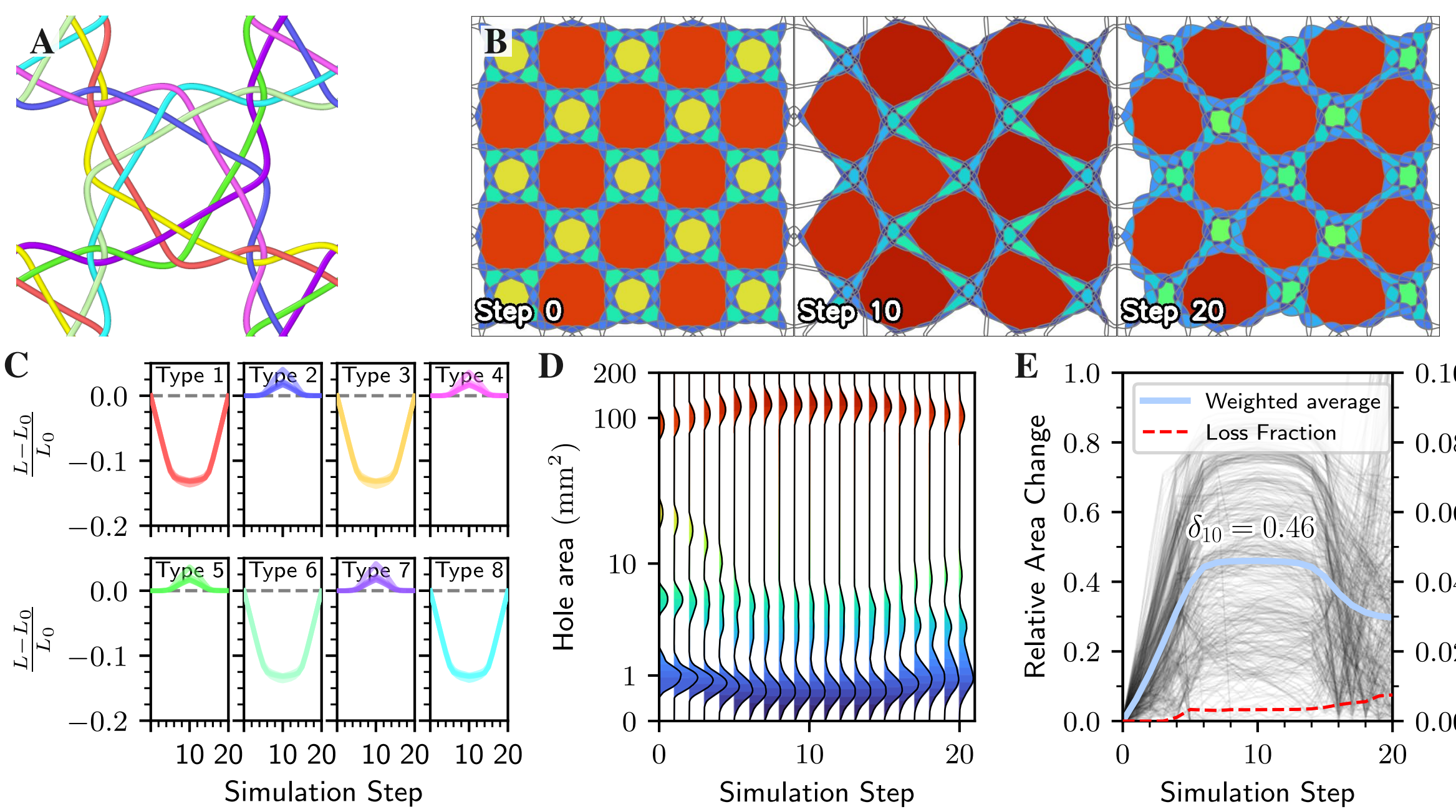

Figure S117: **Simulation of lace structure Rose-4. A** Unit-cell schematic (active bond types {1, 3. 6, 8}). **B** Snapshots at steps 0, 10, and 20, showing the initial structure, maximum deformation (active bonds shrink by 25%), and subsequent relaxation. **C** Variation of the relative length of yarns, grouped by type. **D** Evolution of the distribution of pore areas over time. **E** Relative area change of individual pores.

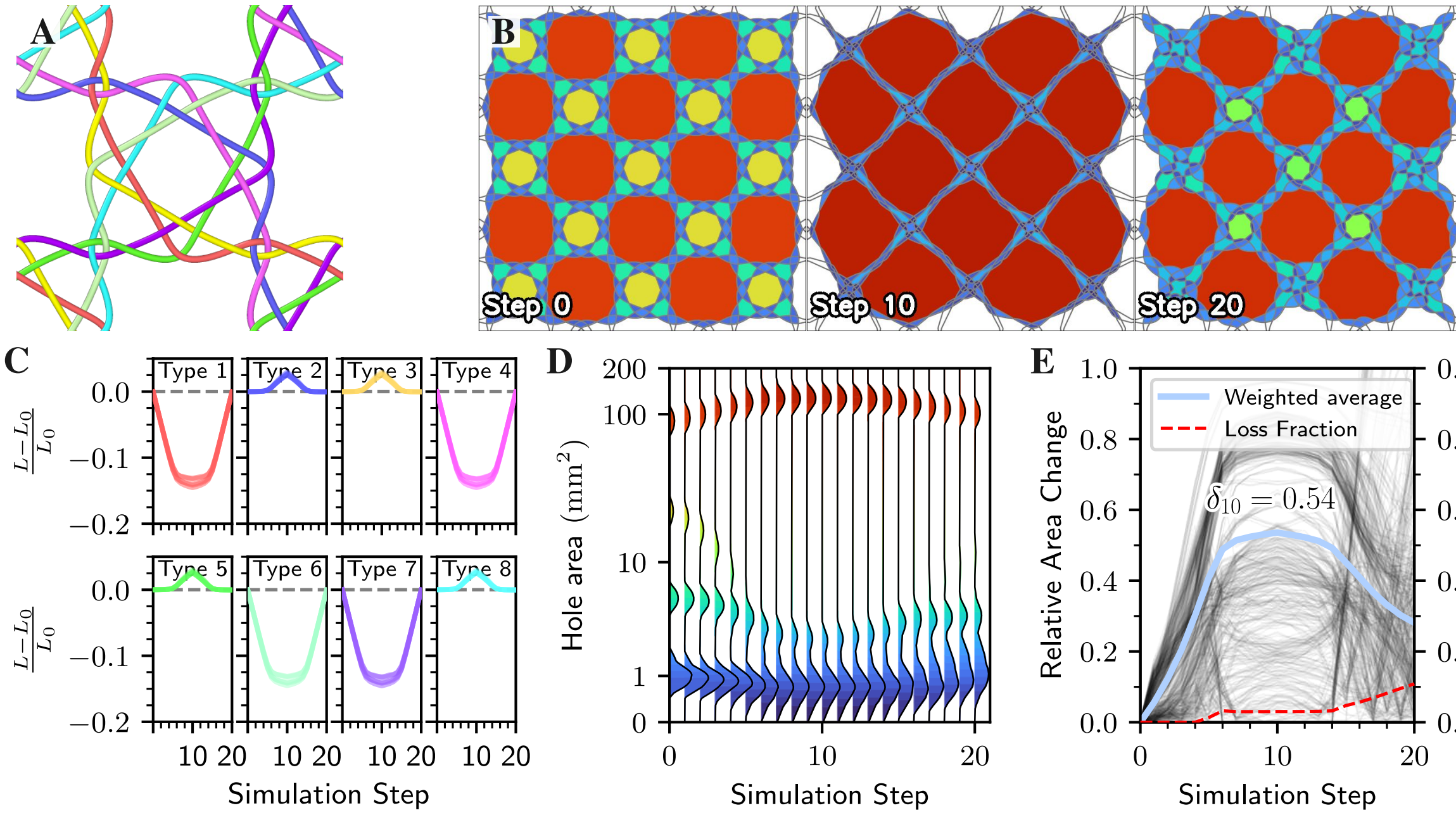

Figure S118: **Simulation of lace structure Rose-4. A** Unit-cell schematic (active bond types {1, 4, 6, 7}). **B** Snapshots at steps 0, 10, and 20, showing the initial structure, maximum deformation (active bonds shrink by 25%), and subsequent relaxation. **C** Variation of the relative length of yarns, grouped by type. **D** Evolution of the distribution of pore areas over time. **E** Relative area change of individual pores.

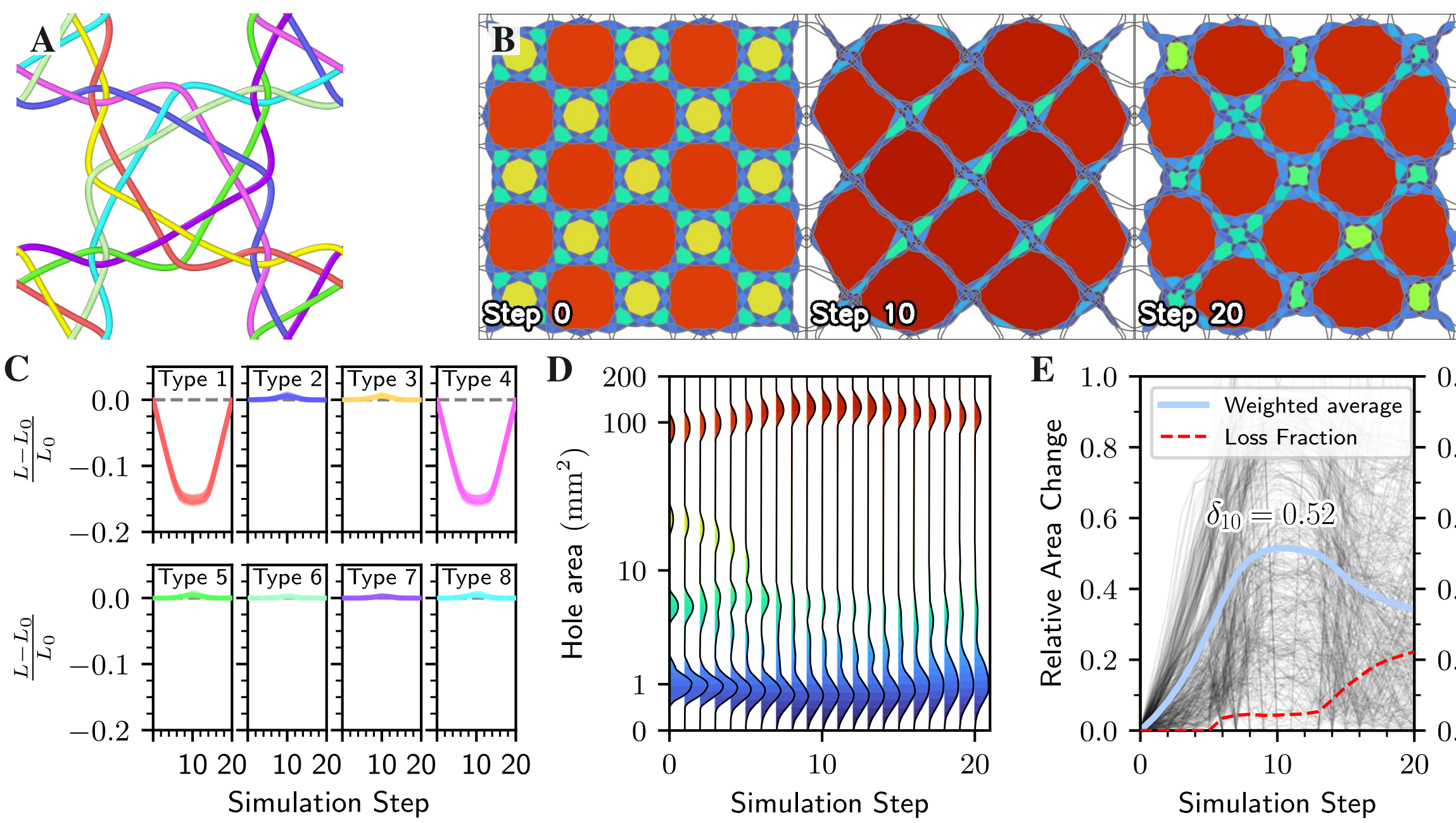

Figure S119: **Simulation of lace structure Rose-4. A** Unit-cell schematic (active bond types {1, 4}). **B** Snapshots at steps 0, 10, and 20, showing the initial structure, maximum deformation (active bonds shrink by 25%), and subsequent relaxation. **C** Variation of the relative length of yarns, grouped by type. **D** Evolution of the distribution of pore areas over time. **E** Relative area change of individual pores.

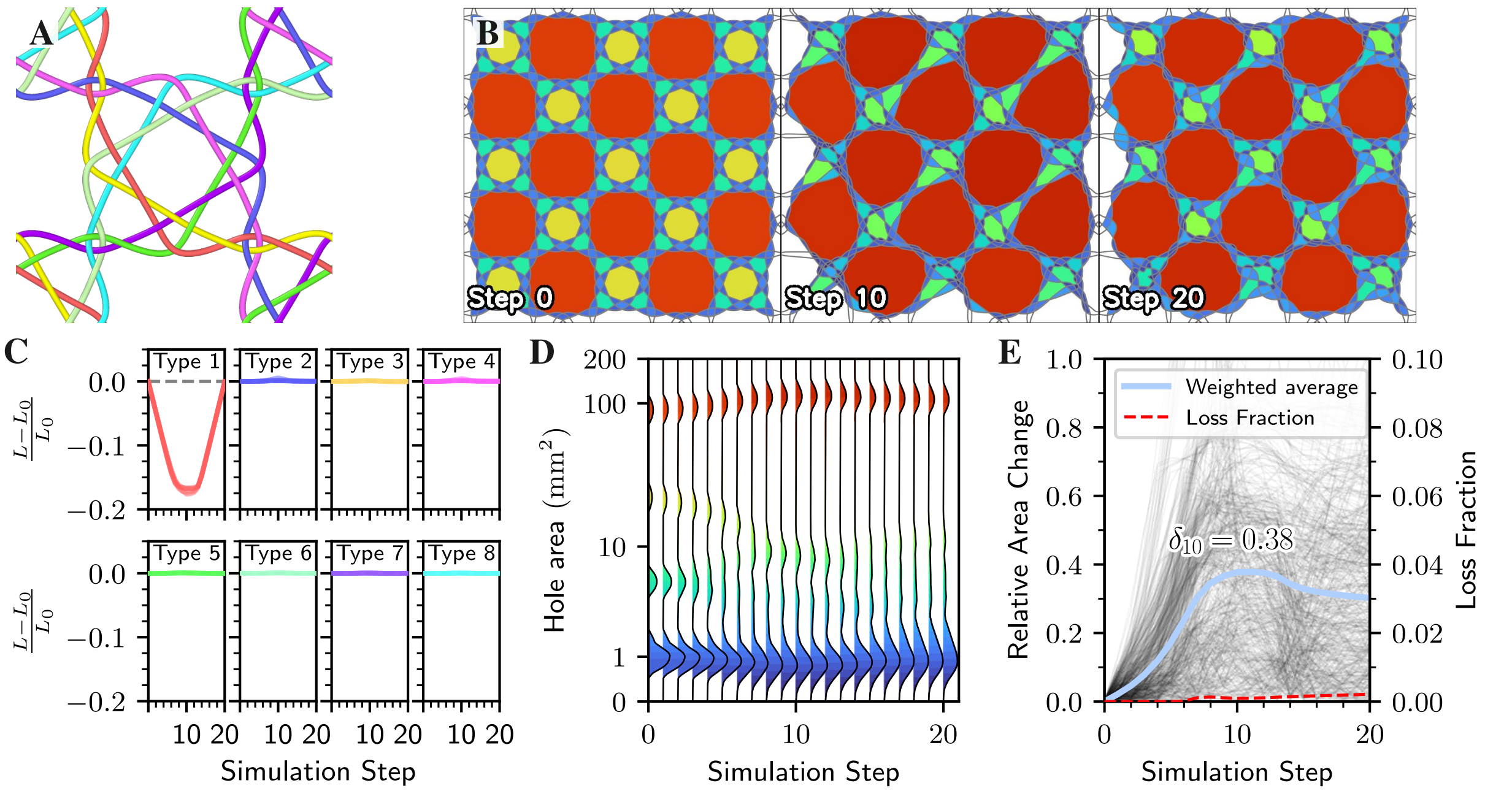

Figure S120: **Simulation of lace structure Rose-4.** **A** Unit-cell schematic (active bond types {1}). **B** Snapshots at steps 0, 10, and 20, showing the initial structure, maximum deformation (active bonds shrink by 25%), and subsequent relaxation. **C** Variation of the relative length of yarns, grouped by type. **D** Evolution of the distribution of pore areas over time. **E** Relative area change of individual pores.

Rose-5

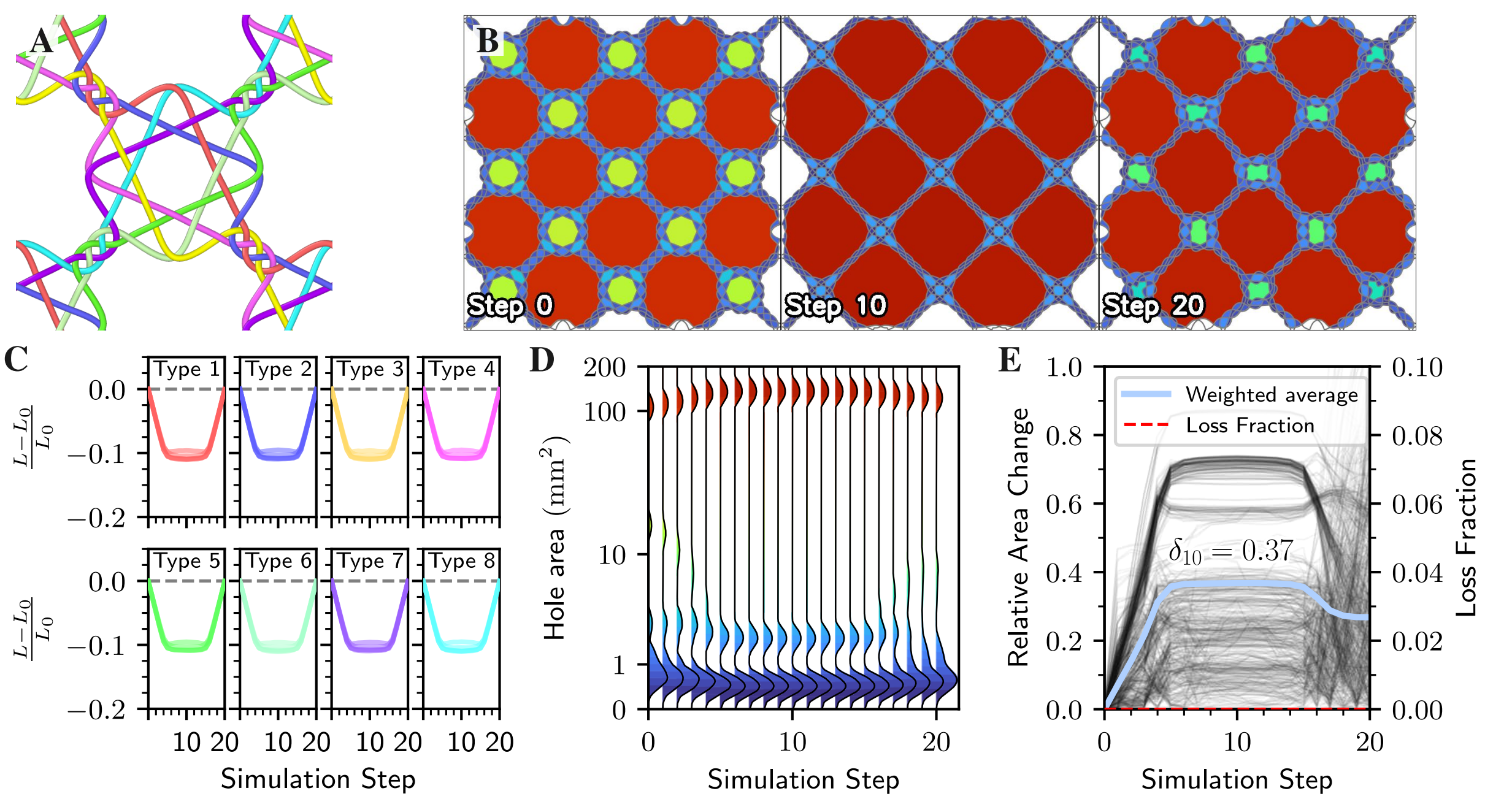

Figure S121: **Simulation of lace structure Rose-5.** **A** Unit-cell schematic (active bond types {1, 2, 3, 4, 5, 6, 7, 8}). **B** Snapshots at steps 0, 10, and 20, showing the initial structure, maximum deformation (active bonds shrink by 25%), and subsequent relaxation. **C** Variation of the relative length of yarns, grouped by type. **D** Evolution of the distribution of pore areas over time. **E** Relative area change of individual pores.

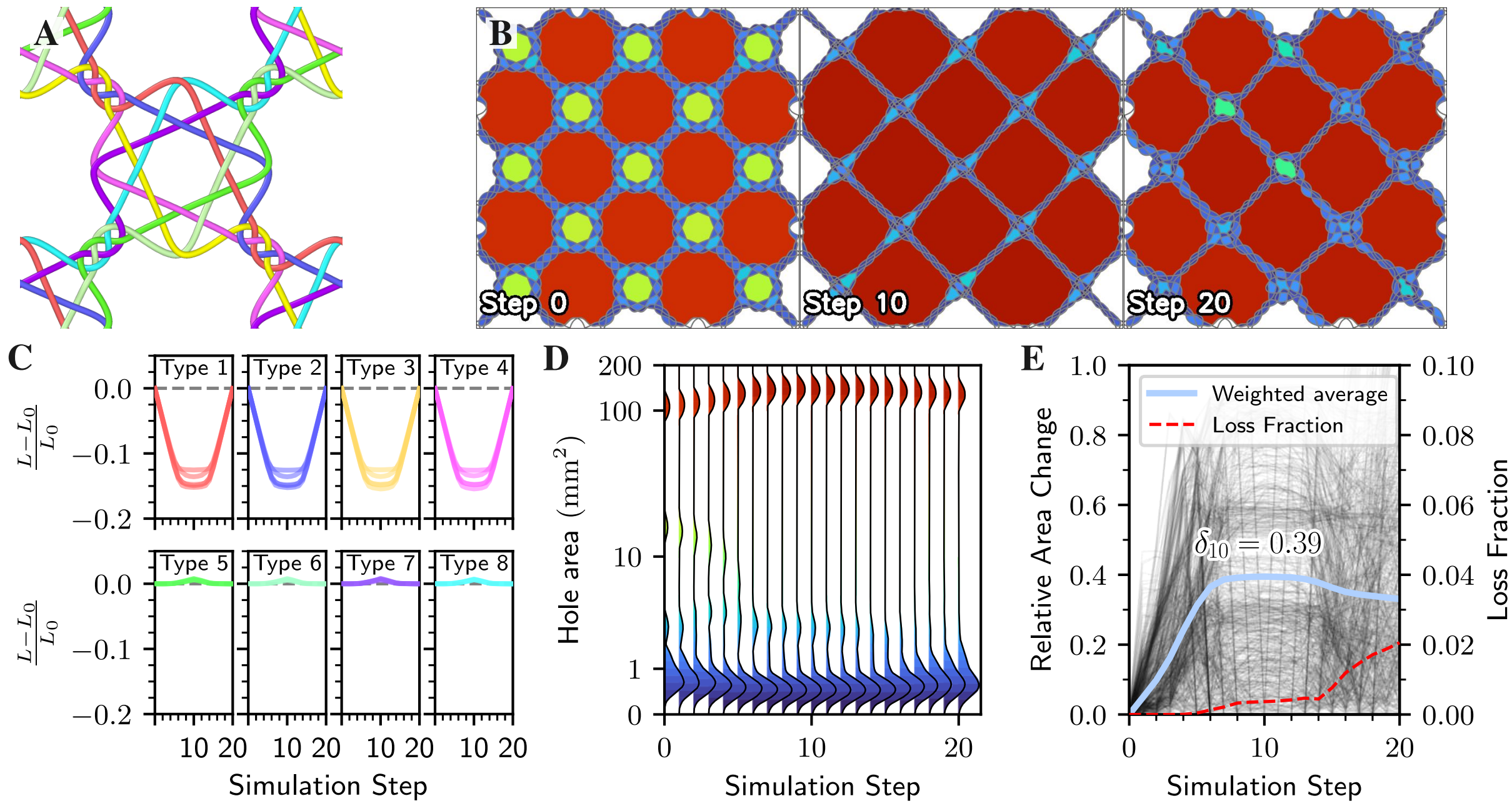

Figure S122: **Simulation of lace structure Rose-5. A** Unit-cell schematic (active bond types {1, 2, 3, 4}). **B** Snapshots at steps 0, 10, and 20, showing the initial structure, maximum deformation (active bonds shrink by 25%), and subsequent relaxation. **C** Variation of the relative length of yarns, grouped by type. **D** Evolution of the distribution of pore areas over time. **E** Relative area change of individual pores.

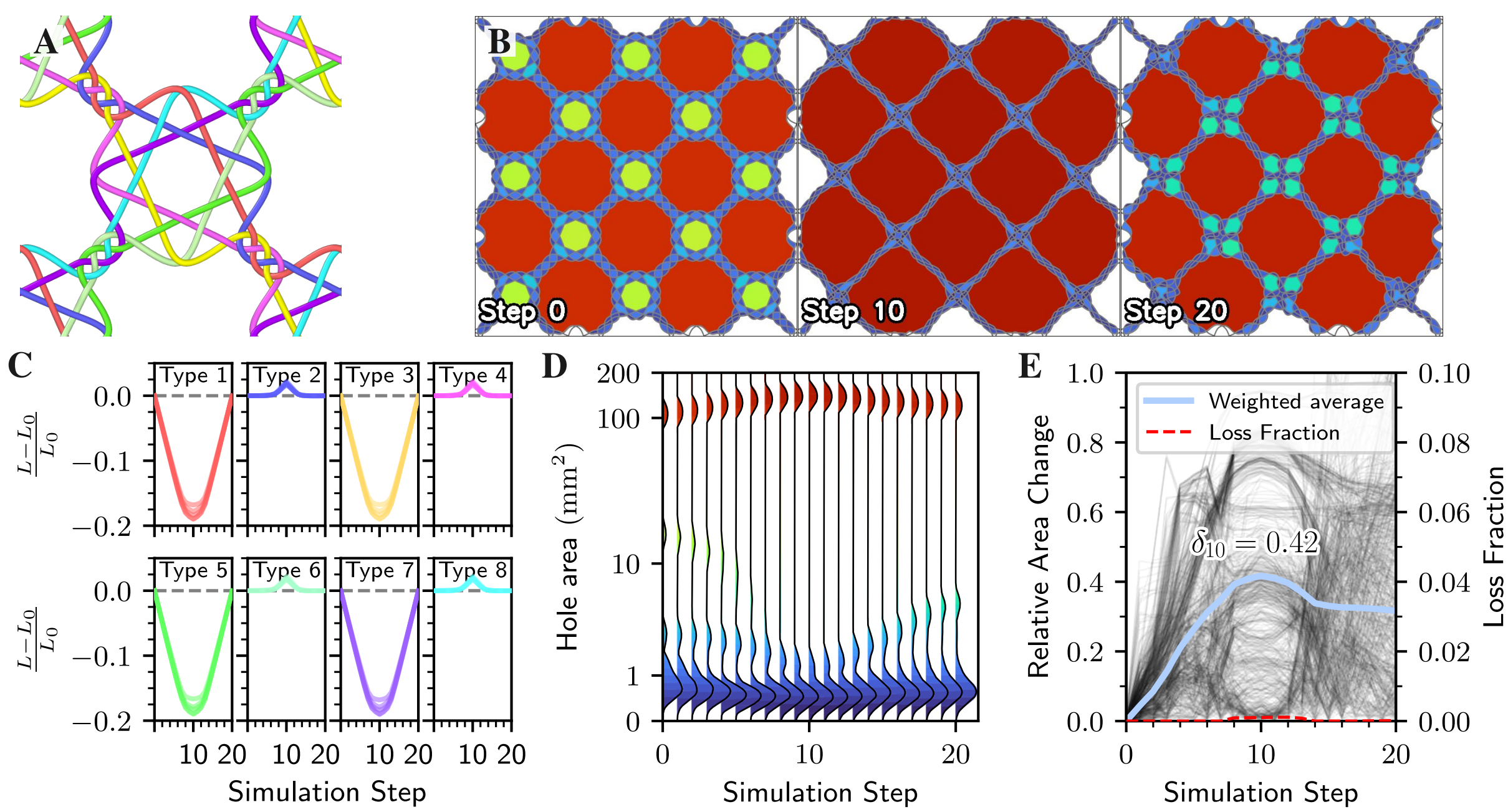

Figure S123: **Simulation of lace structure Rose-5. A** Unit-cell schematic (active bond types {1, 3, 5, 7}). **B** Snapshots at steps 0, 10, and 20, showing the initial structure, maximum deformation (active bonds shrink by 25%), and subsequent relaxation. **C** Variation of the relative length of yarns, grouped by type. **D** Evolution of the distribution of pore areas over time. **E** Relative area change of individual pores.

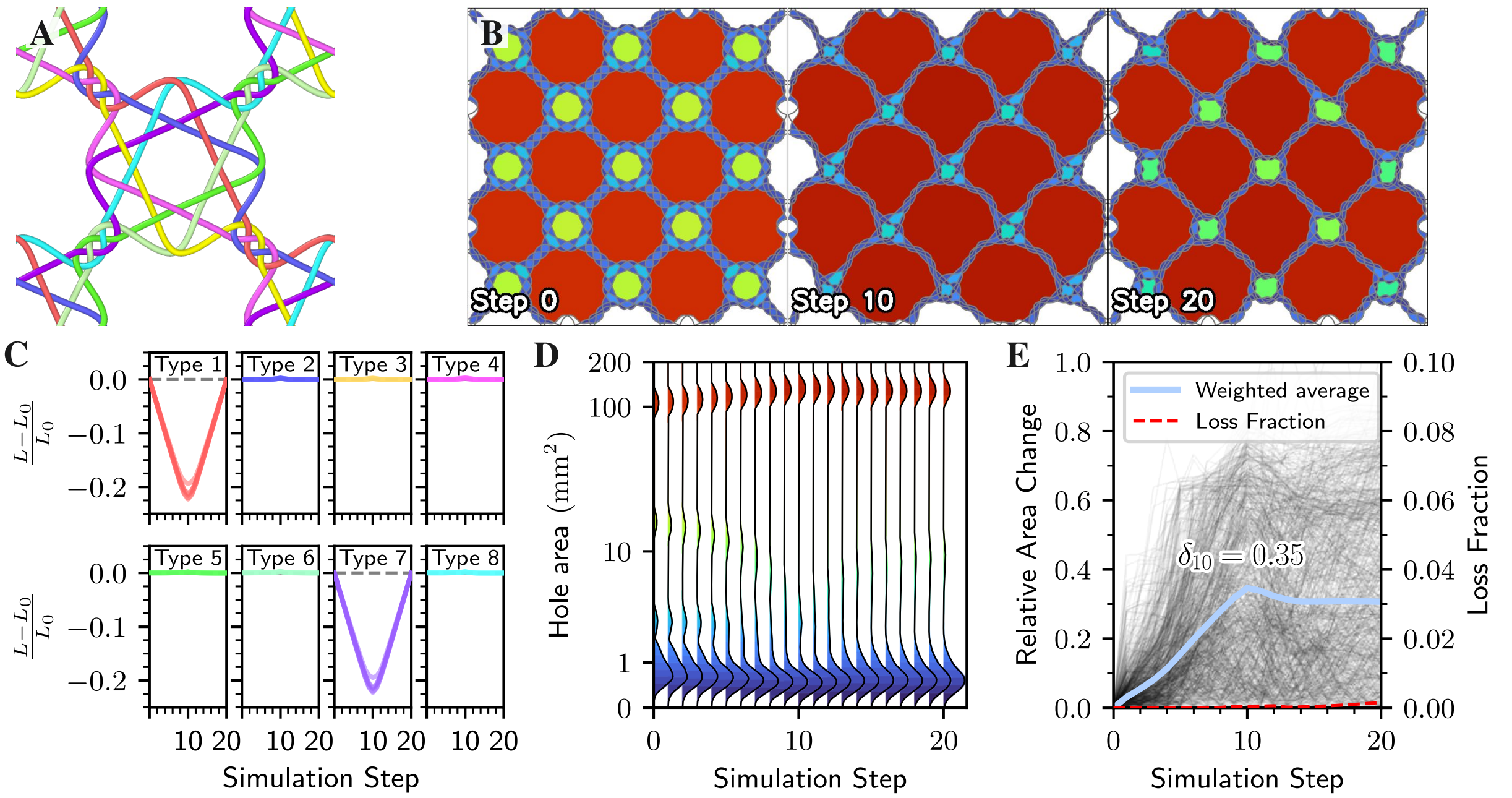

Figure S124: **Simulation of lace structure Rose-5. A** Unit-cell schematic (active bond types {1, 7}). **B** Snapshots at steps 0, 10, and 20, showing the initial structure, maximum deformation (active bonds shrink by 25%), and subsequent relaxation. **C** Variation of the relative length of yarns, grouped by type. **D** Evolution of the distribution of pore areas over time. **E** Relative area change of individual pores.

Rose-6

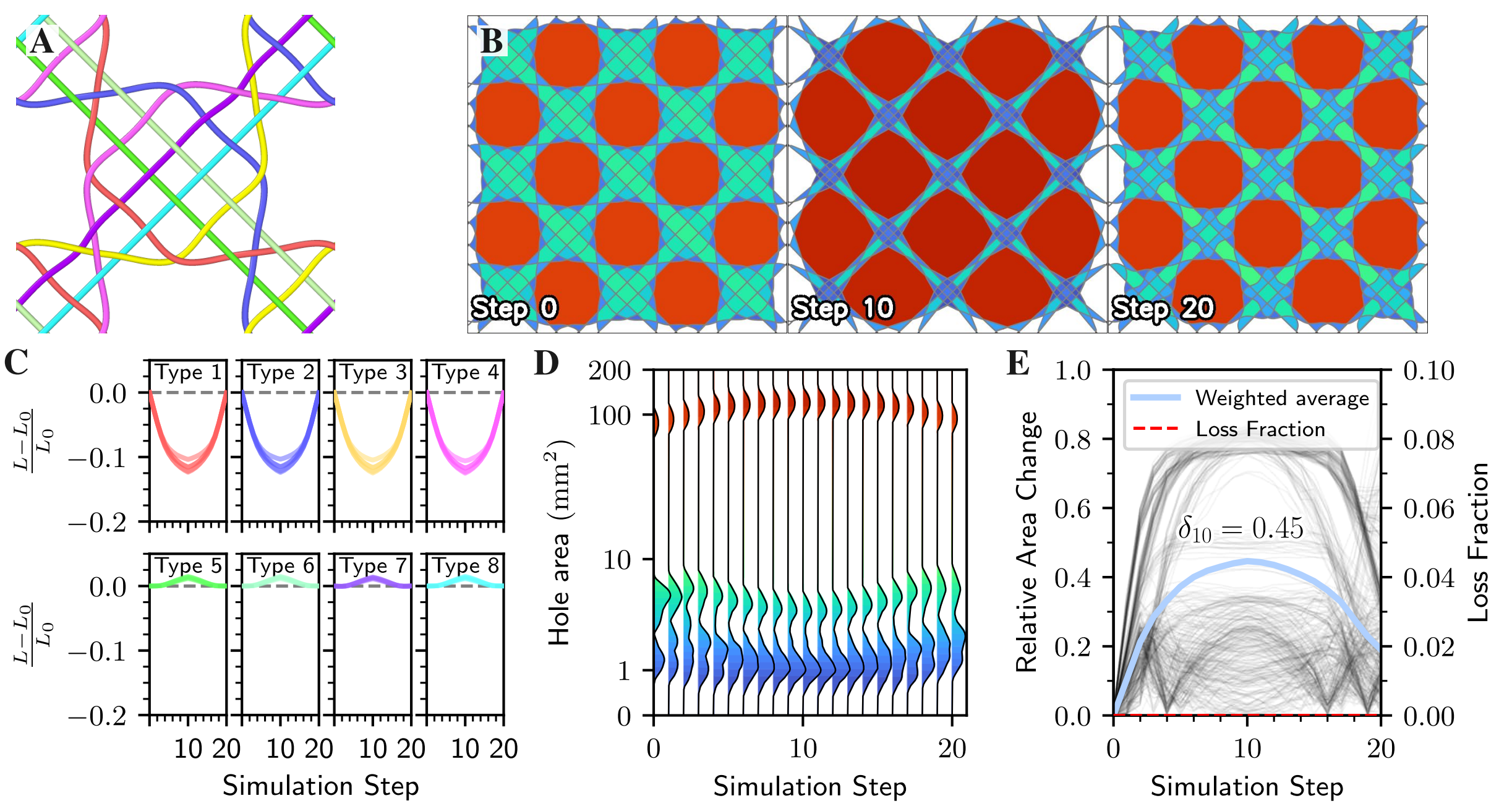

Figure S125: **Simulation of lace structure Rose-6. A** Unit-cell schematic (active bond types {1, 2, 3, 4, 5, 6, 7, 8}). **B** Snapshots at steps 0, 10, and 20, showing the initial structure, maximum deformation (active bonds shrink by 25%), and subsequent relaxation. **C** Variation of the relative length of yarns, grouped by type. **D** Evolution of the distribution of pore areas over time. **E** Relative area change of individual pores.

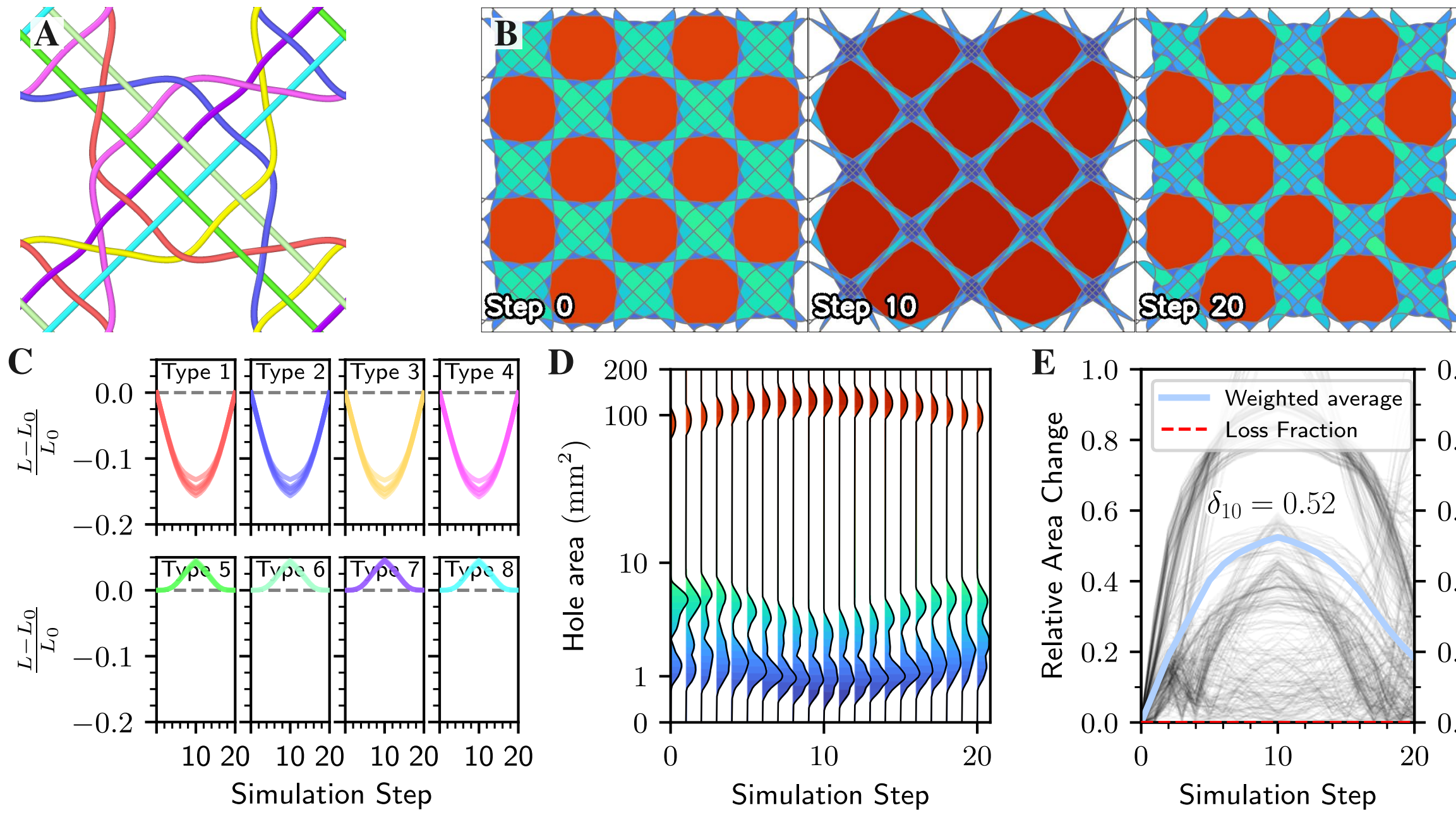

Figure S126: **Simulation of lace structure Rose-6. A** Unit-cell schematic (active bond types {1, 2, 3, 4}). **B** Snapshots at steps 0, 10, and 20, showing the initial structure, maximum deformation (active bonds shrink by 25%), and subsequent relaxation. **C** Variation of the relative length of yarns, grouped by type. **D** Evolution of the distribution of pore areas over time. **E** Relative area change of individual pores.

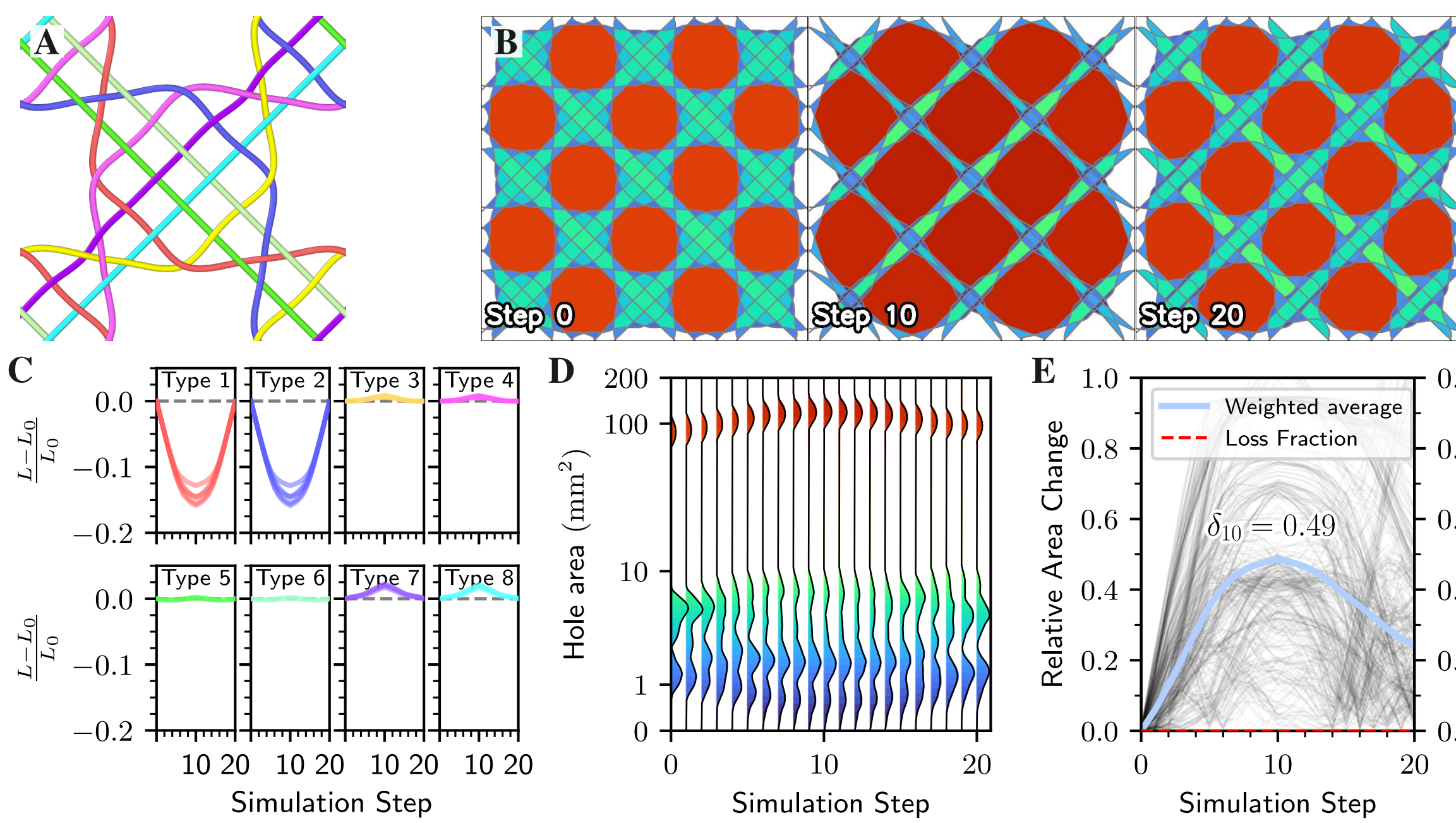

Figure S127: **Simulation of lace structure Rose-6. A** Unit-cell schematic (active bond types {1, 2, 5, 6}). **B** Snapshots at steps 0, 10, and 20, showing the initial structure, maximum deformation (active bonds shrink by 25%), and subsequent relaxation. **C** Variation of the relative length of yarns, grouped by type. **D** Evolution of the distribution of pore areas over time. **E** Relative area change of individual pores.

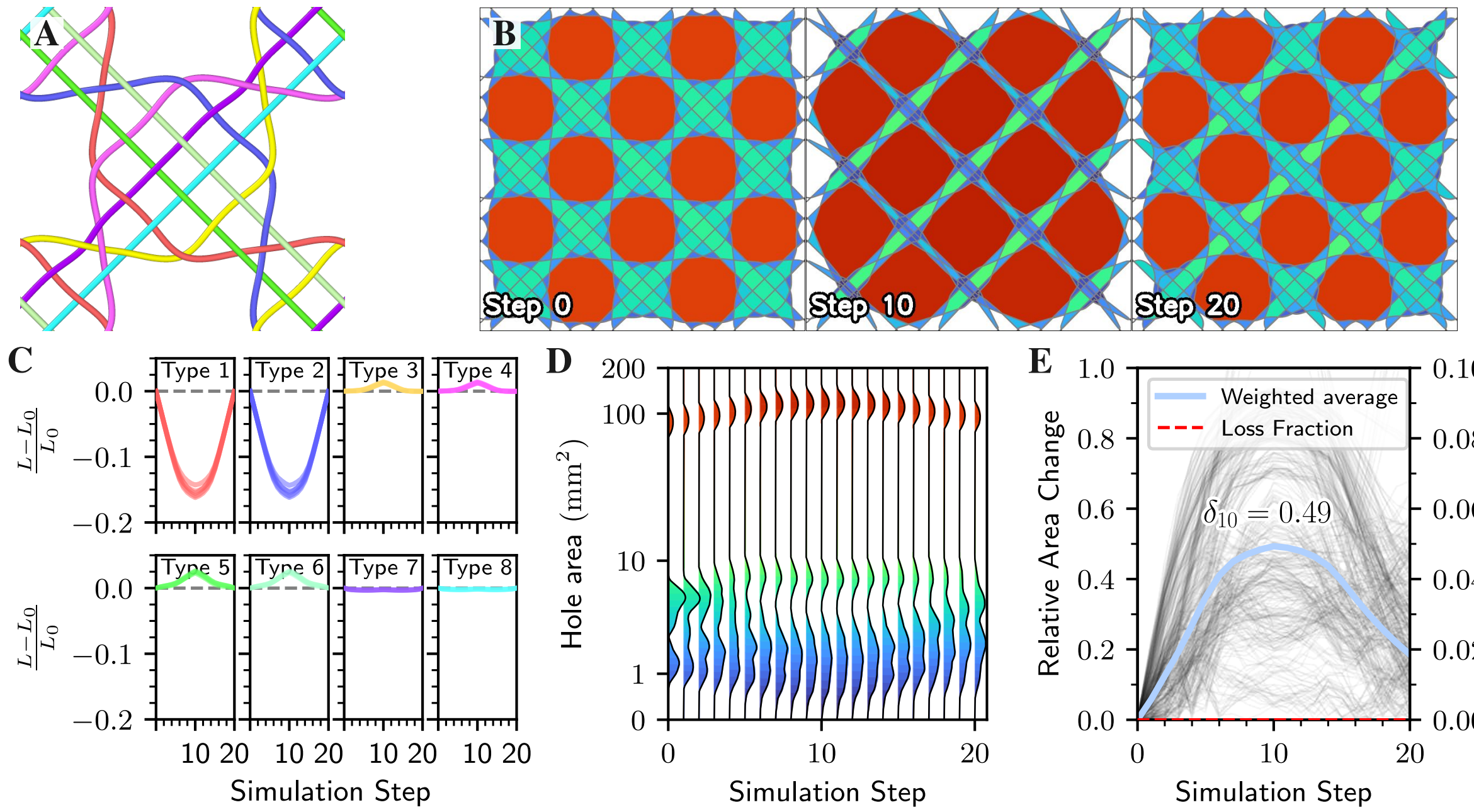

Figure S128: **Simulation of lace structure Rose-6. A** Unit-cell schematic (active bond types {1, 2, 7, 8}). **B** Snapshots at steps 0, 10, and 20, showing the initial structure, maximum deformation (active bonds shrink by 25%), and subsequent relaxation. **C** Variation of the relative length of yarns, grouped by type. **D** Evolution of the distribution of pore areas over time. **E** Relative area change of individual pores.

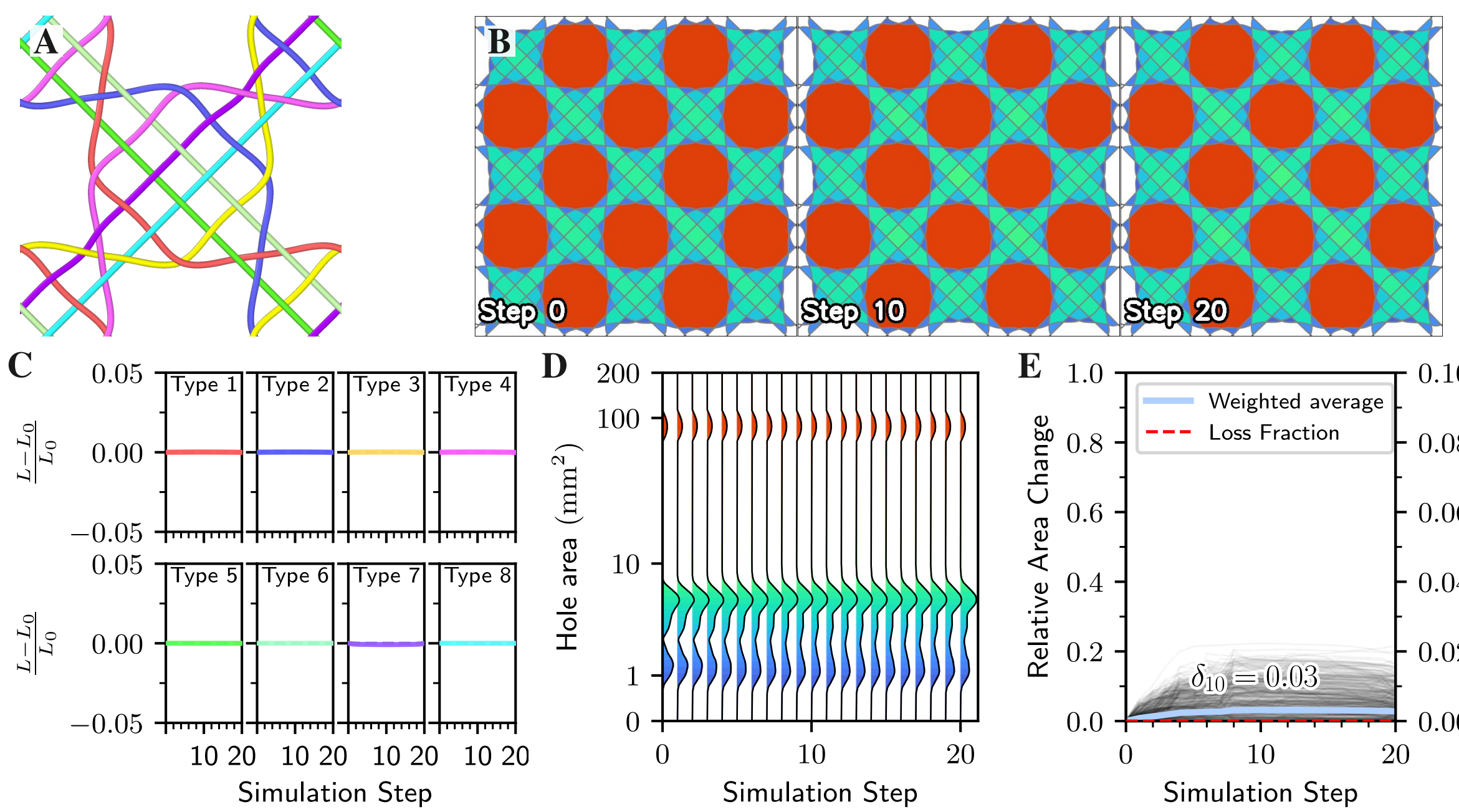

Figure S129: **Simulation of lace structure Rose-6. A** Unit-cell schematic (active bond types {5, 6, 7, 8}). **B** Snapshots at steps 0, 10, and 20, showing the initial structure, maximum deformation (active bonds shrink by 25%), and subsequent relaxation. **C** Variation of the relative length of yarns, grouped by type. **D** Evolution of the distribution of pore areas over time. **E** Relative area change of individual pores.

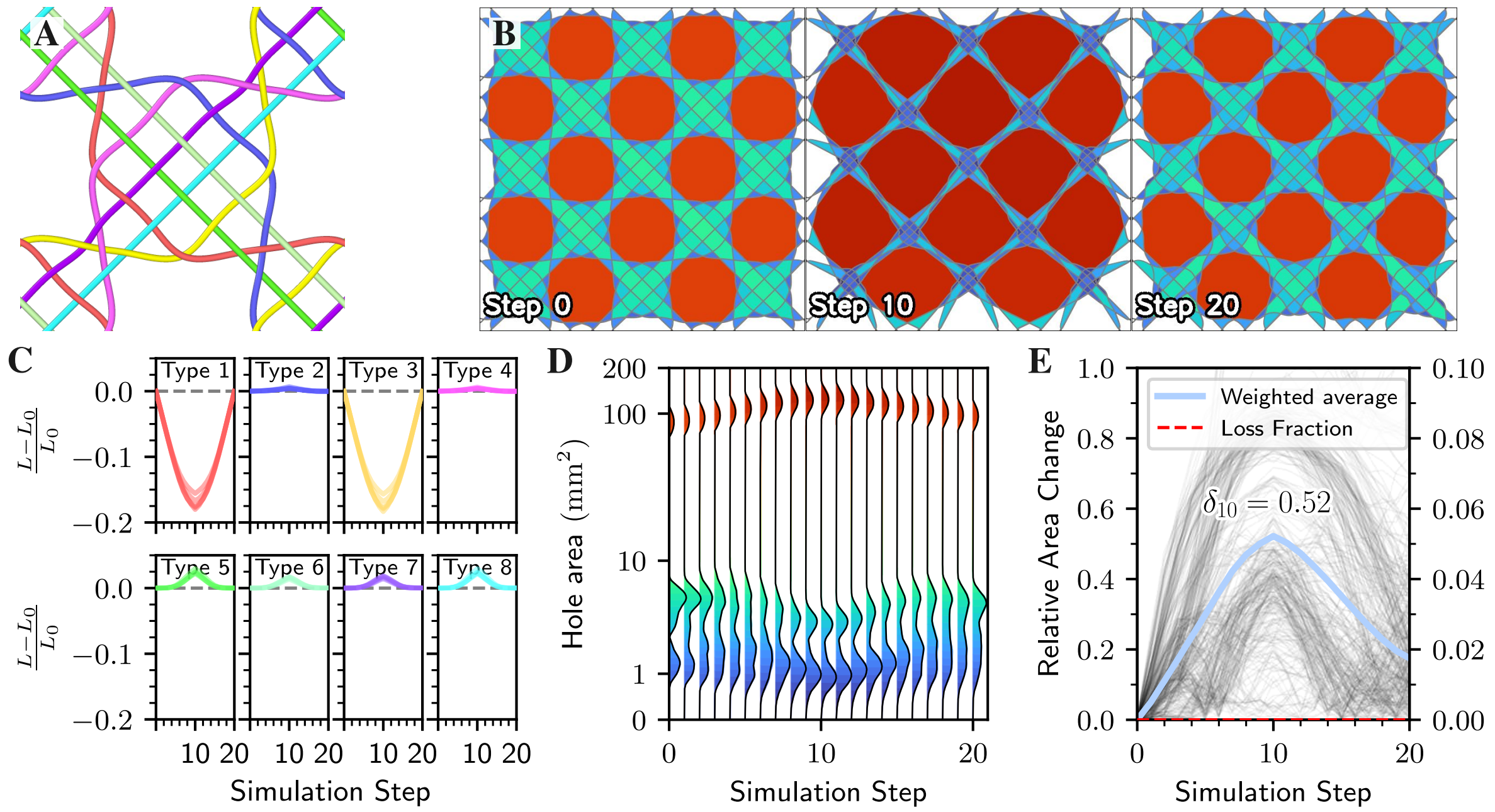

Figure S130: **Simulation of lace structure Rose-6.** **A** Unit-cell schematic (active bond types {1, 3}). **B** Snapshots at steps 0, 10, and 20, showing the initial structure, maximum deformation (active bonds shrink by 25%), and subsequent relaxation. **C** Variation of the relative length of yarns, grouped by type. **D** Evolution of the distribution of pore areas over time. **E** Relative area change of individual pores.

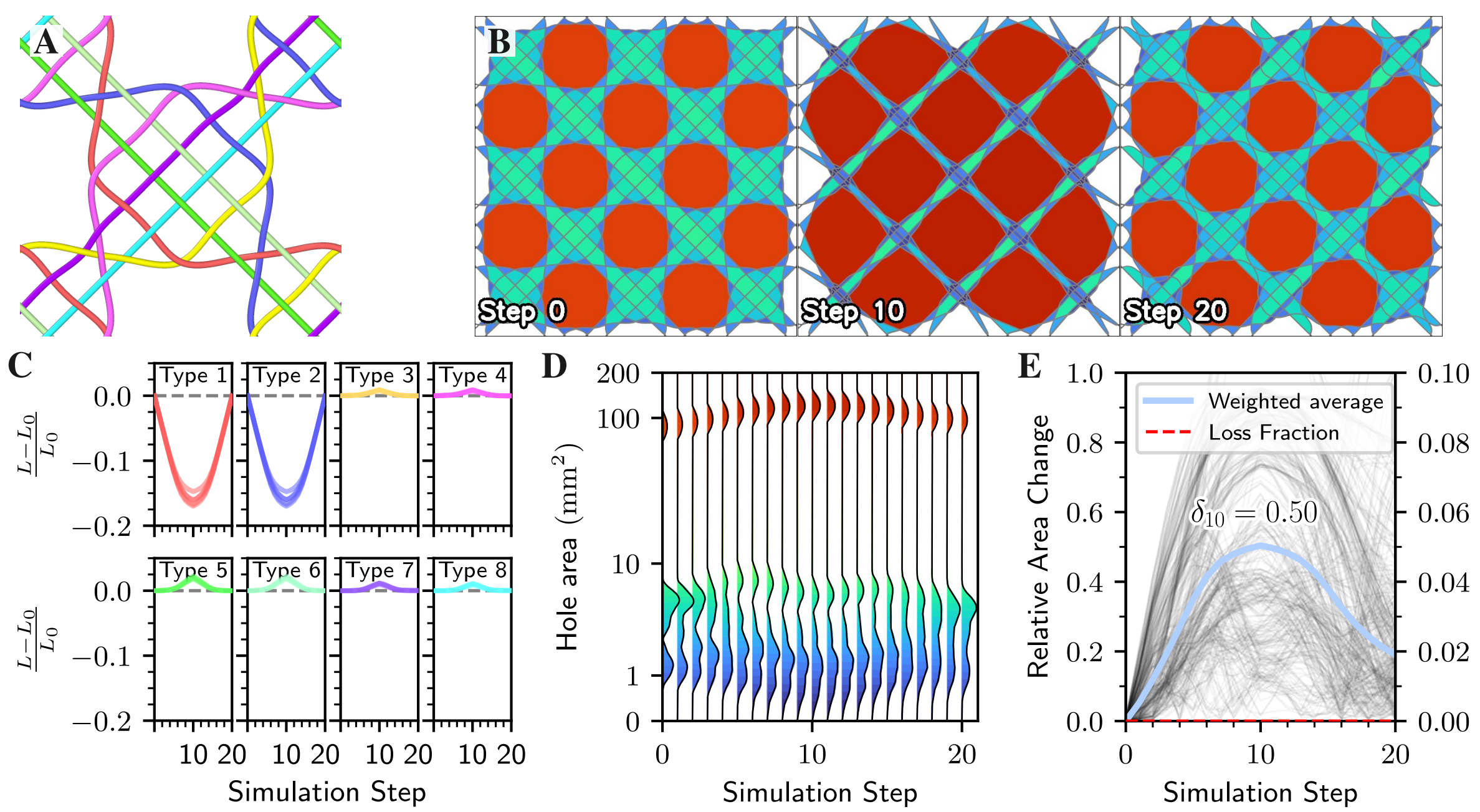

Figure S131: **Simulation of lace structure Rose-6.** **A** Unit-cell schematic (active bond types {1, 2}). **B** Snapshots at steps 0, 10, and 20, showing the initial structure, maximum deformation (active bonds shrink by 25%), and subsequent relaxation. **C** Variation of the relative length of yarns, grouped by type. **D** Evolution of the distribution of pore areas over time. **E** Relative area change of individual pores.

Rose-7

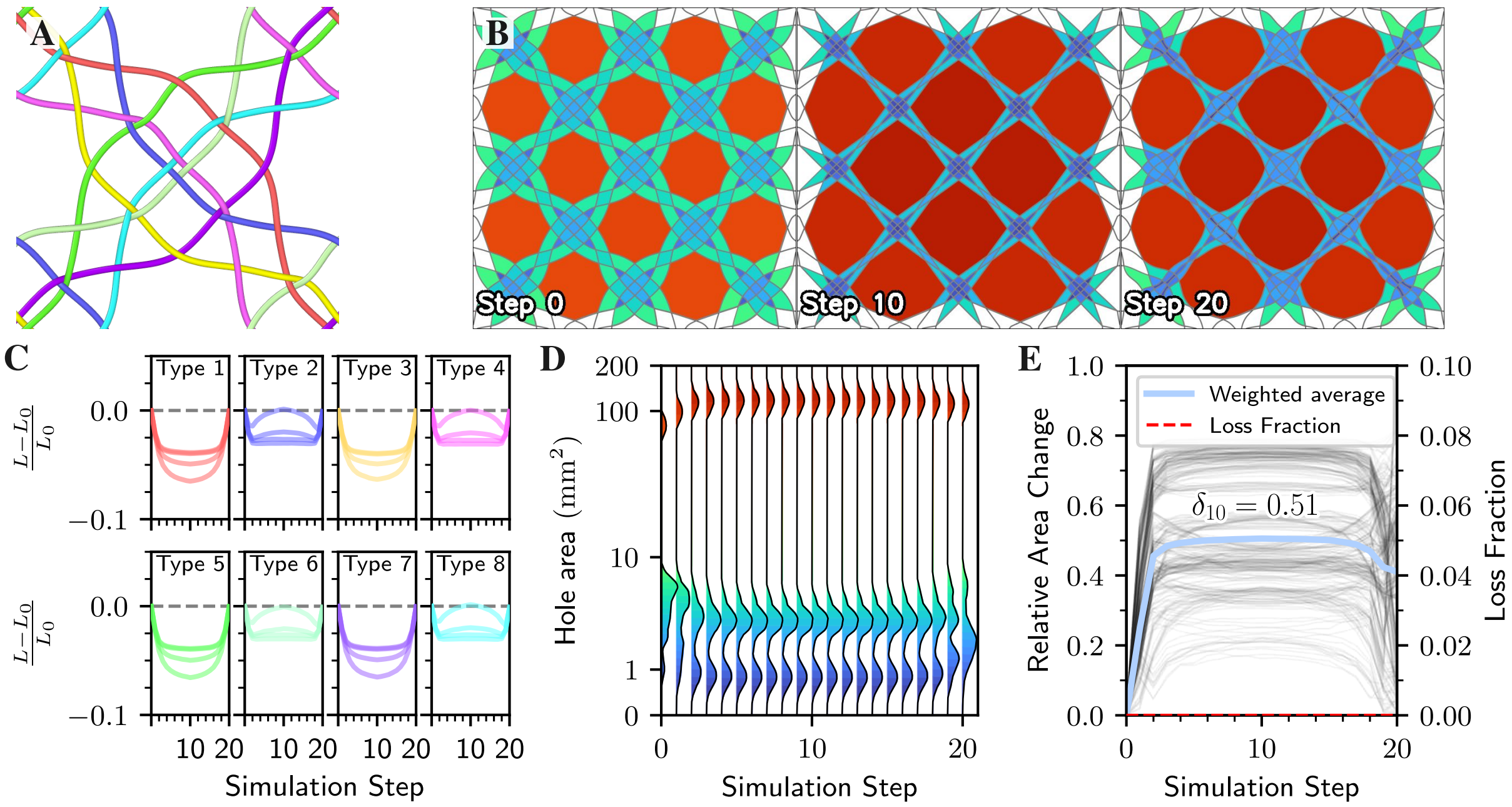

Figure S132: **Simulation of lace structure Rose-7. A** Unit-cell schematic (active bond types {1, 2, 3, 4, 5, 6, 7, 8}). **B** Snapshots at steps 0, 10, and 20, showing the initial structure, maximum deformation (active bonds shrink by 25%), and subsequent relaxation. **C** Variation of the relative length of yarns, grouped by type. **D** Evolution of the distribution of pore areas over time. **E** Relative area change of individual pores.

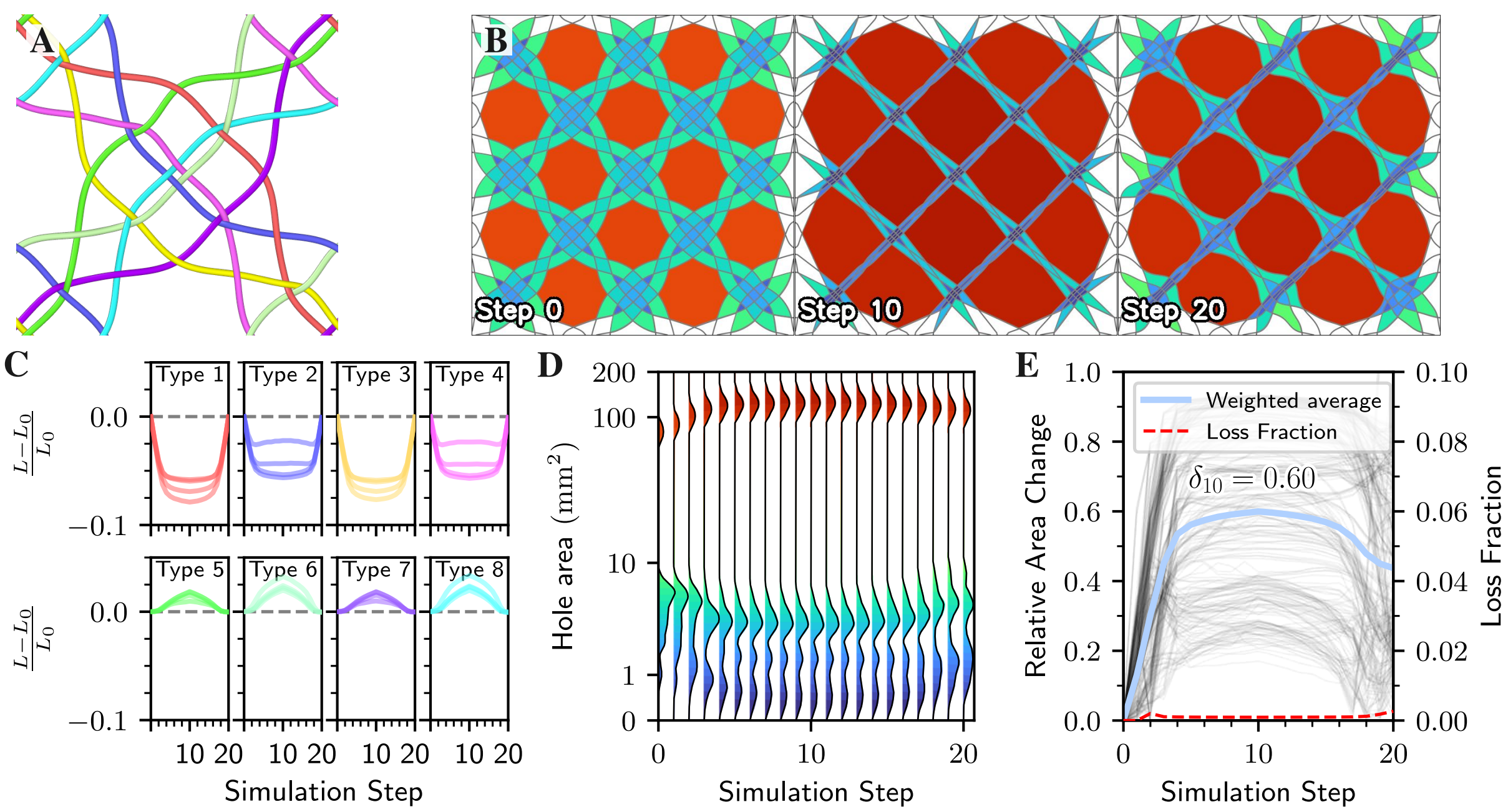

Figure S133: **Simulation of lace structure Rose-7. A** Unit-cell schematic (active bond types {1, 2, 3, 4}). **B** Snapshots at steps 0, 10, and 20, showing the initial structure, maximum deformation (active bonds shrink by 25%), and subsequent relaxation. **C** Variation of the relative length of yarns, grouped by type. **D** Evolution of the distribution of pore areas over time. **E** Relative area change of individual pores.

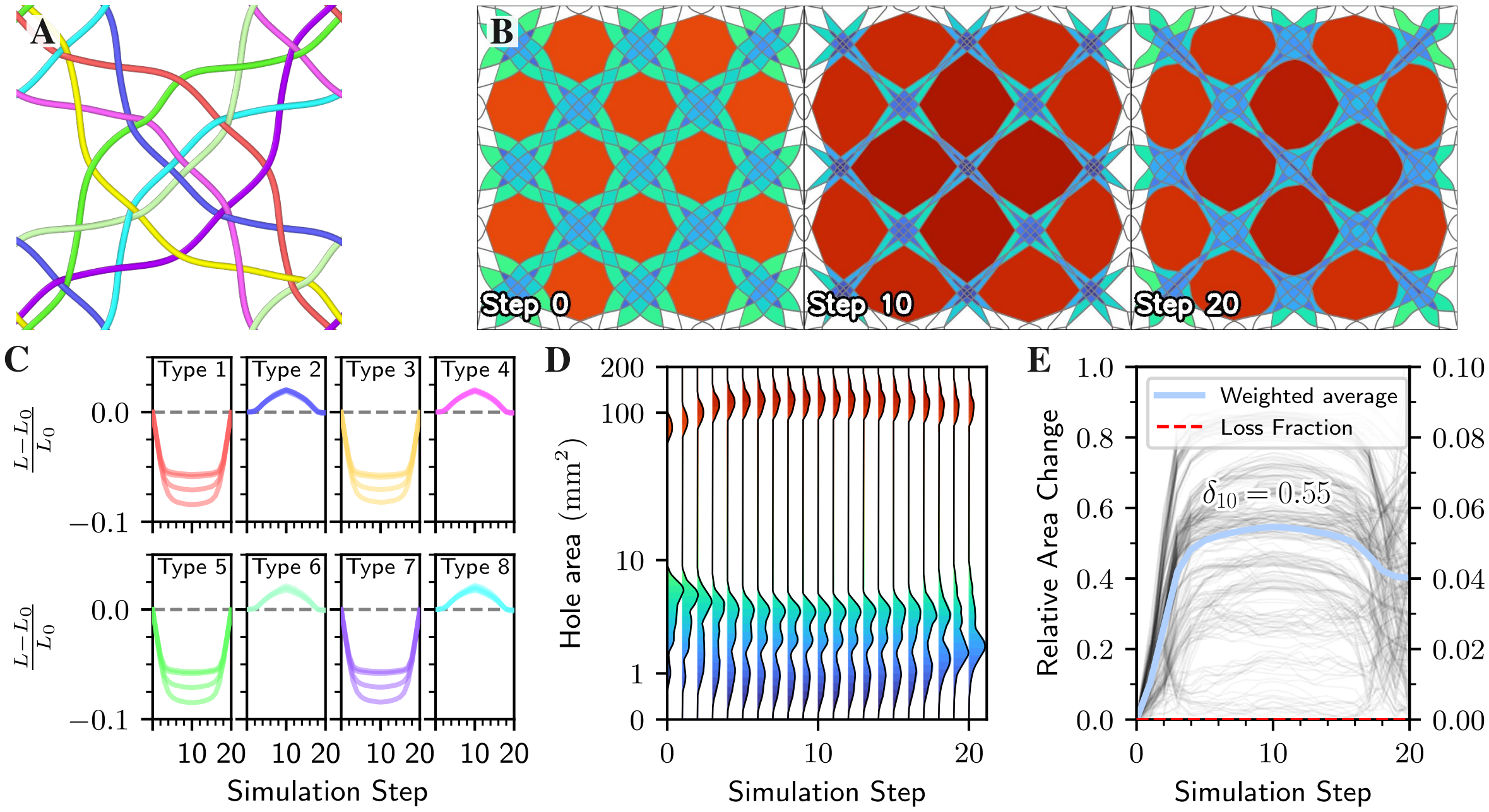

Figure S134: **Simulation of lace structure Rose-7. A** Unit-cell schematic (active bond types {1, 3, 5, 7}). **B** Snapshots at steps 0, 10, and 20, showing the initial structure, maximum deformation (active bonds shrink by 25%), and subsequent relaxation. **C** Variation of the relative length of yarns, grouped by type. **D** Evolution of the distribution of pore areas over time. **E** Relative area change of individual pores.

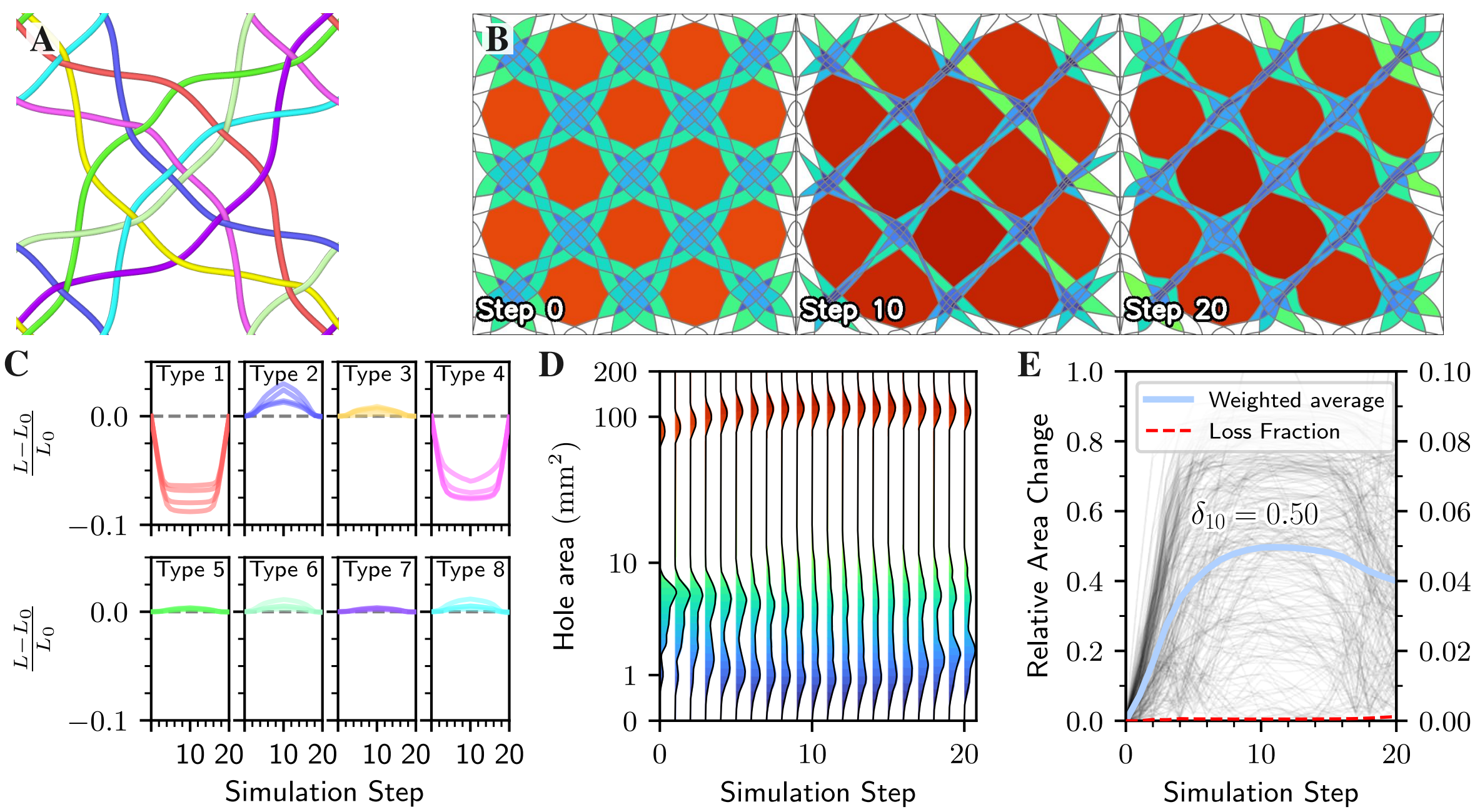

Figure S135: **Simulation of lace structure Rose-7. A** Unit-cell schematic (active bond types {1, 4}). **B** Snapshots at steps 0, 10, and 20, showing the initial structure, maximum deformation (active bonds shrink by 25%), and subsequent relaxation. **C** Variation of the relative length of yarns, grouped by type. **D** Evolution of the distribution of pore areas over time. **E** Relative area change of individual pores.

Rose-8

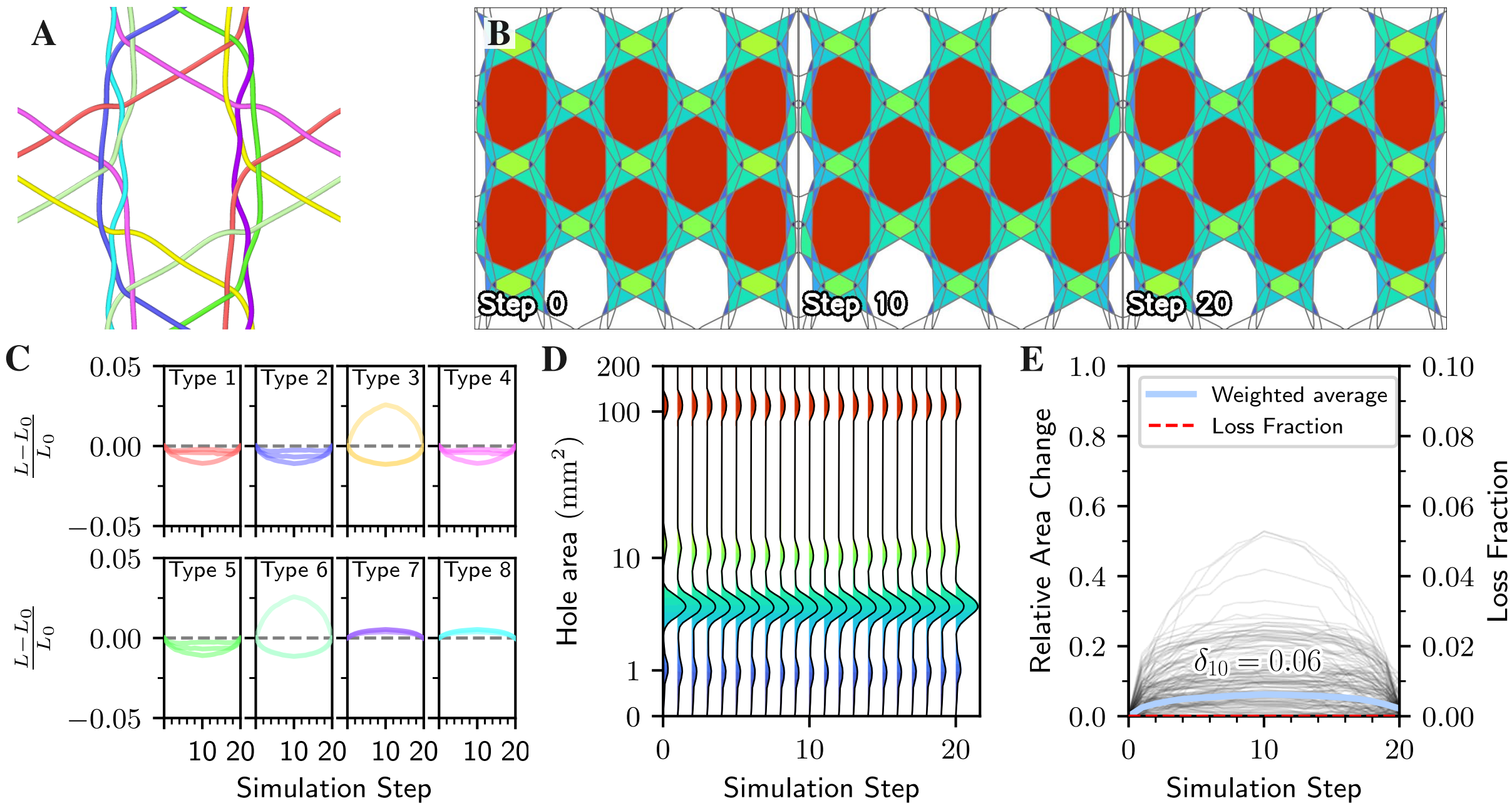

Figure S136: **Simulation of lace structure Rose-8. A** Unit-cell schematic (active bond types {1, 2, 3, 4, 5, 6, 7, 8}). **B** Snapshots at steps 0, 10, and 20, showing the initial structure, maximum deformation (active bonds shrink by 25%), and subsequent relaxation. **C** Variation of the relative length of yarns, grouped by type. **D** Evolution of the distribution of pore areas over time. **E** Relative area change of individual pores.

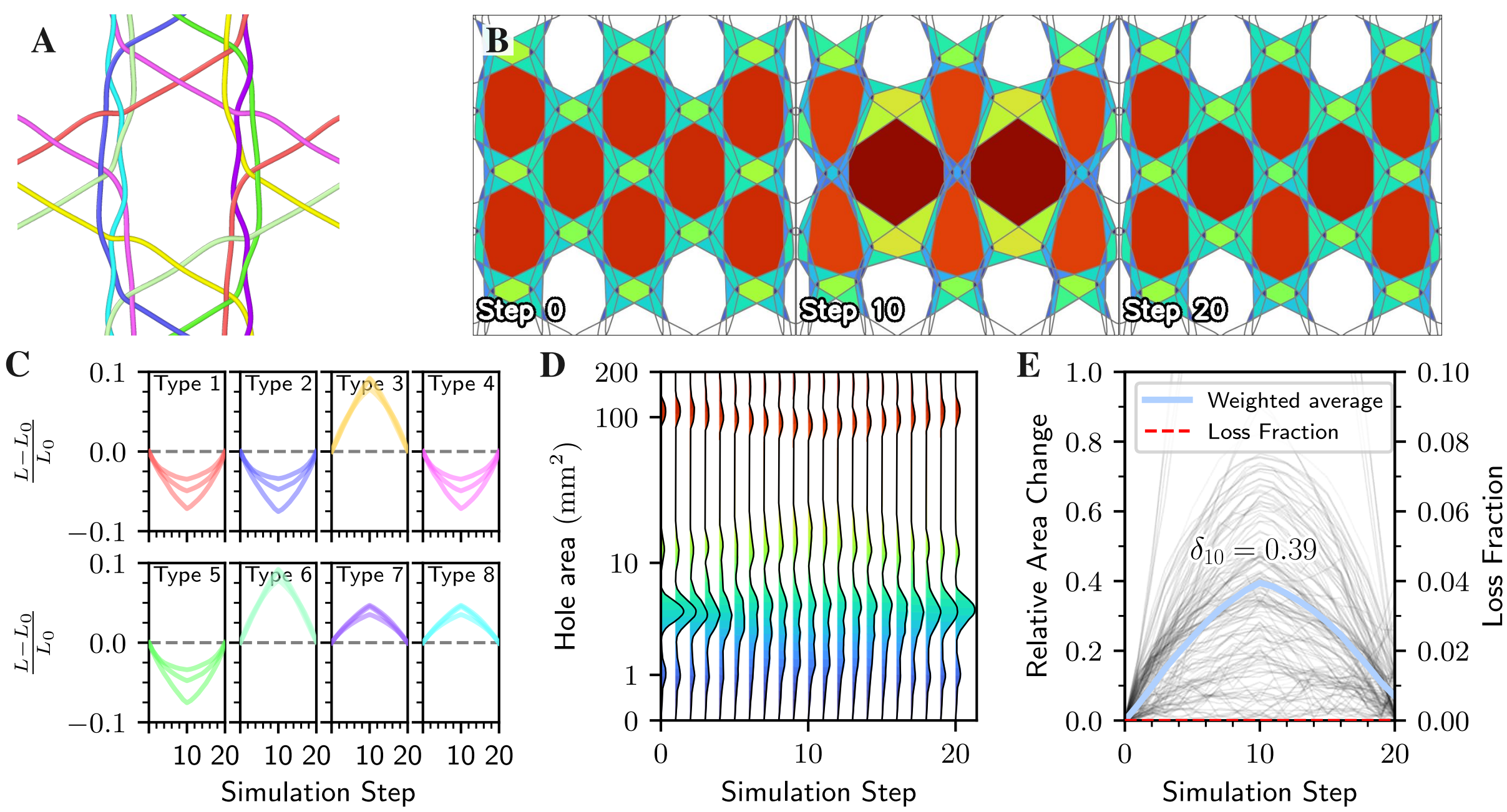

Figure S137: **Simulation of lace structure Rose-8. A** Unit-cell schematic (active bond types {1, 2, 4, 5}). **B** Snapshots at steps 0, 10, and 20, showing the initial structure, maximum deformation (active bonds shrink by 25%), and subsequent relaxation. **C** Variation of the relative length of yarns, grouped by type. **D** Evolution of the distribution of pore areas over time. **E** Relative area change of individual pores.

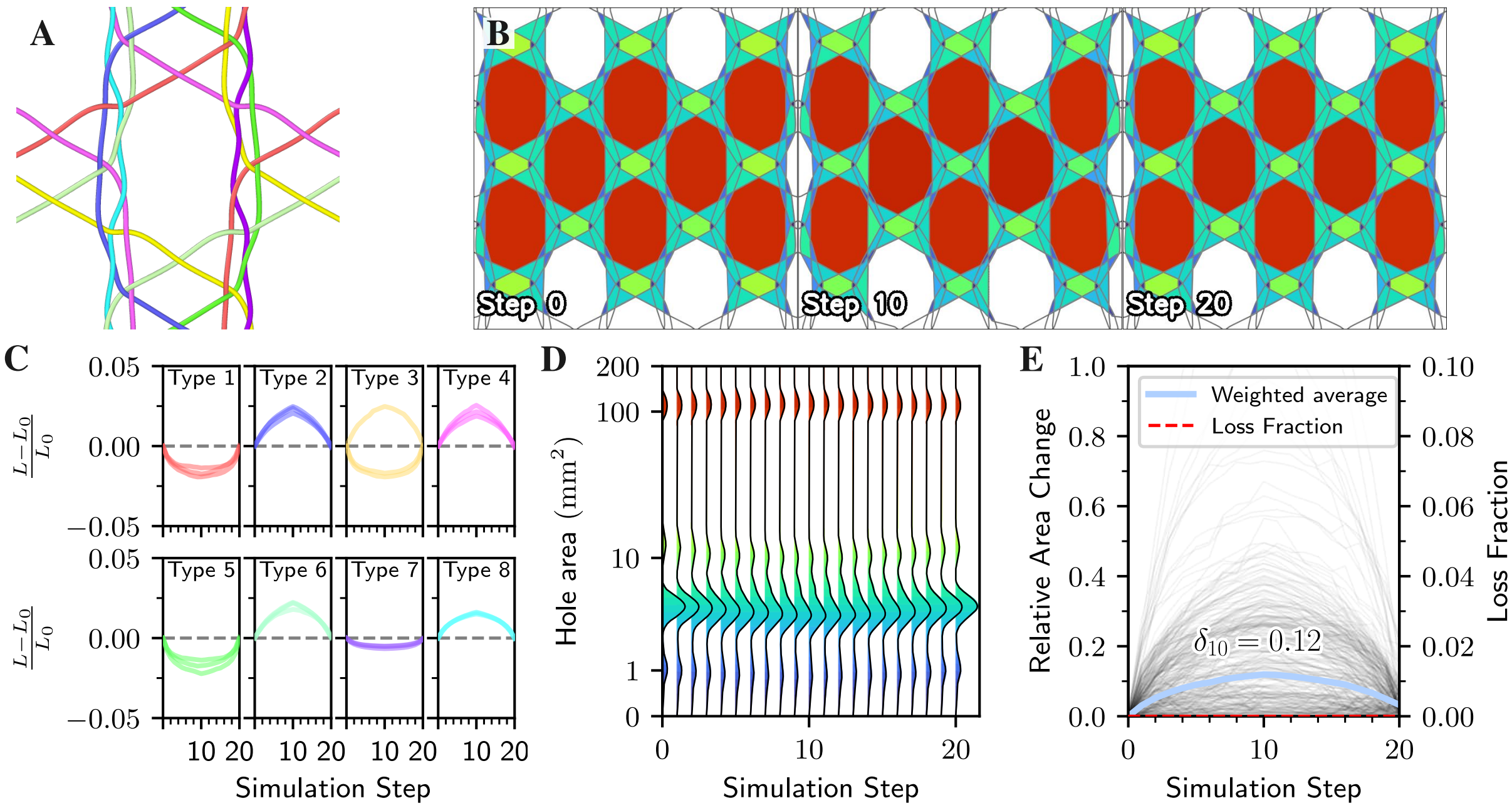

Figure S138: **Simulation of lace structure Rose-8.** **A** Unit-cell schematic (active bond types {1, 3, 5, 7}). **B** Snapshots at steps 0, 10, and 20, showing the initial structure, maximum deformation (active bonds shrink by 25%), and subsequent relaxation. **C** Variation of the relative length of yarns, grouped by type. **D** Evolution of the distribution of pore areas over time. **E** Relative area change of individual pores.

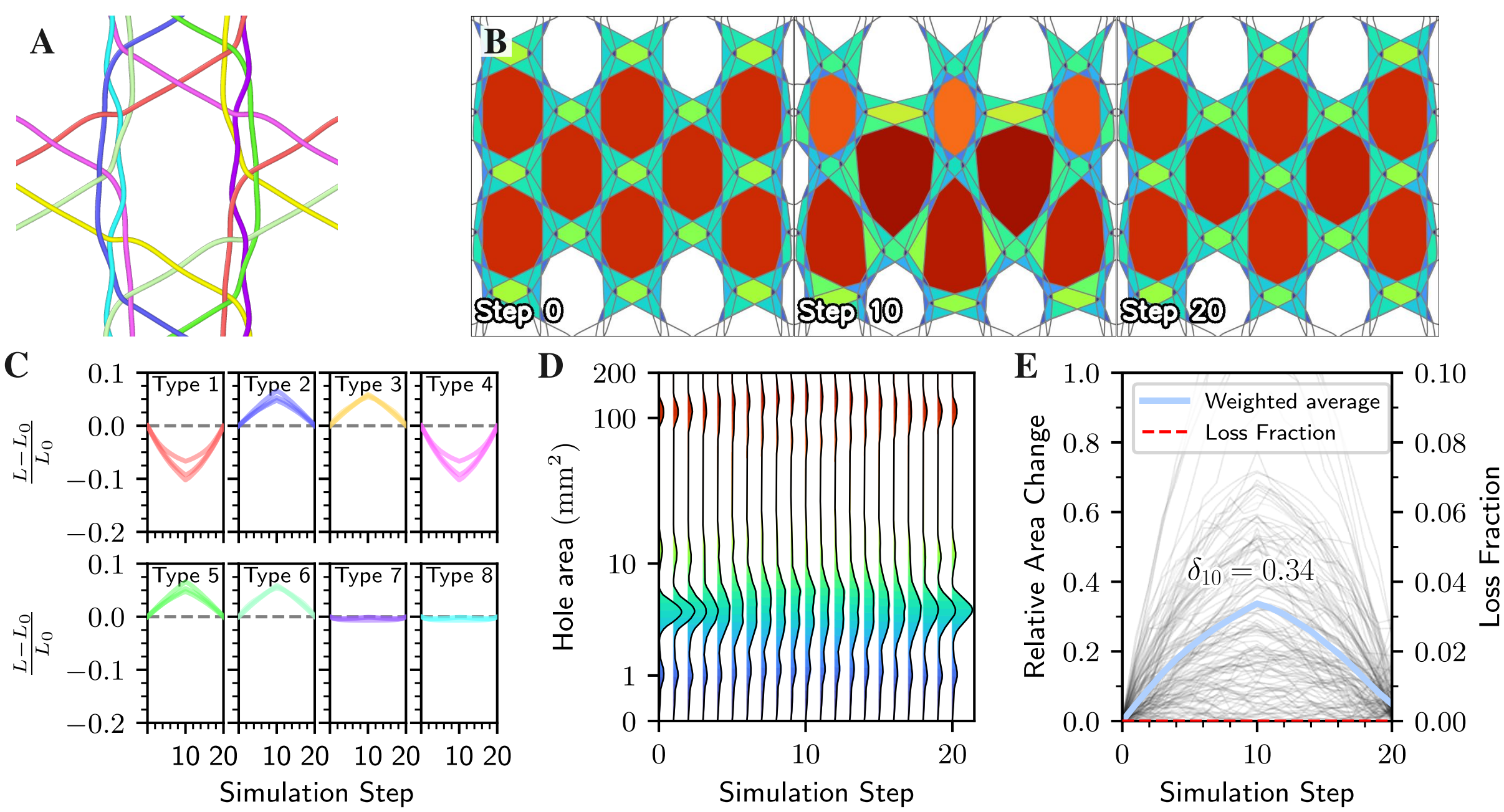

Figure S139: **Simulation of lace structure Rose-8.** **A** Unit-cell schematic (active bond types {1, 4, 7, 8}). **B** Snapshots at steps 0, 10, and 20, showing the initial structure, maximum deformation (active bonds shrink by 25%), and subsequent relaxation. **C** Variation of the relative length of yarns, grouped by type. **D** Evolution of the distribution of pore areas over time. **E** Relative area change of individual pores.

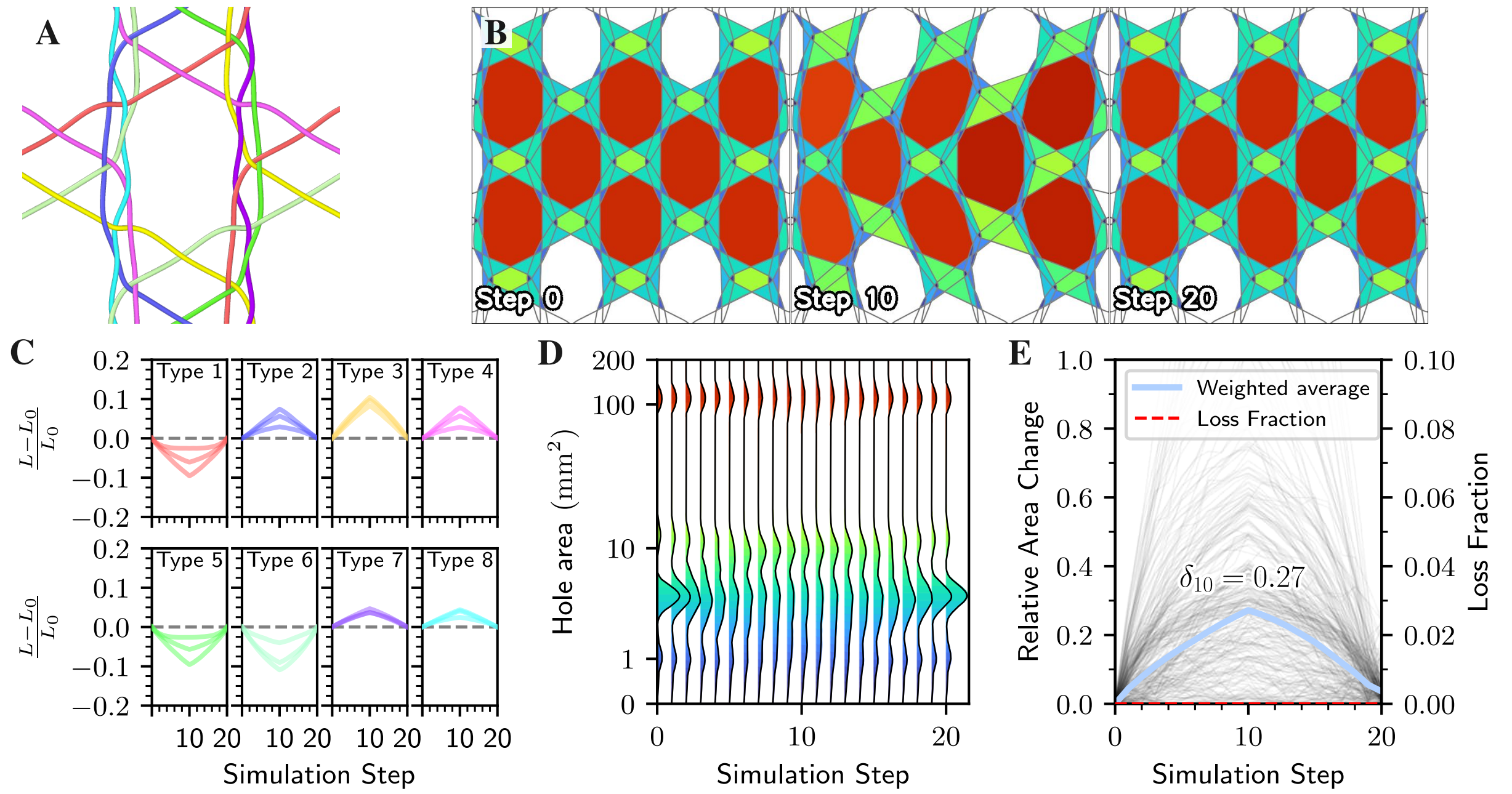

Figure S140: **Simulation of lace structure Rose-8. A** Unit-cell schematic (active bond types {1, 5, 6}). **B** Snapshots at steps 0, 10, and 20, showing the initial structure, maximum deformation (active bonds shrink by 25%), and subsequent relaxation. **C** Variation of the relative length of yarns, grouped by type. **D** Evolution of the distribution of pore areas over time. **E** Relative area change of individual pores.

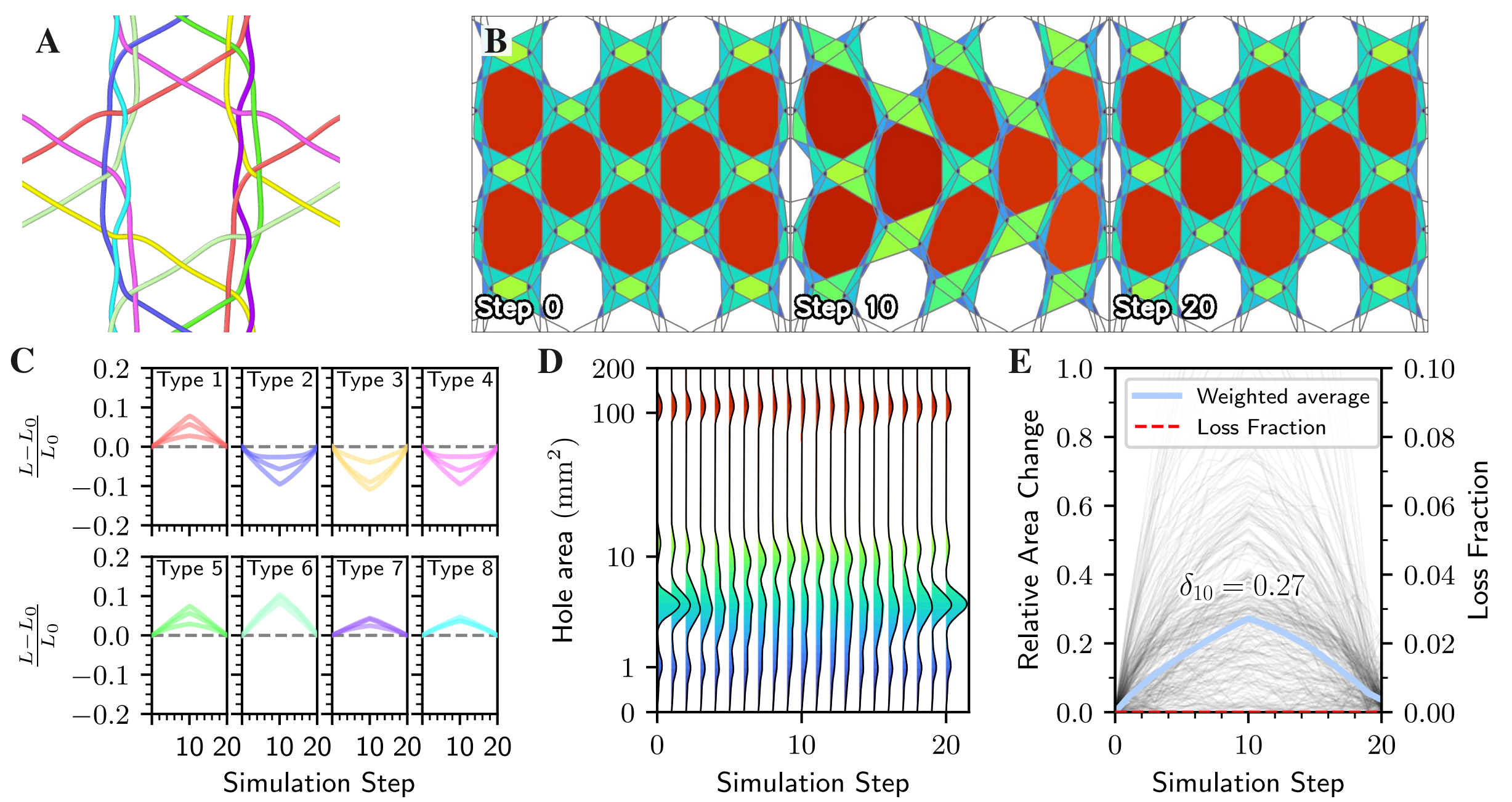

Figure S141: **Simulation of lace structure Rose-8. A** Unit-cell schematic (active bond types {2, 3, 4}). **B** Snapshots at steps 0, 10, and 20, showing the initial structure, maximum deformation (active bonds shrink by 25%), and subsequent relaxation. **C** Variation of the relative length of yarns, grouped by type. **D** Evolution of the distribution of pore areas over time. **E** Relative area change of individual pores.

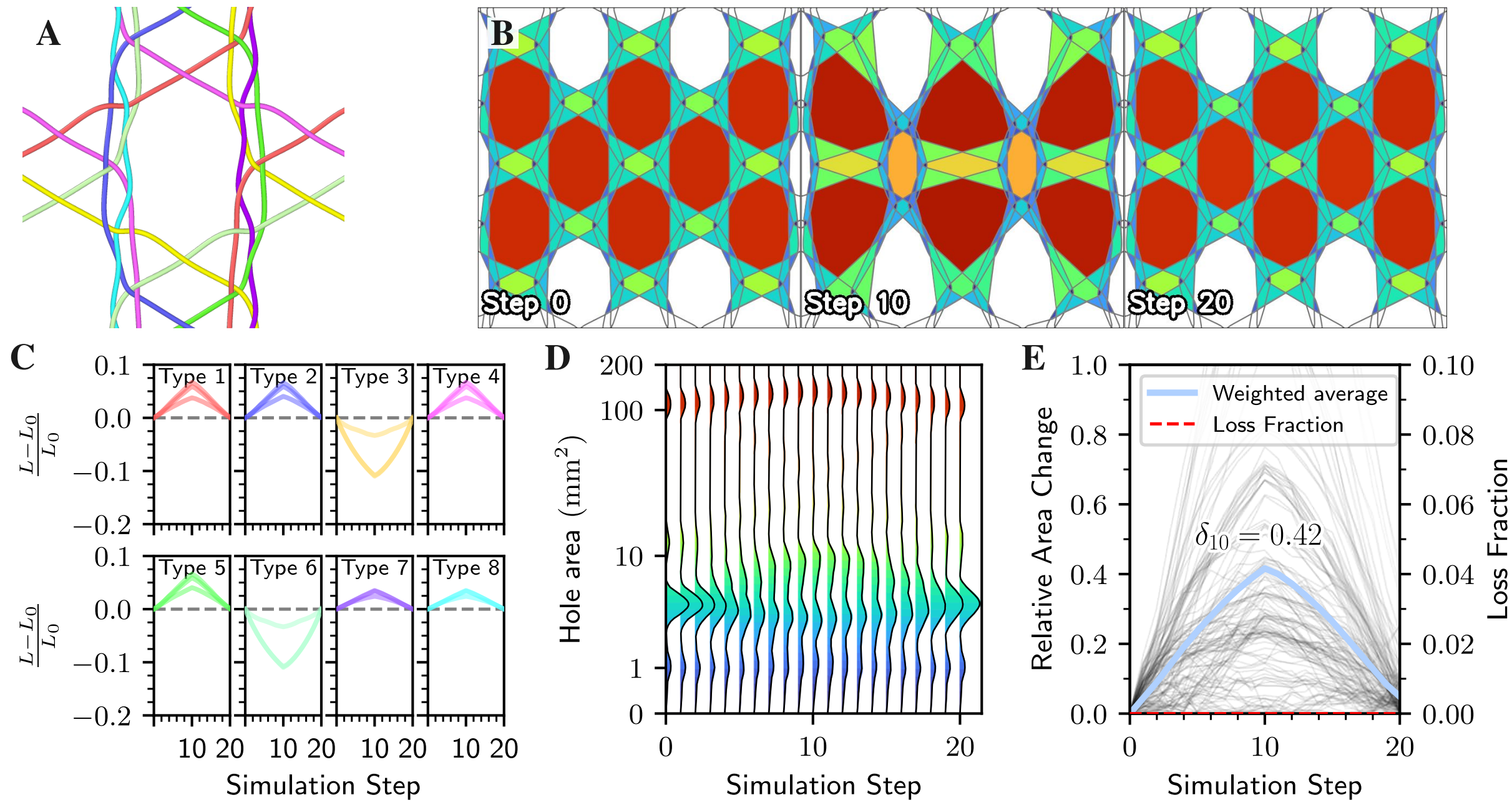

Figure S142: **Simulation of lace structure Rose-8. A** Unit-cell schematic (active bond types {3, 6}). **B** Snapshots at steps 0, 10, and 20, showing the initial structure, maximum deformation (active bonds shrink by 25%), and subsequent relaxation. **C** Variation of the relative length of yarns, grouped by type. **D** Evolution of the distribution of pore areas over time. **E** Relative area change of individual pores.

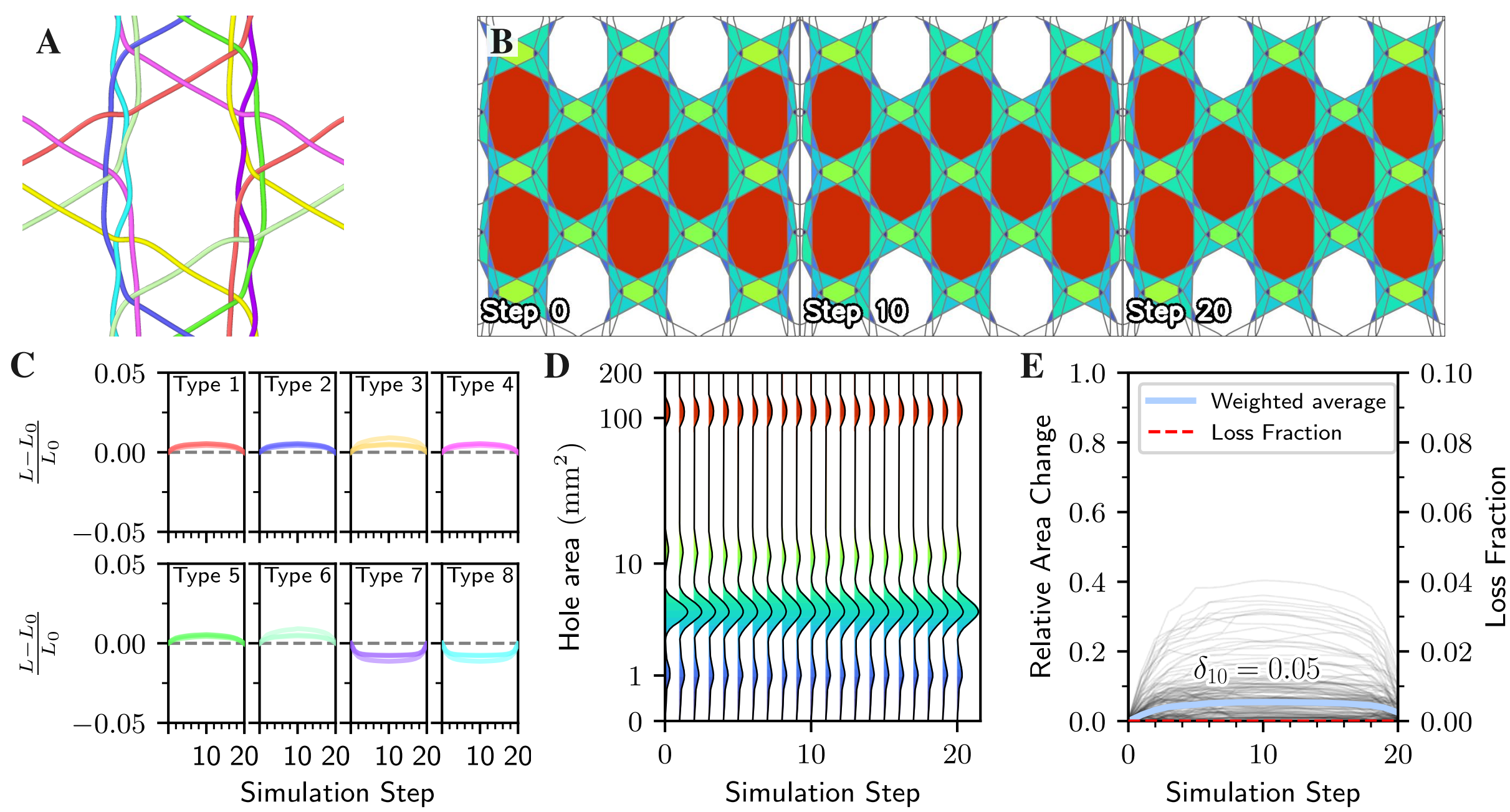

Figure S143: **Simulation of lace structure Rose-8. A** Unit-cell schematic (active bond types {7, 8}). **B** Snapshots at steps 0, 10, and 20, showing the initial structure, maximum deformation (active bonds shrink by 25%), and subsequent relaxation. **C** Variation of the relative length of yarns, grouped by type. **D** Evolution of the distribution of pore areas over time. **E** Relative area change of individual pores.

Rose-9

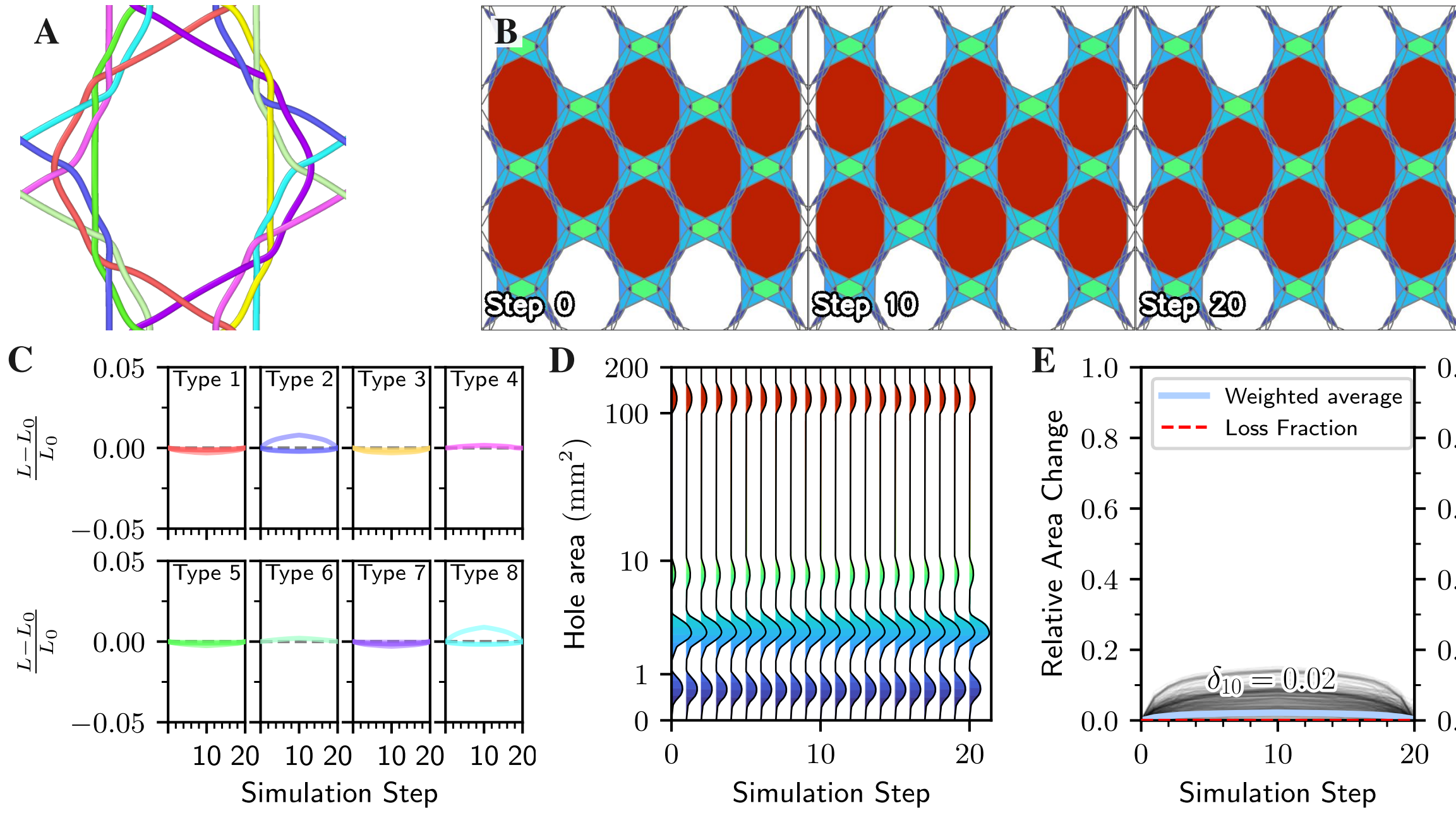

Figure S144: **Simulation of lace structure Rose-9. A** Unit-cell schematic (active bond types {1, 2, 3, 4, 5, 6, 7, 8}). **B** Snapshots at steps 0, 10, and 20, showing the initial structure, maximum deformation (active bonds shrink by 25%), and subsequent relaxation. **C** Variation of the relative length of yarns, grouped by type. **D** Evolution of the distribution of pore areas over time. **e** Relative area change of individual pores.

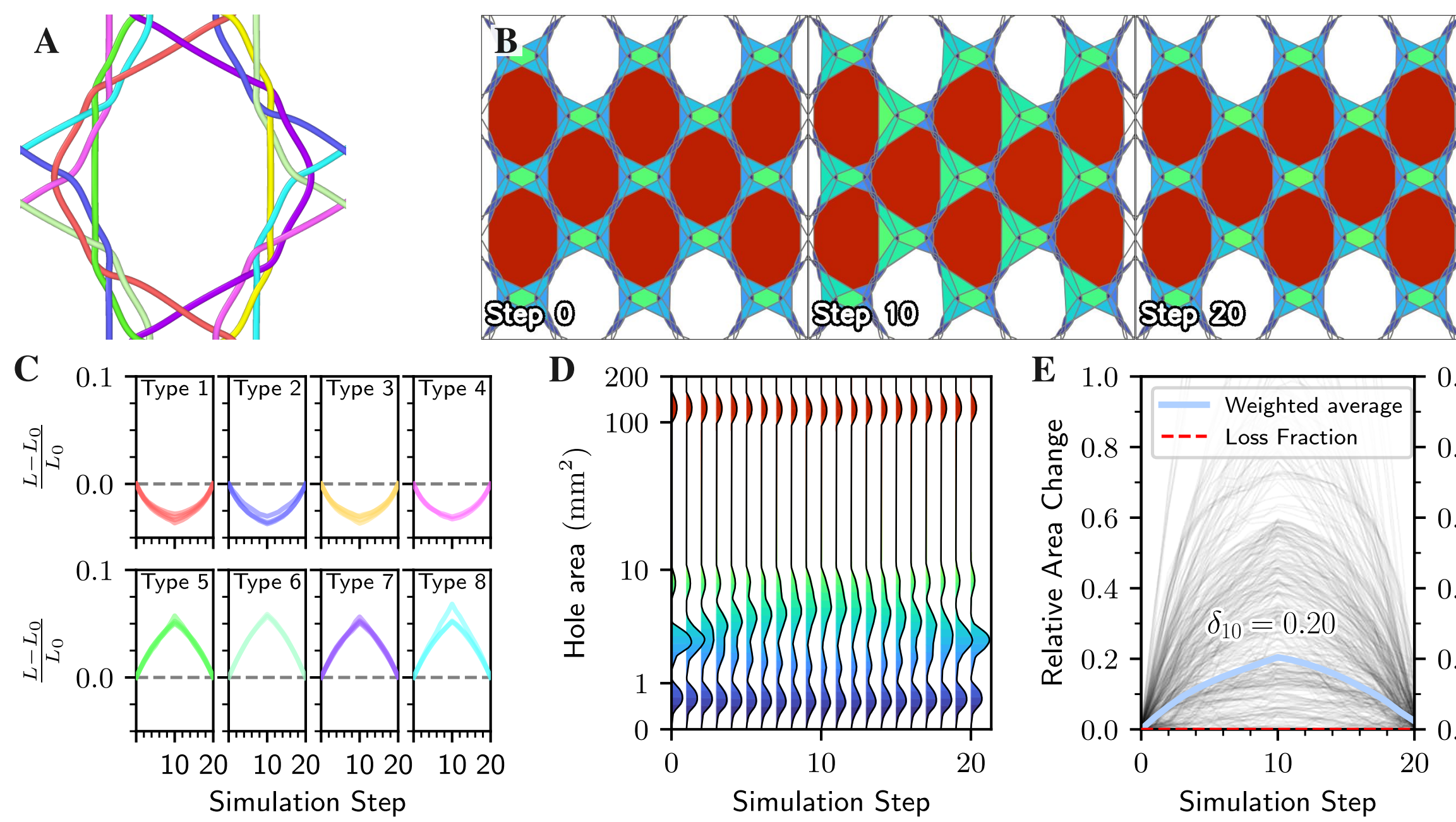

Figure S145: **Simulation of lace structure Rose-9. A** Unit-cell schematic (active bond types {1, 2, 3, 4}). **B** Snapshots at steps 0, 10, and 20, showing the initial structure, maximum deformation (active bonds shrink by 25%), and subsequent relaxation. **C** Variation of the relative length of yarns, grouped by type. **D** Evolution of the distribution of pore areas over time. **e** Relative area change of individual pores.

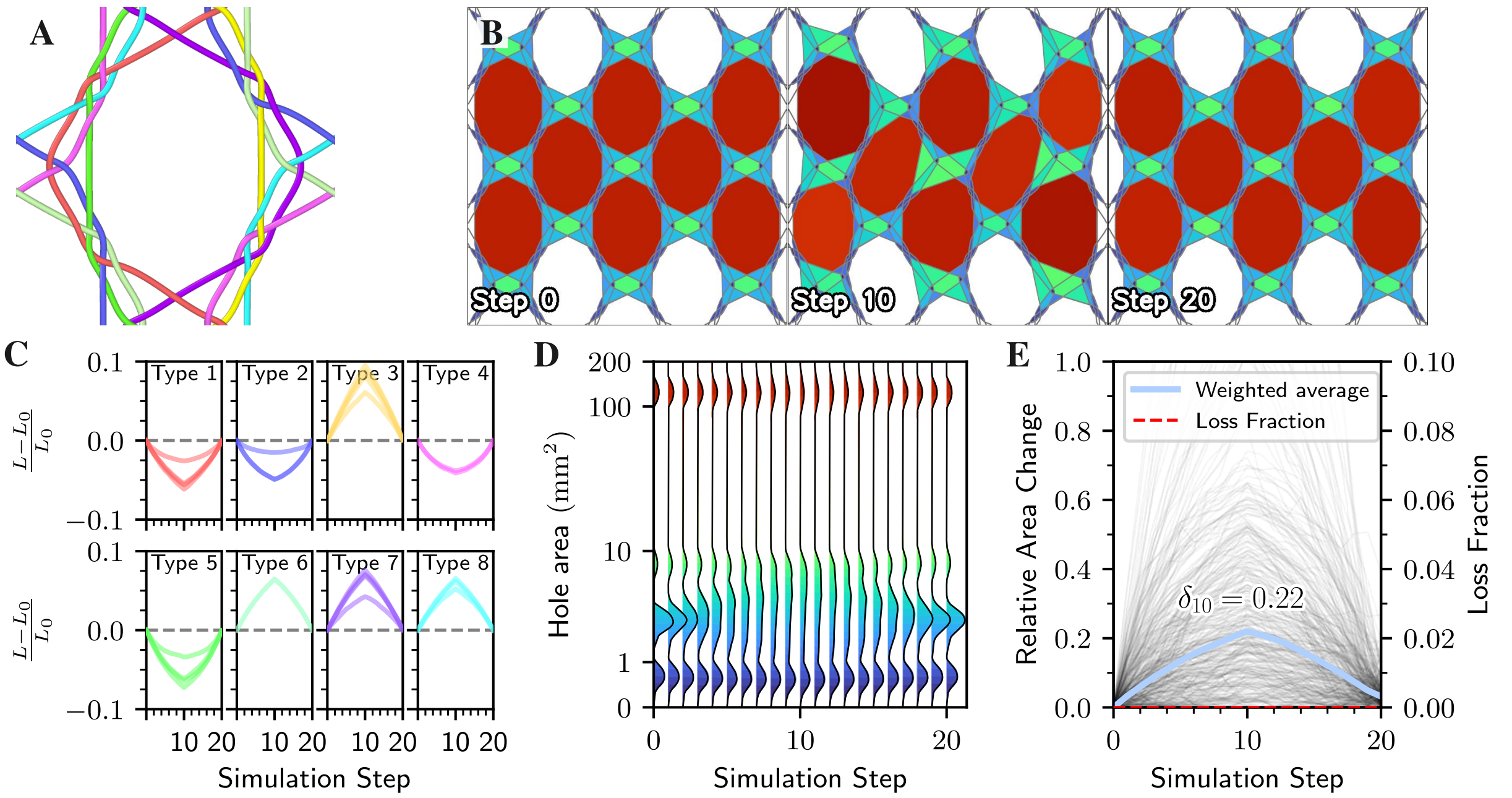

Figure S146: **Simulation of lace structure Rose-9. A** Unit-cell schematic (active bond types {1, 2, 4, 5}). **B** Snapshots at steps 0, 10, and 20, showing the initial structure, maximum deformation (active bonds shrink by 25%), and subsequent relaxation. **C** Variation of the relative length of yarns, grouped by type. **D** Evolution of the distribution of pore areas over time. **e** Relative area change of individual pores.

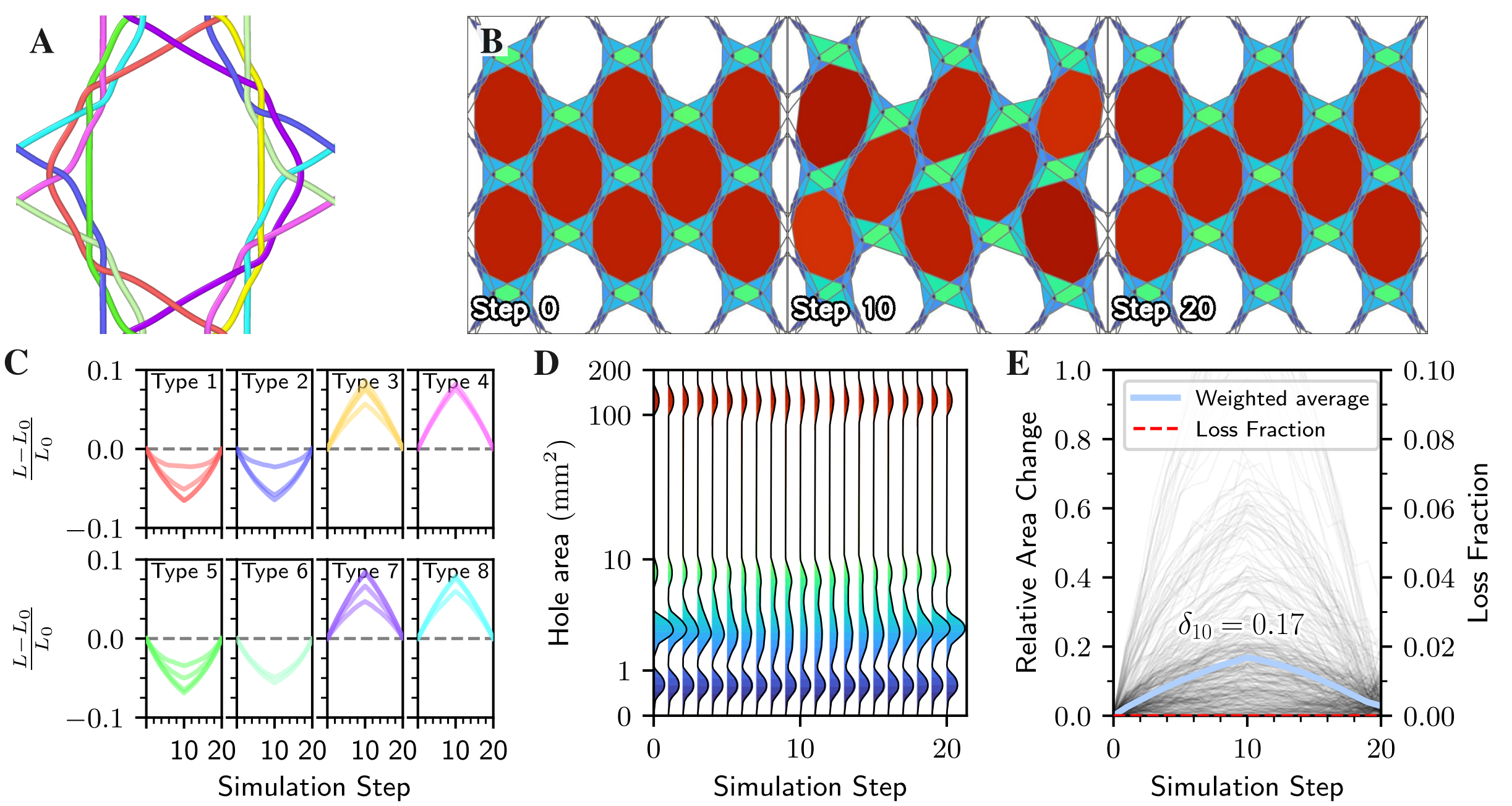

Figure S147: **Simulation of lace structure Rose-9. A** Unit-cell schematic (active bond types {1, 2, 5, 6}). **B** Snapshots at steps 0, 10, and 20, showing the initial structure, maximum deformation (active bonds shrink by 25%), and subsequent relaxation. **C** Variation of the relative length of yarns, grouped by type. **D** Evolution of the distribution of pore areas over time. **e** Relative area change of individual pores.

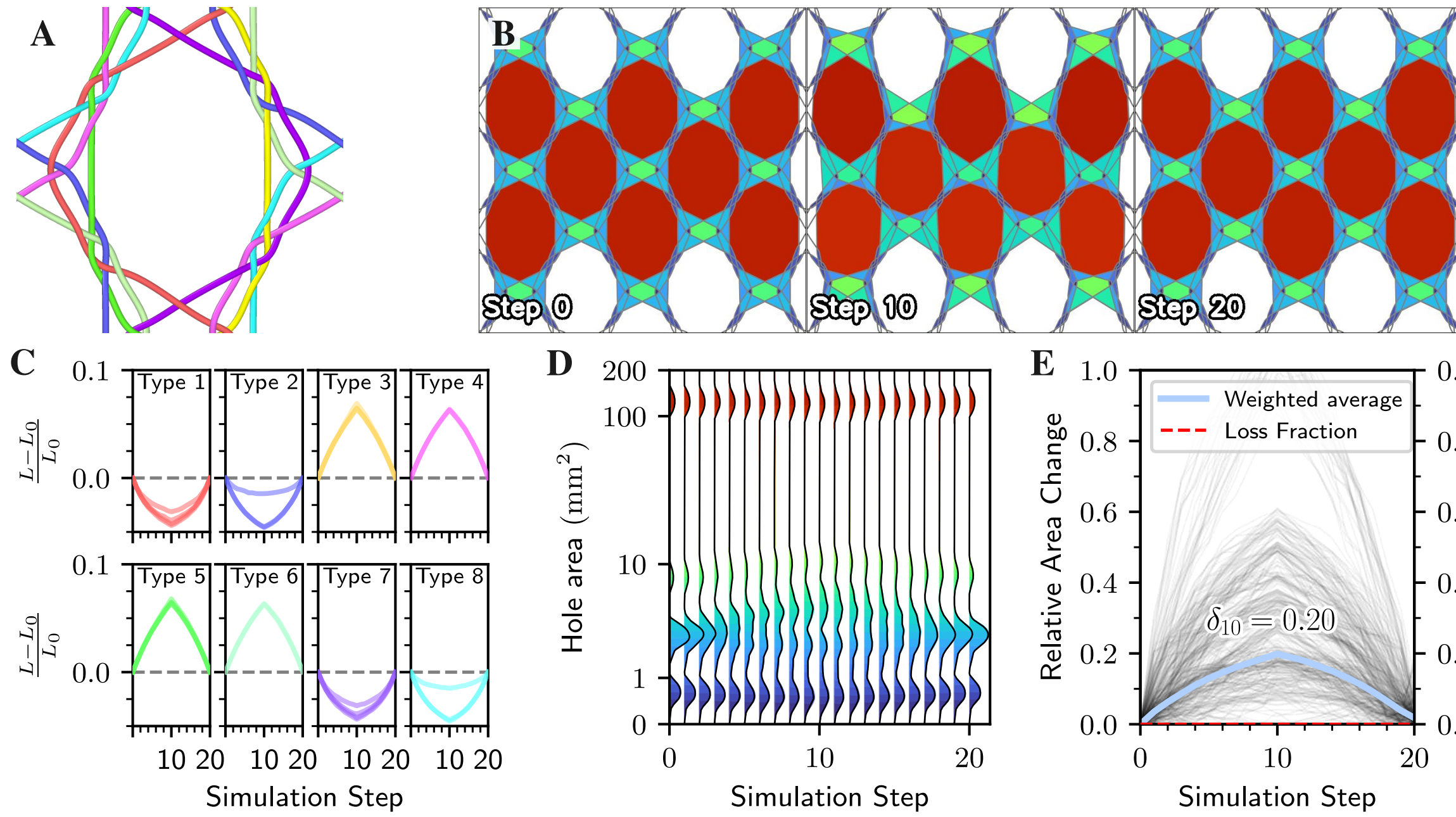

Figure S148: **Simulation of lace structure Rose-9. A** Unit-cell schematic (active bond types {1, 2, 7, 8}). **B** Snapshots at steps 0, 10, and 20, showing the initial structure, maximum deformation (active bonds shrink by 25%), and subsequent relaxation. **C** Variation of the relative length of yarns, grouped by type. **D** Evolution of the distribution of pore areas over time. **e** Relative area change of individual pores.

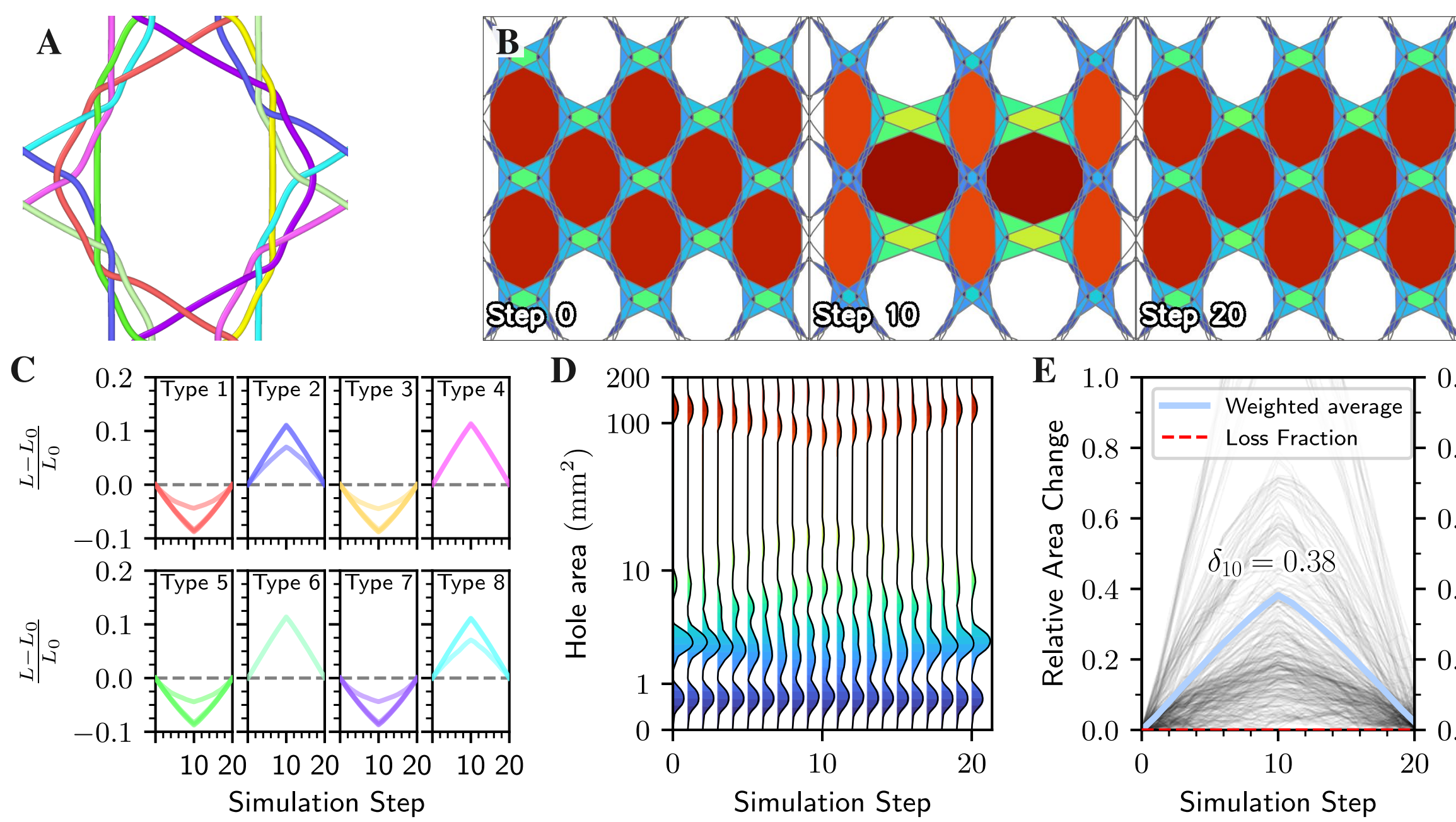

Figure S149: **Simulation of lace structure Rose-9. A** Unit-cell schematic (active bond types {1, 3, 5, 7}). **B** Snapshots at steps 0, 10, and 20, showing the initial structure, maximum deformation (active bonds shrink by 25%), and subsequent relaxation. **C** Variation of the relative length of yarns, grouped by type. **D** Evolution of the distribution of pore areas over time. **e** Relative area change of individual pores.

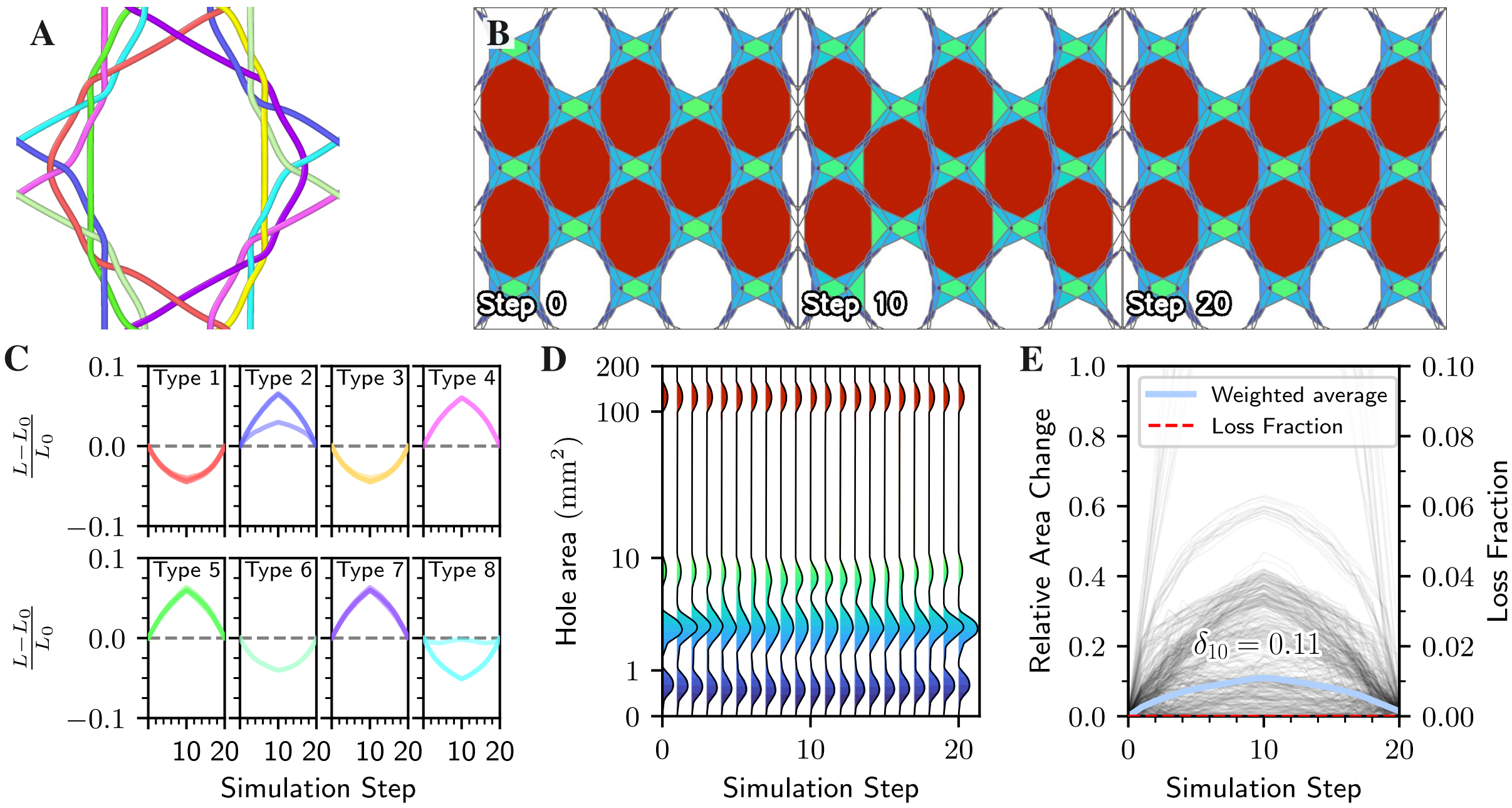

Figure S150: **Simulation of lace structure Rose-9. A** Unit-cell schematic (active bond types {1, 3, 6, 8}). **B** Snapshots at steps 0, 10, and 20, showing the initial structure, maximum deformation (active bonds shrink by 25%), and subsequent relaxation. **C** Variation of the relative length of yarns, grouped by type. **D** Evolution of the distribution of pore areas over time. **e** Relative area change of individual pores.

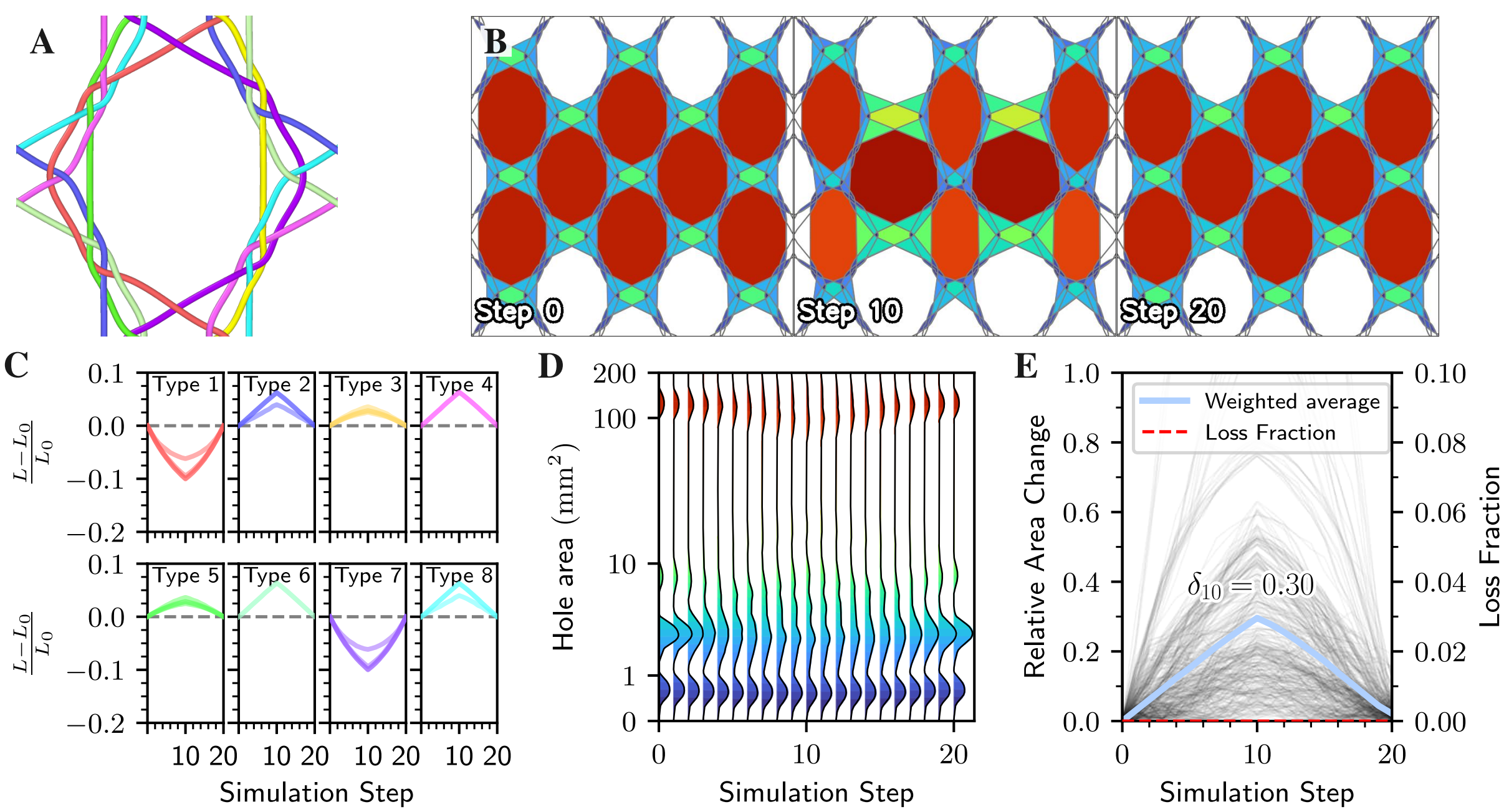

Figure S151: **Simulation of lace structure Rose-9. A** Unit-cell schematic (active bond types {1, 7}). **B** Snapshots at steps 0, 10, and 20, showing the initial structure, maximum deformation (active bonds shrink by 25%), and subsequent relaxation. **C** Variation of the relative length of yarns, grouped by type. **D** Evolution of the distribution of pore areas over time. **e** Relative area change of individual pores.

Rose-10

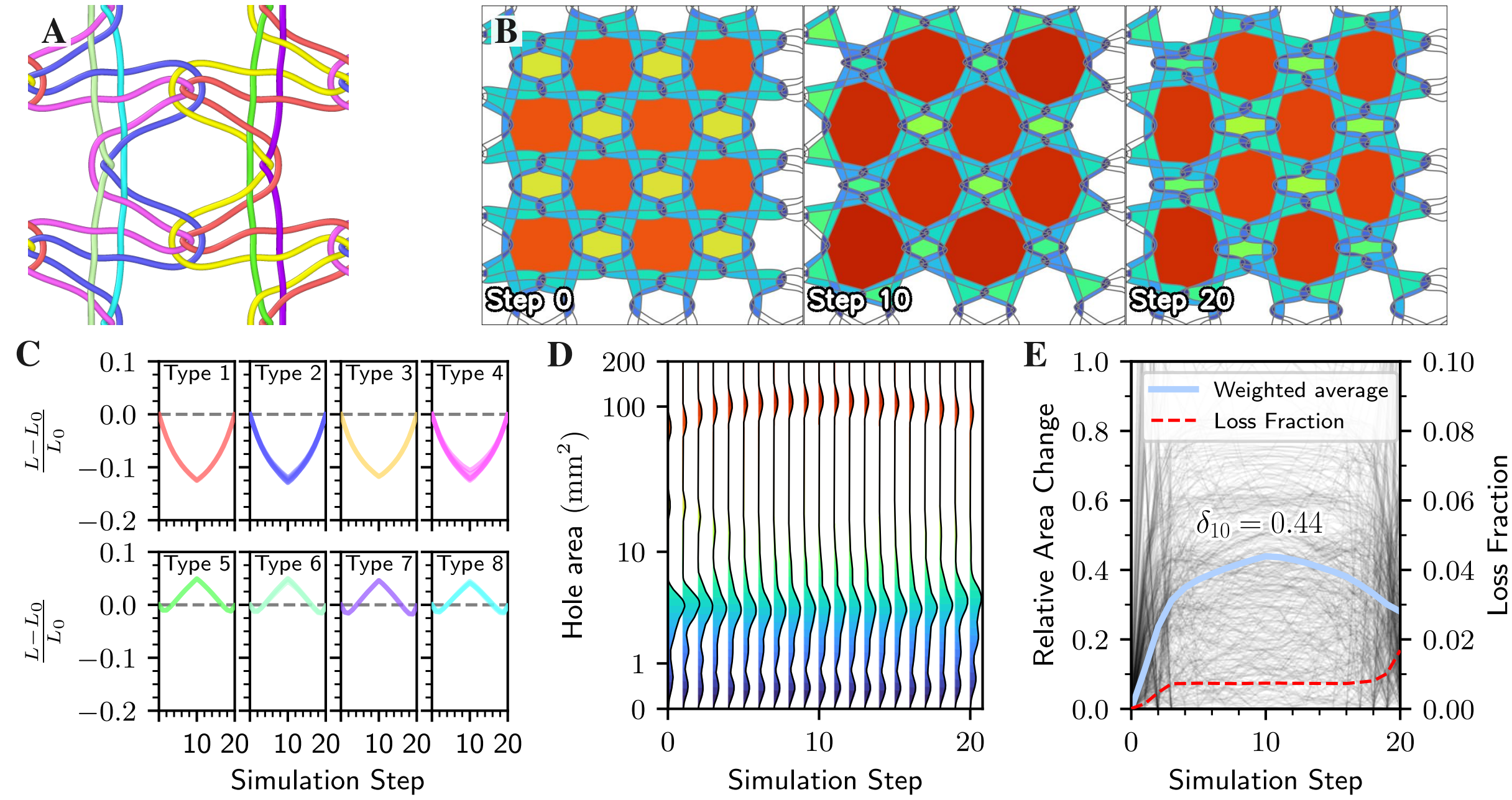

Figure S152: **Simulation of lace structure Rose-10.** **A** Unit-cell schematic (active bond types {1, 2, 3, 4, 5, 6, 7, 8}). **B** Snapshots at steps 0, 10, and 20, showing the initial structure, maximum deformation (active bonds shrink by 25%), and subsequent relaxation. **C** Variation of the relative length of yarns, grouped by type. **D** Evolution of the distribution of pore areas over time. **E** Relative area change of individual pores.

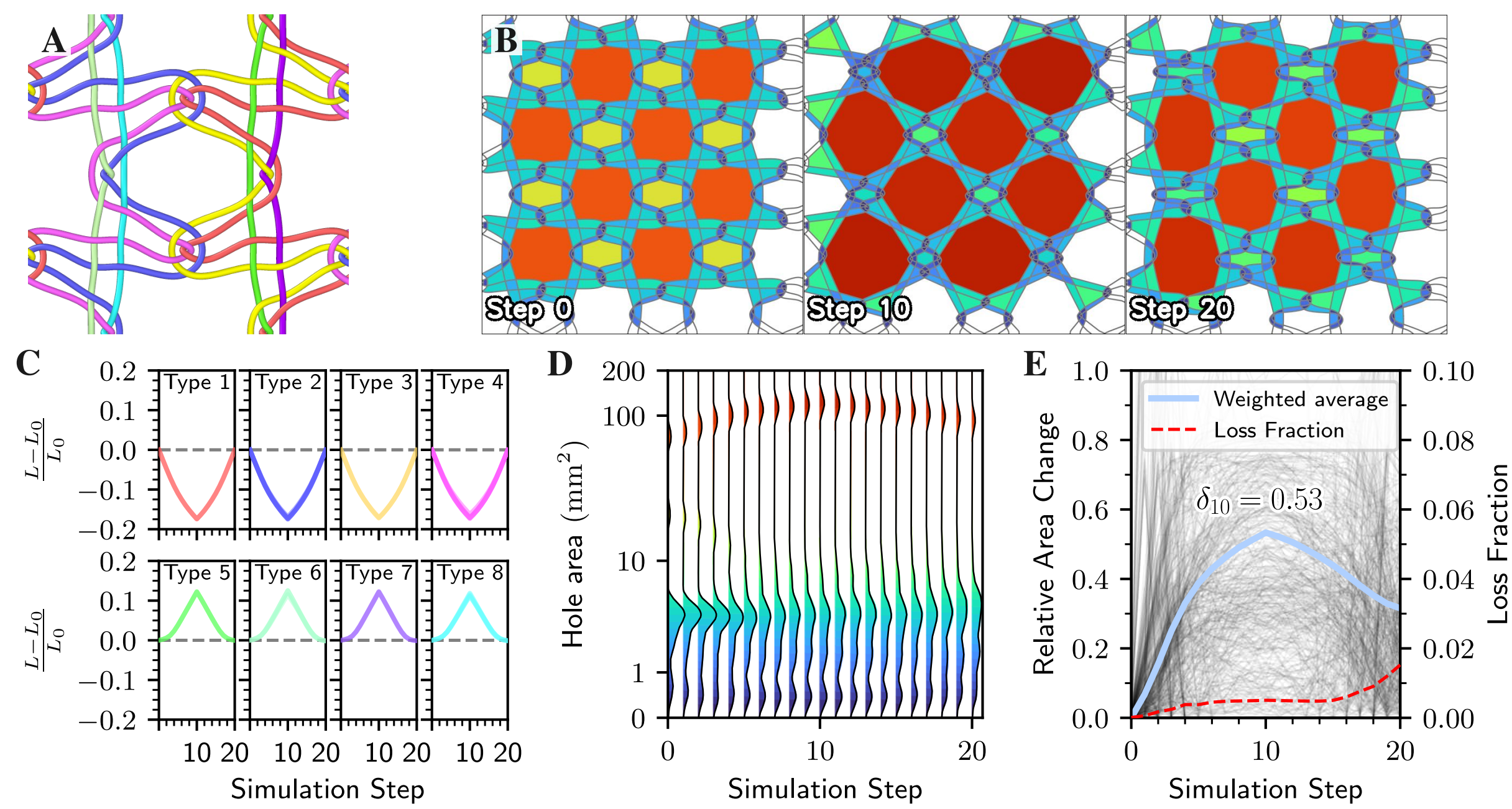

Figure S153: **Simulation of lace structure Rose-10.** **A** Unit-cell schematic (active bond types {1, 2, 3, 4}). **B** Snapshots at steps 0, 10, and 20, showing the initial structure, maximum deformation (active bonds shrink by 25%), and subsequent relaxation. **C** Variation of the relative length of yarns, grouped by type. **D** Evolution of the distribution of pore areas over time. **E** Relative area change of individual pores.

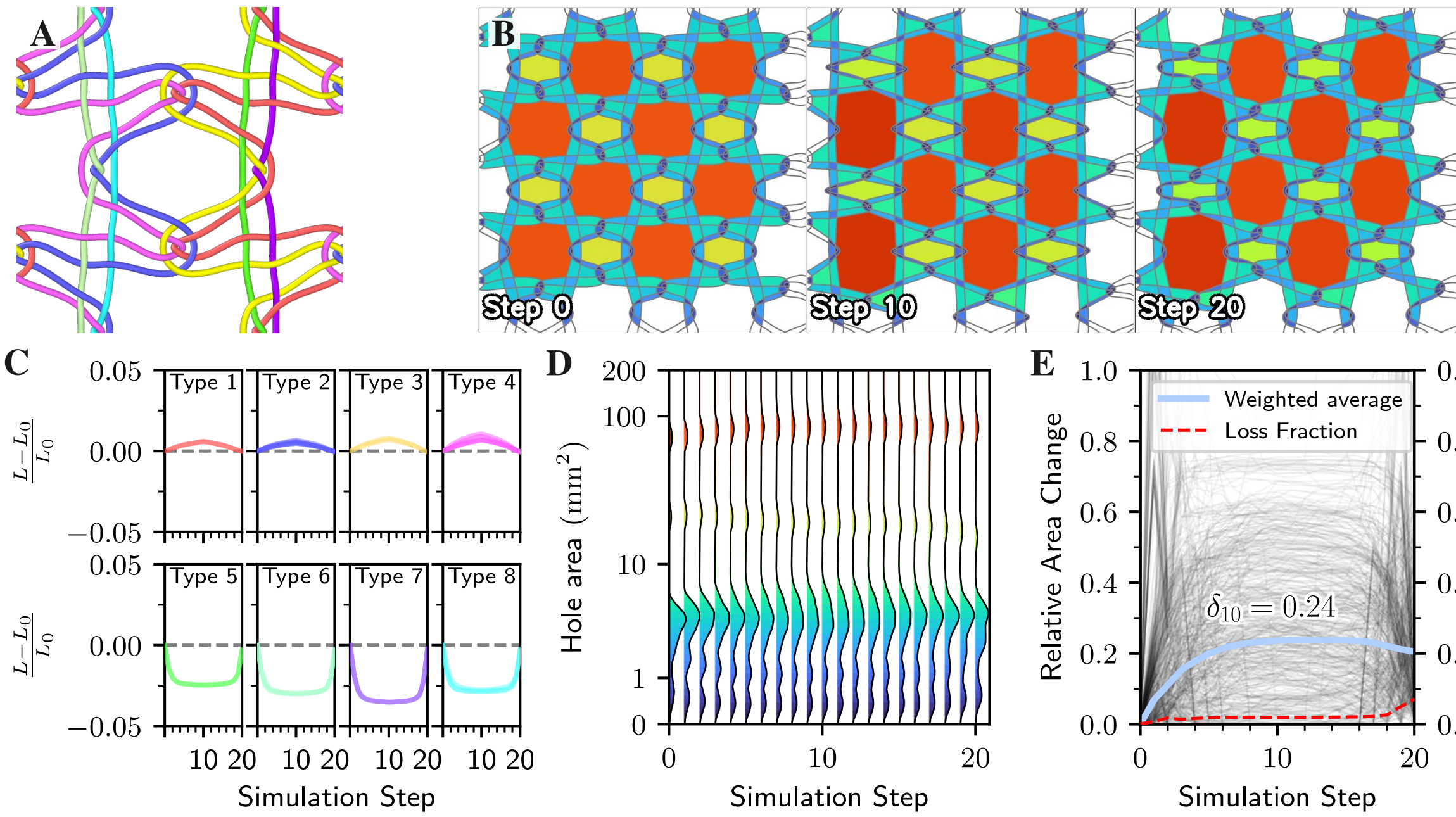

Figure S154: **Simulation of lace structure Rose-10. A** Unit-cell schematic (active bond types {5, 6, 7, 8}). **B** Snapshots at steps 0, 10, and 20, showing the initial structure, maximum deformation (active bonds shrink by 25%), and subsequent relaxation. **C** Variation of the relative length of yarns, grouped by type. **D** Evolution of the distribution of pore areas over time. **E** Relative area change of individual pores.

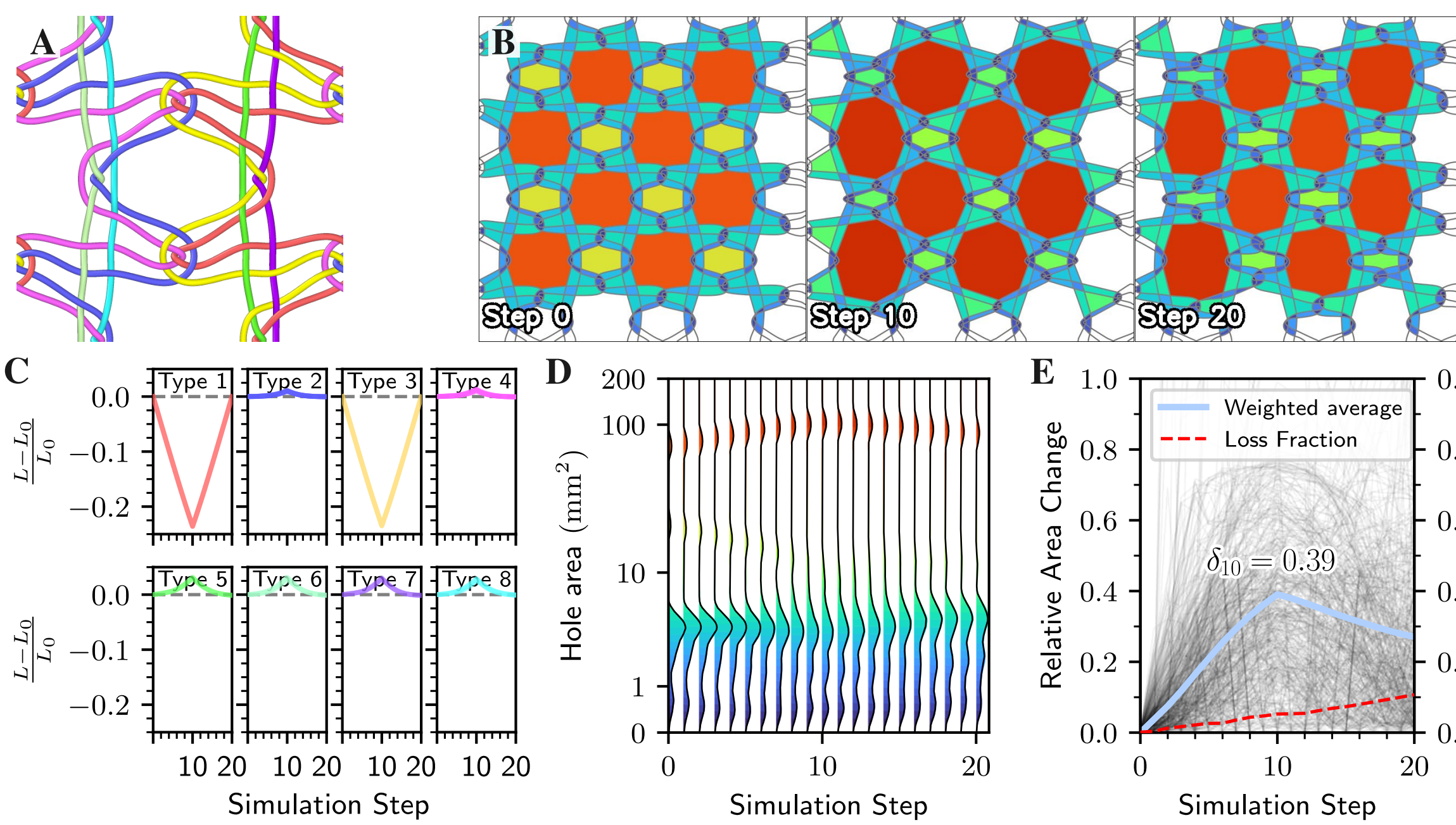

Figure S155: **Simulation of lace structure Rose-10. A** Unit-cell schematic (active bond types {1, 3}). **B** Snapshots at steps 0, 10, and 20, showing the initial structure, maximum deformation (active bonds shrink by 25%), and subsequent relaxation. **C** Variation of the relative length of yarns, grouped by type. **D** Evolution of the distribution of pore areas over time. **E** Relative area change of individual pores.

Rose-11

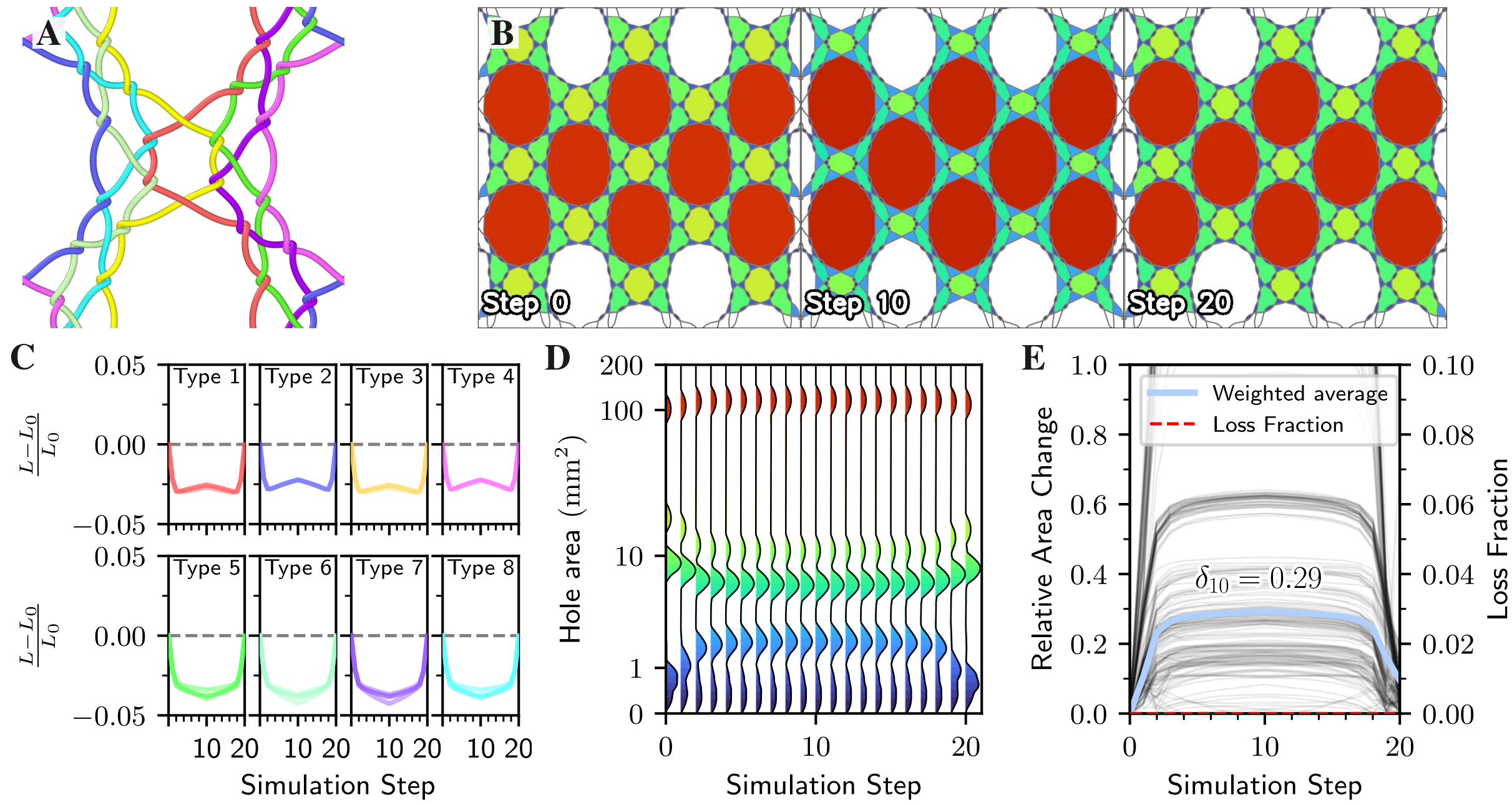

Figure S156: **Simulation of lace structure Rose-11. A** Unit-cell schematic (active bond types {1, 2, 3, 4, 5, 6, 7, 8}). **B** Snapshots at steps 0, 10, and 20, showing the initial structure, maximum deformation (active bonds shrink by 25%), and subsequent relaxation. **C** Variation of the relative length of yarns, grouped by type. **D** Evolution of the distribution of pore areas over time. **E** Relative area change of individual pores.

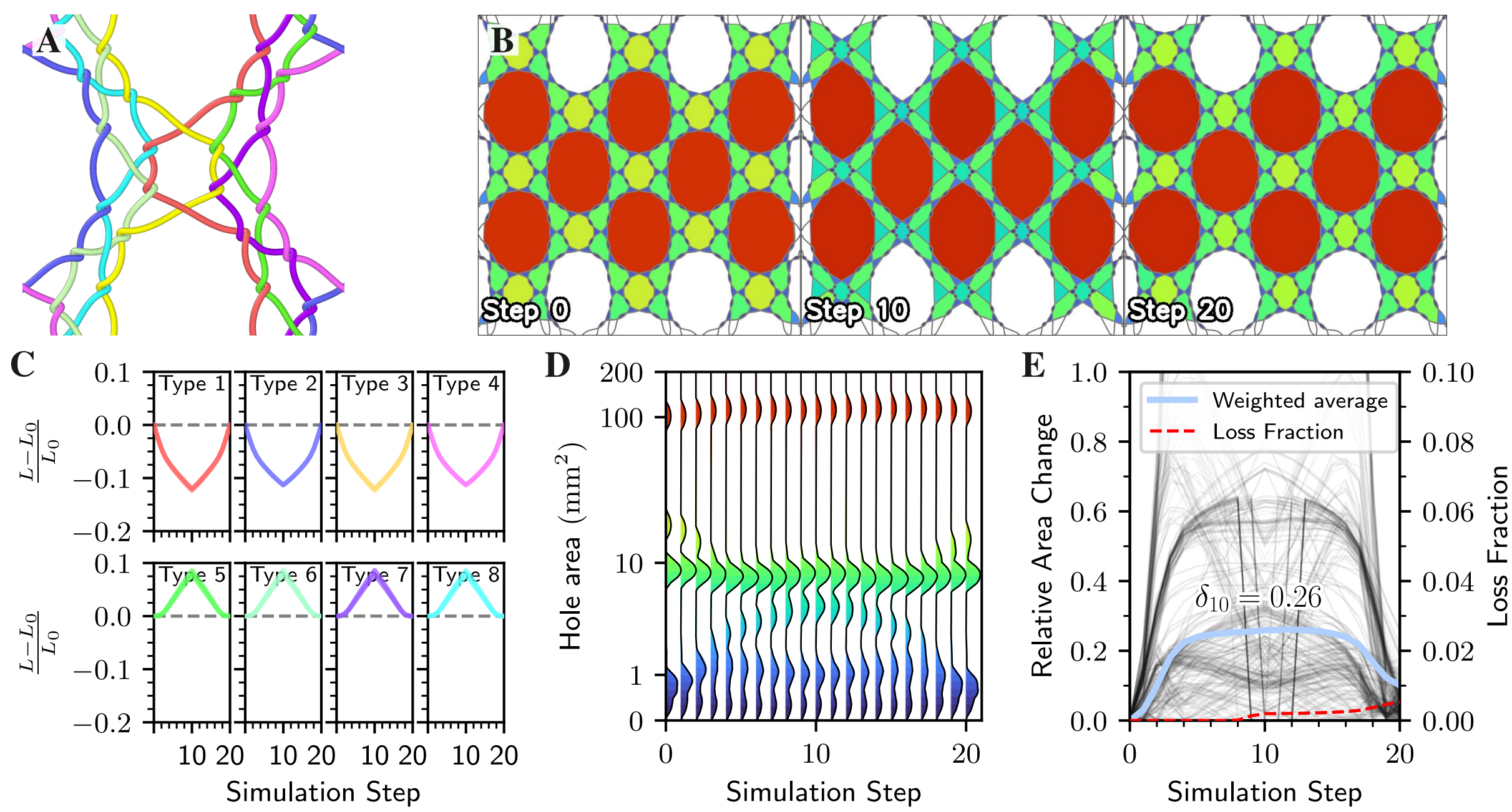

Figure S157: **Simulation of lace structure Rose-11. A** Unit-cell schematic (active bond types {1, 2, 3, 4}). **B** Snapshots at steps 0, 10, and 20, showing the initial structure, maximum deformation (active bonds shrink by 25%), and subsequent relaxation. **C** Variation of the relative length of yarns, grouped by type. **D** Evolution of the distribution of pore areas over time. **E** Relative area change of individual pores.

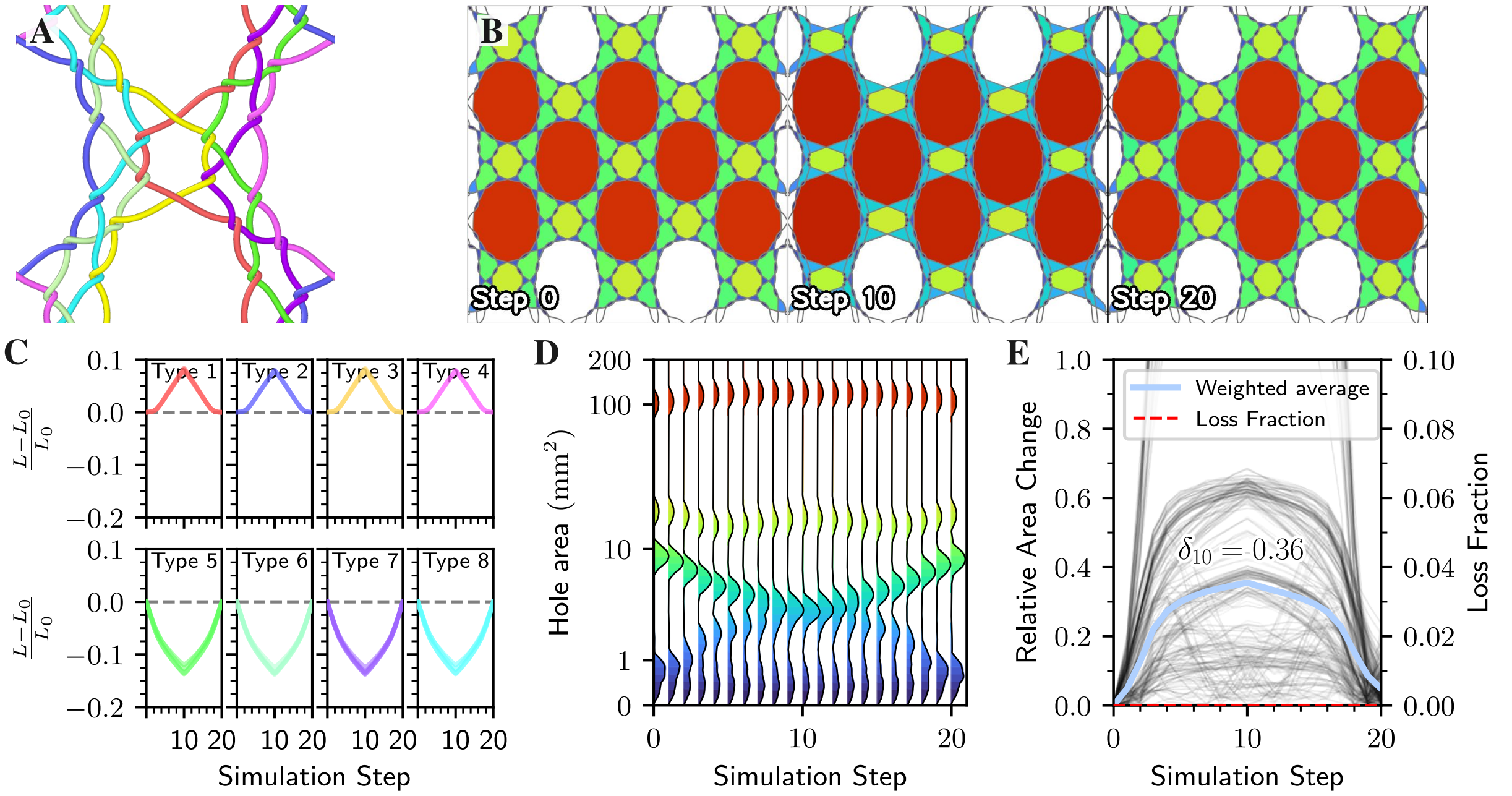

Figure S158: **Simulation of lace structure Rose-11. A** Unit-cell schematic (active bond types {5, 6, 7, 8}). **B** Snapshots at steps 0, 10, and 20, showing the initial structure, maximum deformation (active bonds shrink by 25%), and subsequent relaxation. **C** Variation of the relative length of yarns, grouped by type. **D** Evolution of the distribution of pore areas over time. **E** Relative area change of individual pores.

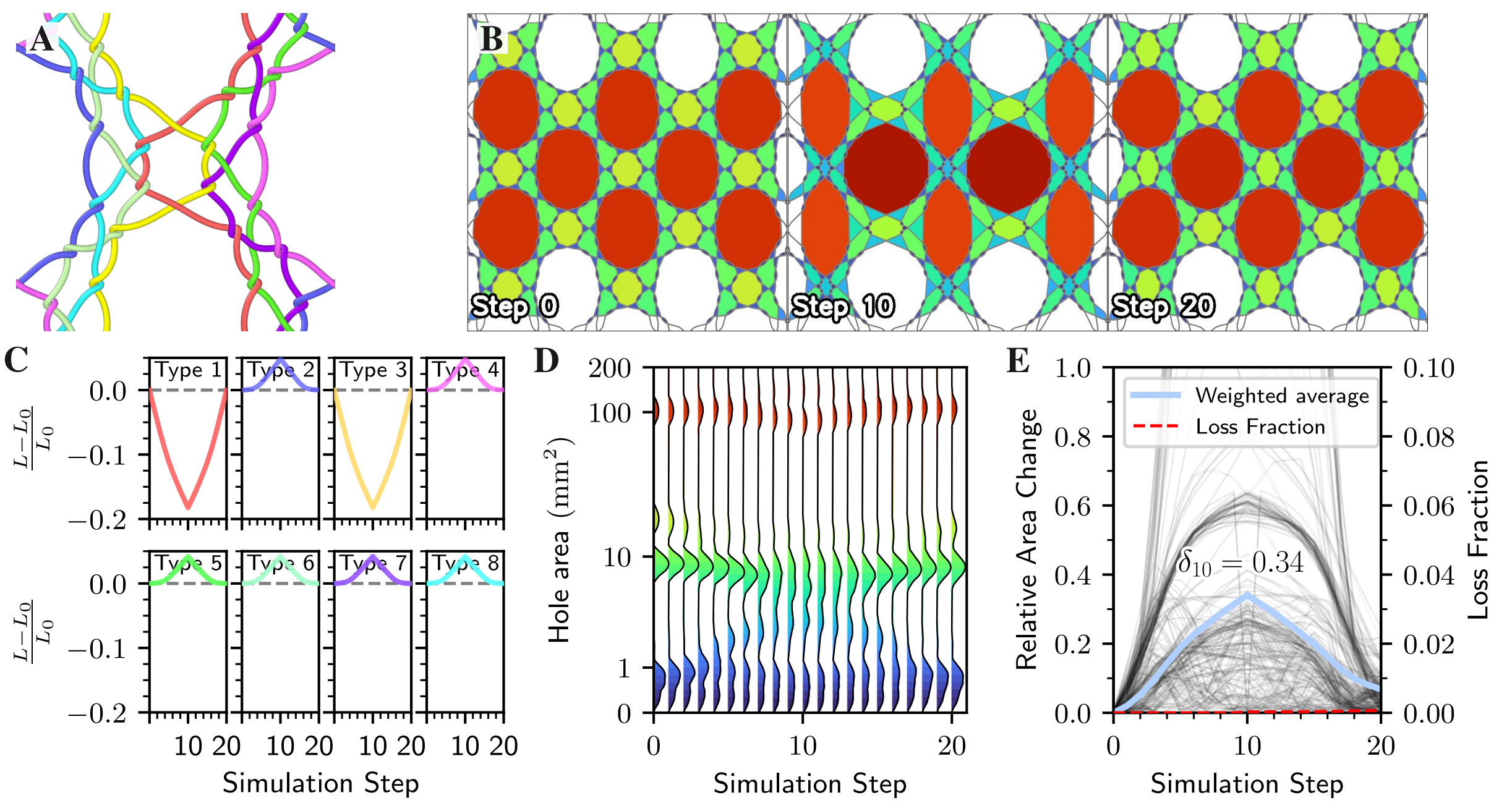

Figure S159: **Simulation of lace structure Rose-11. A** Unit-cell schematic (active bond types {1, 3}). **B** Snapshots at steps 0, 10, and 20, showing the initial structure, maximum deformation (active bonds shrink by 25%), and subsequent relaxation. **C** Variation of the relative length of yarns, grouped by type. **D** Evolution of the distribution of pore areas over time. **E** Relative area change of individual pores.

Rose-12

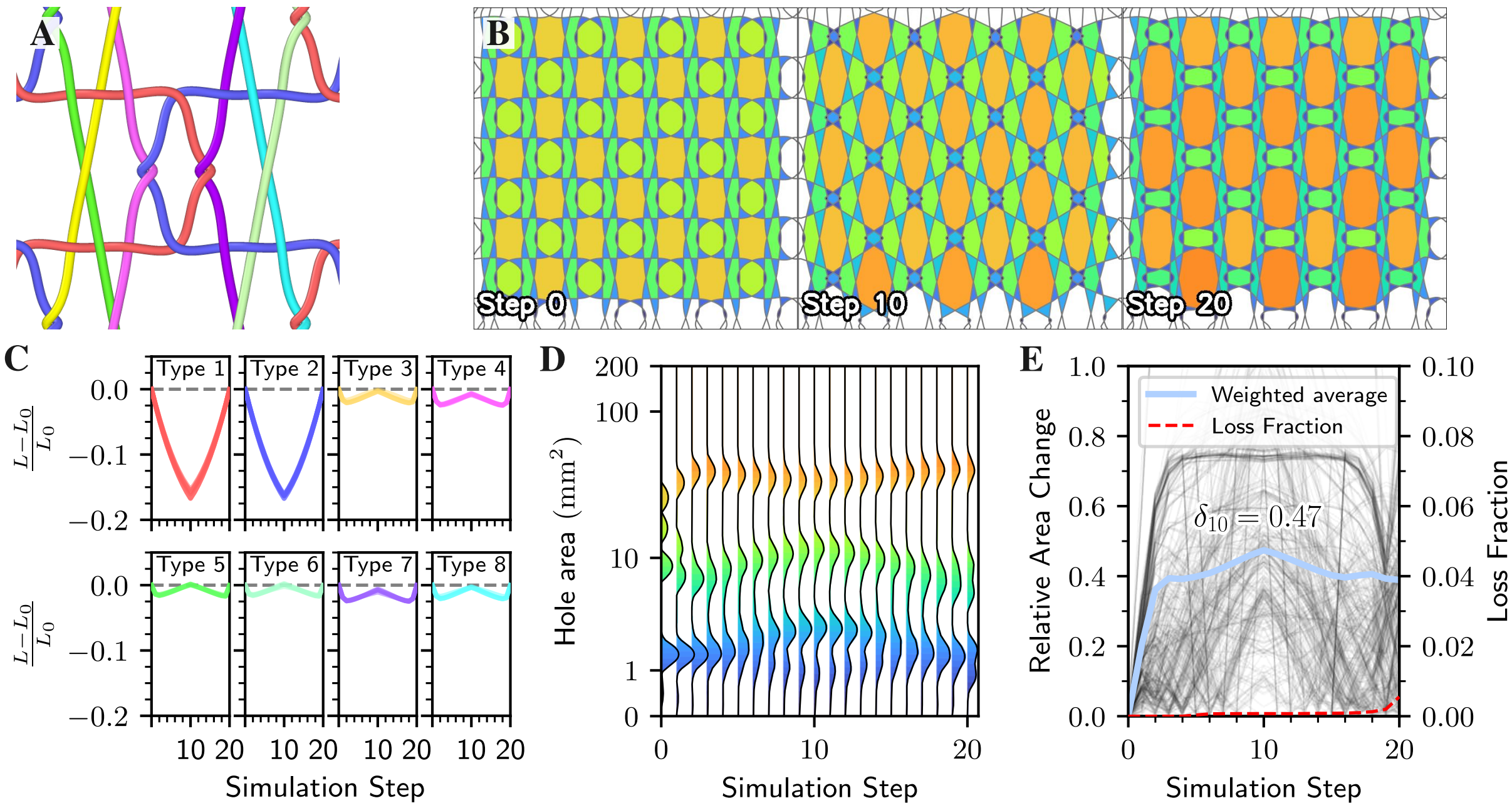

Figure S160: **Simulation of lace structure Rose-12. A** Unit-cell schematic (active bond types {1, 2, 3, 4, 5, 6, 7, 8}). **B** Snapshots at steps 0, 10, and 20, showing the initial structure, maximum deformation (active bonds shrink by 25%), and subsequent relaxation. **C** Variation of the relative length of yarns, grouped by type. **D** Evolution of the distribution of pore areas over time. **E** Relative area change of individual pores.

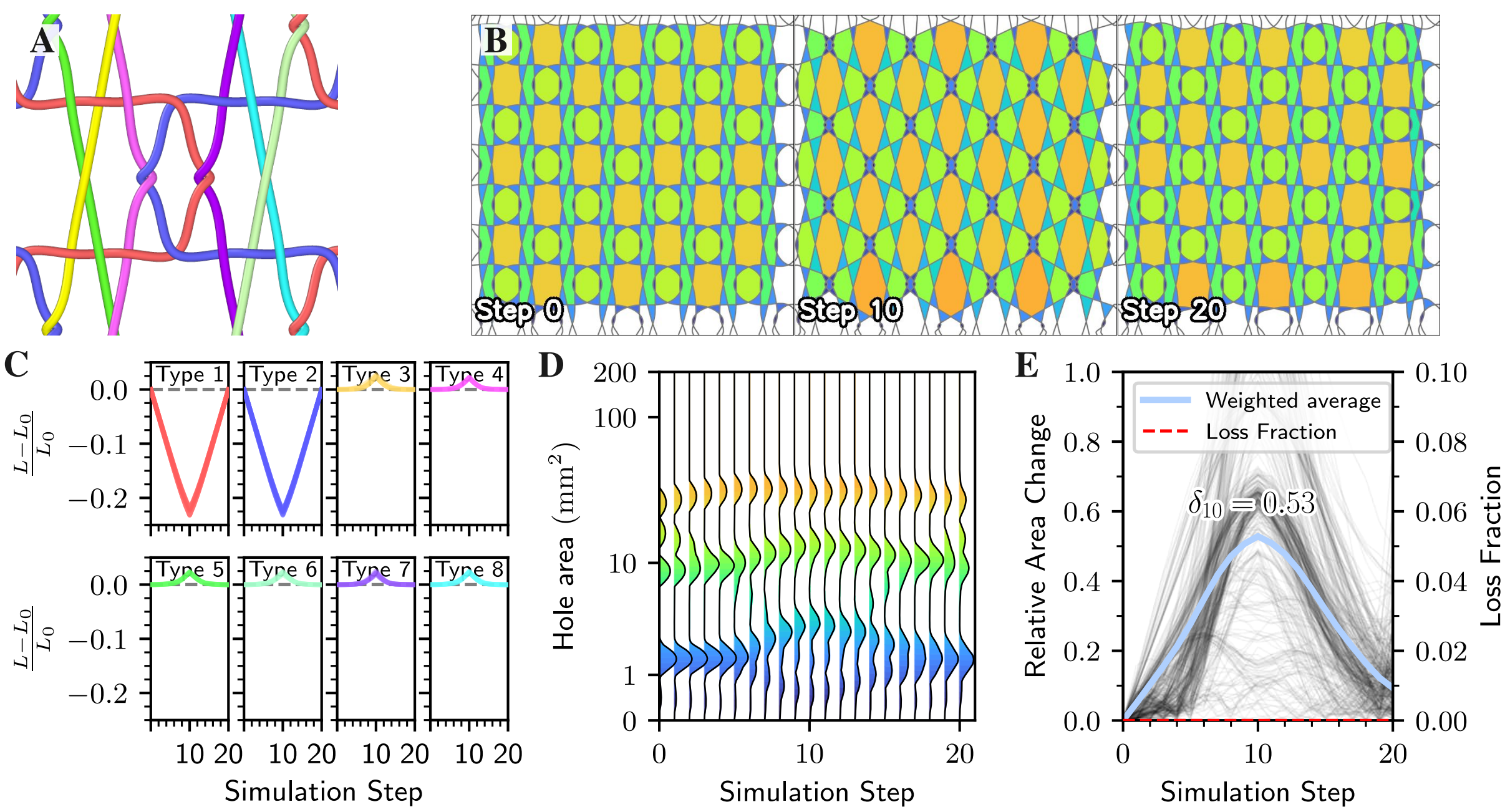

Figure S161: **Simulation of lace structure Rose-12. A** Unit-cell schematic (active bond types {1, 2}). **B** Snapshots at steps 0, 10, and 20, showing the initial structure, maximum deformation (active bonds shrink by 25%), and subsequent relaxation. **C** Variation of the relative length of yarns, grouped by type. **D** Evolution of the distribution of pore areas over time. **E** Relative area change of individual pores.

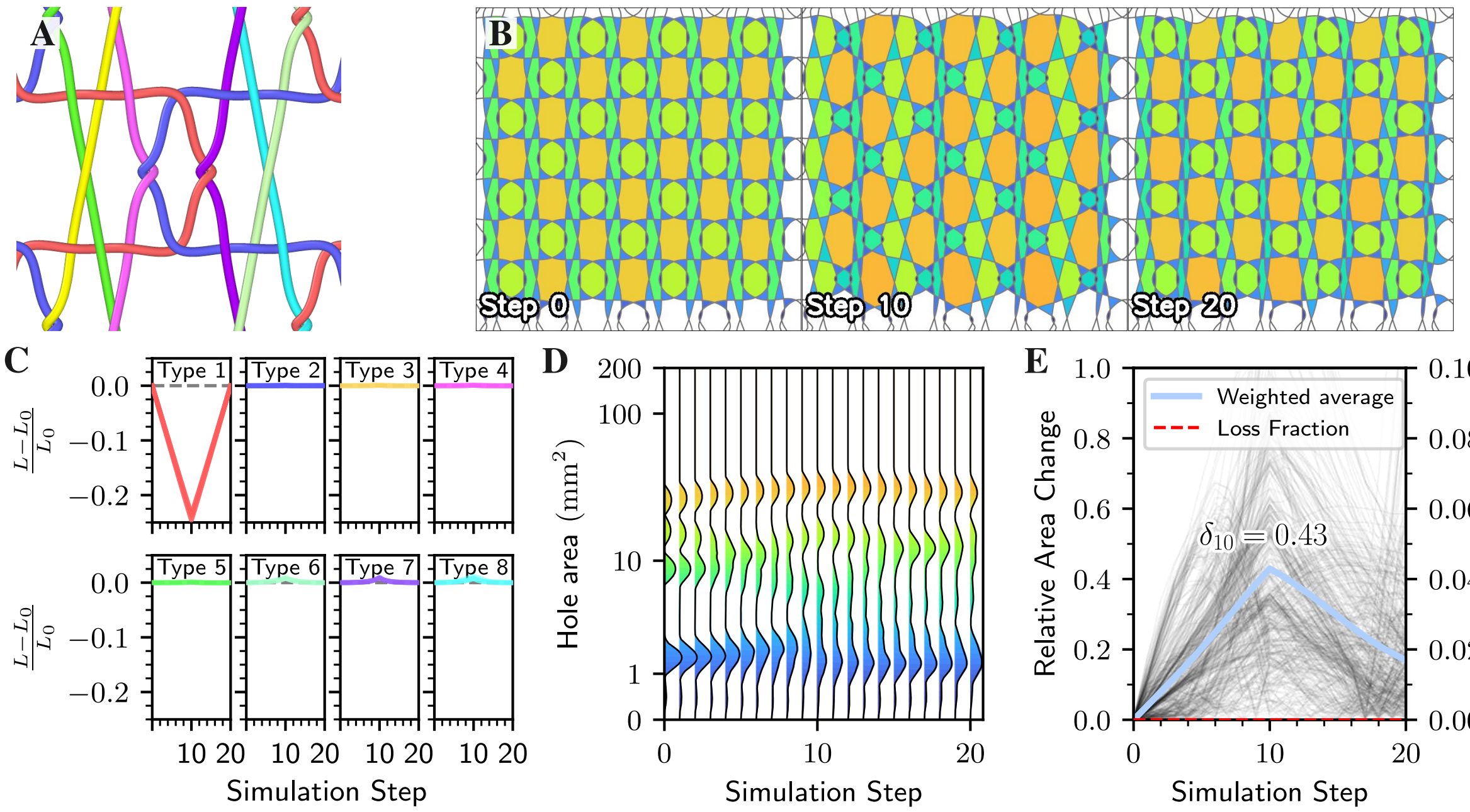

Figure S162: **Simulation of lace structure Rose-12. A** Unit-cell schematic (active bond types {1}). **B** Snapshots at steps 0, 10, and 20, showing the initial structure, maximum deformation (active bonds shrink by 25%), and subsequent relaxation. **C** Variation of the relative length of yarns, grouped by type. **D** Evolution of the distribution of pore areas over time. **E** Relative area change of individual pores.

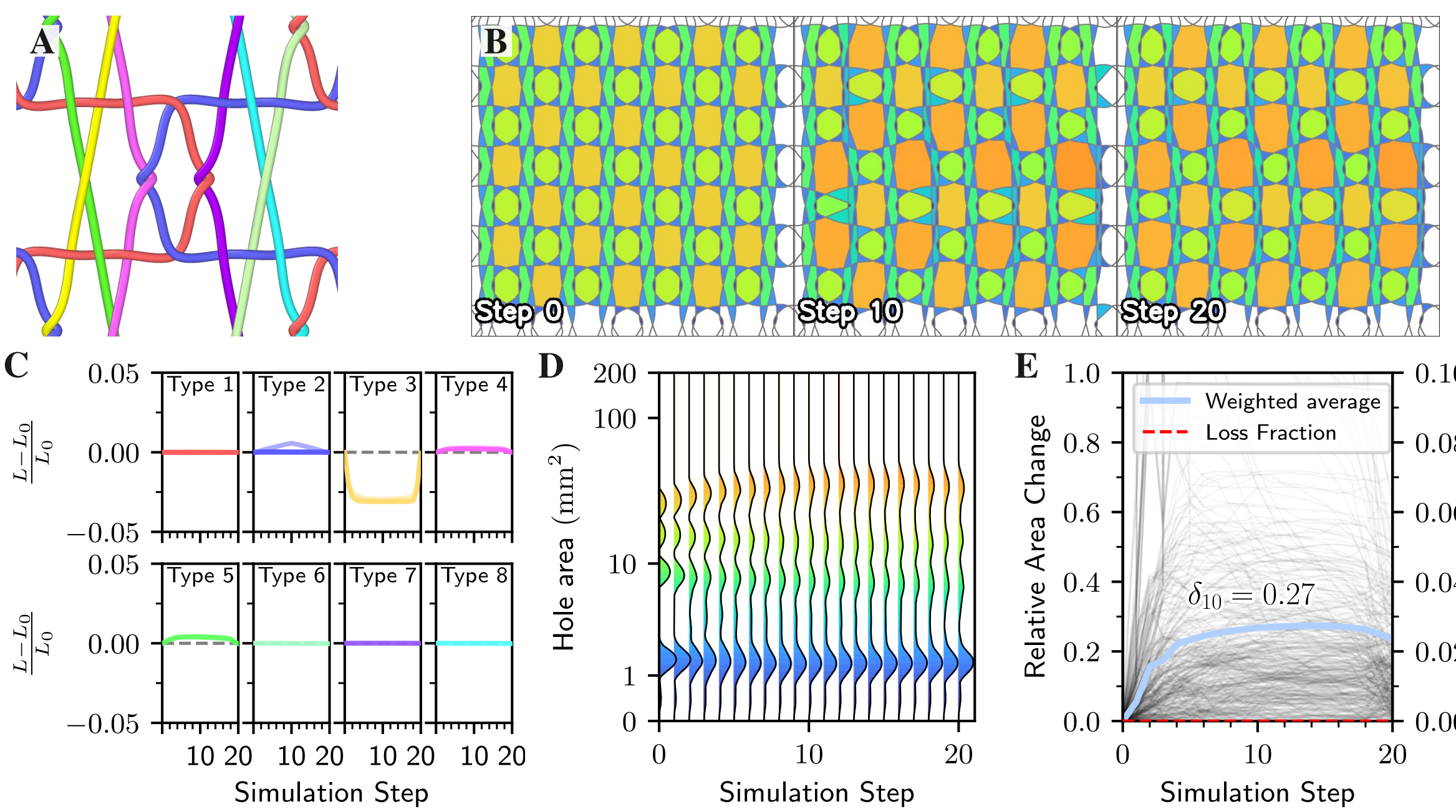

Figure S163: **Simulation of lace structure Rose-12. A** Unit-cell schematic (active bond types {3}). **B** Snapshots at steps 0, 10, and 20, showing the initial structure, maximum deformation (active bonds shrink by 25%), and subsequent relaxation. **C** Variation of the relative length of yarns, grouped by type. **D** Evolution of the distribution of pore areas over time. **E** Relative area change of individual pores.

Rose-13

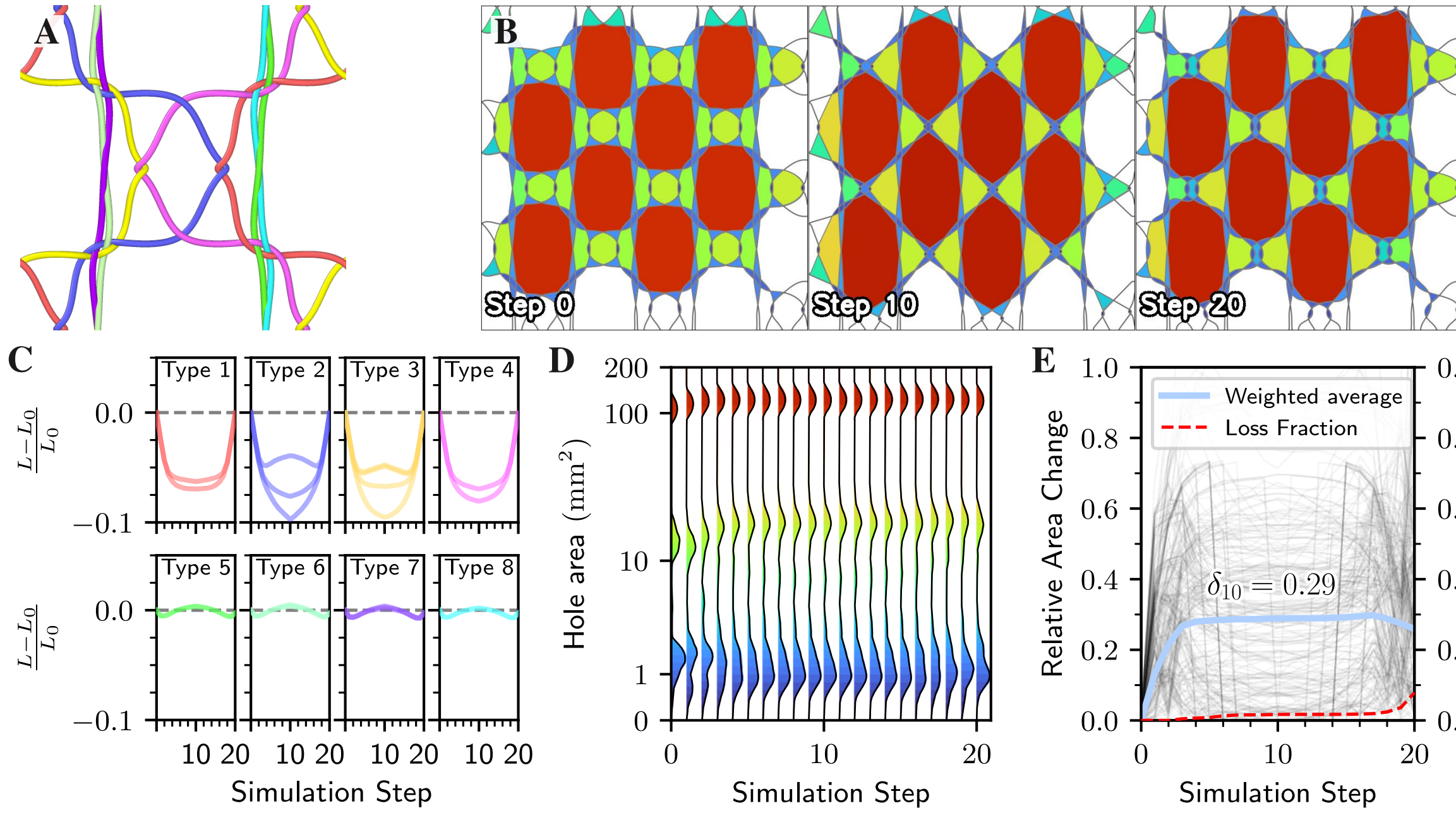

Figure S164: **Simulation of lace structure Rose-13. A** Unit-cell schematic (active bond types {1, 2, 3, 4, 5, 6, 7, 8}). **B** Snapshots at steps 0, 10, and 20, showing the initial structure, maximum deformation (active bonds shrink by 25%), and subsequent relaxation. **C** Variation of the relative length of yarns, grouped by type. **D** Evolution of the distribution of pore areas over time. **E** Relative area change of individual pores.

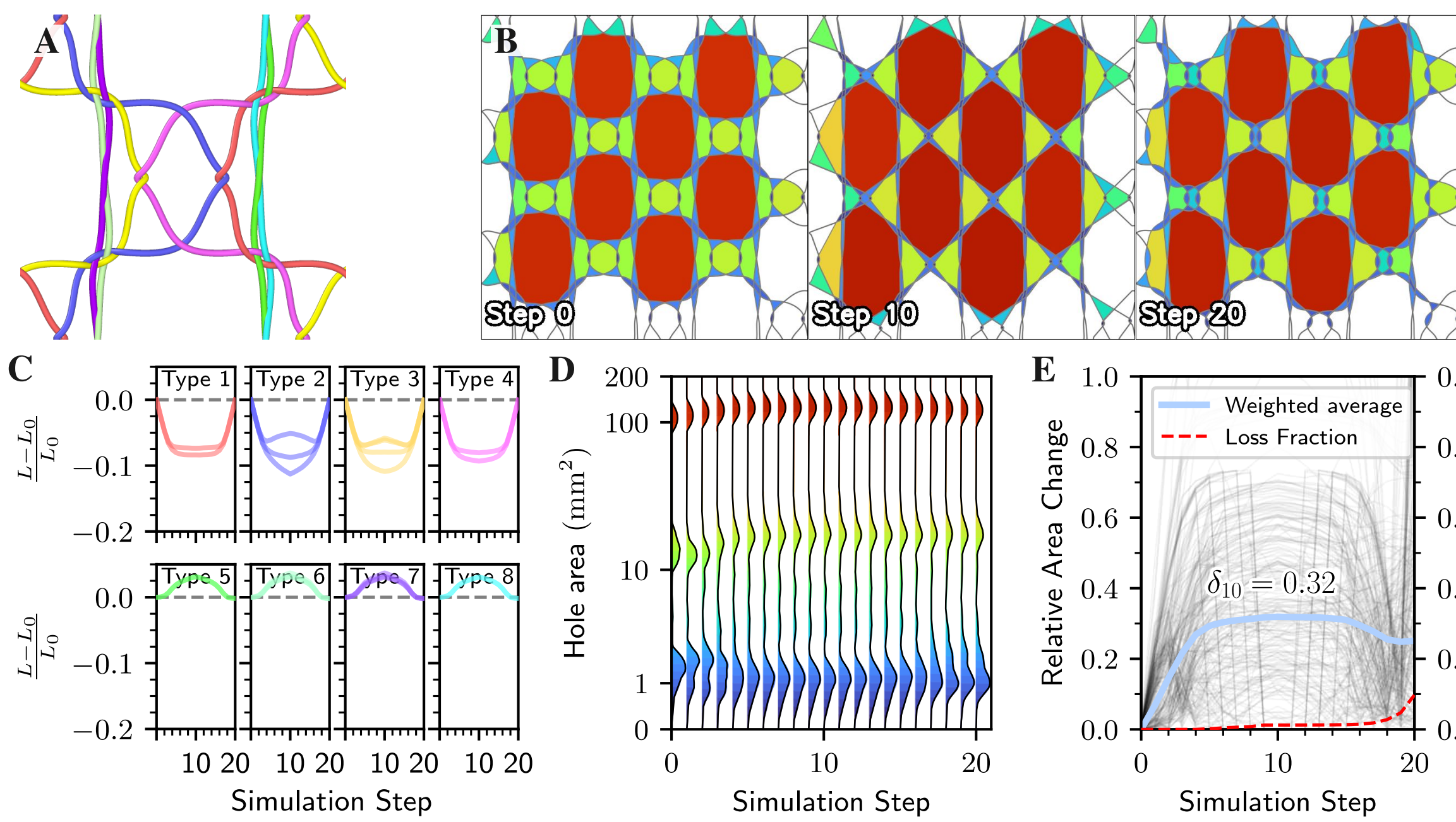

Figure S165: **Simulation of lace structure Rose-13. A** Unit-cell schematic (active bond types {1, 2, 3, 4}). **B** Snapshots at steps 0, 10, and 20, showing the initial structure, maximum deformation (active bonds shrink by 25%), and subsequent relaxation. **C** Variation of the relative length of yarns, grouped by type. **D** Evolution of the distribution of pore areas over time. **E** Relative area change of individual pores.

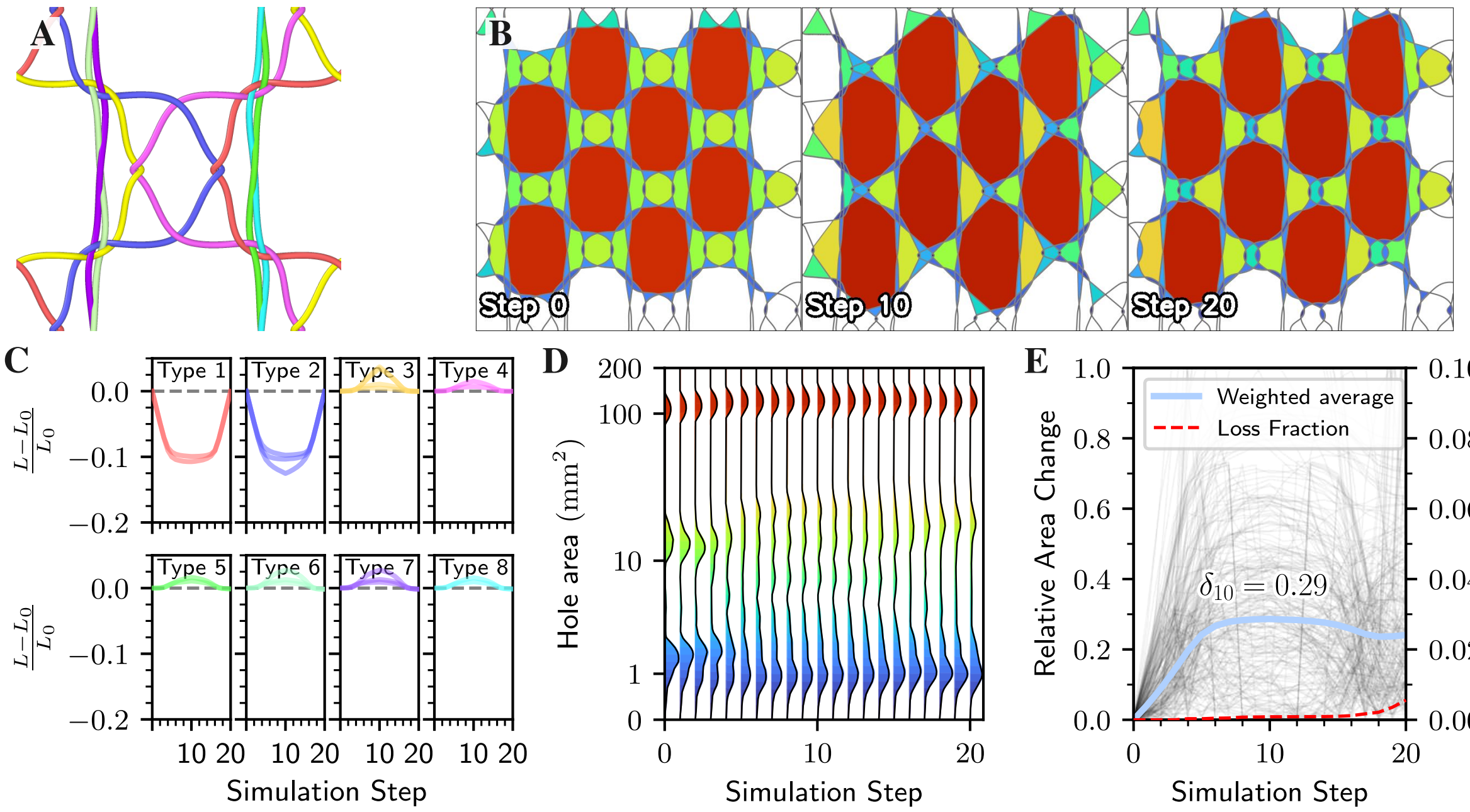

Figure S166: **Simulation of lace structure Rose-13. A** Unit-cell schematic (active bond types {1, 2}). **B** Snapshots at steps 0, 10, and 20, showing the initial structure, maximum deformation (active bonds shrink by 25%), and subsequent relaxation. **C** Variation of the relative length of yarns, grouped by type. **D** Evolution of the distribution of pore areas over time. **E** Relative area change of individual pores.

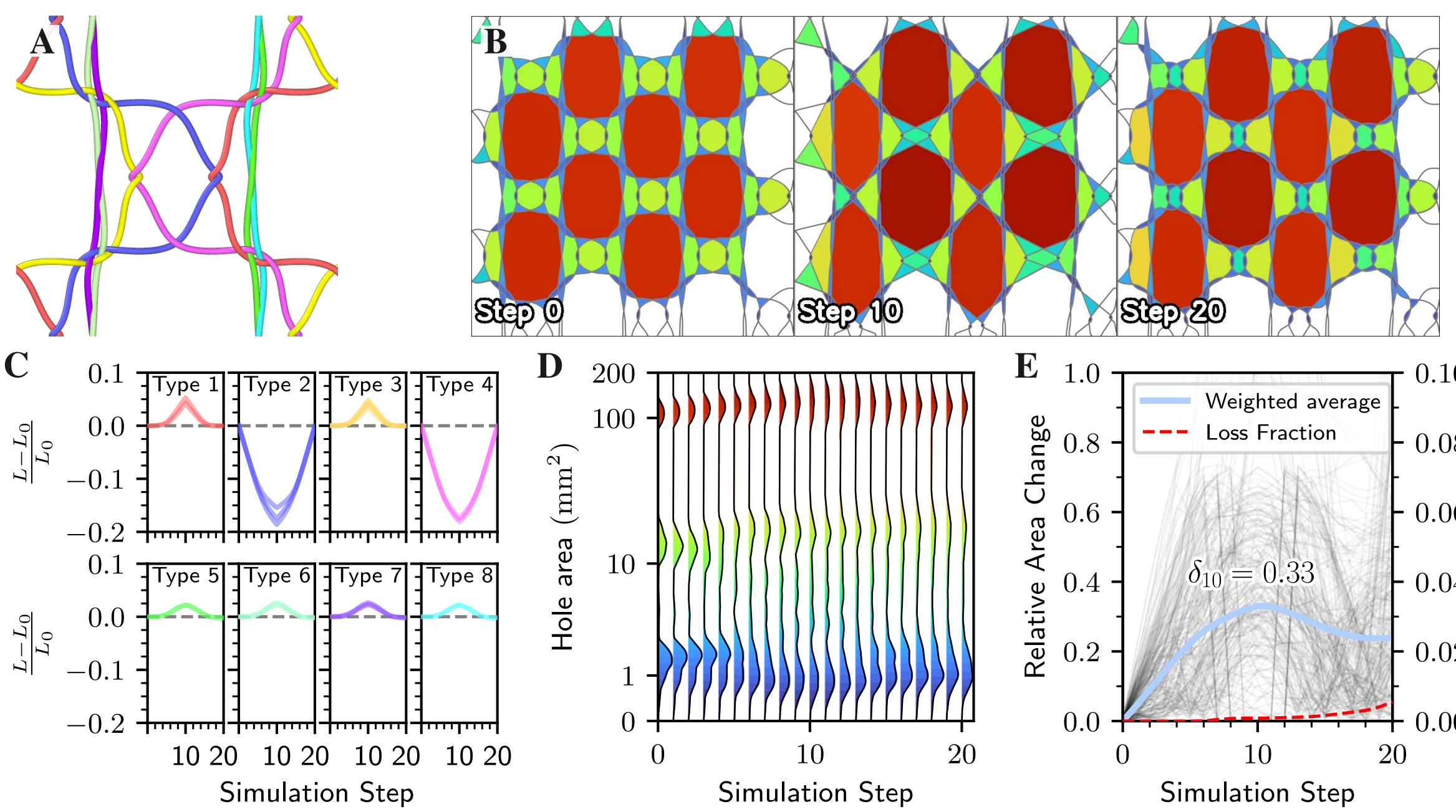

Figure S167: **Simulation of lace structure Rose-13. A** Unit-cell schematic (active bond types {2, 4}). **B** Snapshots at steps 0, 10, and 20, showing the initial structure, maximum deformation (active bonds shrink by 25%), and subsequent relaxation. **C** Variation of the relative length of yarns, grouped by type. **D** Evolution of the distribution of pore areas over time. **E** Relative area change of individual pores.

Spruce-1

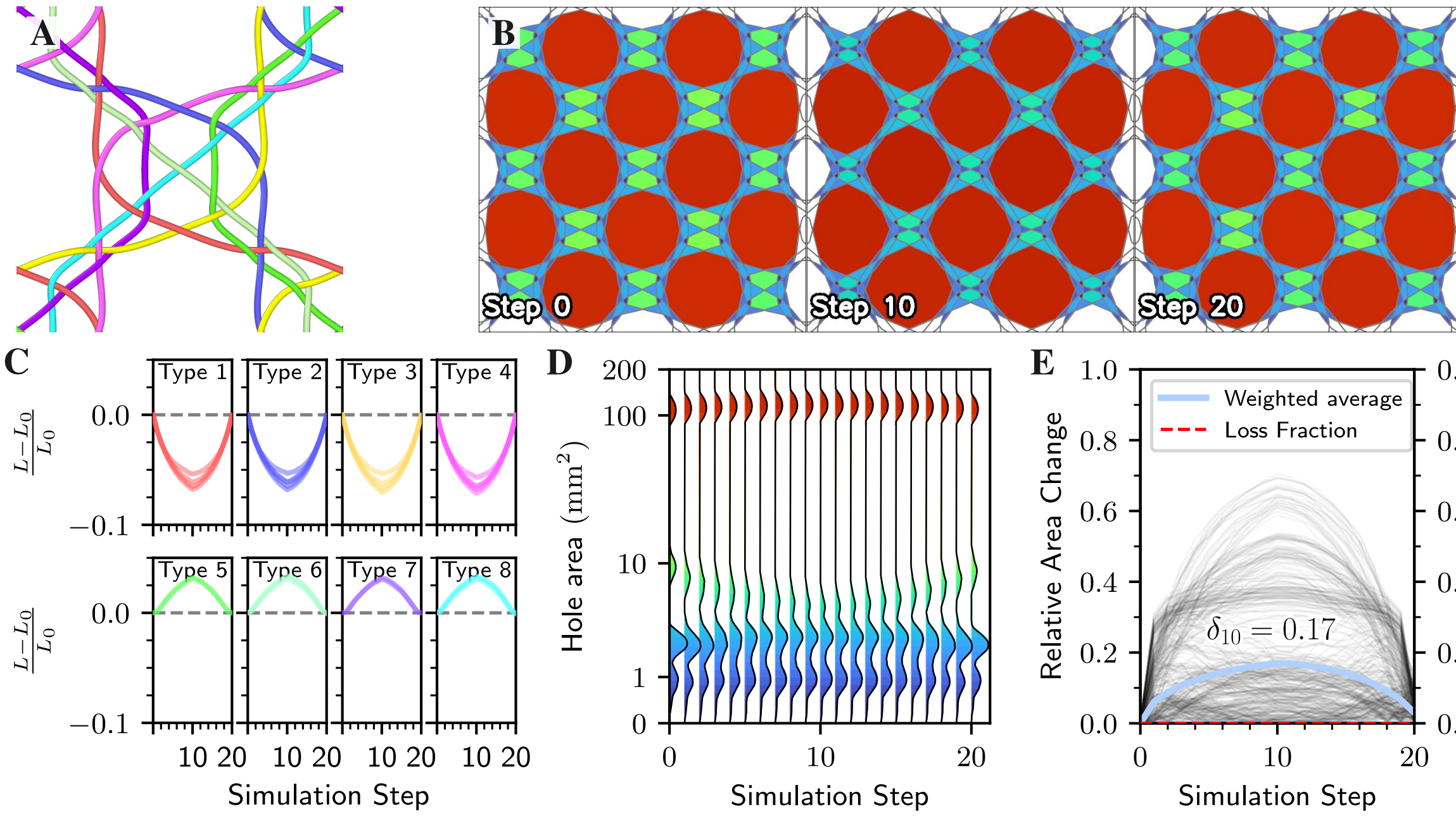

Figure S168: **Simulation of lace structure Spruce-1. A** Unit-cell schematic (active bond types {1, 2, 3, 4, 5, 6, 7, 8}). **B** Snapshots at steps 0, 10, and 20, showing the initial structure, maximum deformation (active bonds shrink by 25%), and subsequent relaxation. **C** Variation of the relative length of yarns, grouped by type. **D** Evolution of the distribution of pore areas over time. **E** Relative area change of individual pores.

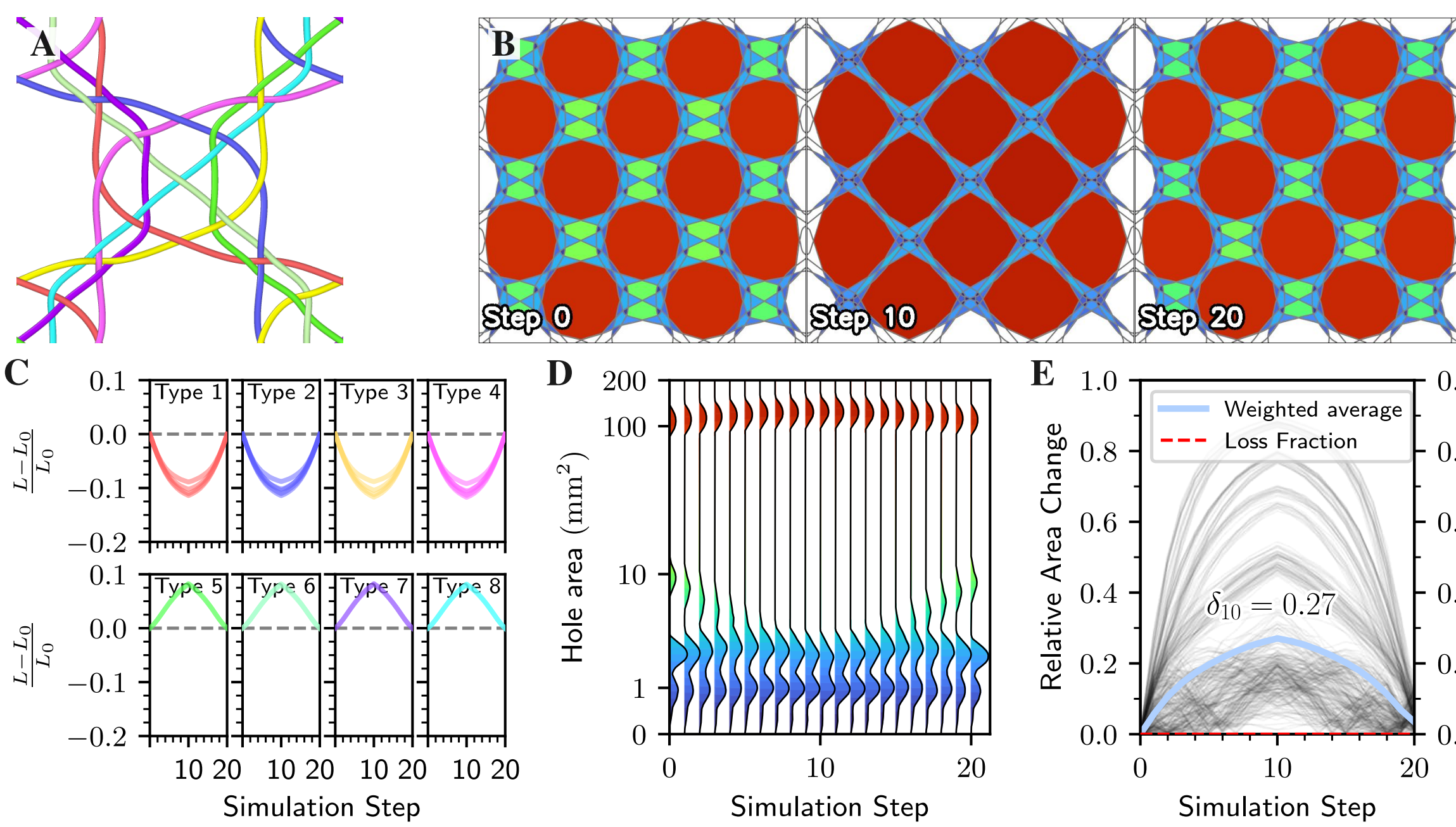

Figure S169: **Simulation of lace structure Spruce-1. A** Unit-cell schematic (active bond types {1, 2, 3, 4}). **B** Snapshots at steps 0, 10, and 20, showing the initial structure, maximum deformation (active bonds shrink by 25%), and subsequent relaxation. **C** Variation of the relative length of yarns, grouped by type. **D** Evolution of the distribution of pore areas over time. **E** Relative area change of individual pores.

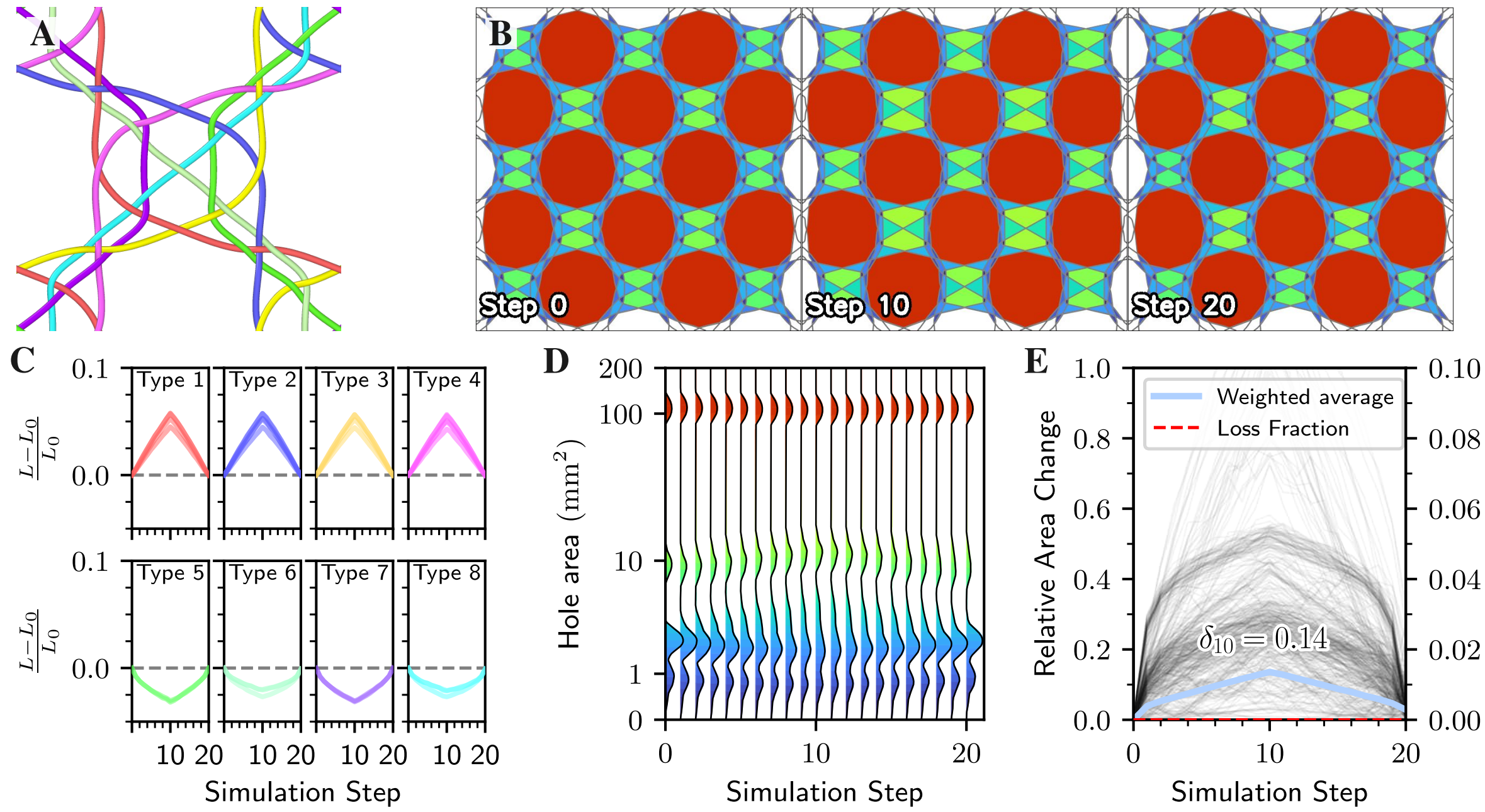

Figure S170: **Simulation of lace structure Spruce-1. A** Unit-cell schematic (active bond types {5, 6, 7, 8}). **B** Snapshots at steps 0, 10, and 20, showing the initial structure, maximum deformation (active bonds shrink by 25%), and subsequent relaxation. **C** Variation of the relative length of yarns, grouped by type. **D** Evolution of the distribution of pore areas over time. **E** Relative area change of individual pores.

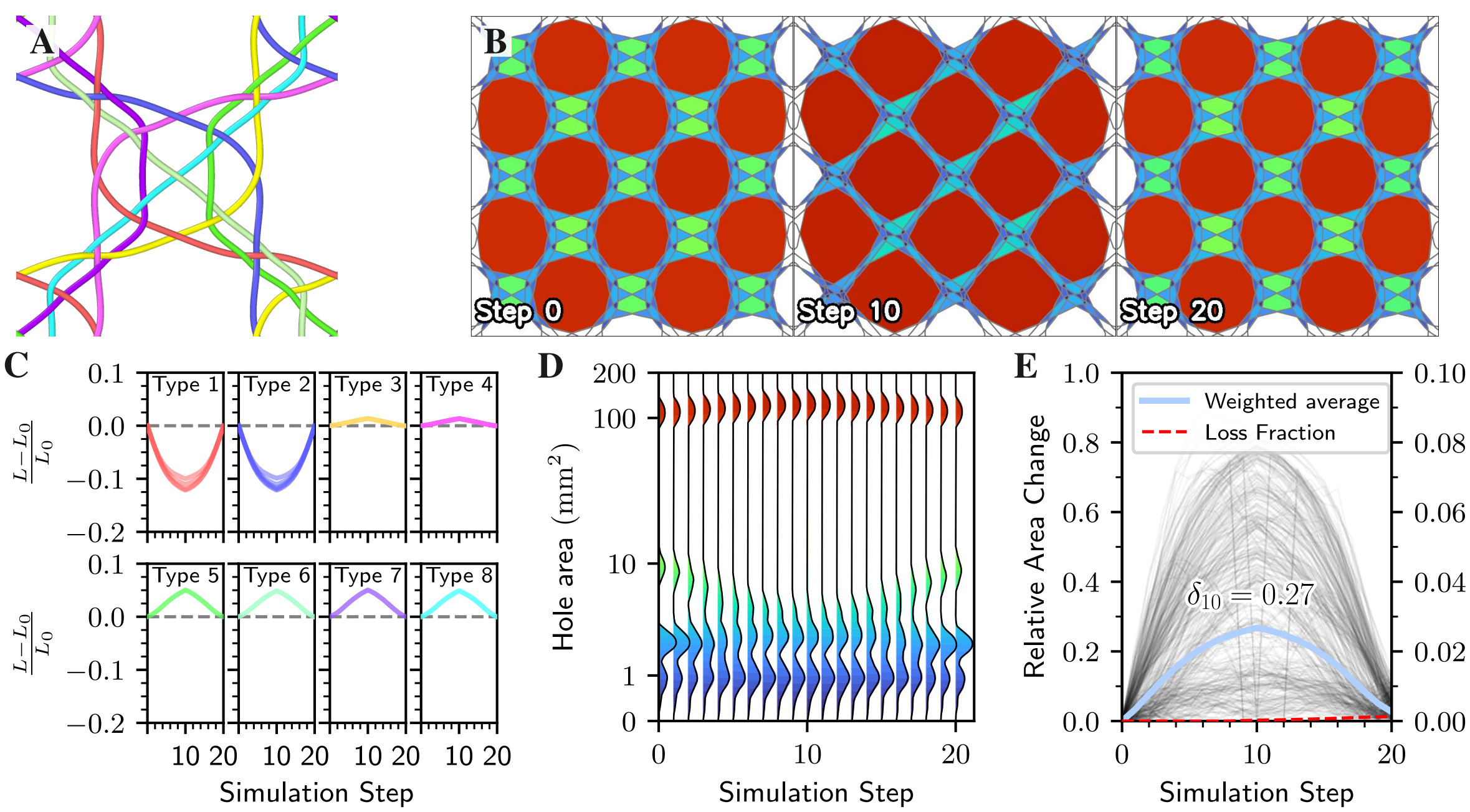

Figure S171: **Simulation of lace structure Spruce-1. A** Unit-cell schematic (active bond types {1, 2}). **B** Snapshots at steps 0, 10, and 20, showing the initial structure, maximum deformation (active bonds shrink by 25%), and subsequent relaxation. **C** Variation of the relative length of yarns, grouped by type. **D** Evolution of the distribution of pore areas over time. **E** Relative area change of individual pores.

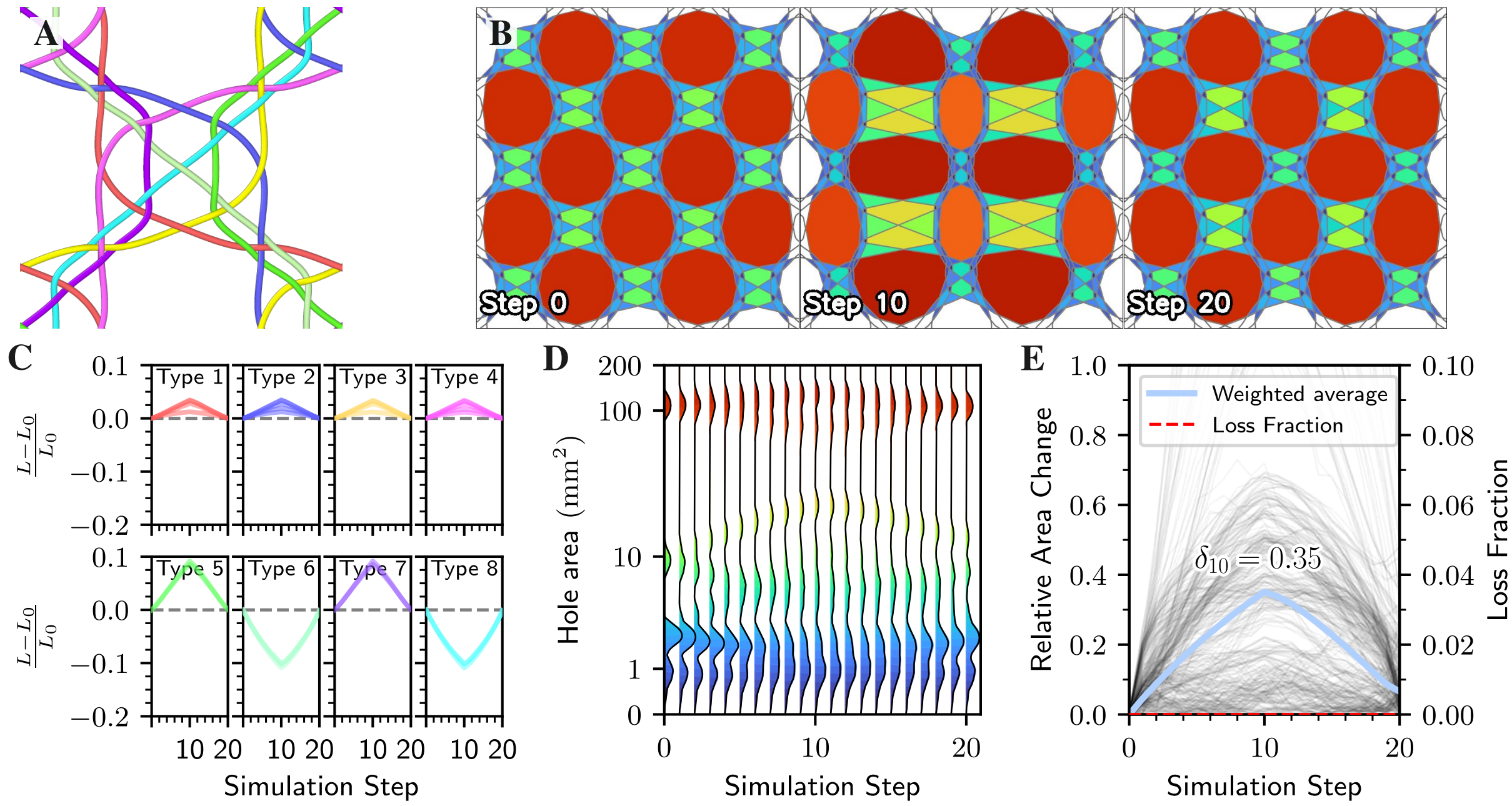

Figure S172: **Simulation of lace structure Spruce-1. A** Unit-cell schematic (active bond types {6, 8}). **B** Snapshots at steps 0, 10, and 20, showing the initial structure, maximum deformation (active bonds shrink by 25%), and subsequent relaxation. **C** Variation of the relative length of yarns, grouped by type. **D** Evolution of the distribution of pore areas over time. **E** Relative area change of individual pores.

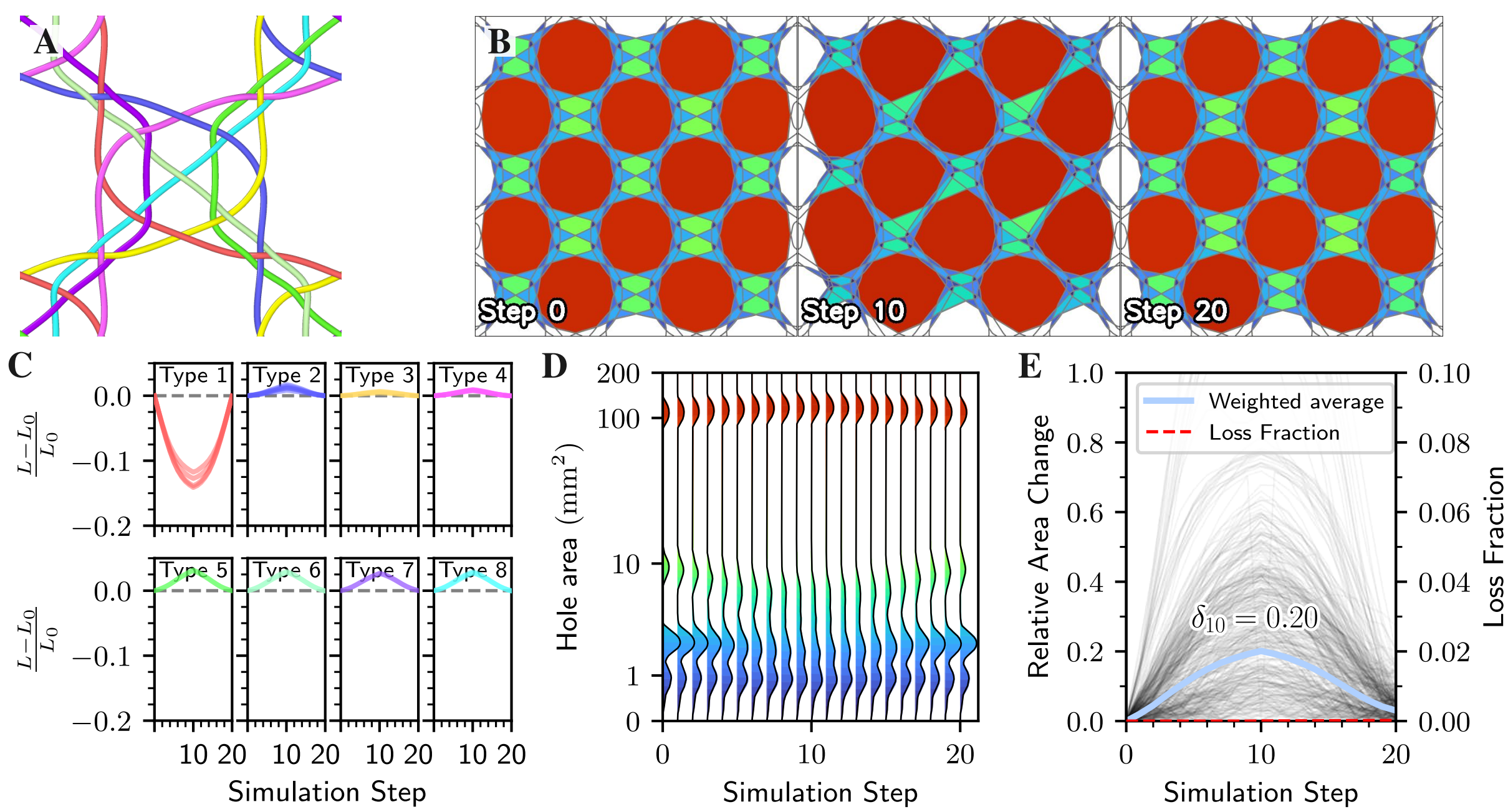

Figure S173: **Simulation of lace structure Spruce-1. A** Unit-cell schematic (active bond types {1}). **B** Snapshots at steps 0, 10, and 20, showing the initial structure, maximum deformation (active bonds shrink by 25%), and subsequent relaxation. **C** Variation of the relative length of yarns, grouped by type. **D** Evolution of the distribution of pore areas over time. **E** Relative area change of individual pores.

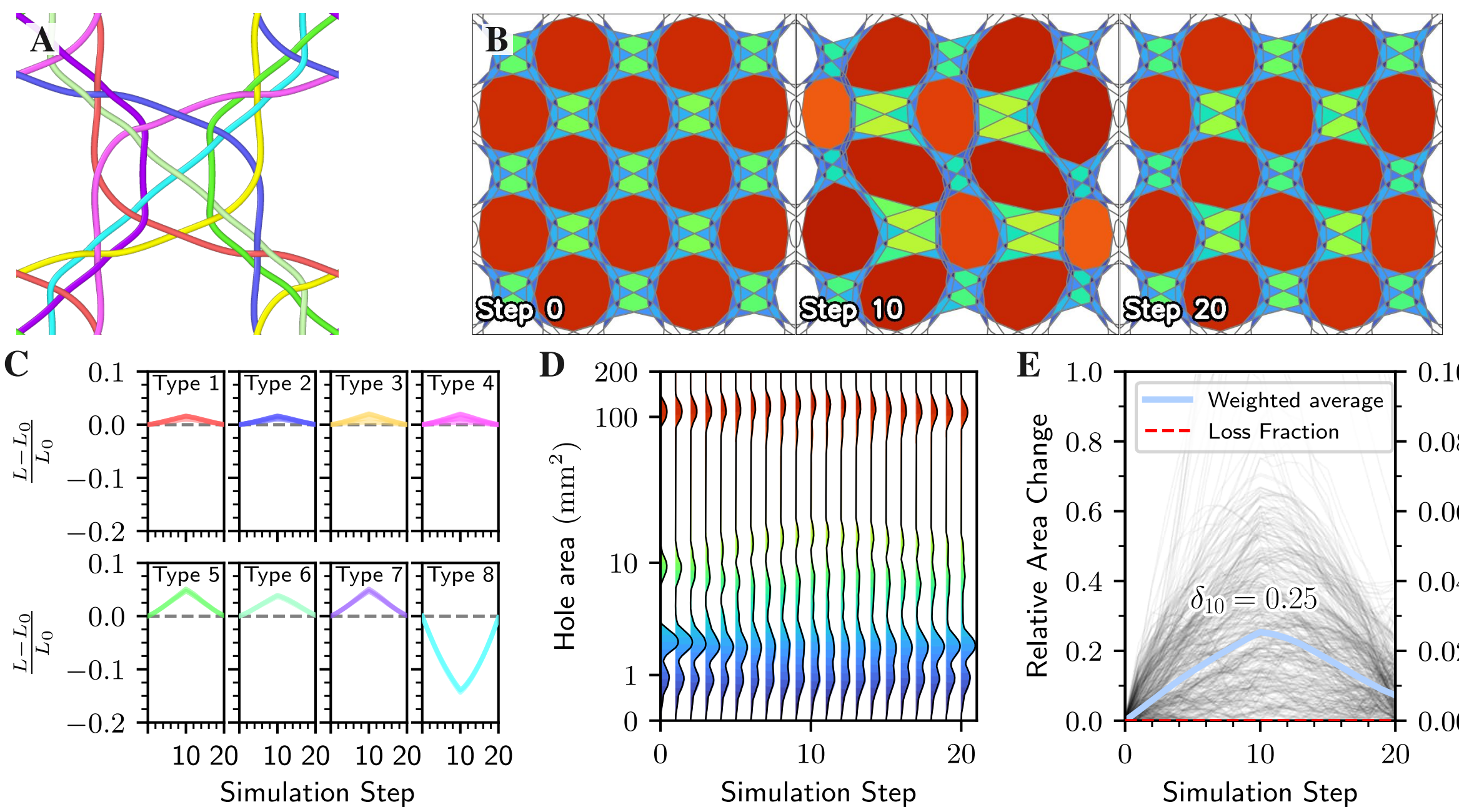

Figure S174: **Simulation of lace structure Spruce-1. A** Unit-cell schematic (active bond types {8}). **B** Snapshots at steps 0, 10, and 20, showing the initial structure, maximum deformation (active bonds shrink by 25%), and subsequent relaxation. **C** Variation of the relative length of yarns, grouped by type. **D** Evolution of the distribution of pore areas over time. **E** Relative area change of individual pores.

Textile sample details

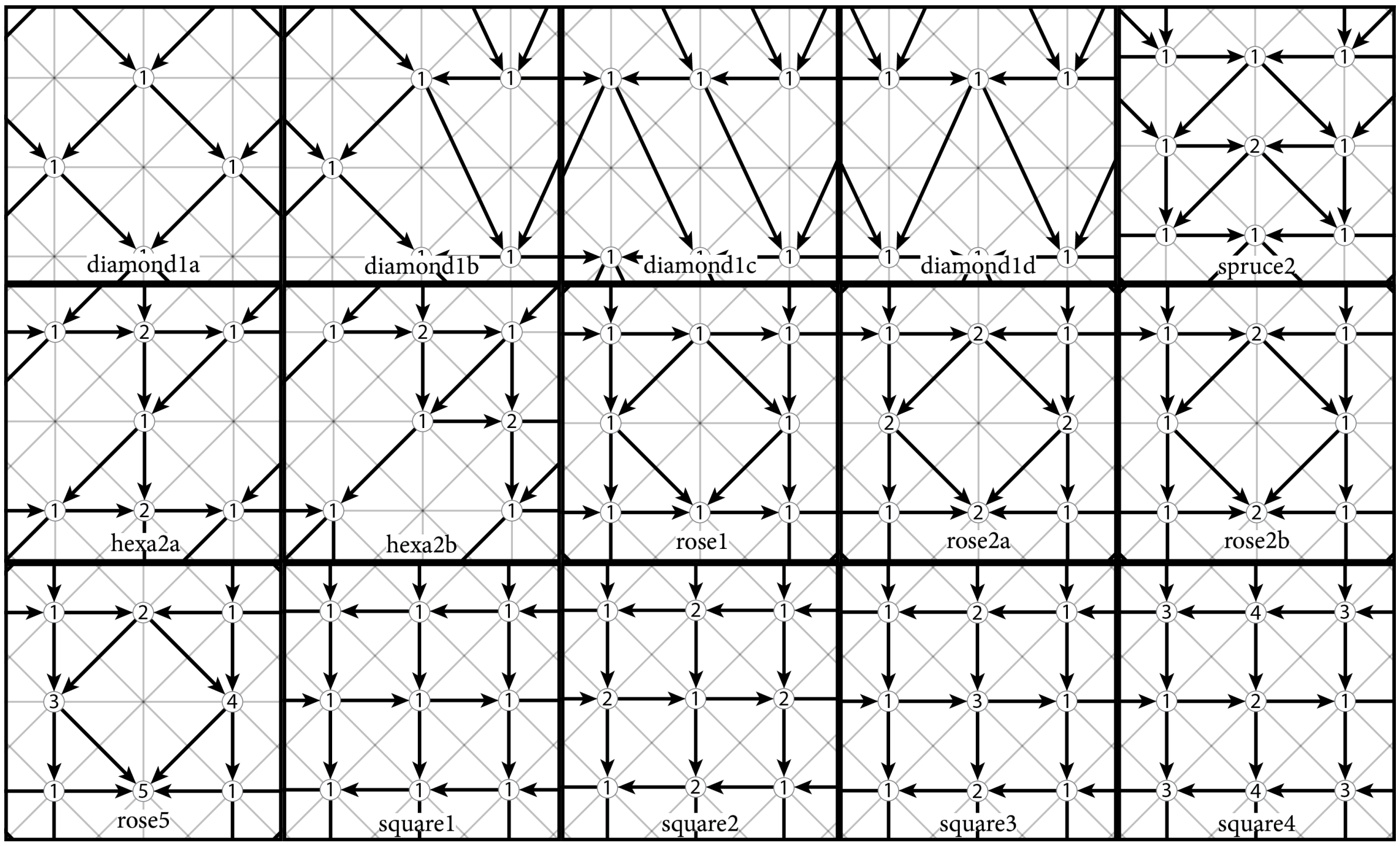

Figure S175: All used grids for simulations and textile samples.

Sample	Grid	Intersection:					Reference
		1	2	3	4	5	
Diamond-1	diamond1a	ctp					2001
Diamond-2	diamond1a	ctttp					2002
Diamond-3	diamond1a	ctcpctc					2009
Diamond-4	diamond1b	ctcpctc					2009
							modif
Diamond-5	diamond1c	ctcpctc					2009
							modif
Diamond-6	diamond1d	ctcpctc					2009
							modif
Diamond-7	diamond1a	clpcr					2023
Hexa-1	hexa2	ctpcr	c				2048
Rose-1	rose5	ctpc	ctpct	ctpcr	ctpcl	ctpc	3023
Rose-2	rose5	ctcpctc	ctpct	ctpcr	ctpcl	ctpc	3025
Rose-3	rose2a	ct	ctpct				3021
Rose-4	rose1	ctpct					3024
Rose-5	rose2a	ctctpctct	ctpct				3026
Rose-6	rose2a	tct	ctpc				3013
Rose-7	rose2a	ctc	ctpc				3018
Rose-8	rose2a	ct	ctp				3001
Rose-9	rose2a	ctpct	ct				3003
Rose-10	rose2a	ctc	ctcpctc				3034
Rose-11	rose2b	cttp	ctt				3043
Rose-12	rose5	ct	cp	rr	ll	pc	3051
							modif
Rose-13	rose5	ctpct	cp	r	l	pc	3052
Spruce-1	spruce2	ctp	ct				3059
Square-1	square1	ctcpctc					1092
							modif
Square-2	square3	ctpc	cll	crr			1084

Table S2: Stitches and grids used in textile samples. Shortenings: c = cross, t = twist, p = pin, l = twist only the left pair and r = twist only the right pair. Reference is the pattern number in the book Gründe mit System (*36*). Some patterns have been slightly modified.

Captions for supplementary movies

Caption for Movie S1. **Textile sample Rose-1 under deformation.** Sample is made of active pemotex threads, attached to the frame and heated with radiant heater. Videos analysis is shown in Figure S34.

Caption for Movie S2. **Simulation of lace structure Rose-1.** Simulated deformation and subsequent relaxation. All bond types are active. Videos analysis is shown in Figure S94

Caption for Movie S3. **Tightening filter.** Video of passing spheres, thread activation (pemotex), and spheres capture. Figure 4 A-B.

Caption for Movie S4. **Opening filter.** Video of trapped spheres, thread activation (pemotex), and spheres release. Figure 4 C-D.

Caption for Movie S5. **Textile OR gate.** Active threads drive conductive thread pairs into/out of contact to realize an OR logic element. Video with voltmeter readout toggling between OFF (0 V) and ON (1̃ V) upon recovery/activation of active threads (LCE). Videos analysis is shown in Figure 4 E-G.
